# 1,4-dihydroxy quininib activates ferroptosis pathways in metastatic uveal melanoma and reveals a novel prognostic biomarker signature

**DOI:** 10.1038/s41420-023-01773-8

**Published:** 2024-02-10

**Authors:** Valentina Tonelotto, Marcel Costa-Garcia, Eve O’Reilly, Kaelin Francis Smith, Kayleigh Slater, Eugene T. Dillon, Marzia Pendino, Catherine Higgins, Paola Sist, Rosa Bosch, Sabina Passamonti, Josep M. Piulats, Alberto Villanueva, Federica Tramer, Luca Vanella, Michelle Carey, Breandán N. Kennedy

**Affiliations:** 1 UCD Conway Institute, University College Dublin, D04 V1W8 Dublin, Ireland; 2 UCD School of Biomolecular and Biomedical Science, University College Dublin, D04 V1W8 Dublin, Ireland; 3 Medical Oncology Department, Catalan Institute of Cancer (ICO), IDIBELL-OncoBell, Barcelona, Spain; 4 Mass Spectrometry Resource, Conway Institute of Biomolecular & Biomedical Research, University College Dublin, D04 V1W8 Dublin, Ireland; 5 UCD School of Mathematics & Statistics, University College Dublin, D04 V1W8 Dublin, Ireland; 6 Department of Life Sciences, University of Trieste, 34127 Trieste, Italy; 7 Xenopat S.L., Business Bioincubator, Bellvitge Health Science Campus, 08907 L’Hospitalet de Llobregat, Barcelona, Spain; 8 Program Against Cancer Therapeutic Resistance (ProCURE), ICO, IDIBELL, Barcelona, Spain; 9 Department of Drug and Health Sciences, University of Catania, 95125 Catania, Italy; 10 CERNUT-Research Centre on Nutraceuticals and Health Products, University of Catania, 95125 Catania, Italy

**Keywords:** Metastasis, Prognostic markers

## Abstract

Uveal melanoma (UM) is an ocular cancer, with propensity for lethal liver metastases. When metastatic UM (MUM) occurs, as few as 8% of patients survive beyond two years. Efficacious treatments for MUM are urgently needed. 1,4-dihydroxy quininib, a cysteinyl leukotriene receptor 1 (CysLT_1_) antagonist, alters UM cancer hallmarks in vitro, ex vivo and in vivo. Here, we investigated the 1,4-dihydroxy quininib mechanism of action and its translational potential in MUM. Proteomic profiling of OMM2.5 cells identified proteins differentially expressed after 1,4-dihydroxy quininib treatment. Glutathione peroxidase 4 (GPX4), glutamate-cysteine ligase modifier subunit (GCLM), heme oxygenase 1 (HO-1) and 4 hydroxynonenal (4-HNE) expression were assessed by immunoblots. Biliverdin, glutathione and lipid hydroperoxide were measured biochemically. Association between the expression of a specific ferroptosis signature and UM patient survival was performed using public databases. Our data revealed that 1,4-dihydroxy quininib modulates the expression of ferroptosis markers in OMM2.5 cells. Biochemical assays validated that GPX4, biliverdin, GCLM, glutathione and lipid hydroperoxide were significantly altered. HO-1 and 4-HNE levels were significantly increased in MUM tumor explants from orthotopic patient-derived xenografts (OPDX). Expression of genes inhibiting ferroptosis is significantly increased in UM patients with chromosome 3 monosomy. We identified IFerr, a novel ferroptosis signature correlating with UM patient survival. Altogether, we demontrated that in MUM cells and tissues, 1,4-dihydroxy quininib modulates key markers that induce ferroptosis, a relatively new type of cell death driven by iron-dependent peroxidation of phospholipids. Furthermore, we showed that high expression of specific genes inhibiting ferroptosis is associated with a worse UM prognosis, thus, the IFerr signature is a potential prognosticator for which patients develop MUM. All in all, ferroptosis has potential as a clinical biomarker and therapeutic target for MUM.

## Introduction

Uveal melanoma (UM) is a rare eye tumor with a global prevalence of 1-9/1,000,000 (www.orpha.net). UM is the most common primary intraocular malignancy in adults, arising from uveal melanocytes [1]. Hematogenously, UM metastasizes in ~50% of patients, most frequently to the liver [1]. Primary UM can be treated by surgery or radiation [2], but therapeutic options for metastatic UM (MUM) patients are very limited. MUM patients have a median survival time of only 6-12 months [3]; around 8% of MUM patients survive beyond two years [4]. Recently, Tebentafusp (Kimmtrak^(R)^), a bispecific fusion protein that redirects CD3 + T cells to target glycoprotein 100-positive melanoma cells, was reported to improve overall MUM survival by 6 months [5, 6]. Tebentafusp is approved by the United States Food and Drug Administration and by the European Medicines Agency for the treatment of HLA-A*02:01-positive adults with unresectable or metastatic UM [5]. Therefore, only a subcategory of MUM patients is eligible for Tebentafusp treatment, underlying the need for additional, more effective treatments.

Activating mutations in *GNAQ* or *GNA11* occur in ~83% of UMs [7], while mutations in *CYSLTR2* or *PLCB4* occur in ~10% of cases [8, 9]. Cysteinyl leukotriene receptors 1 (CysLT_1_) and 2 (CysLT_2_), are G protein-coupled receptors, which signal to downstream effectors, such as phospholipase C-β (PLCβ), protein kinase C (PKC), ADP-ribosylation factor 6 (ARF6) and β-catenin. These cascades modulate pathways including mitogen-activated protein kinase (MAPK), PI3K/AKT, and Rho GTPase [10]. Upstream, the receptors are activated by cysteinyl leukotrienes (CysLTs), inflammatory lipid mediators synthesized through the 5-lipoxygenase (5-LO) pathway [11]. A role for CysLTs in cancer has recently emerged. In retrospective analyses, CysLT_1_ antagonists showed a dose-dependent chemo-preventative effect against 14 cancers and an overall decreased risk of cancer [12, 13]. Overexpression of CysLT_1_ is observed in colorectal cancer, renal cell carcinoma, breast cancer and UM [14–17]. High expression of the CysLT receptors genes, *CYSLTR1* and *CYSLTR2*, is significantly associated with poor disease-free survival (DFS) and poor overall survival (OS) in UM patients [17, 18]. The CysLT_1_ antagonist quininib and its analog 1,4-dihydroxy quininib significantly alter viability, long-term proliferation, secretion of inflammatory and angiogenic factors, and oxidative phosphorylation in primary and metastatic UM cell lines [17]. CysLT_1_ antagonists also significantly inhibit tumor burden in zebrafish xenograft models of UM [17]. Furthermore, in tumors from a cell line-derived mouse orthotopic xenograft model of MUM, 1,4-dihydroxy quininib significantly decreases expression levels of ATP synthase F1 β subunit (ATP5B), a protein marker of oxidative phosphorylation [18]. Notably, high expression of *ATP5F1B* in primary UM is significantly associated with reduced progression-free survival and reduced OS, and patients with disomy 3 and low *ATP5F1B* expression have a reduced risk of metastatic disease [18]. MUM prognosis is still very challenging, with only 1% of patients displaying metastases at the time of primary UM diagnosis [19]. Thus, a deeper understanding of 1,4-dihydroxy quininib molecular mechanisms may also support the discovery of novel prognostication biomarkers for UM patients.

In this study, we elucidated for the first time that 1,4-dihydroxy quininib modulates key ferroptosis hallmarks in MUM cell lines and in MUM tumor explants from orthotopic patient-derived xenografts (OPDX) models. Ferroptosis is a form of regulated cell death characterized by iron-dependent lipid peroxidation [20], which has emerged as a new player in anticancer therapies [21–23]. Indeed, it can inhibit cancer cell growth, improve the sensitivity of chemotherapy and radiotherapy, and inhibit distant metastases [24]. Cancer cells may enhance their oxidative stress defence ability by inhibiting ferroptosis, suggesting that induction of ferroptosis can be exploited to overcome ineffectiveness or resistance to conventional therapies. Furthermore, suitable companion biomarkers may improve clinical monitoring in cancer patients [22–26]. Importantly, several recent studies highlight ferroptosis as a new treatment target for MUM [27, 28].

Ferroptosis is morphologically and biochemically distinct from autophagy, apoptosis, necrosis, and necroptosis [29]. Core ferroptosis hallmarks include: i) loss of lipid peroxide repair capacity through glutathione peroxidase 4 (GPX4) [30, 31], ii) bioavailability of redox-active iron [32, 33], and iii) oxidation of polyunsaturated fatty acid (PUFA)-containing phospholipids and accumulation of lipid oxidation products *e.g*. 4-HNE [34–36]. The nuclear factor erythroid 2-related factor (NRF2) plays a key role in redox homeostasis [37]. One of its major targets, heme oxygenase 1 (HO-1), is an antioxidant and detoxifying gene, which can exert either a cytoprotective or detrimental action in cancer, based on the specific cellular conditions [38–42].

Here, we used an unbiased proteomic profiling approach to understand the “global” molecular changes induced by 1,4 dihydroxy quininib. We showed that 20 µM 1,4-dihydroxy quininib modulates specific ferroptosis hallmarks in MUM cells in a time-dependent manner, with the activation of the NRF2/HO-1 axis after 4 or 8 h treatment and the inhibition of GPX4 expression after 24 h. Importantly, 1,4-dihydroxy quininib also significantly increases HO-1 and 4-HNE levels in MUM tumor explants derived from OPDX mouse models. Finally, we identified IFerr, a ferroptosis gene signature, which correlates with UM patient survival. Overall, this work is significant since it supports ferroptosis as a potential target for the treatment and as a prognostication biomarker for MUM, and provides an opportunity to enhance the clinical management of this devastating disease.

## Results

### 1,4-dihydroxy quininib significantly modulates proteomic markers of ferroptosis in OMM2.5 cells

**Fig. 1:**
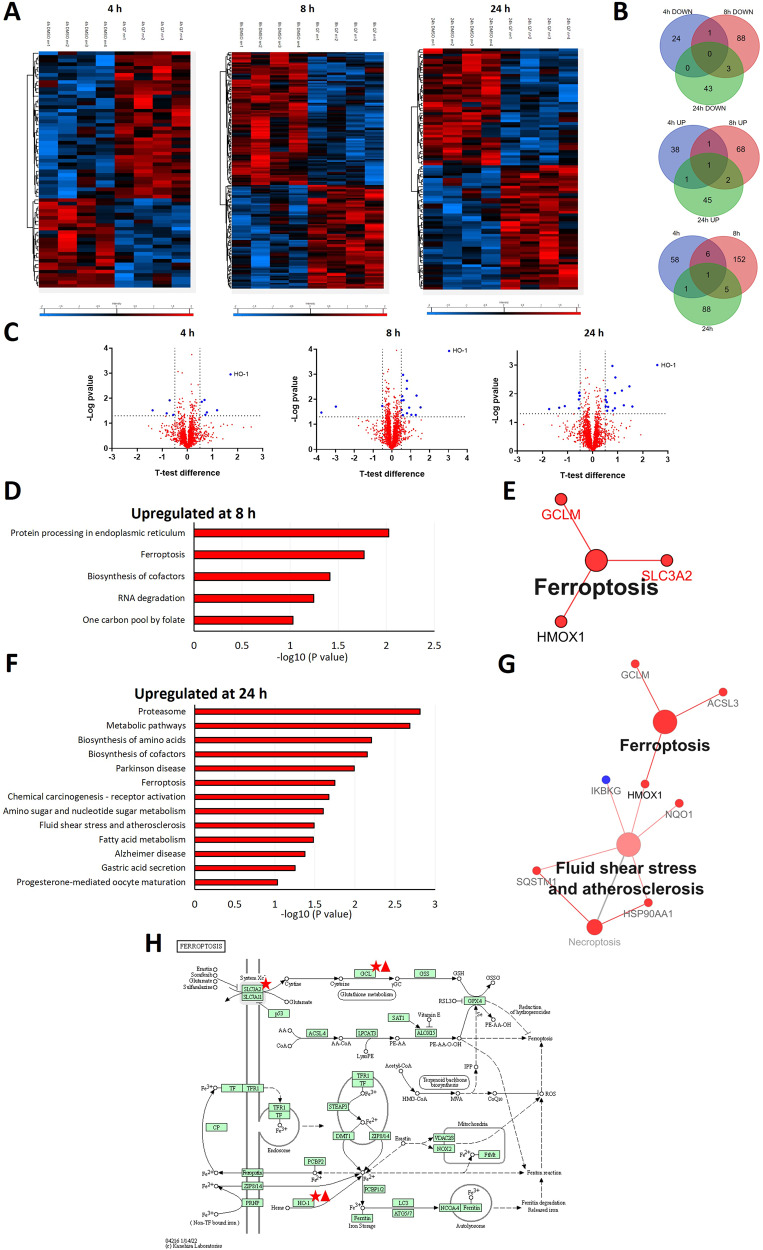
Analysis of proteome profiling uncovered ferroptosis as a biological process affected by 20 µM 1,4 dihydroxy quininib in OMM2.5 cells. **A** Heatmap chart depicting all the differentially expressed proteins between 0.5% DMSO and 20 µM 1,4 dihydroxy quininib (Q7) treated OMM2.5 cells after 4, 8 or 24 h of treatment. The heat maps show *n* = 4 for each time point with the respective colour scale located below each figure. The blue and red refer to down-regulated and upregulated proteins, respectively. The 4 h heat map (left panel) represents 66 differentially expressed proteins where 41 proteins are upregulated and 25 proteins are downregulated. The 8 h heat map (middle panel) represents 164 differentially expressed proteins where 72 proteins are upregulated and 92 proteins are downregulated. The 24 h heat map (right panel) represents 95 differentially expressed proteins where 49 proteins are upregulated and 46 proteins are downregulated. **B** Venn diagram analyses showing the unique and shared downregulated proteins between 0.5% DMSO and 20 µM Q7 treated OMM2.5 cells at 4, 8 and 24 h (upper panel), the unique and shared upregulated proteins between 0.5% DMSO and 20 µM Q7 treated OMM2.5 cells at 4, 8 and 24 h (middle panel), and the total amount of unique and shared differentially expressed proteins between 0.5% DMSO and 20 µM Q7 treated OMM2.5 cells at 4, 8 and 24 h (lower panel). **C** Volcano plots depicting the differentially expressed proteins between 0.5% DMSO and 20 µM Q7 treated OMM2.5 cells at 4 h (left panel), 8 h (middle panel) and 24 h (right panel). The proteins highlighted in red represent the proteins with a Student’s T-Test Difference ≥ 0.5. The proteins highlighted in blue represent those with a Student’s T-Test Difference ≤ − 0.5. The most consistently upregulated protein after 4, 8 or 24 h of treatment is heme oxygenase 1 (HO-1). **D**–**H** Gene ontology (GO) classification of proteomic data for differentially expressed proteins between 0.5% DMSO and 20 µM Q7-treated OMM2.5 cells after 8 h (**D**, **E**) and 24 h (**F**, **G**) of treatment. **D** KEGG pathway analysis of significantly upregulated proteins in 20 µM Q7-treated vs 0.5% DMSO-treated OMM2.5 cells after 8 h of treatment, showing the most enriched categories in biological process. **E** Protein-protein interaction network showing the significantly upregulated proteins in 20 µM Q7-treated vs 0.5% DMSO-treated OMM2.5 cells associated to ferroptosis, after 8 h of treatment. **F** KEGG pathway analysis of significantly upregulated proteins in 20 µM Q7-treated vs 0.5% DMSO-treated OMM2.5 cells after 24 h of treatment, showing the most enriched categories in biological process. **G** Protein-protein interaction network showing the significantly upregulated proteins in 20 µM Q7-treated vs 0.5% DMSO-treated OMM2.5 cells associated to ferroptosis, after 24 h of treatment. The panel also shows upregulated (red nodes) and downregulated (blue node) proteins associated to fluid shear stress and atherosclerosis process. HO-1 upregulation is common to both pathways. (**H**) DAVID pathway analysis displaying significantly upregulated proteins at 8 h (red stars) and 24 h (red triangles) in 20 µM Q7-treated vs 0.5% DMSO-treated OMM2.5 cells in ferroptosis process. Image obtained from KEGG [102]. Q7 = 1,4-dihydroxy quininib; HMOX1 = HO-1; h = hours.

**Fig. 2:**
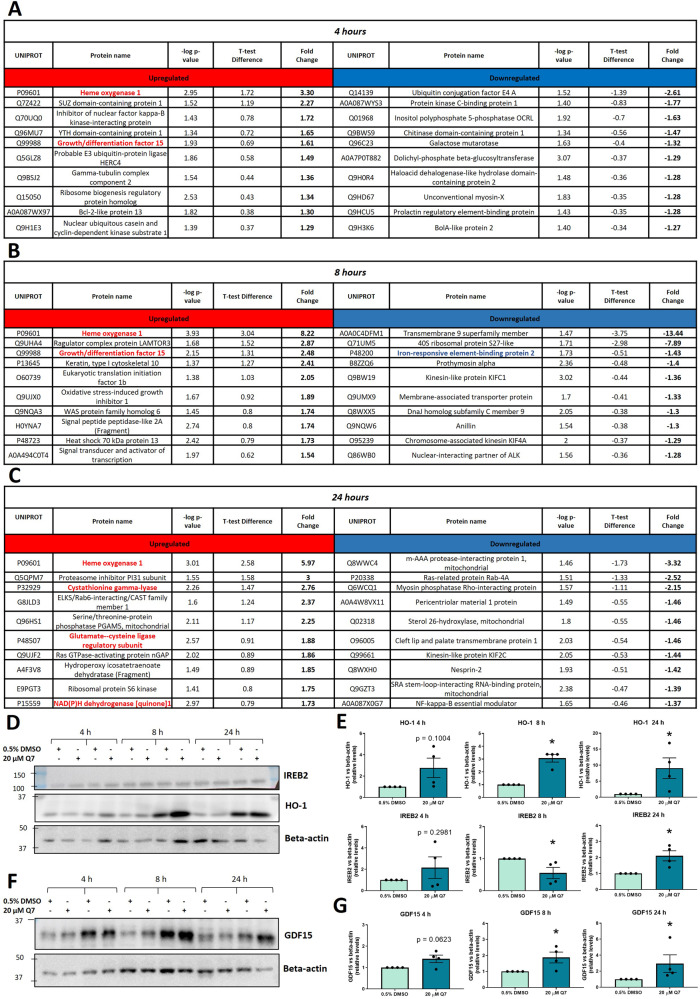
Most significantly altered proteins identified by proteomic profiling following 1,4 dihydroxy quininib treatment in OMM2.5 cells and immunoblot validation. **A** Table showing the 10 most up- and down-regulated proteins in OMM2.5 cells after 4 h of treatment with 20 µM Q7. **B** Table showing the 10 most up- and down-regulated proteins in OMM2.5 cells after 8 h of treatment with 20 µM Q7. **C** Table showing the 10 most up- downregulated proteins in OMM2.5 cells after 24 h of treatment with 20 µM Q7. Protein names coloured in red and blue in tables **A**, **B** and **C** are Q7-upregulated and downregulated proteins, respectively, known to be associated to the ferroptosis pathway. **D** Immunoblot analysis of HO-1 and IREB2 in protein extracts from OMM2.5 cells treated with 0.5% DMSO or 20 µM Q7 for 4, 8 or 24 h. **E** Densitometric quantification of HO-1 and IREB2 normalized to beta-actin, as determined by *n* = 4 independent western blot experiments as in **D** (*, *p* < 0.05). **F** Immunoblot analysis of GDF-15 in protein extracts from OMM2.5 cells treated with 0.5% DMSO or 20 µM Q7 OMM2.5 cells for 4, 8 or 24 h. **G** Densitometric quantification of GDF15 normalized to beta-actin, as determined by at least three independent western blot experiments as in **F** (*, *p* < 0.05). The histograms report mean ± SEM. Q7 = 1,4-dihydroxy quininib; h = hours.

To understand the molecular changes induced by 1,4-dihydroxy quininib in OMM2.5 cells, proteome profiling was performed on whole cell extracts at 4, 8 and 24 h post-treatment (hpt) (Fig. 1, Supplementary Dataset 1). A total of 4381 proteins were detected across all samples. Applying stringency cut-offs of *p* value < 0.05 and fold-change > ± 1.2, at 4 hpt 66 differentially expressed proteins (DEPs) were identified, with 41 proteins significantly upregulated and 25 proteins significantly downregulated. At 8 hpt, 164 DEPs were detected: 72 significantly upregulated and 92 significantly downregulated. 95 DEPs were uncovered at 24 hpt, with 49 significantly upregulated and 46 significantly downregulated proteins (Fig. 1A–C). Additional analysis revealed 157 proteins showing a significant time-dependent expression upon 1,4-dihydroxy quininib treatment (Supplementary Fig. 1). The most consistently upregulated protein after 4, 8 or 24 h treatment was heme oxygenase 1 (HO-1), with changes of 3.3, 8.2 or 6-fold respectively (Figs. 1C, 2A–E). The HO-1 enzyme catabolizes cellular heme to biliverdin, carbon monoxide, and free iron [43, 44]. HO-1 has dual roles in cancer cells, since in several malignant human neoplastic diseases it promotes cell growth, proliferation and invasion [45, 46], but in other cancers it displays anti-tumoral effects [39, 47].

KEGG pathway analysis revealed that at 8 and 24 hp, upregulated DEPs were associated with specific cellular processes, including *protein processing in endoplasmic reticulum, ferroptosis, biosynthesis of cofactors, RNA degradation, one carbon pool by folate* at 8 hpt (Fig. 1D, E) and *proteasome, metabolic pathways, biosynthesis of amino acids, biosynthesis of cofactors, parkinson disease, ferroptosis, chemical carcinogenesis - receptor activation, amino sugar and nucleotide sugar metabolism, fluid shear stress and atherosclerosis, fatty acid metabolism, alzheimer disease, gastric acid secretion, and progesterone-mediated oocyte maturation* at 24 hpt (Fig. 1F, G). Interestingly, the analysis highlighted that ferroptosis upregulation was present at both 8 and 24 hpt with 1,4-dihydroxy quininib (Fig. 1D–H). Protein-protein interaction network analysis elucidated which significantly DEPs were associated with ferroptosis, including HO-1 (Fig. 1E, G) which mediates protective or detrimental effects via ferroptosis induction [38–42]. At 8 hpt, additional DEPs linked to ferroptosis were solute carrier family 3 member 2 (SCL3A2) and glutamate-cysteine ligase regulatory subunit (GCLM) (Fig. 1E). HO-1 regulation was also associated to *fluid shear stress* and *atherosclerosis* processes at 24 hpt (Fig. 1G). GCLM was differentially expressed after 1,4-dihydroxy quininib treatment also at 24 hpt, together with the ferroptosis marker acyl-CoA synthetase long-chain family member 3 (ACSL3) (Fig. 1G).

To corroborate the proteomics data, we selected some of the 10 most DEPs after 1,4-dihydroxy quininib treatment and performed immunoblotting (Fig. 2A–C). Significant HO-1 upregulation at 8 and 24 hpt (*p* = 0.0286 and *p* = 0.0463, respectively), iron-responsive element binding protein 2 (IREB2) downregulation at 8 hpt (*p* = 0.0412) and growth/differentiation factor-15 (GDF15) upregulation at 8 hpt (*p* = 0.0416) were observed (Fig. 2D–G). Intriguingly, IREB2 and GDF15 have been previously described as ferroptosis modulators [36, 48].

### 1,4-dihydroxy quininib modulates the NRF2/HO-1 and the GSH-GPX4-LOOH axes in OMM2.5 cells in a time dependent manner

**Fig. 3:**
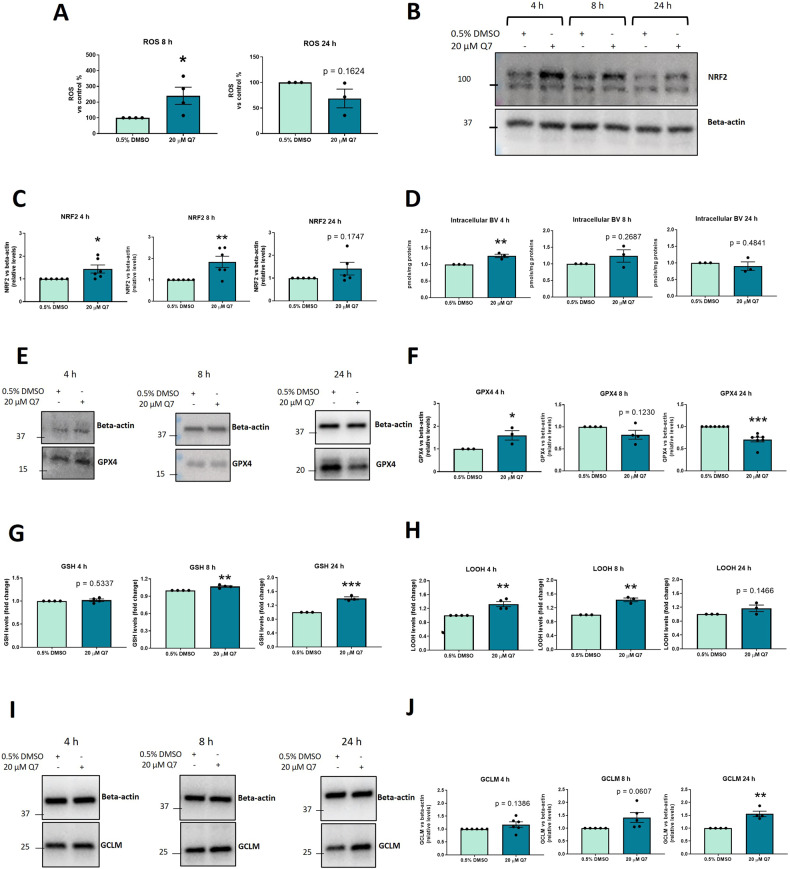
Treatment with 20 µM 1,4 dihydroxy quininib affects ROS, biliverdin, GSH, LOOH levels and NRF2, GPX4, GCLM expression in OMM2.5 cells. **A** Evaluation of the effects of 20 µM Q7 on ROS levels in OMM2.5 cells after 8 (*n* = 4 independent experiments) and 24 (*n* = 3 independent experiments) h of treatment (*p < 0.05). **B** Western blot analysis of NRF2 expression in 0.5% DMSO or 20 µM Q7 treated OMM2.5 cells after 4 (n = 6 independent experiments), 8 (*n* = 6 independent experiments) and 24 (*n* = 5 independent experiments) h of treatment (*, *p* < 0.05; ** *p* < 0.01). **C** Densitometric quantification of NRF2 vs beta-actin as determined by at least three independent western blot experiments as in **B**. The results are expressed as means ± SEM. **D** Measurement of intracellular biliverdin in 0.5% DMSO- or 20 µM Q7- treated OMM2.5 cells after 4, 8 and 24 h of treatment (** *p* < 0.01). N = 3 independent experiments. **E** Western blot showing GPX4 expression in 0.5% DMSO- or 20 µM Q7- treated OMM2.5 cells after 4 (*n* = 3 independent experiments), 8 (*n* = 4 independent experiments) and 24 (*n* = 7 independent experiments) h. **F** Densitometric quantification of GPX4 vs beta-actin as determined by at least three independent western blot experiments as in **E** (*p < 0.05; ***p < 0.001). **G** Measurement of non-protein thiol group (RSH) concentrations after 4 (*n* = 4 independent experiments), 8 (n = 4 independent experiments) and 24 (*n* = 3 independent experiments) h of treatment (**p < 0.01; ***p < 0.001). **H** Evaluation of LOOH levels after 4 (*n* = 4 independent experiments), 8 (*n* = 4 independent experiments) and 24 (*n* = 3 independent experiments) h of treatment (**, *p* < 0.01). The results are expressed as means ± SEM. **I** Western blot showing GCLM expression in 0.5% DMSO- or 20 µM Q7- treated OMM2.5 cells after 4 (*n* = 6 independent experiments), 8 (*n* = 5 independent experiments), and 24 (*n* = 4 independent experiments) hours. **J** Densitometric quantification of GCLM normalized to beta-actin as determined by at least three independent western blot experiments as in **I** (** *p* < 0.01). The histograms report mean ± SEM. Q7 = 1,4-dihydroxy quininib; h = hours.

Since HO-1 expression is regulated by NRF2, a transcription factor involved in cellular detoxification processes [37, 48–50], we investigated whether HO-1 upregulation correlated to increased reactive oxygen species (ROS) and NRF2 levels. ROS levels significantly increased after 8 h of 1,4-dihydroxy quininib treatment (*p* = 0.0419), but did not change following the 24 h one (*p* = 0.1624) (Fig. 3A). Interestingly, increased ROS amount after 8 h was associated with a significant (*p* = 0.0096) increase in phosphorylated NRF2. This form of NRF2 preferentially localizes in the nucleus [51], and binds within regulatory regions of target genes (e.g., HO-1, glutamate-cysteine ligase catalytic subunit (GCLC), GCLM, multidrug-resistant proteins (MRPs), and sequestosome-1 protein (p62)) [37]. Phosphorylated NRF2 was also significantly increased after 4 h of treatment (*p* = 0.0337), while no significant change was observed with the 24 h-treatment (Fig. 3B, C).

To evaluate whether increased HO-1 expression correlated to increased HO-1 enzymatic activity, biliverdin levels were measured (Fig. 3D) [52, 53]. Intriguingly, we detected a significant increase in biliverdin levels following 4 h of treatment (*p* = 0.0083), while no differences were observed with the 8 or 24-h treatment (Fig. 3D).

We then investigated whether 1,4-dihydroxy quininib modulates additional ferroptosis hallmarks in OMM2.5 cells. Western blot analysis revealed significantly (*p* = 0.0444) increased GPX4 expression after 4 h of 1,4-dihydroxy quininib treatment, while the 24-h treatment significantly decreased GPX4 levels (*p* = 0.0006) (Fig. 3E, F). No statistically significant differences were observed after 8 h of treatment (Fig. 3E, F). 1,4-dihydroxy quininib did not affect glutathione (GSH) content after 4 h of treatment, but led to a significant increase in GSH levels when administered to OMM2.5 cells for 8 or 24 h (*p* = 0.0023; *p* = 0.0008, respectively) (Fig. 3G). Concurrently, lipid hydroperoxide (LOOH) levels were significantly upregulated following 4 (*p* = 0.0043) and 8 (*p* = 0.0013) h of 1,4-dihydroxy quininib treatment, but not after 24 h (Fig. 3H). The proteomic analysis uncovered an upregulation of GCLM (Fig. 1E, G, Supplementary Dataset 1), a regulatory subunit of the glutamate cysteine ligase (GCL), the rate-limiting enzyme in GSH synthesis [54, 55]. Increased GCLM expression enhances capacity for GSH synthesis [56, 57]. In agreement, western blot analysis showed a significant increase in GCLM levels after 24 h of 1,4-dihydroxy quininib (*p* = 0.022) and a slight, but not significant (*p* = 0.0607), increase after the 8 h-treatment. No significant changes were observed at 4 h (Fig. 3I, J). Therefore, the increase in GSH content after 8 and 24 h may be related to an upregulation in GCLM expression, as a consequence of the prolonged treatments.

Together these results suggest that 1,4-dihydroxy quininib activates the NRF2/HO-1 axis in OMM2.5 cells after 4 and 8 h of treatment, with a significant increase in the expression of phosphorylated NRF2 and HO-1 at 4 and 8 h and in the amount of intracellular biliverdin following a short treatment (4 h). In addition, 1,4-dihydroxy quininib alters redox homeostasis, leading to lipid peroxidation and a concomitant upregulation of GPX4 expression after 4 h of treatment. Interestingly, lipid peroxidation was not present following the 24 h-treatment, but a significant decrease in GPX4 expression and an accumulation of GSH were detected, highlighting how 1,4-dihydroxy quininib modulates specific ferroptosis hallmarks depending on the duration of treatment. Notably, a significant increase in HO-1 expression (*p* = 0.0167) and a significant decrease (*p* = 0.0102) in GPX4 expression were observed also in Mel285 cells, a primary UM cell line, supporting the ability of 1,4-dihydroxy quininib to modulate ferroptosis also in primary UM cells (Supplementary Fig. 2).

### CysLT_1_ antagonists and CysLT_2_ antagonist have overlapping and distinct effects on specific ferroptosis markers, while erastin reduces OMM2.5 cells metabolic activity

**Fig. 4:**
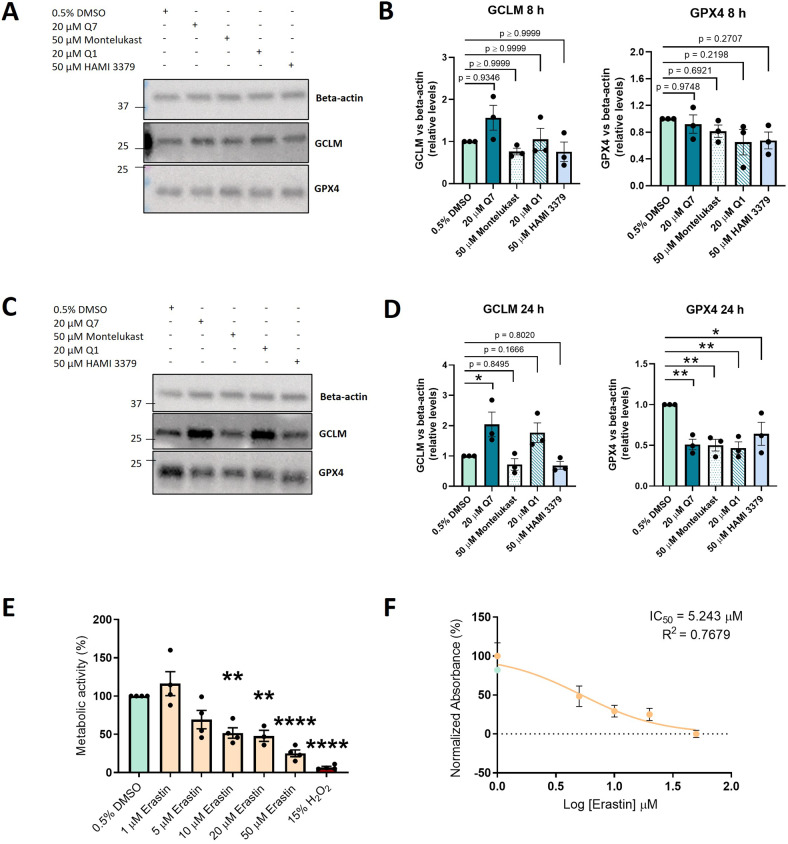
Quininib drugs and CysLT receptor antagonists exert overlapping and distinct effects on ferroptosis markers, while erastin reduces OMM2.5 cells metabolic activity. **A** Western blot showing GPX4 and GCLM expression in 0.5% DMSO- or 20 µM Q7-, 50 µM montelukast-, 20 µM quininib-, 50 µM HAMI 3379- treated OMM2.5 cells after 8 h of treatment. **B** Densitometric quantification of GCLM (left) and GPX4 (right) vs beta-actin as determined by three independent western blot experiments as in **A**. **C** Western blot showing GPX4 and GCLM expression in 0.5% DMSO- or 20 µM Q7-, 50 µM montelukast-, 20 µM quininib-, 50 µM HAMI 3379- treated OMM2.5 cells after 24 h of treatment. **D** Densitometric quantification of GCLM (left) and GPX4 (right) vs. beta-actin as determined by three independent western blot experiments as in **C** (*, *p* < 0.05; ** *p* < 0.01). **E** A significant dose-dependent decrease of OMM2.5 cell metabolic activity was observed following 96 h of erastin treatment in comparison to 0.5% DMSO control. *N* = 3-4 independent experiments **F** IC_50_ value of erastin in OMM2.5 cell metabolic activity assays. Metabolic activity of cells was determined using MTT (3-(4,5-dimethylthiazol-2-yl)-2,5-diphenyltetrazolium bromide) assay. All the data are expressed as mean ± SEM (**p < 0.01; ****p < 0.0001). Q1 = quininib; Q7 = 1,4-dihydroxy quininib; h = hours.

To investigate whether other CysLT receptor antagonists modulate ferroptosis in OMM2.5 cells, we tested CysLT_1_ antagonists quininib [58] and montelukast [59] and CysLT_2_ antagonist HAMI 3379 [60] and analysed GPX4 and GCLM expression levels (Fig. 4A–D). 50 µM montelukast, 20 µM quininib and 50 µM HAMI 3379 did not significantly affect GCLM or GPX4 expression after 8 h, similarly to 1,4-dihydroxy quininib (Fig. 4A, B). Intriguingly, while 20 µM 1,4-dihydroxy quininib, 50 µM montelukast, 20 µM quininib and 50 µM HAMI 3379 all significantly reduced GPX4 expression (*p* = 0.0066, *p* = 0.0059, *p* = 0.00390, *p* = 0.0395, respectively) at 24 h, only 20 µM 1,4-dihydroxy quininib significantly increased GCLM expression (*p* = 0.0496) (Fig. 4C, D). These results suggest that 1,4-dihydroxy quininib modulates the GSH/GPX4 axis in OMM2.5 cells differently from other CysLT_1_ or CysLT_2_ antagonists, highlighting a unique mechanism of action in these cells.

We next evaluated whether erastin, an established ferroptosis inducer, exerts anti-cancer effects on OMM2.5 cells (Fig. 4E, F). Significant dose-dependent reductions of cells metabolic activity were observed following 96 h of 10 μM (*p* = 0.0035), 20 μM (*p* = 0.0036) or 50 μM (*p* < 0.0001) erastin treatment in comparison to vehicle control (Fig. 4E), with a calculated IC_50_ of 5.243 μM (Fig. 4F).

### 1,4-dihydroxy quininib significantly increases HO-1 and 4-HNE levels in tumors from orthotopic patient-derived xenograft MUM mouse models

**Fig. 5:**
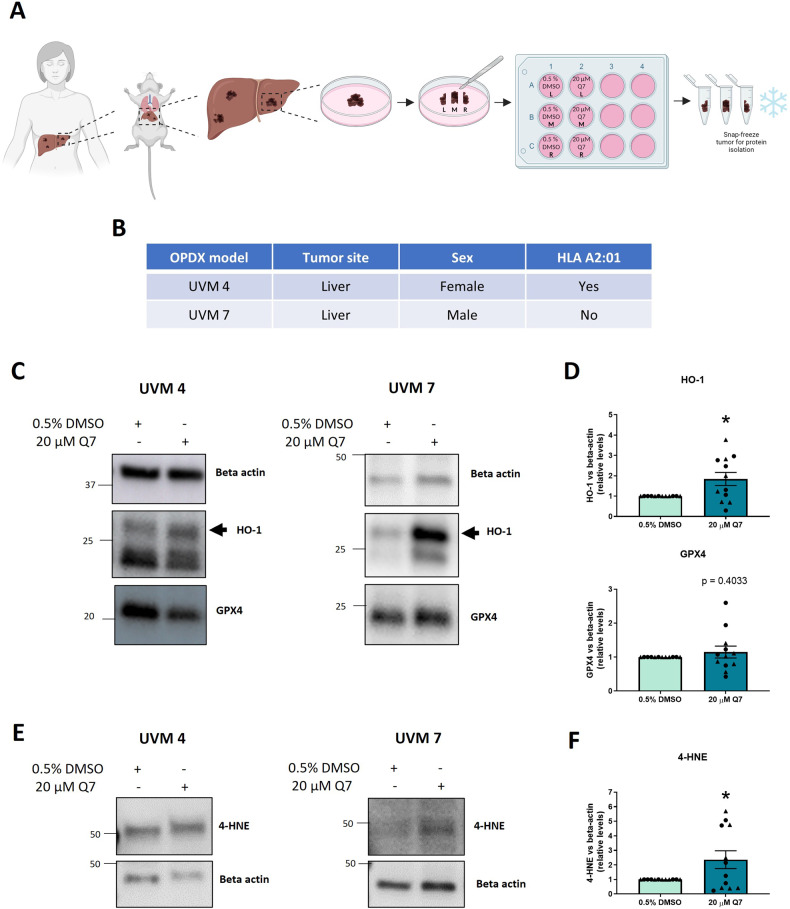
1,4-dihydroxy quininib significantly increases HO-1 and 4-HNE expression in MUM OPDX explants. **A** Schematic of the explant culture protocol to evaluate 1,4 dihydroxy quininib effects. Rodent xenograft models of MUM have been generated by transplanting fresh patient’s tumor samples into the liver of mouse models. MUM tumours were removed from mouse liver and then dissected into 3 fragments, arbitrarily named left (L), middle (M), right (R). Each fragment was divided into 2 pieces and grown in complete media with 20 μM 1,4-dihydroxy quininib (Q7) or vehicle (0.5% DMSO) for 72 h. Tumour tissue was snap-frozen for protein isolation and western blot experiments. **B** Table showing the 2 MUM patients’ clinical characteristics. Tumors from these patients were used to generate OPDX mouse models, named UVM 4 and UVM 7. **C** Western blot analysis of HO-1 and GPX4 expression in 0.5% DMSO- or 20 µM Q7- treated MUM tumours cultured ex vivo for 72 h (*p < 0.05). **D** Densitometric quantification of HO-1 and GPX4 vs beta-actin as determined by at least three independent western blot experiments as in **C** (*p < 0.05). Data represent the mean ± SEM. *N* = 3 MUM OPDX models for each type of tumor, *i.e*., tumors from 3 UVM4 and 3 UVM7 mouse models. For each tumor fragments L and R were used to perform western blot. Triangles represent data from UVM 4 and circles represent data from UVM 7. **E** Western blot analysis of 4-HNE expression in 0.5% DMSO- or 20 µM Q7- treated MUM tumours cultured ex vivo for 72 h (*p < 0.05). **F** Densitometric quantification of 4-HNE vs beta-actin as determined by at least three independent western blot experiments as in **E** (*p < 0.05). Data represent the mean ± SEM. *N* = 3 MUM OPDX models for each type of tumor, i.e., tumors from 3 UVM4 and 3 UVM7 mouse models. For each tumor fragments L and R were used to perform Western blot. Triangles represent data from UVM 4 and circles represent data from UVM 7. Q7 = 1,4-dihydroxy quininib.

To translate these in vitro findings into more clinically relevant UM tissues, we analyzed the effects of 1,4-dihydroxy quininib on MUM OPDX (Fig. 5) obtained from a HLA-A*02:01-positive female (UVM4) and a HLA-A*02:01-negative male (UVM7) patients (Fig. 5A, B). MUM OPDX tumor samples grown in the mice liver were dissected into explant pieces, arbitrarily renamed as left (L), middle (M) or right (R) (Fig. 5A). Western blot data showed a significant upregulation of HO-1 expression (*p* = 0.0151) (Fig. 5C, D), no significant difference in GPX4 expression (*p* = 0.4033) (Fig. 5C, D) and a significant increase in 4-HNE levels (*p* = 0.0371) (Fig. 5E, F) after 1,4-dihydroxy quininib treatment of MUM tissues from both UVM4 and UVM7. 4-HNE is a reactive aldehyde derived from the oxidative cleavage of lipid peroxides. Therefore, the data suggest that 1,4-dihydroxy quininib induces ferroptosis pathways in patient-derived MUM explants.

### High expression of genes inhibiting ferroptosis correlates with reduced OS and DFS in primary UM patients

**Fig. 6:**
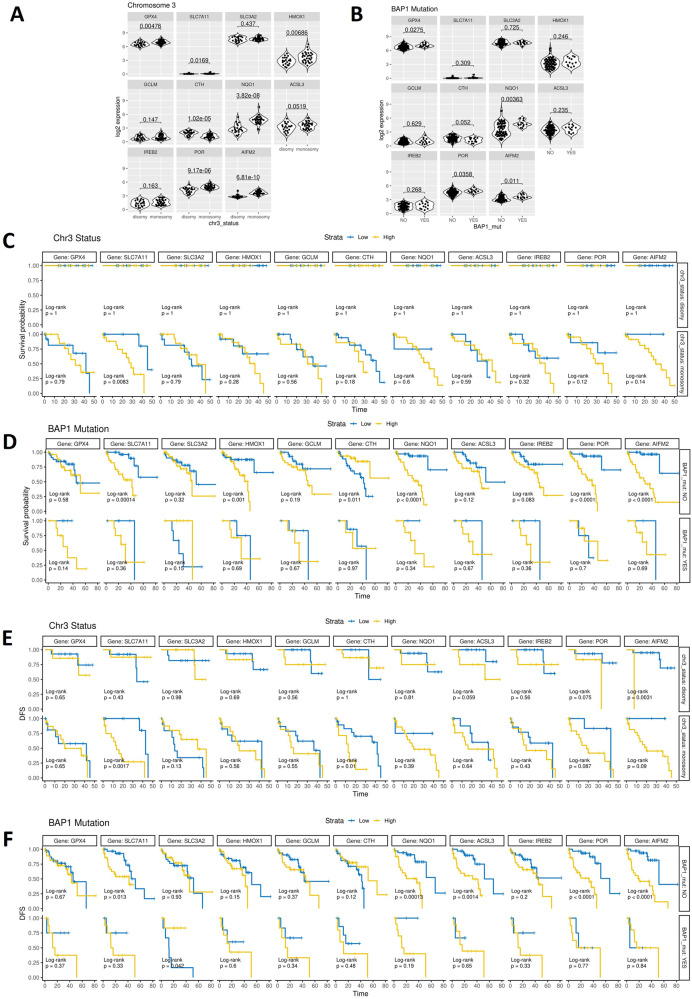
High expression levels of key genes involved in ferroptosis modulation are associated with a worse prognosis in the TCGA-UM cohort. **A** Graphs showing the statistically significant relationship between expression levels of *GPX4, SCL7A11, SLC3A2, HMOX1, GCLM, CTH, NQO1, ACSL3, IREB2, POR, AIFM2* and disomy 3 or monosomy 3 in the TCGA-UM cohort. **B** Graphs showing the statistically significant relationship between expression levels of *GPX4, SCL7A11, SLC3A2, HMOX1, GCLM, CTH, NQO1, ACSL3, IREB2, POR, AIFM2* and the normal BAP1 (BAP1_mut NO) or mutated BAP1 (BAP1_mut YES) TCGA-UM cohort. **(C)** Kaplan–Meier survival curves showing the statistically significant relationship between the expression levels of *GPX4, SCL7A11, SLC3A2, HMOX1, GCLM, CTH, NQO1, ACSL3, IREB2, POR, AIFM2* and overall survival in TCGA-UM patients presenting chromosome 3 disomy (chr3_status: disomy) or chromosome 3 monosomy (chr3_status: monosomy) UM patients. **D** Kaplan–Meier survival curves showing the statistically significant relationship between the expression levels of *GPX4, SCL7A11, SLC3A2, HMOX1, GCLM, CTH, NQO1, ACSL3, IREB2, POR, AIFM2* and overall survival probability in normal BAP1(BAP1_mut: NO) or in mutated BAP1 (BAP1_mut: YES) TCGA-UM samples. **E** Kaplan–Meier survival curves showing the statistically significant relationship between the expression levels of *GPX4, SCL7A11, SLC3A2, HMOX1, GCLM, CTH, NQO1, ACSL3, IREB2, POR, AIFM2* and DFS in TCGA-UM patients presenting chromosome 3 disomy (chr3_status: disomy) or chromosome 3 monosomy (chr3_status: monosomy). **F** Kaplan–Meier survival curves showing the statistically significant relationship between the expression levels of *GPX4, SCL7A11, SLC3A2, HMOX1, GCLM, CTH, NQO1, ACSL3, IREB2, POR, AIFM2* and DFS in normal BAP1(BAP1_mut: NO) or in mutated BAP1 (BAP1_mut: YES) TCGA-UM samples.

To further interrogate the significance of ferroptotic markers modulated by 1,4-dihydroxy quininib in primary UM, we analyzed their gene expression in 80 primary UMs from The Cancer Genome Atlas (TCGA). The markers selected were *SLC3A2, HMOX1, GCLM*, cystathionine gamma-lyase (*CTH*), NAD(P)H Quinone Dehydrogenase 1 (*NQO1*)*, ACSL3, IREB2*, cytochrome P450 oxidoreductases *(POR)*, and apoptosis-inducing factor mitochondria associated 2 (*AIFM2*). These markers were chosen due to their up- or down-regulation after the 1,4-dihydroxy quininib treatment, as revealed by the OMM2.5 proteome-profiling (Figs. 1, 2, Supplementary Dataset 1), and to their link with the ferroptosis process. In addition, we analysed *GPX4* and solute carrier family 7 member 11 (*SCL7A11*) transcripts, since a correlation between the expression levels of these genes and UM patients’ survival was recently reported [27]. All the selected genes were expressed in the TCGA UM samples, confirming their potential disease relevance. As chromosome 3 status is an existing predictor of UM DFS and OS [61, 62], we stratified the data based on whether patients presented with monosomy or disomy 3 and high or low expression of each selected marker gene. Monosomy 3 is observed together with alterations in BRCA1 Associated Protein 1 (BAP1) in >80% of patients [62], therefore we also stratified the data based on the presence/absence of BAP1 alterations (indicated as mutated/normal BAP1, respectively). The analysis revealed that *GPX4* (*p* = 0.00476), *SCL7A11* (*p* = 0.0169), *HMOX1* *(p* = 0.00686), *NQO1* (*p* = 3.82e-08), *POR* (*p* = 9.17e-06) and *AIFM2* (*p* = 6.81e-10) are significantly increased in primary UM samples from monosomy 3 patients compared to those with disomy 3. Conversely, disomy 3 primary UM patient samples present with significantly increased *CTH* (*p* = 1.02e-05), compared with those with monosomy 3 (Fig. 6A). Significantly higher levels of *GPX4* (*p* = 0.0275), *NQO1* (*p* = 0.00363), *POR* (*p* = 0.0358) and *AIFM2* (*p* = 0.011), are observed in primary UM patient samples presenting with mutated *BAP1*, compared to those with normal *BAP1* (Fig. 6B).

A statistically significant difference in OS probability was detected between primary UM patient samples with monosomy 3 and high *SCL7A11*, compared to those with monosomy 3 and low *SCL7A11* (*p* = 0.0083) (Fig. 6C). In particular, high levels of *SCL7A11* correlate with a decreased survival probability. In the primary UM cases with normal BAP1, we observed that high levels of *SCL7A11* (*p* = 0.00014), *HMOX1* (*p* = 0.001), *NQO1* (*p* < 0.0001), *POR* (*p* < 0.0001), *AIFM2* (*p* < 0.0001) and low levels of *CTH* (*p* = 0.011) correlate with a significant decrease in survival probability (Fig. 6D). We detected a significant difference in DFS between patients presenting chromosome 3 disomy and low *AIFM2* (*p* = 0.0031) and those with chromosome 3 disomy and high *AIFM2*. In addition, patients with monosomy 3 and high *SLC7A11* (*p* = 0.0017) or high *CTH* (*p* = 0.01) expression show a significantly decreased DFS when compared to patients with chromosome 3 monosomy and low *SLC7A11* or low *CTH* expression (Fig. 6E). In primary UM patient samples with normal BAP1, high levels of *SLC7A11* (*p* = 0.013), *ACSL3* (*p* = 0.0014), *NQO1* (*p* = 0.00013), *POR* (*p* < 0.0001) and *AIFM2* (*p* < 0.0001) correlate with a significant decrease in DFS. A significant difference in DFS was also observed between UM patients with mutated *BAP1* and low *SLC3A2* expression versus those with mutated *BAP1* and high *SLC3A2* expression (*p* = 0.042) (Fig. 6F).

**Fig. 7:**
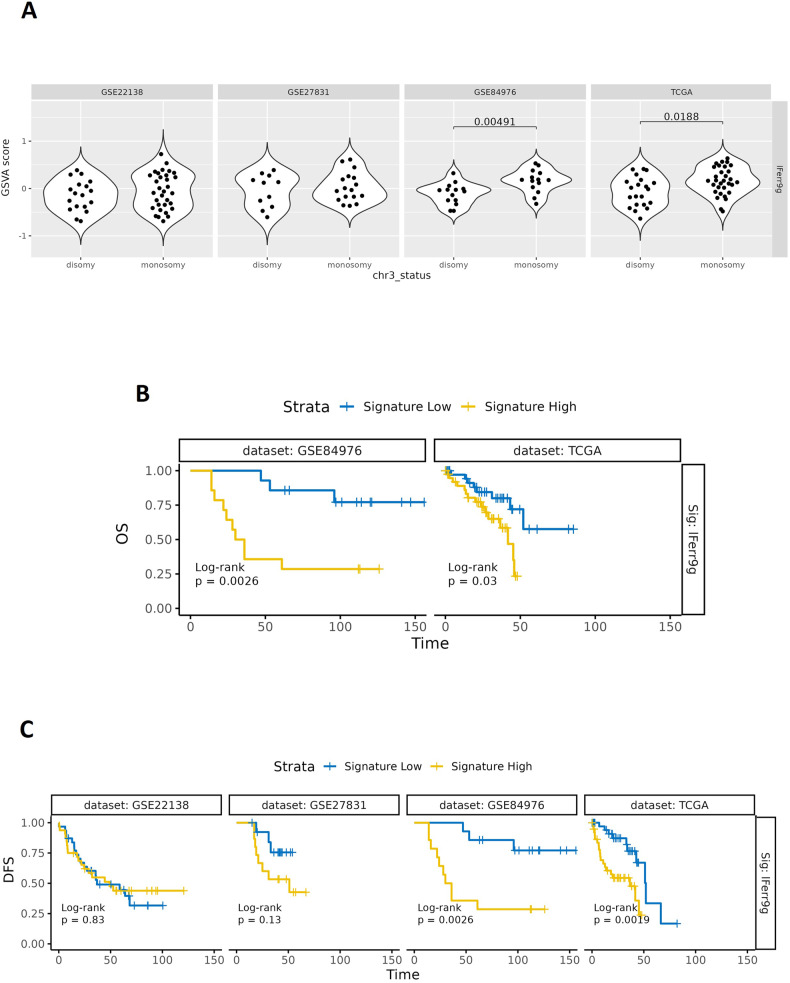
High expression of a combination of genes inhibiting ferroptosis (IFerr) correlates with reduced overall and disease-free survival in the GSE84976 and TCGA-UM cohorts. **A** Graphs showing the statistically significant relationship between expression levels of a novel ferroptosis signature (IFerr: *GPX4, SCL7A11, SLC3A2, GCLM, CTH, ACSL3, IREB2, NQO1, AIFM2*) and disomy 3 or monosomy 3 in the GSE22138, GSE27831, GSE84976 and TCGA-UM cohorts. GSVA = Gene Set Variation Analysis. **B** Kaplan-Meier curves revealing the statistically significant relationship between IFerr and OS in the GSE84976 and TCGA-UM cohorts. **C** Kaplan-Meier graphs showing the statistically significant relationship between IFerr and DFS in the GSE22138, GSE27831, GSE84976 and TCGA-UM cohorts.

We hypothesised that combinations of these ferroptotic markers may produce more significant UM prognostication value. Therefore, we created a “ferroptosis signature”, the IFerr, and investigated its relevance in the TCGA plus other UM databases. These included GSE22138, with transcriptomic data of 63 primary UM enucleations from untreated patients, (https://www.ncbi.nlm.nih.gov/geo/query/acc.cgi?acc=GSE22138); GSE27831, with gene expression profiles of 29 samples from UM patients (https://www.ncbi.nlm.nih.gov/geo/query/acc.cgi?acc=GSE27831); and GSE84976, containing data from total RNA isolated from malignant UM cells, obtained from 28 specimens surgically removed from UM patients (https://www.ncbi.nlm.nih.gov/geo/query/acc.cgi?acc=GSE84976). The IFerr signature includes genes involved in ferroptosis inhibition: *GPX4*, *SCL7A11*, *SLC3A2*, *GCLM*, *CTH*, *ACSL3*, *IREB2*, *NQO1* and *AIFM2*. Firstly, we stratified the data based on whether patients presented with monosomy or disomy 3 and high or low expression of the signature. The results showed a significantly higher IFerr signature in monosomy 3 patients in both the GSE84976 (*p* = 0.00491) and TCGA (*p* = 0.0188) databases, suggesting that in those UM cohorts a high expression of genes inhibiting ferroptosis correlates with the chromosome alteration highly associated with disease relapse (Fig. 7A). We then investigated the correlation between the expression level of IFerr and OS in GSE84976 and TCGA databases and found a high IFerr signature to correlate with a significant decrease in OS (*p* = 0.0026, *p* = 0.03, respectively), once again indicating that increased levels of genes blocking ferroptosis correlate with a worse clinical outcome (Fig. 7B). Finally, we analysed the correlation between IFerr and DFS in the 4 databases. Intriguingly, high IFerr also correlates with a significant decrease in DFS in GSE84976 (*p* = 0.0026) and TCGA (*p* = 0.0019) databases (Fig. 7C). Collectively, these results suggest that monosomy 3 patients may present mechanisms involved in preventing ferroptosis and that high levels of genes involved in ferroptosis inhibition are associated with worse clinical outcome in UM patients.

## Discussion

In UM, metastases are associated with poor prognosis, ineffective treatments and death within 12 months [3]. There is an unmet need for better biomarkers for MUM prognosis plus more effective and affordable treatments. Here, we identified ferroptosis as a mechanism of the anti-MUM effects mediated by 1,4-dihydroxy quininib and central to IFerr, a novel biomarker signature for the prognostication of MUM.

Previously, small molecule drugs preferentially targeting CysLT_1_ over CysLT_2_ exerted anti-UM phenotypes [17]. Quininib, a CysLT_1_ antagonist, is an anti-inflammatory and anti-angiogenic [58, 63] molecule with anti-cancer activity in human ex vivo colorectal cancer patient tumor explants and colorectal cancer xenograft models [64]. Quininib and its analogue 1,4-dihydroxy quininib significantly inhibit survival, long-term proliferation and oxidative phosphorylation in primary and MUM cell lines [17]. Furthermore, 1,4-dihydroxy quininib alters the secretion of inflammatory factors in primary UM samples and decreases the expression of an oxidative phosphorylation marker in a cell line-derived mouse orthotopic xenograft model of MUM [18].

However, the fundamental molecular mechanism of action of quininib drugs in these suite of models was poorly understood. Ferroptosis is a new, druggable target for MUM [27, 28]. Modulation of ferroptosis can inhibit cancer cell growth, improve the sensitivity of chemotherapy and radiotherapy and expand treatment options for different cancer types [23]. Thus the evidence reported here linking, for the first time, 1,4-dihydroxy quininib with ferroptosis modulation in UM is original and significant, since it provides a potential opportunity to fundamentally change the clinical management of this rare and aggressive cancer type.

OMM2.5 cells treated with 1,4-dihydroxy quininib for 8 and 24 h showed increased levels of key ferroptotic and anti-oxidant modulators *e.g*. HO-1, GCLM, oxidative stress-induced growth inhibitor 1 (OSGIN1) and thioredoxin (TXN) (Figs. 1, 2, Supplementary Dataset 1). These are regulated by NRF2 and oxidative stress [65]. In agreement, 1,4-dihydroxy quininib significantly increased the levels of ROS and phosphorylated NRF2 after the 8 h-treatment (Fig. 3B, C). HO-1, one of the main targets of NRF2, is the first rate-limiting enzyme in the conversion of heme into ferrous ions, carbon monoxide, and biliverdin [66]. HO-1 can act either in a cytoprotective or cytotoxic manner depending on specific cellular conditions [67]. In particular, HO-1 overexpression is associated with malignant cancer cell growth, proliferation and invasion [46, 47, 68, 69], but its induction can also reduce tumor cell proliferation [39, 47, 70, 71]. Indeed, increased HO-1 activity can promote the accumulation of the intracellular labile iron pool (LIP) essential for lipid peroxidation and ferroptosis [53]. LIP is a cofactor for enzymes producing ROS, such as nicotinamide adenine dinucleotide phosphate (NADPH) oxidases (NOXs), POR, nitric oxide synthases and lipoxygenases [21]. Notably, we observed significantly increased POR expression after the 8-h 1,4-dihydroxy quininib treatment (Supplementary Dataset 1). POR, by transferring electrons from NAD(P)H to oxygen, generates hydrogen peroxide, which reacts with iron generating reactive hydroxyl radicals for peroxidation of PUFA chains in membrane phospholipids, thereby disrupting membrane integrity during ferroptosis [72].

1,4-dihydroxy quininib increased HO-1 enzymatic activity and downstream ferroptosis in OMM2.5 cells based on biliverdin content and LOOH levels (Fig. 3D, H). Intriguingly, intracellular biliverdin significantly increased after the 4-h treatment and higher LOOH levels were observed after the 4- and 8-h treatment. In parallel, at 8 hpt IREB2 levels decreased (Fig. 2B, D, E). IREB2 is degraded when intracellular iron is excessive, thus, indirectly confirming accumulation of cytosolic Fe^2+^ in OMM2.5 cells [73]. Collectively, these data indicate that the 4- and 8-h 1,4-dihydroxy quininib treatment induces ferroptosis in OMM2.5 cells through the NRF2/HO-1 axis.

GPX4 is a fundamental anti-oxidant system, which blocks lipid peroxidation [74]. 1,4-dihydroxy quininib significantly increased GPX4 levels after 4 h, but not after 8 h (Fig. 3E, F). This may reflect the cells trying to escape ferroptosis by increasing lipid repair capacity via activation of the xc- system. This is further corroborated by increased GSH content and GCLM expression (Figs. 1E; 3G, I). Indeed, GSH is a cofactor and synthetic substrate for GPX4 and derives from cystine, which is imported intracellularly through the xc- system [29].

At 24 h, phosphorylated NRF2, biliverdin content and LOOH did not change after 1,4-dihydroxy quininib exposure (Fig. 3B, C, D, H). However, we observed a significant decrease in GPX4 expression, therefore, the core defence mechanism against ferroptosis is affected by the longest treatment duration (Fig. 3E, F). Intriguingly, we detected a significantly increase in GSH content and GCLM expression (Fig. 3G), suggesting again that OMM2.5 cells are adopting an enhanced antioxidant capacity. CysLT_1_ antagonists quininib and montelukast and CysLT_2_ antagonist HAMI 3379 did reduce GPX4 but did not affect GCLM expression at 24 h (Fig. 4C, D), suggesting that the 1,4-dihydroxy quininib mechanism of action in OMM2.5 cells differs from those of the other CysLT_1/2_ antagonists. Human SH-SY5Y neuroblastoma cells activate an adaptive response of the GSH system to prolonged iron loads through increased expression of the GCLC and GCLM subunits of GCL [56]. Therefore, our results suggest that 24-h 1,4-dihydroxy quininib treatment may promote ferroptosis by specifically inhibiting GPX4, similarly to observations with RSL3, which inhibits GPX4 directly [31].

**Fig. 8:**
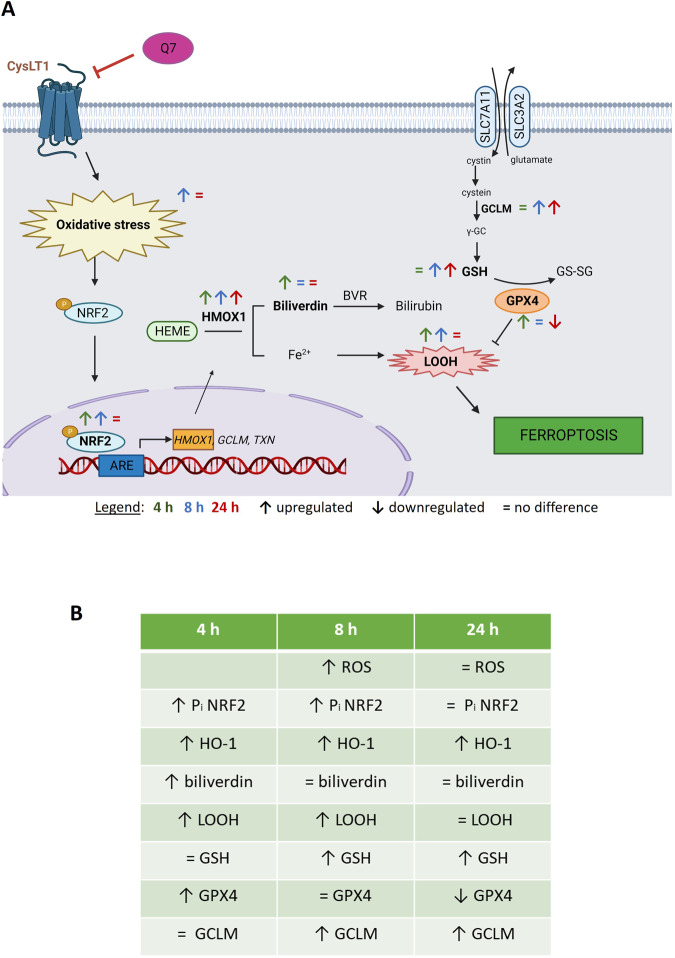
Schematic model summarising alterations in the expression of the indicated ferroptosis hallmarks in MUM samples after 1,4-dihydroxy quininib treatment. **A** Figure showing the specific factors modulated by 1,4-dihydroxy quininib in a time-dependent fashion. Created with BioRender.com. Subscription: Institution (University College Dublin) **B**. Table summarising the significant differences in ferroptosis hallmarks after 1,4-dihydroxy quininib. HO-1 and GCLM upregulations are based on OMM2.5 cells proteome profiling results. Non-significant differences are represented with “=”, significant downregulation with “**↓**” and significant upregulation with “**↑**”. Q7 = 1,4-dihydroxy quininib; Pi = phosphorylated; HMOX = HO-1; h = hours.

Altogether, our evidence suggests a dynamic effect of 1,4-dihydroxy quininib on ferroptosis in OMM2.5 cells, potentially due to transient activity or upregulation of compensatory resistance pathways (Fig. 8). Importantly, we demonstrated that 1,4-dihydroxy quininib increases HO-1 and 4-HNE levels in tumors explants from MUM OPDX mouse models. These are models in which human MUM is orthotopically implanted in the liver, thus they mantain the genetic heterogeneity of the original MUM patients’ tumors [75]. 4-HNE is a toxic product deriving from the oxidative cleavage of lipid peroxides, therefore it increases during ferroptosis [36]. The evidence obtained with MUM tumor explants confirms the significant role of 1,4-dihydroxy quininib in modulating ferroptosis in more clinically relevant models of MUM. Indeed, tumor biopsies better recapitulate the physiology of the tumor microenvironment, allowing cell-to-cell contact and cell-to-matrix synthesis as well as the development of the oxygen, nutrient, and hormone levels typically found in patient’s tumors [76]. At the same time, the upregulation of HO-1 and lipid peroxidation, observed both in OMM2.5 cells and in MUM tumor biopsies, validate OMM2.5 cells as a good model where to investigate MUM biology and carry out drug screening.

The quininib series of drugs preferentially antagonise CysLT_1_ receptors [58, 63]. In cell-based assays, the salt form of 1,4 dihydroxy quininib exerted its greatest antagonistic potency at CysLT_1,_ with moderate antagonism of CysLT_2_, and inhibited VEGFR2 and VEGF3 in reconstituted kinase assays [63]. Considering CysLT_1_ as the prototypical target, a possible explanation for 1,4 dihydroxy quininib inducing ferroptosis is via modulation of a STAT3/GSK-3β/β-catenin/GPX4 pathway. In mouse embryonic stem cells, CysLT_1_ signaling promoted STAT3 and GSK-3β phosphorylation but inhibited β-catenin phosphorylation [77]. CysLT signalling ultimately led to the activation of β-catenin in intestinal epithelial cells [78] and in colon cancer cells [79]. Intriguingly, beta-catenin conferred ferroptosis resistance in gastric cancer by binding to the promoter region of GPX4 and inducing its expression [80]. This highlighted how aberrant Wnt/β-catenin activation led to ferroptosis resistance, suggesting a therapeutic option to enhance chemo-sensitivity for advanced gastric cancer patients. In gastric cancer, STAT3 directly bound to the promoters of negative ferroptosis regulators (GPX4, SLC7A11, and FTH1) and modulated their expression [81]. Genetic ablation of STAT3 activity triggered ferroptosis through lipid peroxidation and Fe^2+^ accumulation in gastric cancer cells [81]. Thus, inhibition of the STAT3-ferroptosis inhibitory axis reduced tumor growth and alleviated chemoresistance [81]. Further proof of ferroptosis induction by impairing STAT3/NRF2/GPX4 signaling is observed in osteosarcoma cells, where STAT3 inhibitors reactivated ferroptosis and increased cisplatin sensitivity [82].

Overall, CysLT_1_ antagonism in UM may lead to a downregulation in STAT3 and GSK-3β phosphorylation, reducing β-catenin activation and decreasing GPX4 expression (*e.g*. after a 24 h of 1,4-dihydroxy quininib treatment) or promoting Fe^2+^ accumulation and lipid peroxidation (*e.g*. after the 4- and 8- h 1,4-dihydroxy quininib treatment). The timely activation of the different components in the STAT3/GSK-3β/β-Catenin/GPX4 pathway may explain the observed dynamic effect of 1,4-dihydroxy quininib, and further evidence will be fundamental to understand the specific antioxidant programs used by MUM cells. This will be key to uncover potential resistance mechanisms and thus extend drug efficacy. Importantly, ferroptosis induction-based treatment can also be combined with immune checkpoint blockade options. Upregulation of CysLT_1_, which enhances Wnt/β-catenin signaling, led to endogenous and IFNγ-induced PD-L1 expression in colorectal cancer cells [79]. Inhibiting CysLT_1_ reduced both Wnt/β-catenin and PD-L1 expression, thus elucidating the importance of targeting CysLT_1_ to reach beneficial outcomes of immune checkpoint blockade in colorectal cancer patients [79]. Immunotherapy in combination with ferroptosis induction represents a promising therapeutic option, since the two treatment modalities mutually potentiate each other, triggering synergistic anticancer effects [83]. Therefore, further exploration of 1,4-dihydroxy quininib effects in UM samples must be performed, in order to facilitate the identification of new therapies that will benefit UM patients.

1,4-dihydroxy quininib modulates the expression of proteins which can be used as potential therapeutic targets. Indeed, unbalancing ferroptosis defences in cancer cells can reveal biomarkers to select patients for effective therapeutic options and for the optimal application of ferroptosis-inducing drugs. Several studies report prognostic gene signatures associated with ferroptosis in cancer [84, 85]. A link between ferroptosis-related, long noncoding RNAs (FRLs) and UM was identified in the TCGA and FerrDb databases. In particular, a five-FRLs (*AC104129.1, AC136475.3, LINC00963, PPP1R14B.AS1, and ZNF667.AS1*) signature impacted survival prediction and selection of immunotherapies for UM patients [86]. Luo et al. reported a ferroptosis-related seven-gene signature (*ALOX12, CD44, MAP1LC3C, STEAP3, HMOX1, ITGA6, and AIFM2/FSP1*) using TCGA-UVM as the training cohort and GSE22138 from Gene Expression Omnibus (GEO) as the validation cohort [87]. The signature could predict UM outcome related to mast cell resting [87]. Another study exploited weighted gene co-expression network analysis (WGCNA) to identify a prognostic signature including 5 genes (*EEFSEC, EEF1A2, ALDH1A3, CTNNB1 and COMMD2*) [88]. More recently, *GPX4* and *SLC7A11* levels negatively correlated with UM survival, and ferroptosis susceptibility correlated with loss of BAP1 [27]. Clearly, biomarkers have the potential to improve clinical management of UM. Here, we reported several ferroptosis-associated genes which correlate with UM patient survival, by analysing the TCGA database and stratifying the data based on chromosome 3 and *BAP1* status. Furthermore, we identified IFerr, a novel ferroptosis signature including negative regulators of ferroptosis (*GPX4, SCL7A11, SLC3A2, GCLM, CTH, ACSL3, IREB2, NQO1 and AIFM2*), which can predict OS and DFS. In particular, an increased IFerr signature, thus increased levels of genes involved in ferroptosis inhibition, correlates with a worse clinical outcome. Importantly, we validated the reliability of this signature in independent cohorts. Thus, the IFerr signature displays potential as a biomarker for UM prognostication and treatment.

Overall, this study elucidated for the first time the role of 1,4-dihydroxy quininib in modulating ferroptosis hallmarks in MUM samples and revealed a novel ferroptosis gene signature which predicts UM patient outcomes. This evidence justifies additional translational studies in clinically relevant in vivo and ex vivo UM models, aimed to improve the clinical management and treatment of this challenging cancer type.

## Material and Methods

### Cell culture

UM cell line OMM2.5 derived from a metastatic UM tumor and Mel285 derived from primary UM tumor were kindly provided by Dr. Martine Jager (Leiden, The Netherlands) [89]. Cell cultures were maintained at 37°C/5% CO_2_ in RPMI 1640 Medium (Gibco, Gaithersburg, MD, USA) supplemented with 10% FBS and 2% Penicillin/Streptomycin. OMM2.5 and Mel285 cells were authenticated by short-tandem repeat (STR) profiling performed by American Type Culture Collection (ATCC) and tested for absence of mycoplasma contamination.

### Ethics

Tumors samples were obtained from Bellvitge Hospital (HUB) and the Catalan Institute of Oncology (ICO) with approval by the Ethical Committee (CEIC Bellvitge Hospital) and ethical and legal protection guidelines of human subjects, including informed patient consent, were followed. The animal experimental design was approved by the IDIBELL animal facility committee (AAALAC Unit1155) under approved procedure 9111. All animal models were generated in accordance with the guideline for Ethical Conduct in the Care and Use of Animals as stated in The International Guiding Principles for Biomedical Research Involving Animals, developed by the Council for International Organizations of Medical Sciences.

### MUM OPDX mouse models

Human tumors were aseptically isolated and placed at room temperature in Dulbecco’s modified Eagle’s medium (DMEM) supplemented with 10% FBS plus 50 U/ml penicillin and 50 mg/ml streptomycin. Tumors were orthotopically implanted into the liver of two six-week-old male athymic nude mice (strain Hsd:Athymic Nude-Foxn1nu) purchased from Envigo. Briefly, mice were anesthetized with a continuous flow of 1% to 3% isoflurane and oxygen mixture (2 L/min). After performing a median laparotomy, the tumor fragment was anchored with a Prolene 7-0 suture into a small pocket created in the anterior hepatic lobe, and the abdominal incision will be closed with surgical staples. After implantation, mice were inspected twice a week, and at euthanasia, OPDX were harvested, cut into small fragments and serially transplanted into new animals for tumor perpetuation and/or experimental procedures. Animals were housed in a sterile environment, cages and water were autoclaved and bedding and food was γ-ray sterilized.

### Explant culture of MUM liver tumors from OPDX mouse model

Immediately following dissection, MUM tissue was placed into complete culture medium at room temperature. Upon arrival at UCD, the tissue was washed three times in sterile PBS wash buffer (PBS and 2% Penicillin/Streptomycin). Using a sterile scalpel and forceps, the tumor was cut into 3 pieces, arbitrarly renamed as left (L), middle (M) or right (R). (Fig. 5A). 2 fragments of each piece were incubated in 20 μM 1,4-dihydroxy quininib or 0.5% DMSO, made up to 1 ml in complete culture medium in a 12-well plate. Explants were incubated for 72 h at 37°C/5% CO2. Plates were wrapped in parafilm to prevent evaporation of medium during the incubation period. After 72 h, the explant tissue was snap-frozen in liquid nitrogen and stored at –80°C.

### Drug preparation for use in cell and explant culture

Quininib, 1,4-dihydroxy quininib, montelukast (Sigma-Aldrich, St. Louis, MO, USA #SML0101), HAMI 3379 (Cayman Chemical, Ann Arbor, MI, USA #10580) and erastin (Sigma-Aldrich; St. Louis, MO, USA #E7781) were dissolved in 100% DMSO and stored as (10–50 mM) stock solutions. Working solutions (100 μM) were prepared fresh prior to each experiment in complete cell culture medium as described above. Drugs were made to final test concentrations by adding the required volume of the working solution to cells in complete media. 0.5% DMSO was used as a control for all other drug treatment experiments.

### Proteomics sample preparation

OMM2.5 cells were seeded at a density of 1 × 10^6^ cells in triplicate wells and drug treated for 4, 8 or 24 h with 0.5% DMSO or 20 µM 1,4-dihydroxy quininib. Four independent experiments were performed. Proteins were isolated using PreOmics iST 8X for protein/proteomics preparation kit (PreOmics GmbH; Martinsried, Germany) according to manufacturer’s protocol.

The samples were analyzed by the UCD Conway Institute Mass Spectrometry Resource (MSR) on a Thermo Fisher Scientific Inc. Q Exactive mass spectrometer connected to a Dionex Ultimate 3000 (RSLCnano) chromatography system. Peptides were separated on C18 home-made column (C18-AQ Dr. Maisch Reprosil-Pur 100 × 0.075 mm × 3 μm) over 120 min at a flow rate of 250 nL/min with a linear gradient of increasing ACN from 1% to 27%. The mass spectrometer was operated in data-dependent mode; a high resolution (70,000) MS scan (300–1600 m/z) was performed to select the twelve most intense ions and fragmented using high energy C-trap dissociation for MS/MS analysis.

Raw data from the Q Exactive was processed using MaxQuant (version 2.0.3.0) incorporating the Andromeda search engine, as described in [90]. Briefly, to identify peptides and proteins, MS/MS spectra were matched against Uniprot homo sapiens database (2021_03) containing 78,120 entries. All searches were performed using the default setting of MaxQuant, with trypsin as specified enzyme allowing two missed cleavages and a false discovery rate of 1% on the peptide and protein level. The database searches were performed with carbamidomethyl (C) as fixed modification and acetylation (protein N terminus) and oxidation (M) as variable modifications. For the generation of label-free quantitative (LFQ) ion intensities for protein profiles, signals of corresponding peptides in different nano-HPLC MS/MS runs were matched by MaxQuant in a maximum time window of 1 min. Perseus software was used to process the data and create heatmaps. After screening a large number of proteins simultaneously an important statistical concept is multiple hypothesis testing, in which many statistical tests are conducted at the same time and detection of a protein with a relatively small *p*-value may be a false discovery. To address this problem, Storey and Tibshirani propose a new metric, the *q*-value [91]. The *q*-value is intended to be analogous to the *p*-value but takes into account multiple testing corrections. The results with the multiple testing corrections are shown in Supplementary Dataset 2. The *p*-value produces false discoveries but the *q*-values fail to detect some true discoveries, as revealed by immunoblots (Fig. 2D–G). In light of this, in the present work, we discuss the results without the multiple testing corrections (i.e. the *p*-values) but include the results with the multiple testing corrections (i.e. the *q*-values) in the Supplementary material (Supplementary Dataset 2). ClueGo (v2.5.8) and Cluepedia (v1.5.8) plugins in Cytoscape (v3.8.2) with the Homo sapiens (9606) marker set was utilized for GO:Biological processes pathway analysis of enriched proteins. Functional Enrichment analysis tool (FunRich v3.1.3) was used to create Venn diagrams using associated gene names identified. The mass spectrometry proteomics data is deposited to the ProteomeXchange Consortium via the PRIDE [92] partner repository with the dataset identifier PXD046822 and 10.6019/PXD046822.

### Explant total protein determination

Total protein was extracted from each piece of tumor explant tissue. Each individual explant was placed in a tube with 400 μl of ice-cold T-PER lysis reagent (Thermo Fisher Scientific, Rockford, IL, USA) supplemented with 10 μl/ml protease inhibitor and a 3 mm stainless steel bead. Tubes were placed in a TissueLyser II (Qiagen) for 2.5 min to homogenize the tissue. The tissue lysate was centrifuged at 14,000 rpm for 30 min at 4°C. The supernatant was used immediately for protein determination or stored at −80°C. The BCA (Thermo Fisher Scientific, Rockford, IL, USA) kit was used to quantify the total protein extracted from explant tissue in μg/ml as per the manufacturer’s instructions.

### Western blot

OMM2.5 cells were seeded at 1 × 10^6^ cells per well of a 6-well plate and left to adhere for 24 h. Cells were treated with 0.5% DMSO or 20 μM 1,4-dihydroxy quininib for 4, 8, or 24 h. Mel285 cells were seeded at 1 × 10^6^ cells per well of a 6-well plate, left to adhere for 24 h and then treated with 0.5% DMSO or 20 μM 1,4-dihydroxy quininib for 24 h. Total protein was extracted from cells as described [18]. Cells protein concentrations were measured using BCA protein assay kit (ThermoFisher Scientific; Waltham, MA, United States) in accordance with manufacturer’s instructions, and 10 μg of protein was loaded per lane (*N* = 3, at least). Explants protein concentrations were determined as described above and 8 μg of protein was loaded per lane. 3 tumors (=3 animals) for each OPDX model were used for these experiments, i.e. 3 tumors for UVM4 and 3 tumors for UVM7. For each tumor, fragments “L” and “R” of were used for western blot experiments. Blots were probed for HMOX1 (10701-1-AP, Proteintech, IREB2 (23829-1-AP, Proteintech), GDF15 (27455-1-AP, Proteintech), NRF2 (16396-1-AP, Proteintech), GPX4 (67763-1-Ig Proteintech), GCLM (14241-1-AP, Proteintech), 4-HNE (JaICA, MHN-100P) and β-actin (A5441, Sigma-Aldrich). Anti-rabbit IgG, HRP-linked Antibody (1:3000; #7074 s, Cell Signaling Technology) and anti-mouse IgG, HRP-linked Antibody (1:3000; #7076 s, Cell Signaling Technology) were used as secondary antibodies. Signal was detected with enhanced chemiluminescence substrate (Pierce™ ECL Western Blotting Substrate; ThermoFisher Scientific). For 4-HNE, the 48 kDa band was considered for the quantification, based on previous evidence showing that a 48 kDa protein exbits a strong reactivity with the antibody in hepatocytes treated with 10 µM HNE [93].

### Measurement of ROS levels

Levels of reactive oxygen species (ROS) were determined using the fluorescent probe 2,7-dichlorofluorescein diacetate (DCFH-DA, Sigma-Aldrich, St. Louis, MO, USA #D6883). Cells were seeded at a density of 2 ×10^4^ into 96-well plates and allowed to adhere overnight. They were then treated for 8 or 24 h with 0.5% DMSO or 20 µM 1,4-dihydroxy quininib in RPMI 1640 Medium (Gibco, Gaithersburg, MD, USA) supplemented with 10% FBS and 2% Penicillin/Streptomycin. Media was removed and cell were washed with a 0.1% Triton solution in complete media to enhance cellular probe permeation. 100 µL of a DCFH-DA working solution (200 µM) prepared in RPMI 1640 Medium (Gibco, Gaithersburg, MD, USA) supplemented with 2% Penicillin/Streptomycin but no FBS was added to each well and incubated at 37 °C/5% CO_2_ for 30 min in the dark. After incubation, the solution was removed and wells were washed with in 1X PBS. 200 µl of radioimmunoprecipitation assay (RIPA, Sigma-Aldrich, St. Louis, MO, USA, #R0278) buffer were added to each well. The plate was incubated on ice for 5 min, then cell lysate was collected into 1.5 mL tubes. Tubes underwent centrifugation at 21,130× *g* for 10 min at 4 °C. 100 μL of supernatant were transferred to a 96 well plate and the fluorescence intensity was measured using a fluorescence a microplate reader at an excitation wavelength of 485 nm and an emission wavelength of 530 nm. The same protocol was used on wells without cells, which represented the negative control. 10 μL of the supernatant were used to perform the Bicinchoninic Acid (BCA) Protein Assay (#23227, ThermoFisher Scientific name of kit). Six replicate wells were used for each group. The mean fluorescence intensity value of wells without cells was substracted to each fluorescence intensity value obtained for the other conditions. The final results are expressed as fluorescence intensity (AU)/proteins (mg/mL). Experiments were conducted in triplicate (24 h-treatment) or quadruplicate (8 h-treatment). The results are expressed as percentage of the control.

### Biliverdin measurement

OMM2.5 cells were seeded at 1 × 10^6^ cells per well of a 6-well plate and left to adhere for 24 h. Cells were treated for 4, 8, or 24 h with 0.5% DMSO or 20 μM 1,4-dihydroxy quininib. At the end of the treatment cells were detached with trypsin, resuspended in a medium, centrifuged at 800 g for 5 min, washed once in PBS, and centrifuged again. The pellet was frozen at -80°C. Each cell pellet was resuspended in 0.65 mL of lysis buffer consisting of 1% Triton-X100, 0.4 g/L bovine serum albumin in PBS, pH 8.5. The lysed pellets were divided into 3 aliquots for the determination of autofluorescence (blank), bilirubin, and biliverdin. For fluorescence calibration, bilirubin standard solutions (2, 5, 10, 20 nM) were prepared in lysis buffer. Aliquots from the autofluorescence wells were used for protein determination. Quantification of intracellular bilirubin was performed using a high-throughput fluorometric method based on the high-affinity selective binding of bilirubin to the recombinant protein HELP -UnaG (HUG) [94]. The assay was adapted for the measurement of biliverdin by adding biliverdin reductase (BVR) to the assay buffer as described by Tramer and colleagues [94–96]. Experiments were conducted in triplicate.

### Determination of thiol groups

The concentration of nonprotein thiol groups (RSH), reflecting about 90% of the GSH cellular content, was measured in total cell lysates. OMM2.5 cells were seeded at a density of 1 ×10^6^ cells per well of a 6-well plate and left adhere for 24 h. Cells were then treated for 4, 8 or 24 h with 0.5% DMSO or 20 µM 1,4-dihydroxy quininib in RPMI 1640 Medium (Gibco, Gaithersburg, MD, USA) supplemented with 1% FBS and 2% Penicillin/Streptomycin. Media was removed and cells were washed in 1X PBS. 1 ml of trypsin was added to each well to promote cells detachment. 1 ml of fresh media was then added to each well to neutralize the trypsin activity. Cells underwent centrifugation at 1200 rpm for 4 min. The surnatant was removed, the cell pellet was washed in 1 ml of PBS media and centrifugated at 1200 rpm for 4 min. The last step was performed twice. Cell pellet was snap frozen in liquid nitrogen and stored at -80° C. RSH levels were evaluated by a spectrophotometric assay based on the reaction of thiol groups with 2,2-dithio-bis-nitrobenzoic acid (DTNB). A DTNB solution and the samples were mixed and incubated at room temperature for 20 min in the dark until the noticeable appearance of a yellow color. After incubation, samples were centrifuged at 3000 rpm for 10 min. The supernatant was collected and set in a black 96-well plate for measurement of the absorbance in a microplate reader (Biotek Synergy-HT, Winooski, VT, USA) at λ = 412 nm. Experiments were conducted in quadruplicate, with 3 technical replicates per condition. The results are expressed as percentage of the control.

### Measurement of lipid peroxidation

Levels of LOOH were evaluated through the oxidation of Fe^2+^ to Fe^3+^ in the presence of xylenol orange. OMM2.5 cells were seeded at a density of 1 ×10^6^ cells per well of a 6-well plate and treated for 4, 8 or 24 h with 0.5% DMSO or 20 µM 1,4-dihydroxy quininib, as above (see the method Determination of thiol Groups). The assay mixture contained 200 μg of the sample (total cell lysate), 100 μM xylenol orange, 250 μM ammonium ferrous sulphate, 90% ethanol, 4 mM butylated hydroxytoluene and 25 mM H_2_SO_4_. Samples were incubated at room temperature for 30 min, and the absorbance was finally measured at λ = 560 nm using a microplate reader (Biotek Synergy-HT, Winooski, VT, USA). Experiments were conducted in quadruplicate. The results are expressed as a percentage of the control.

### MTT assay

Cell metabolism, an indirect measure of viability, was determined using MTT (3-(4,5-dimethylthiazol-2-yl)-2,5-diphenyltetrazolium bromide (#M5655), Sigma-Aldrich; St. Louis, MO, USA) assay [97]. Briefly, 5 × 10^3^ cells/well were seeded into 96-well plates and allowed to adhere for 24 h. Cells were treated in triplicates with either 0.5% DMSO (vehicle control), 15% H_2_O_2_ (positive control) or 1, 5, 10, 20, 50 µM erastin (#E7781, Sigma-Aldrich; St. Louis, MO, USA), prepared in complete media for 96 h. Once drug solution was removed, MTT dye and serum-free media were added in a 1:10 ratio, to each well and incubated in the dark for 2 h at 37 °C. Then, 100% DMSO (1:1 ratio) was added to each well and absorbance was measured at 570 nm using a SpectraMax® M2 microplate reader (Molecular Devices Corporation, Sunnyvale, CA, USA). Experiments were conducted in triplicate. The results are expressed as percentage of the control.

### TCGA data analysis

Clinical and mutational data of primary uveal tumors was collected from TCGA-UM dataset included in The Cancer Genome Atlas (*n* = 80). Annotated mutational data was downloaded from the cBioPortal [98]. RNA-seq was downloaded in fragments per kilobase per million (FPKM), then converted to log2 scale. Bulk RNA-seq data together with clinical annotation from the studies by Laurent et al., Gangemi et al., van Essen et al. [99–101] were obtained from the GEO database (dataset identifiers: GSE22138, GSE27831 and GSE84976, respectively). Expression between chromosome 3 monosomic or disomic patients was statistically compared using the Mann Whitney U test. Survival analysis was performed using Overall Survival (OS) or Disease Free Survival (DFS) from annotation. Kaplan–Meier curves were plotted to represent the result and log-rank test was computed.

### Statistics

Statistical analyses were performed using GraphPad Prism v7.00 (GraphPad Software, San Diego, CA, USA). The normal distribution was assessed using the Shapiro-Wilk test. For non-normally distributed sample data, comparisons between two groups were analyzed using Mann-Whitney U test and comparisons between three or more groups were analysed using Kruskal-Wallis test. In the case of normally distributed data, two-group comparisons employed a two-tailed t-test, while comparisons involving three or more groups utilized a one-way ANOVA. The Brown-Forsythe test accompanied the one-way ANOVA to evaluate the homogeneity of variance among different groups. The obtained p-values for all tests indicated homogeneity of variance across groups. Sample sizes were chosen based on previously published experiments [17, 18, 39, 97]. Error bars depict the standard error of the mean (SEM), indicating the variability among samples. Central values are presented as the mean. The investigators were not blinded to allocation during experiments and outcome assessment. Significance was set at *p* value less than 0.05.

### Supplementary information

Supplementary Dataset 1

Supplementary Figure 1

Supplementary Figure 2

Supplementary Dataset 2

Supplementary Figure Legends

Original Western Blot Figure 2

Original Western Blot Figure 3

Original Western Blot Figure 4

Original Western Blot Figure 5

## Data Availability

The datasets generated during and analysed during the current study are not publicly available but will be available from the corresponding author once the paper will be accepted for publication.
