# Supplementary material for: 1,4-dihydroxy quininib activates ferroptosis pathways in metastatic uveal melanoma and reveals a novel prognostic biomarker signature
Source: Cell Death Discov. 2024 Feb 10;10:70. doi: 10.1038/s41420-023-01773-8 (PMC10858877; doi:10.1038/s41420-023-01773-8)
Supplement: Supplementary file 2 — Supplementary Figure 1 [file 41420_2023_1773_MOESM2_ESM.pdf]

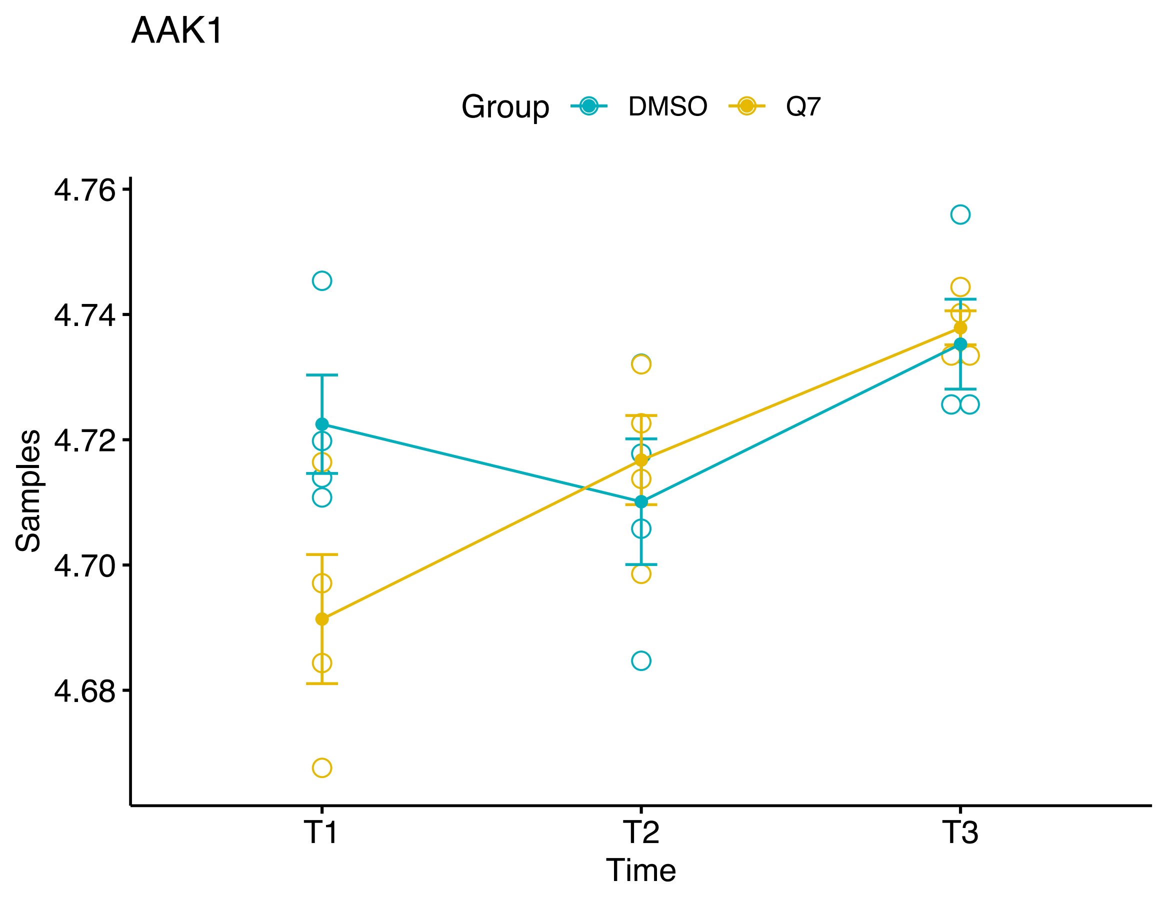
AAK1
Group
DMSO
Q7
4.76
4.74
4.72
4.70
4.68
Samples
T1
T2
T3
Time

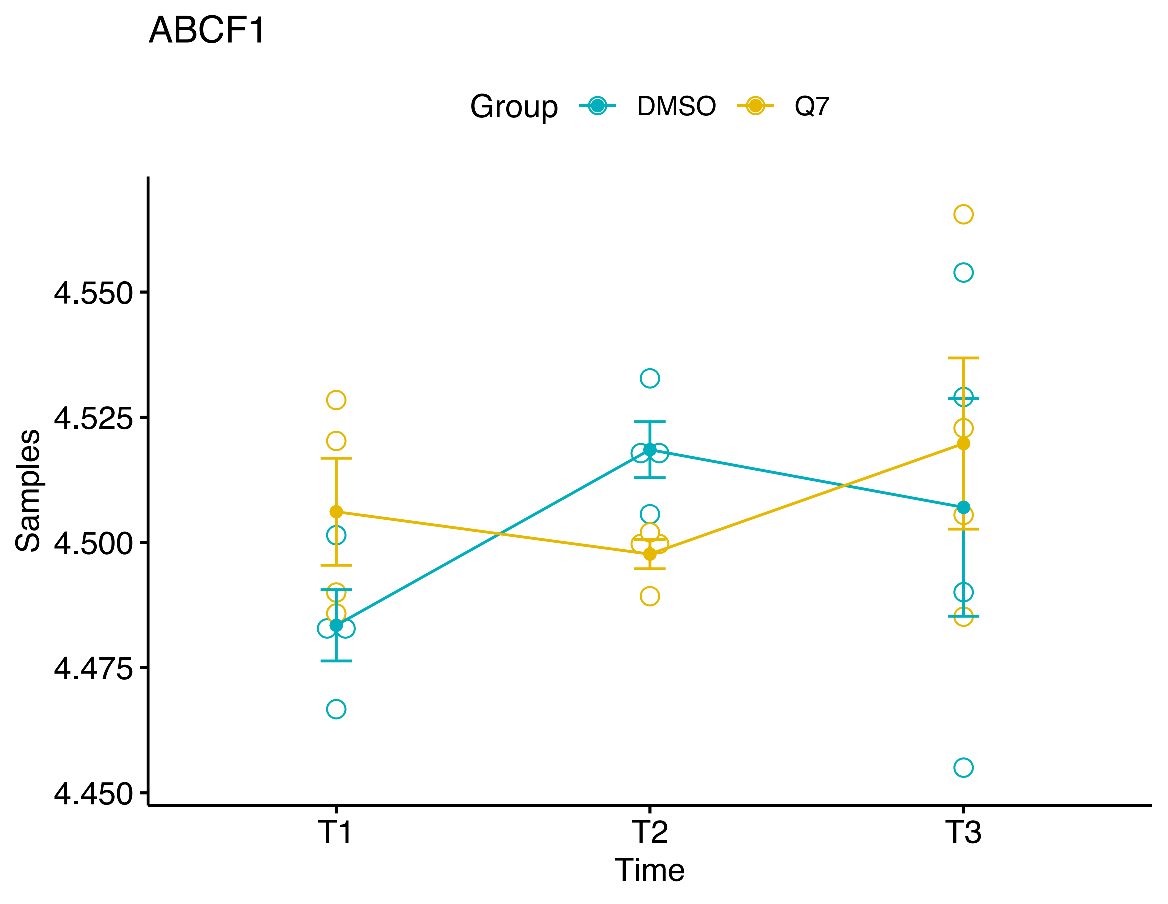

ABCF1
Group
DMSO
Q7
Samples
4.550
4.525
4.500
4.475
4.450
T1
T2
T3
Time

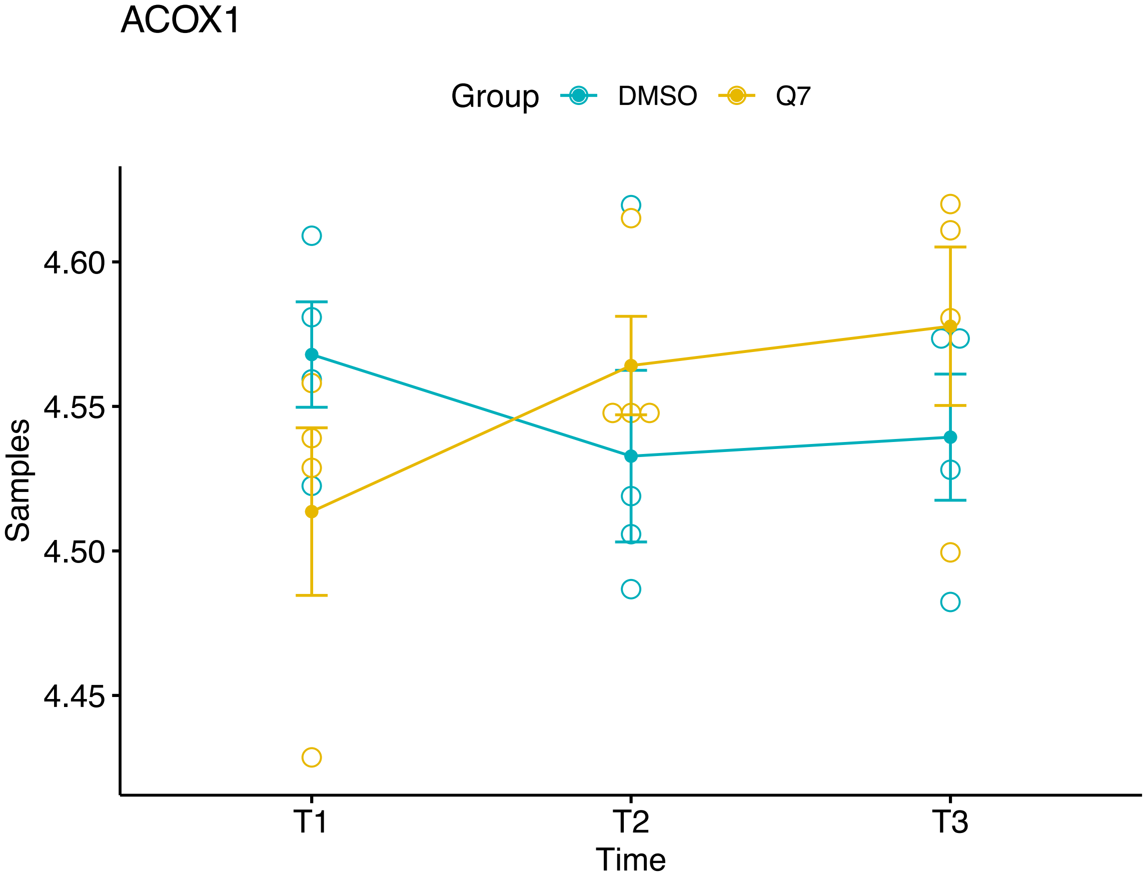
ACOX1
Group
DMSO
Q7
4.60
4.55
4.50
4.45
Samples
T1
T2
T3
Time

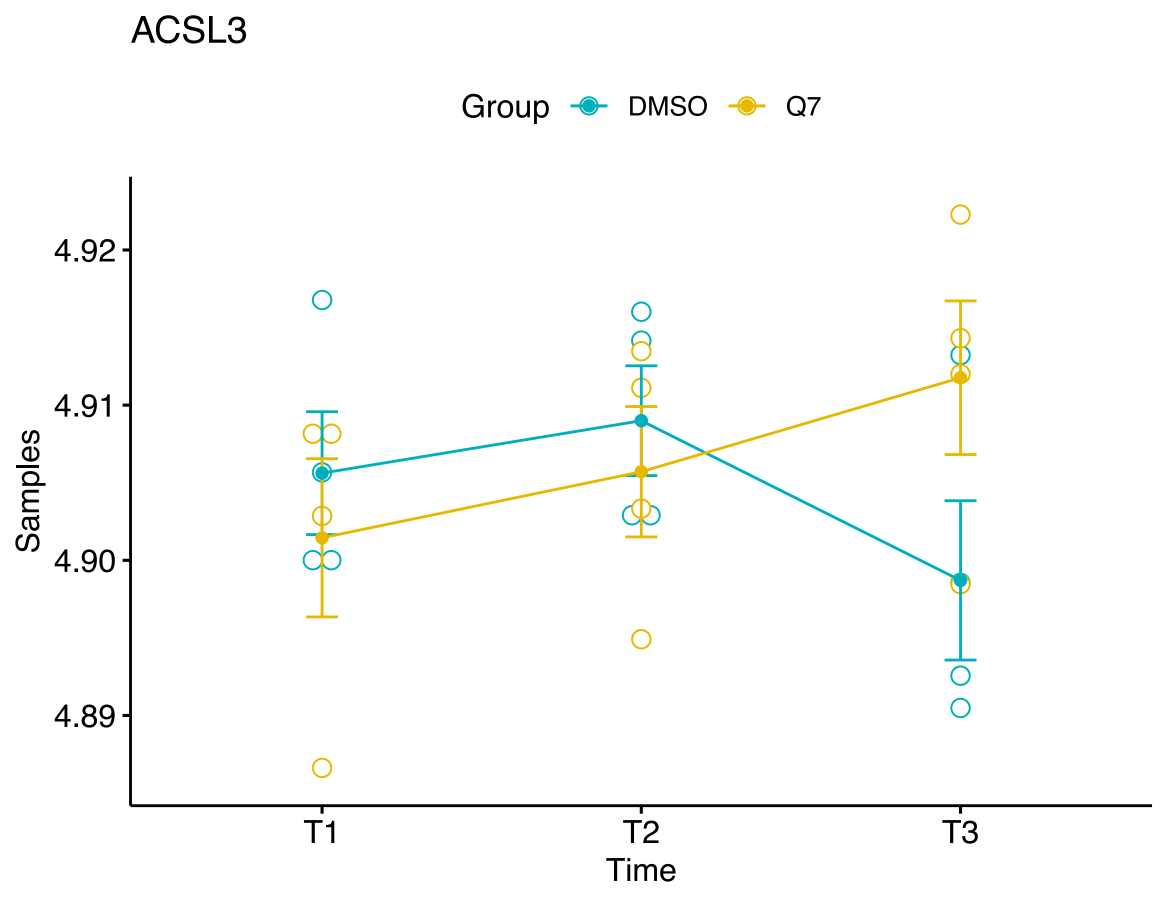
ACSL3
Group
DMSO
Q7
Samples
4.92
4.91
4.90
4.89
T1
T2
T3
Time

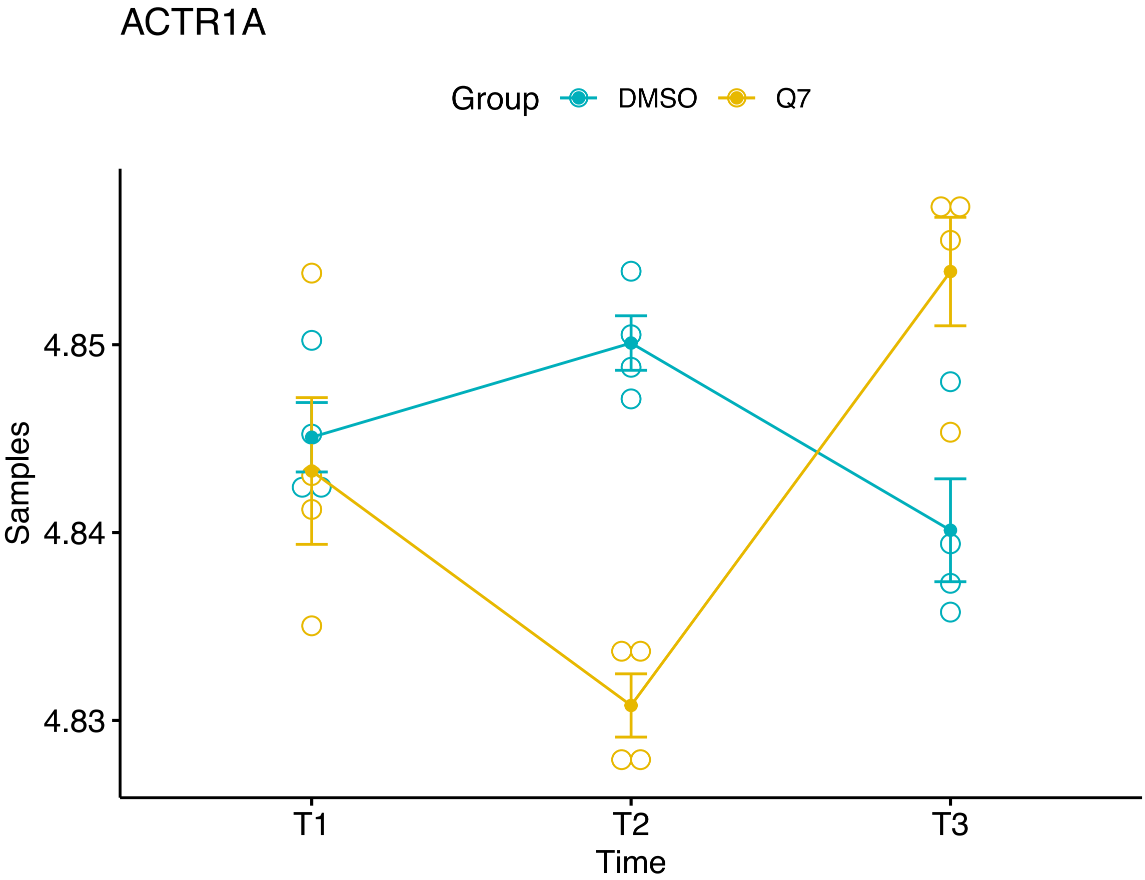

ACTR1A
Group
DMSO
Q7
Samples
4.85
4.84
4.83
T1
T2
T3
Time

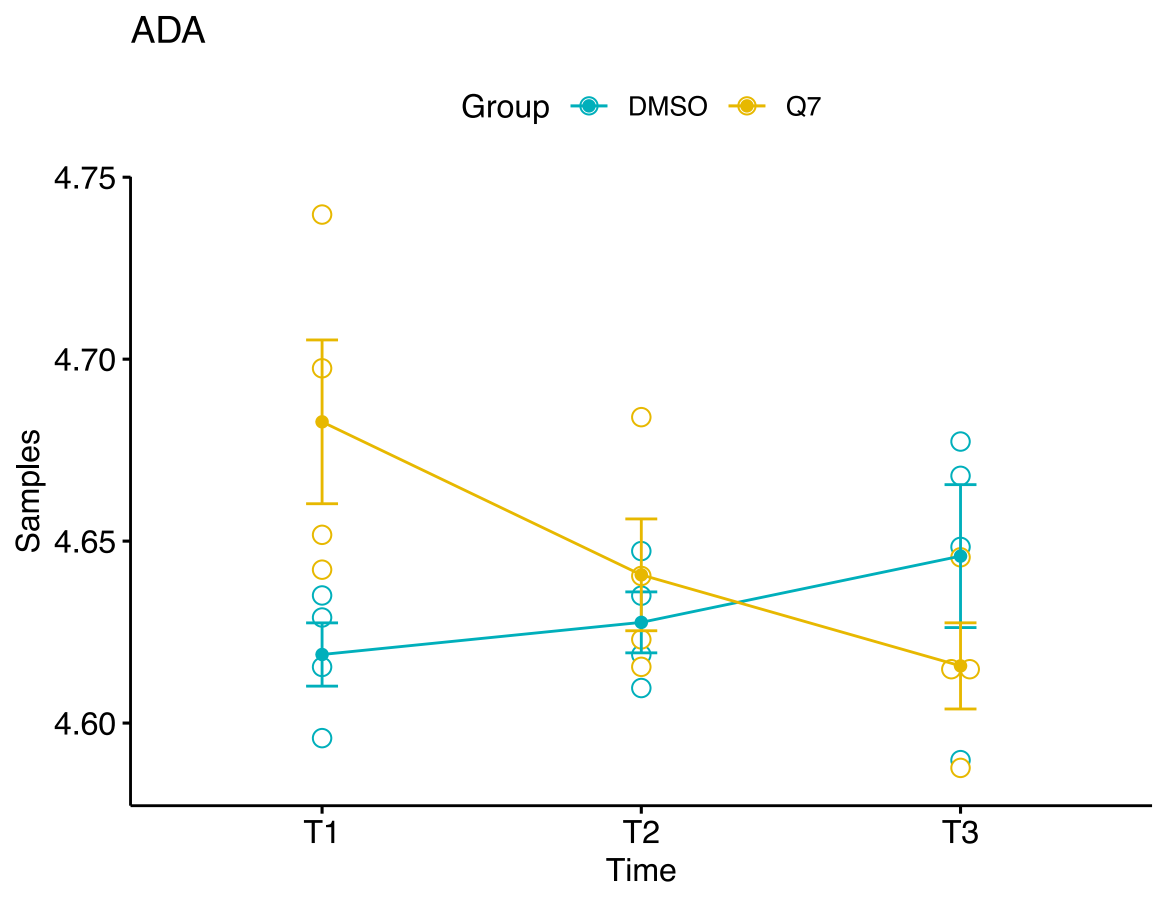
ADA
Group
DMSO
Q7
4.75
4.70
4.65
4.60
Samples
T1
T2
T3
Time

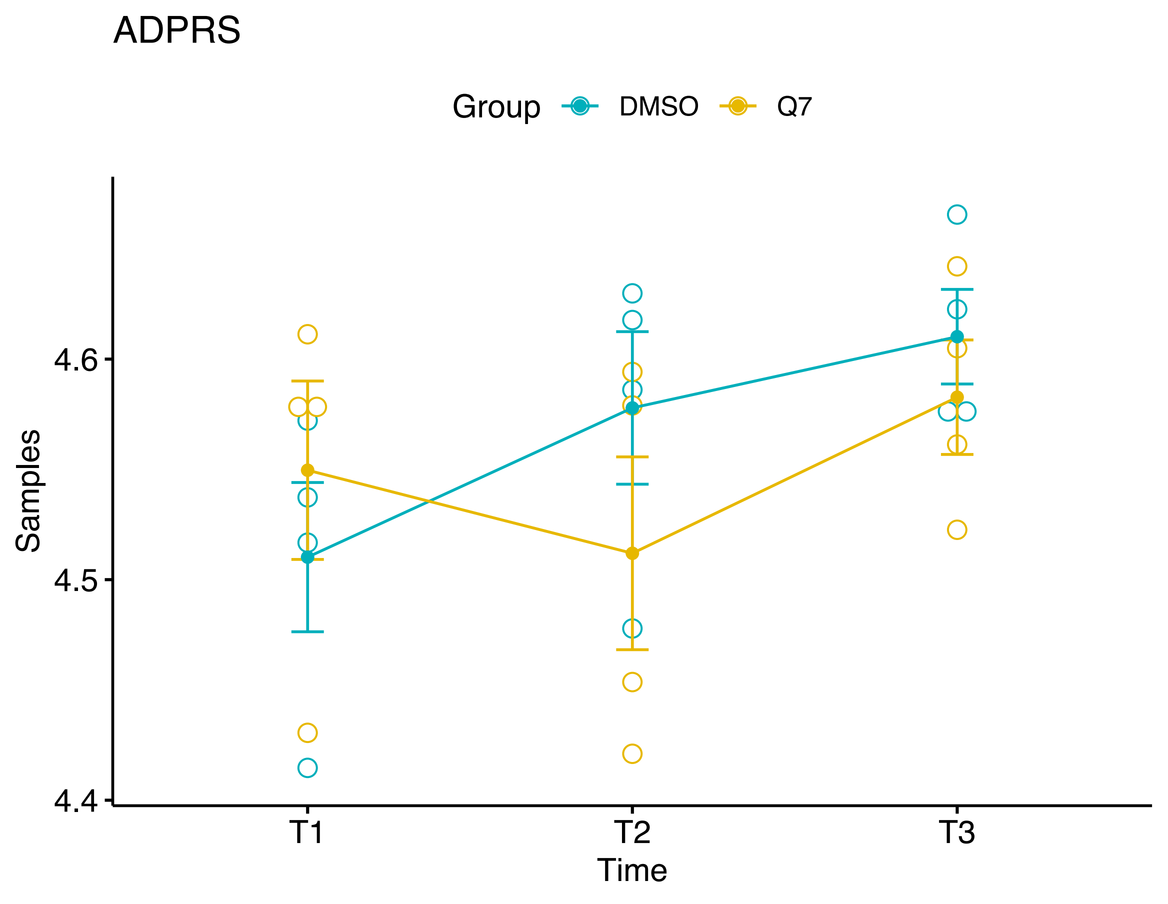

ADPRS
Group
DMSO
Q7
Samples
4.4
4.5
4.6
T1
T2
T3
Time

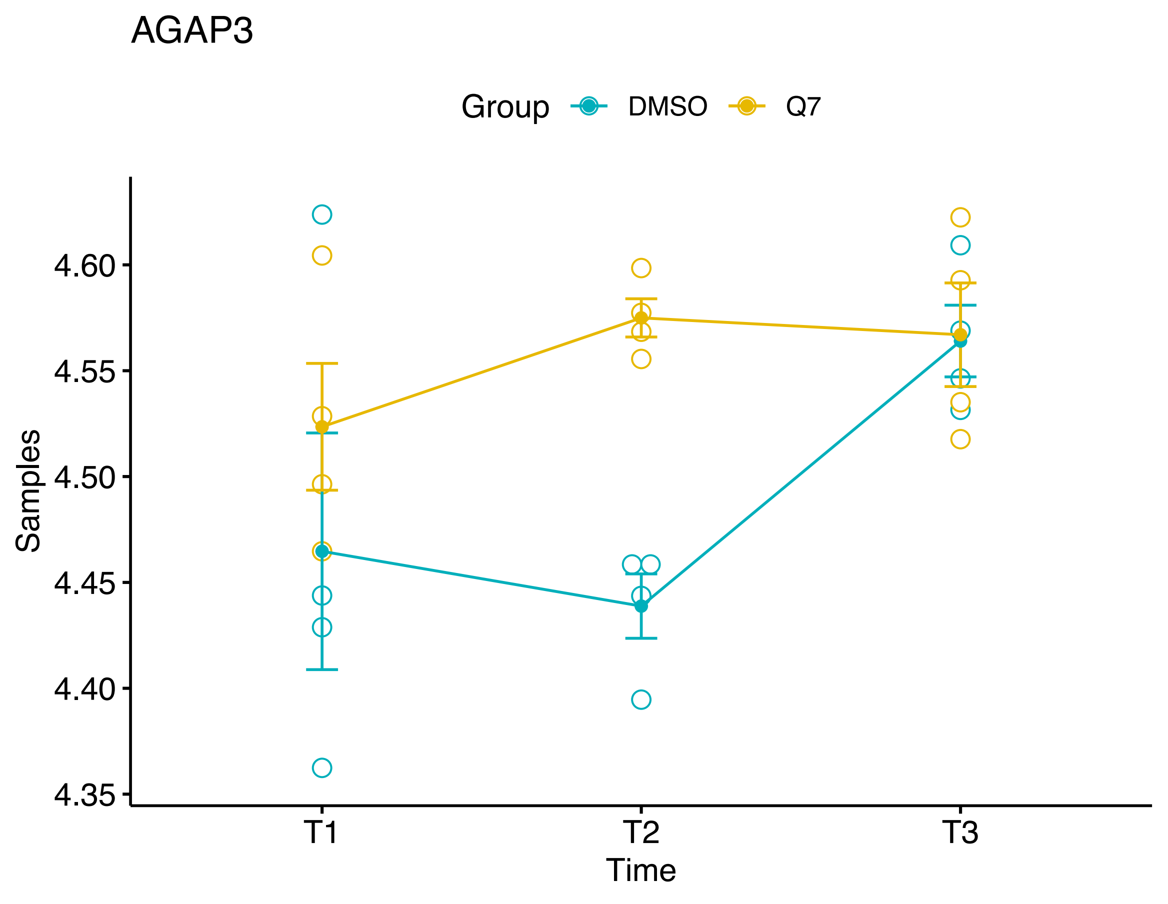
AGAP3
Group
DMSO
Q7
Samples
4.60
4.55
4.50
4.45
4.40
4.35
T1
T2
T3
Time

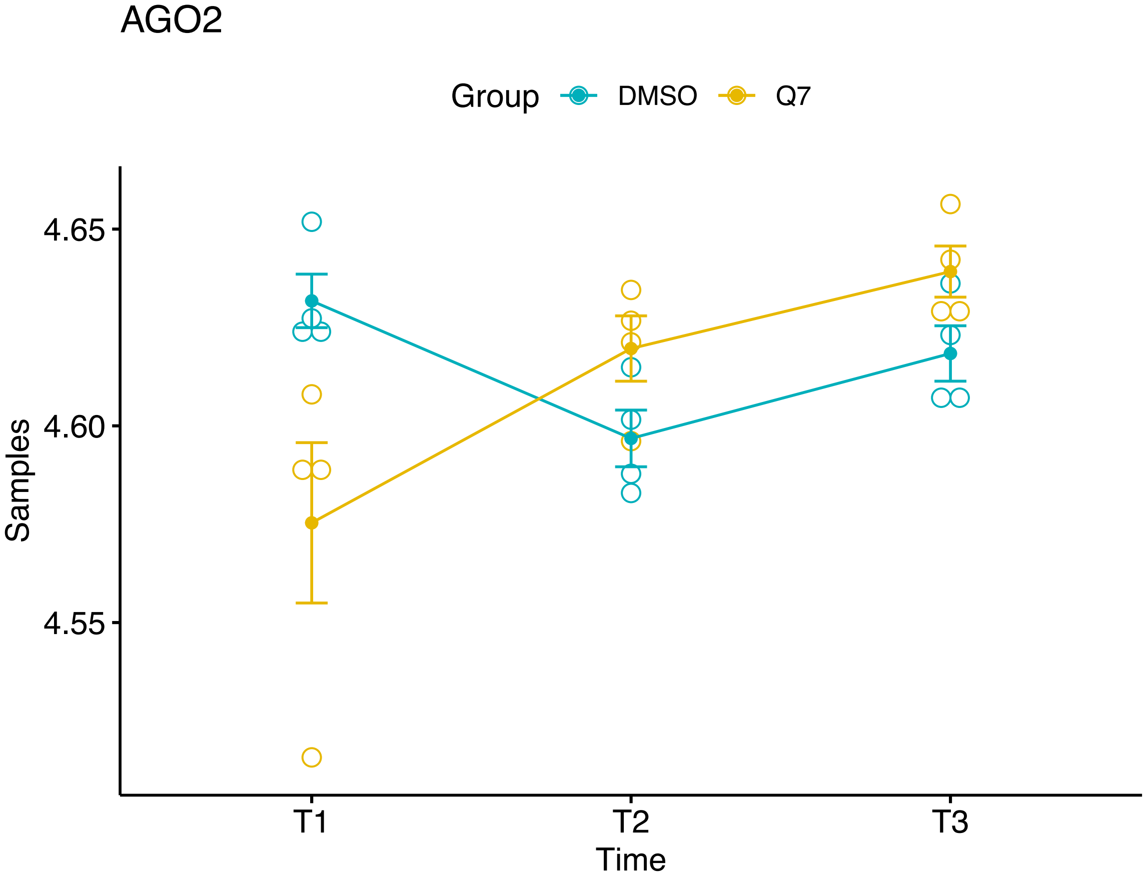
AGO2
Group
DMSO
Q7
4.65
4.60
4.55
Samples
T1
T2
T3
Time

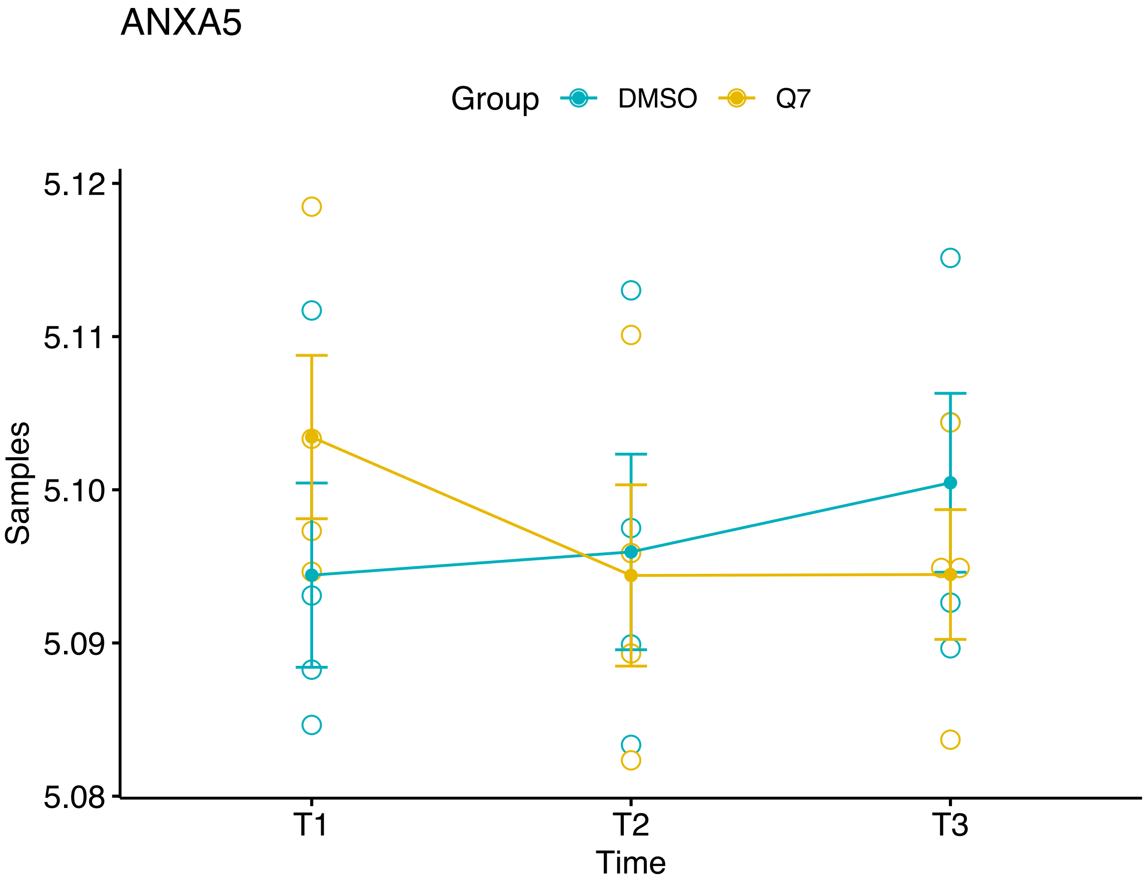
ANXA5
Group
DMSO
Q7
5.12
5.11
5.10
5.09
5.08
Samples
T1
T2
T3
Time

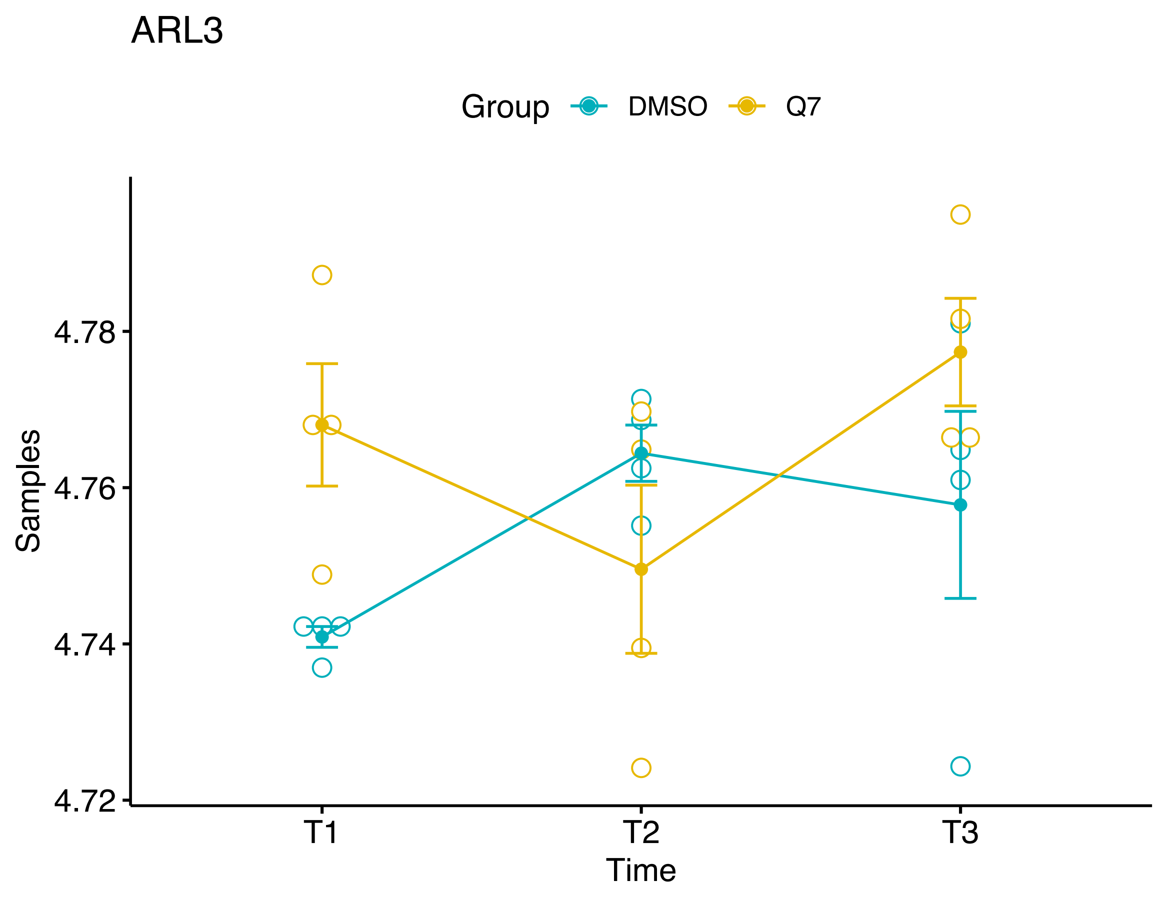

ARL3
Group
DMSO
Q7
Samples
4.72
4.74
4.76
4.78
T1
T2
T3
Time

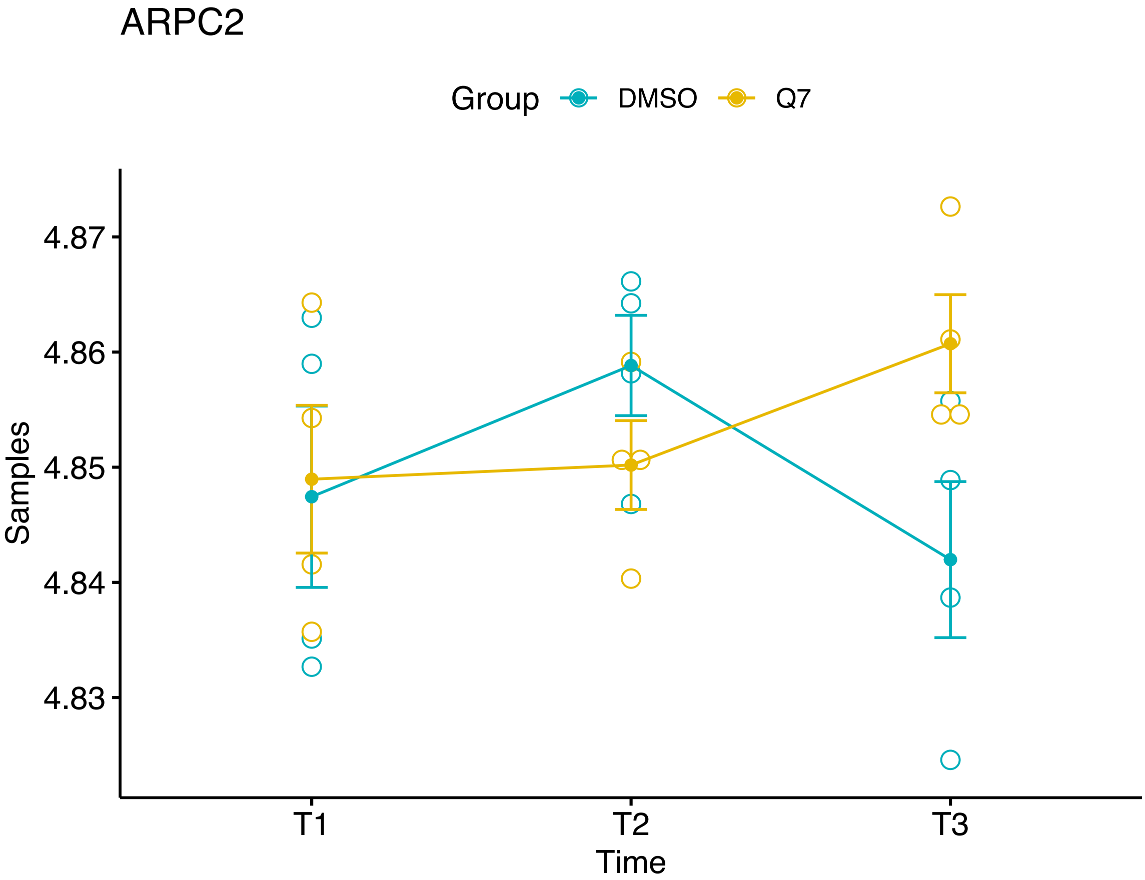

ARPC2
Group
DMSO
Q7
Samples
4.87
4.86
4.85
4.84
4.83
T1
T2
T3
Time

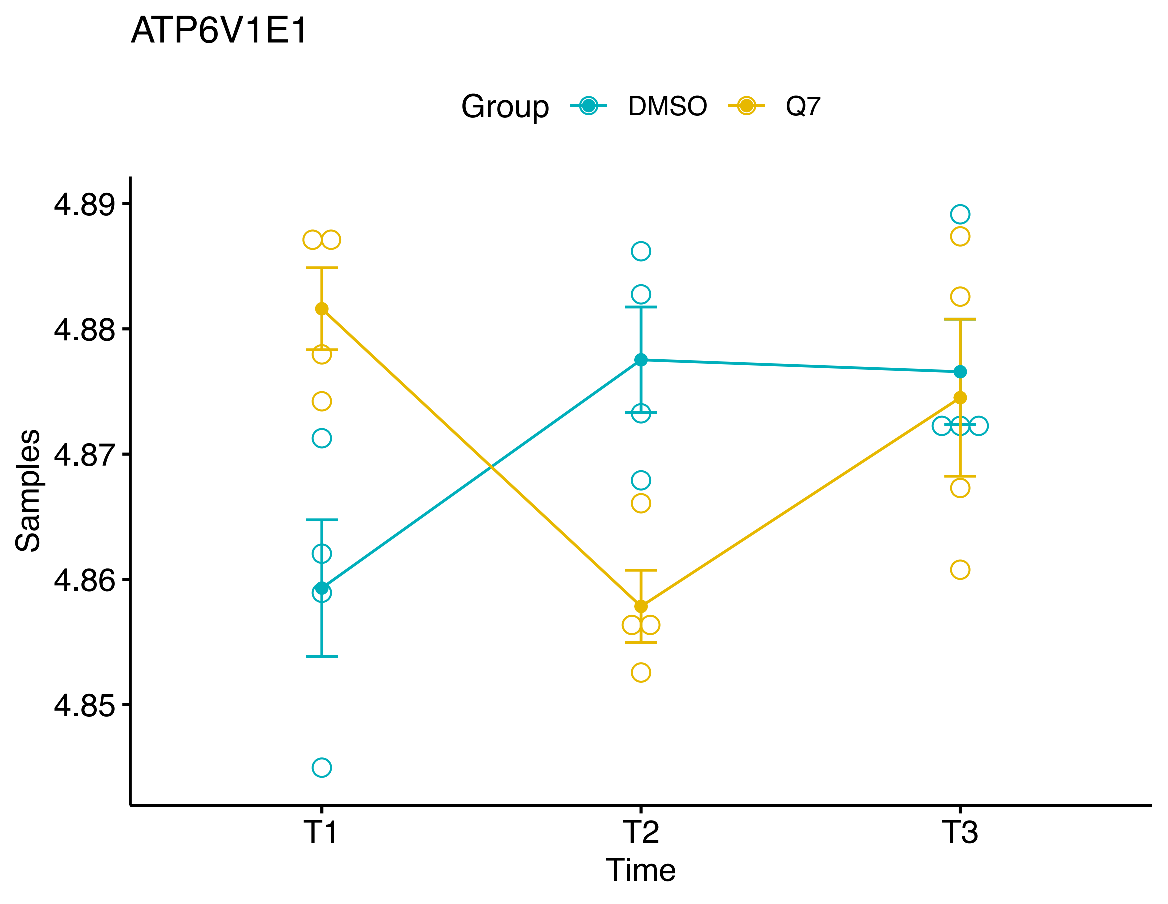
ATP6V1E1
Group
DMSO
Q7
Samples
4.89
4.88
4.87
4.86
4.85
T1
T2
T3
Time

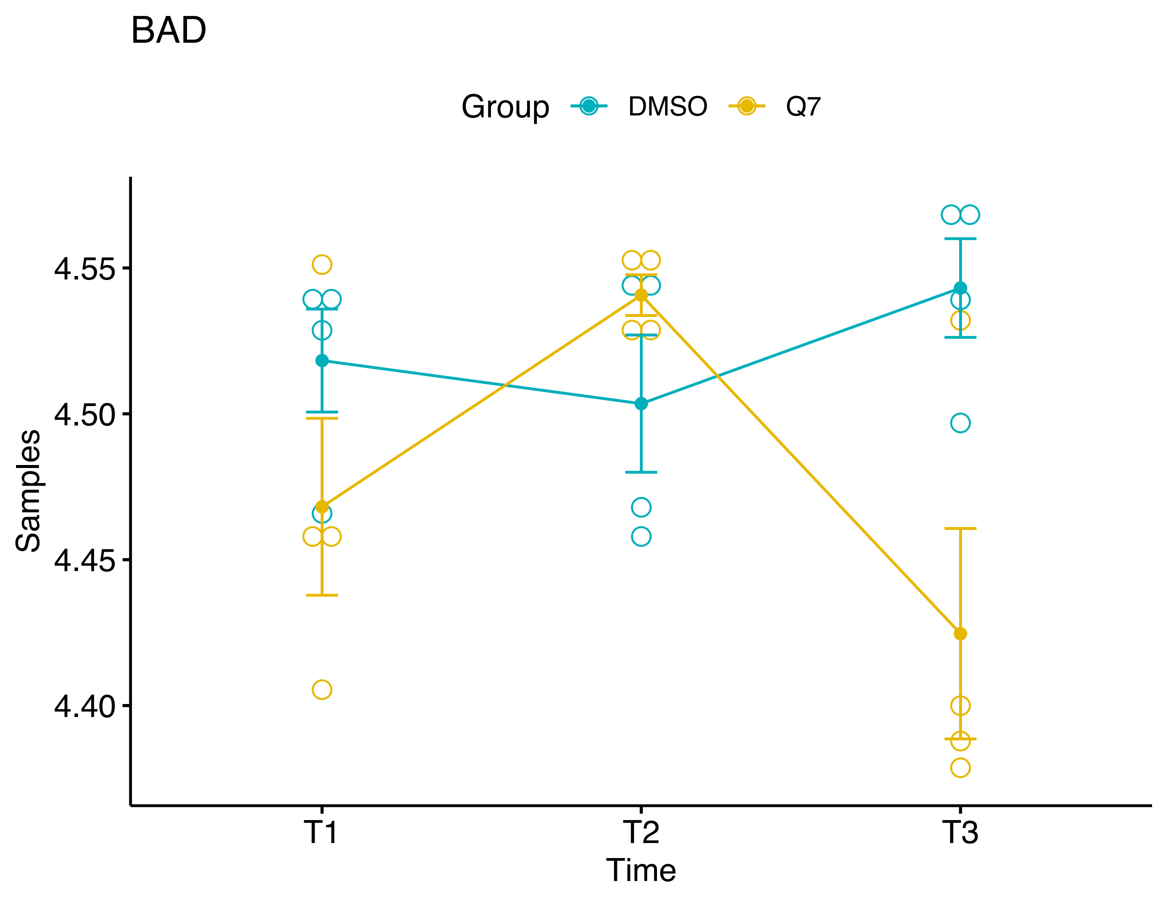
BAD
Group
DMSO
Q7
Samples
4.55
4.50
4.45
4.40
T1
T2
T3
Time

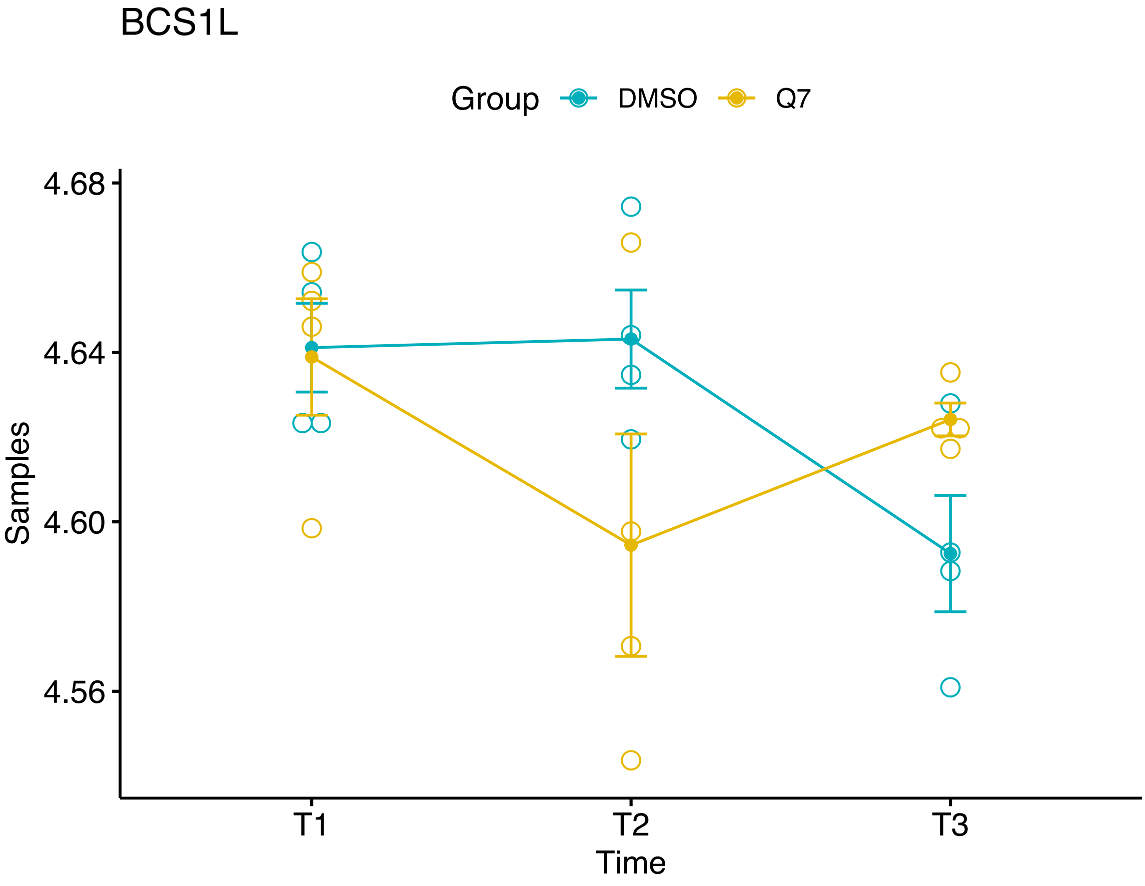

BCS1L
Group
DMSO
Q7
4.68
4.64
4.60
4.56
Samples
T1
T2
T3
Time

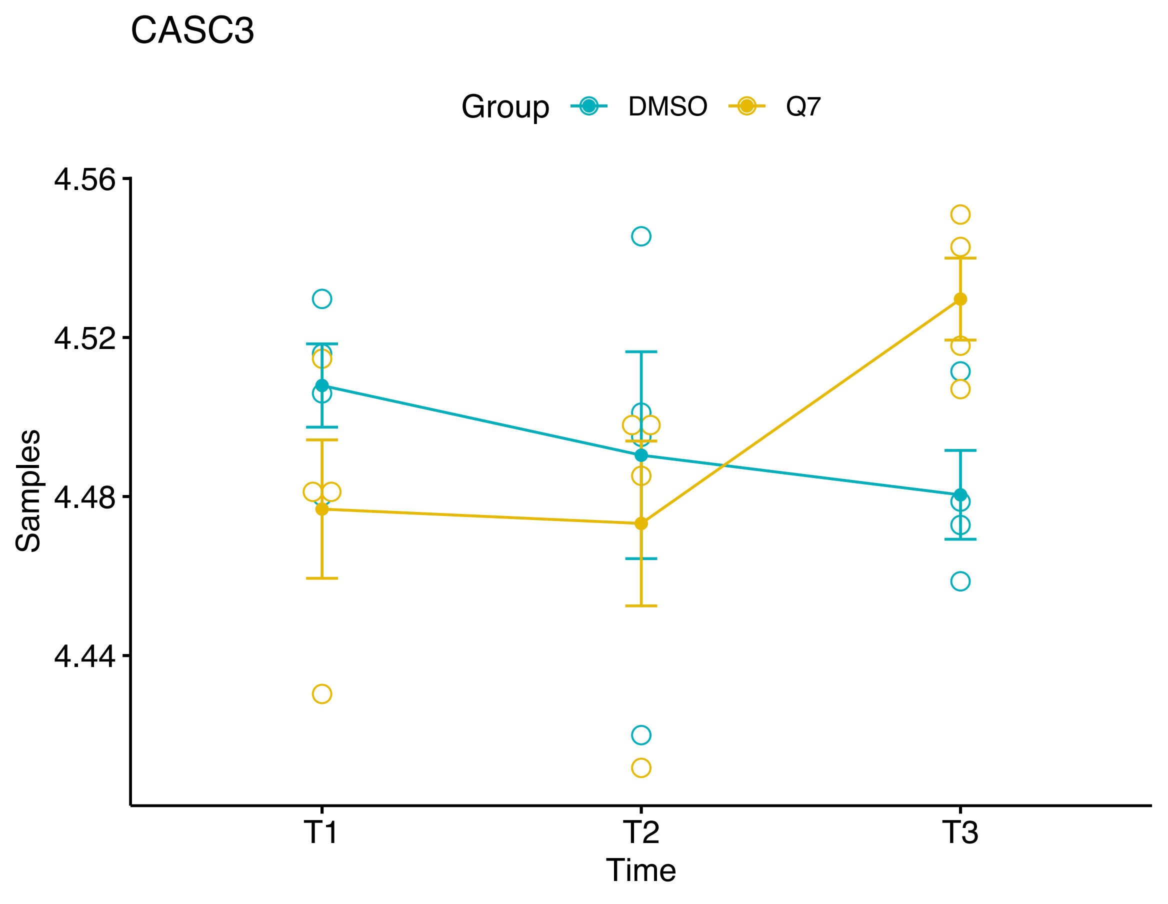
CASC3
Group
DMSO
Q7
4.56
4.52
4.48
4.44
Samples
T1
T2
T3
Time

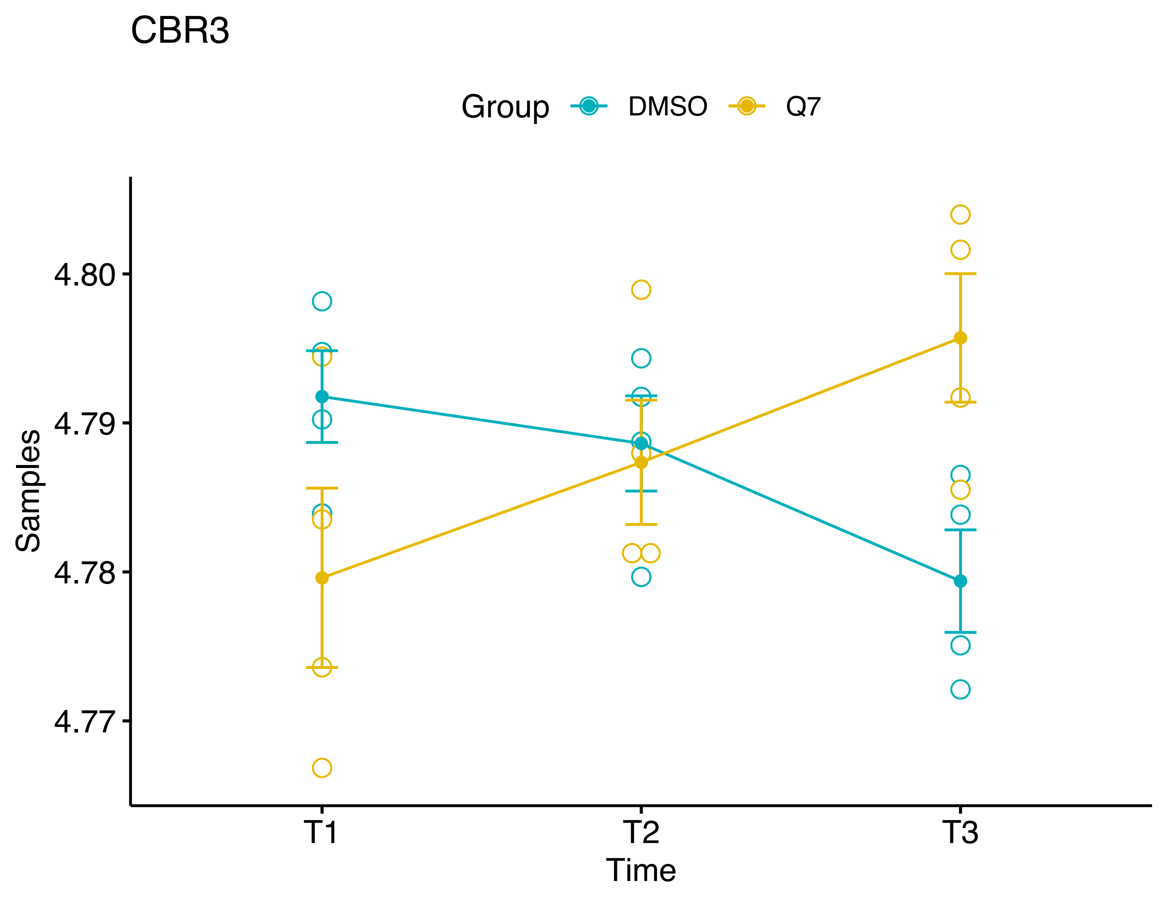
CBR3
Group
DMSO
Q7
Samples
4.80
4.79
4.78
4.77
T1
T2
T3
Time

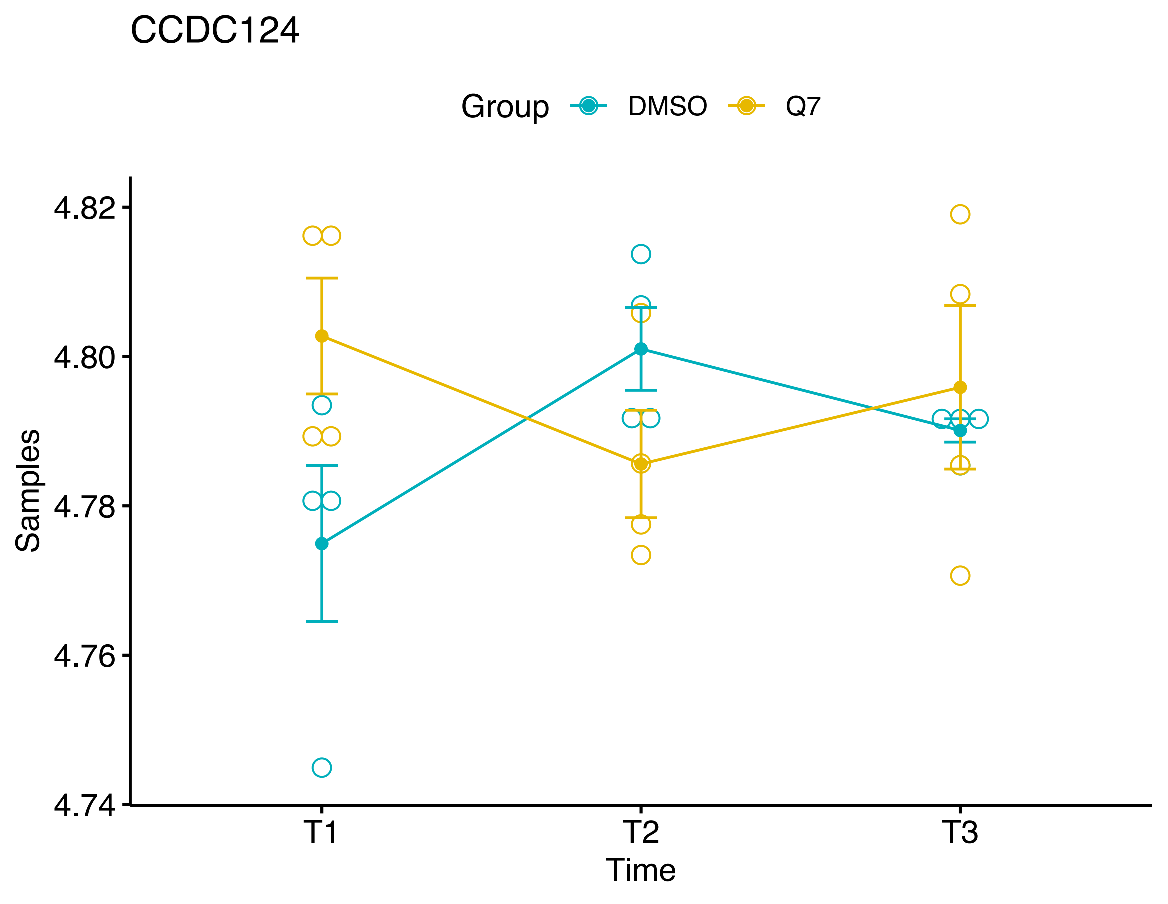

CCDC124
Group
DMSO
Q7
4.82
4.80
4.78
4.76
4.74
Samples
T1
T2
T3
Time

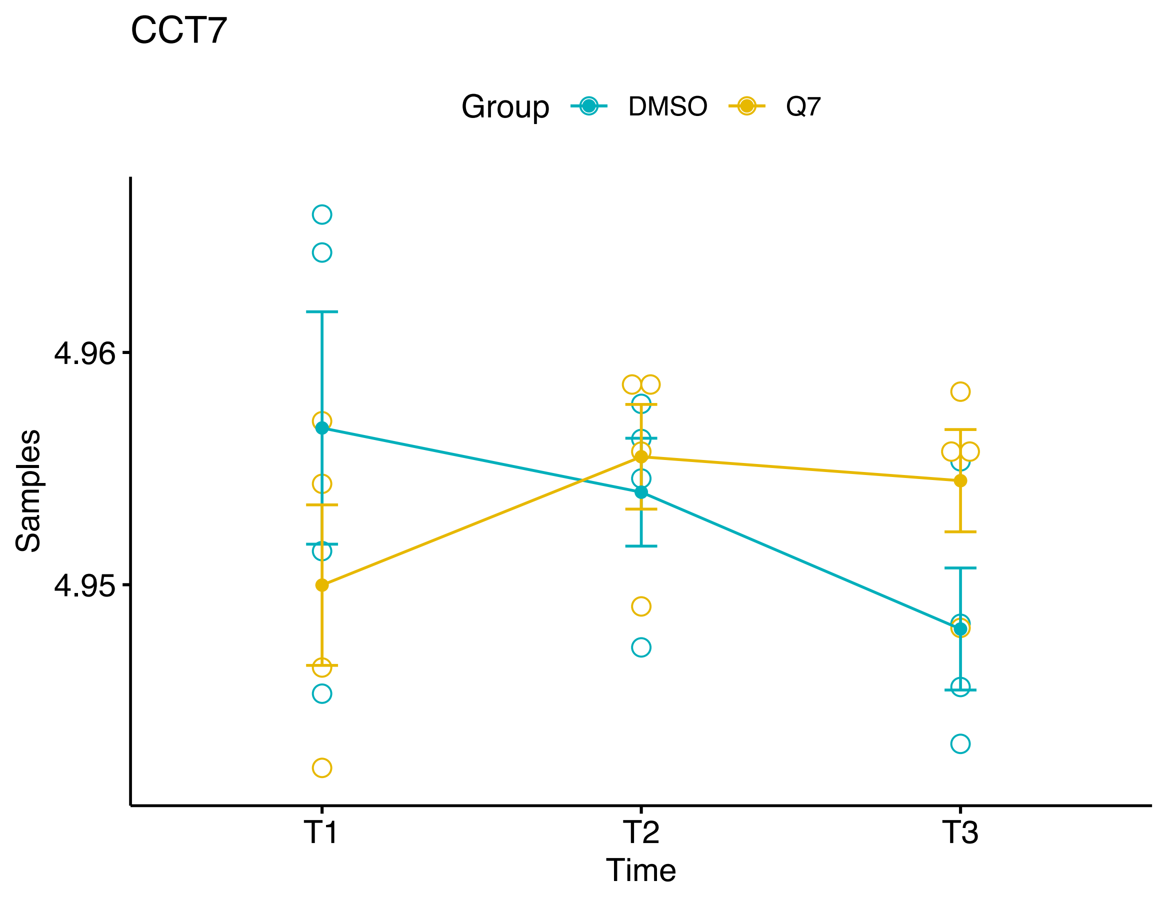

CCT7
Group
DMSO
Q7
4.96
4.95
Samples
T1
T2
T3
Time

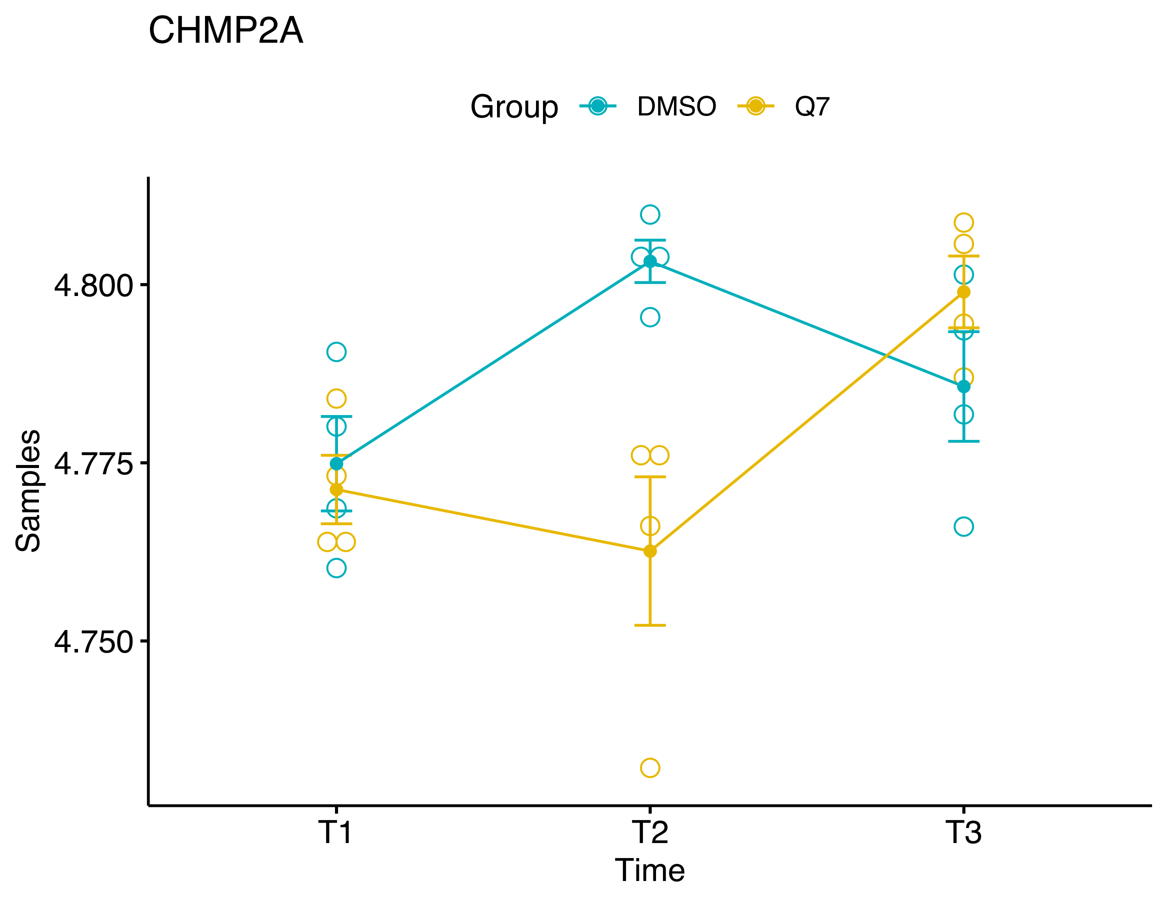
CHMP2A
Group
DMSO
Q7
4.800
4.775
4.750
Samples
T1
T2
T3
Time

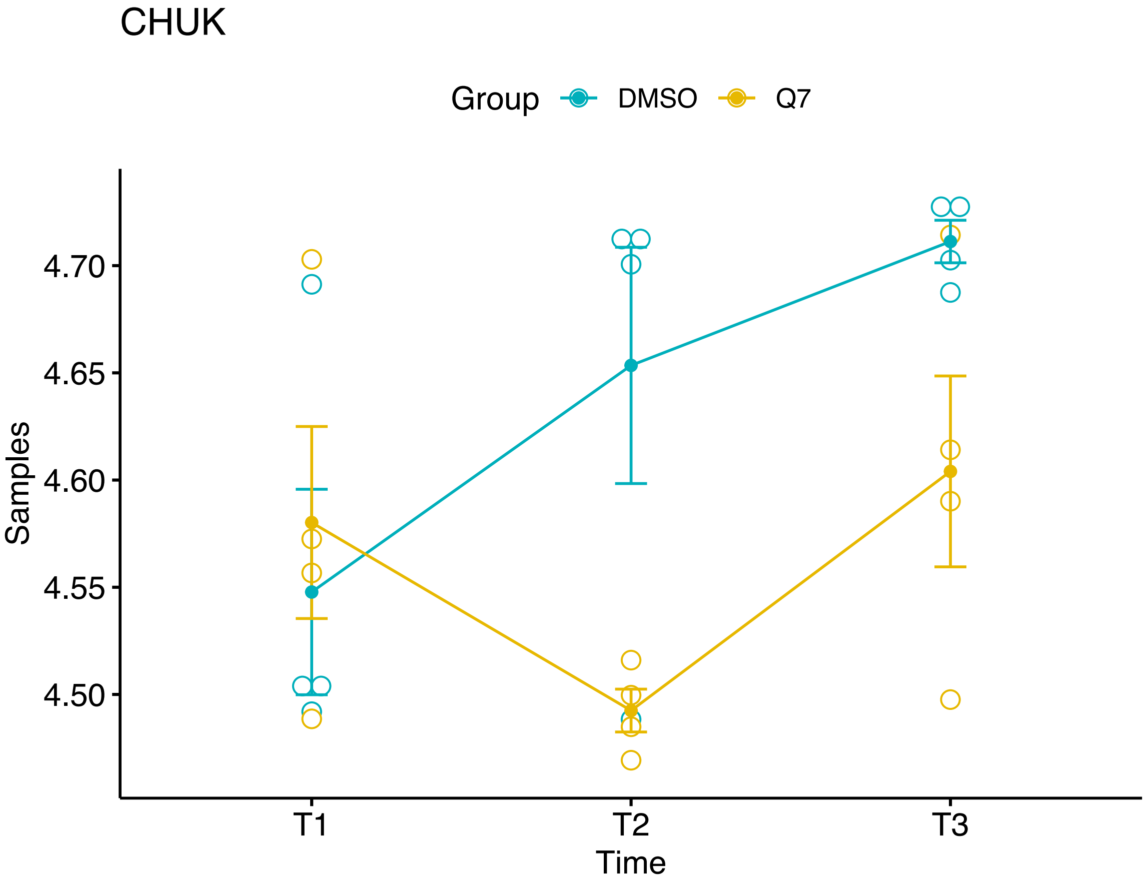
CHUK
Group
DMSO
Q7
Samples
4.70
4.65
4.60
4.55
4.50
T1
T2
T3
Time

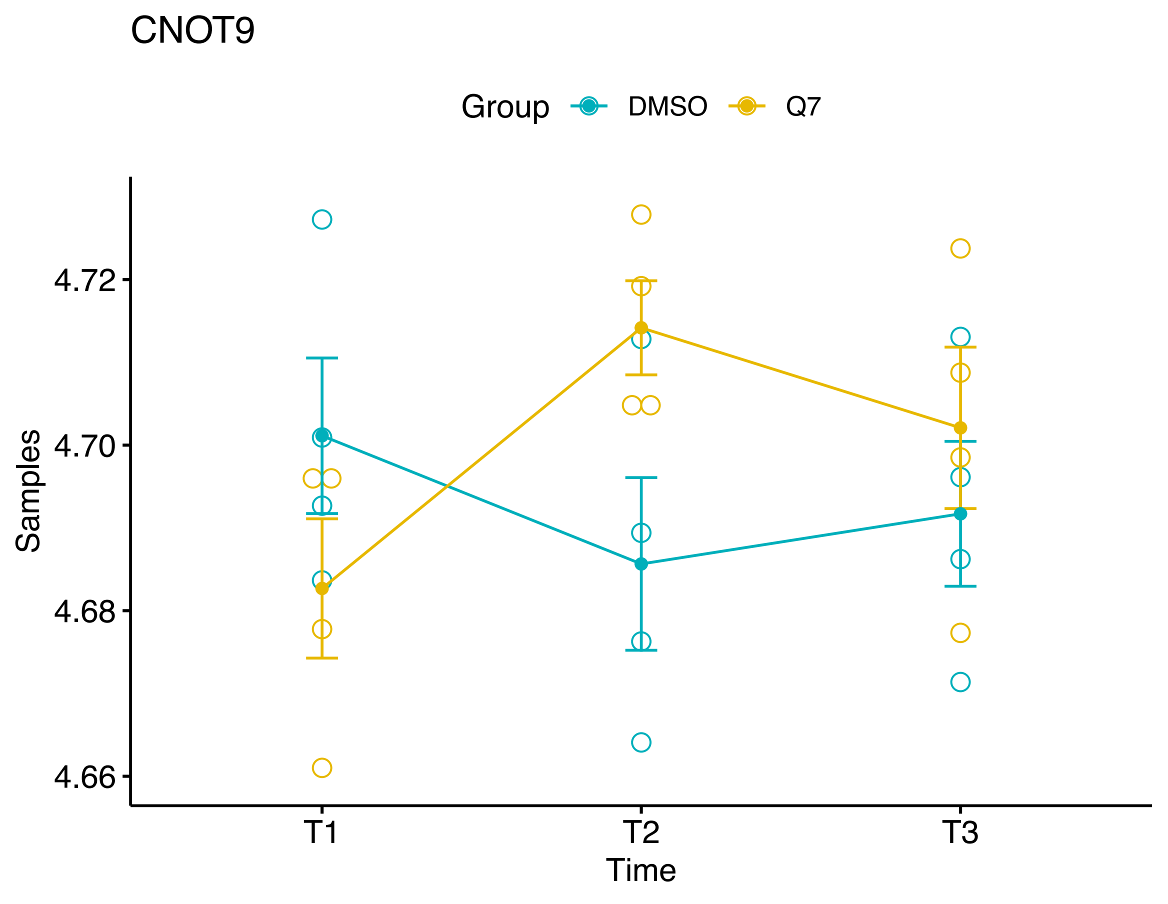
CNOT9
Group
DMSO
Q7
Samples
4.72
4.70
4.68
4.66
T1
T2
T3
Time

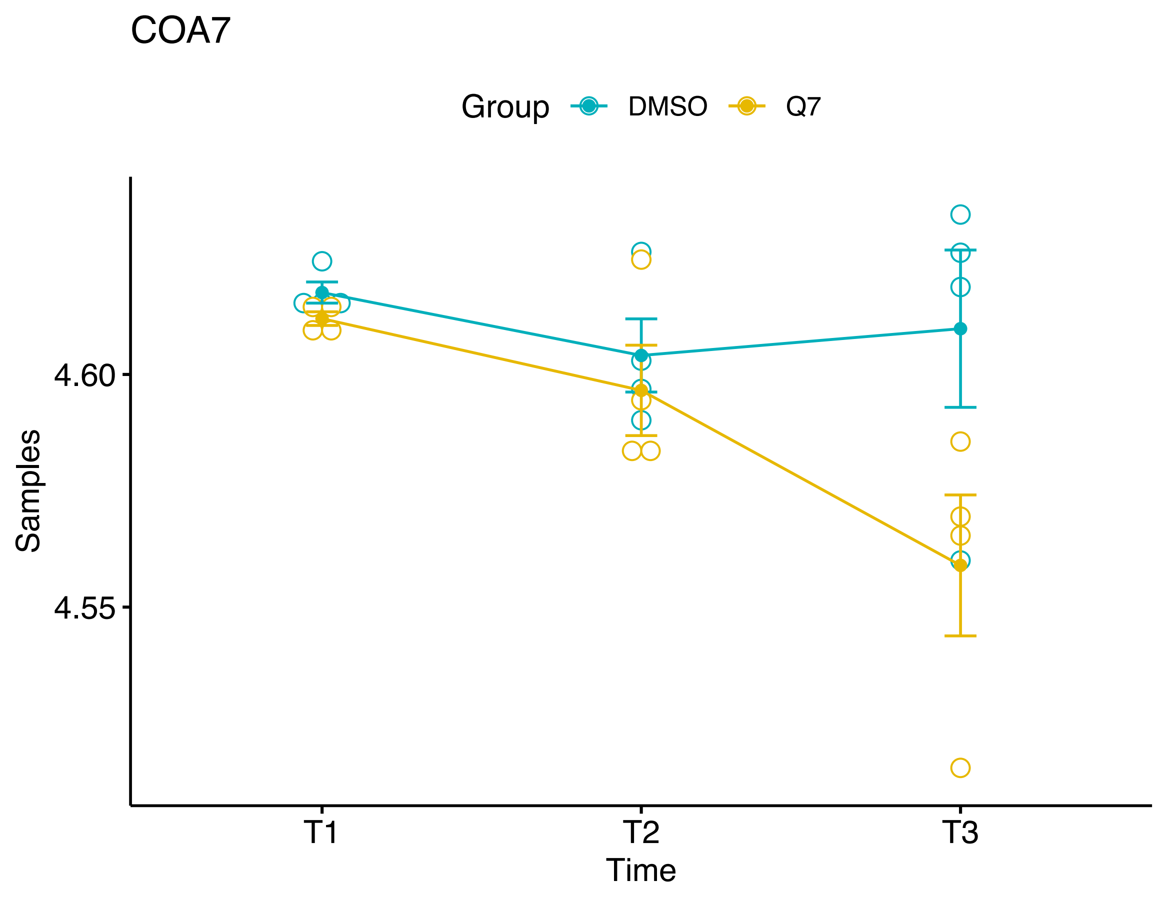

COA7
Group
DMSO
Q7
4.60
4.55
Samples
T1
T2
T3
Time

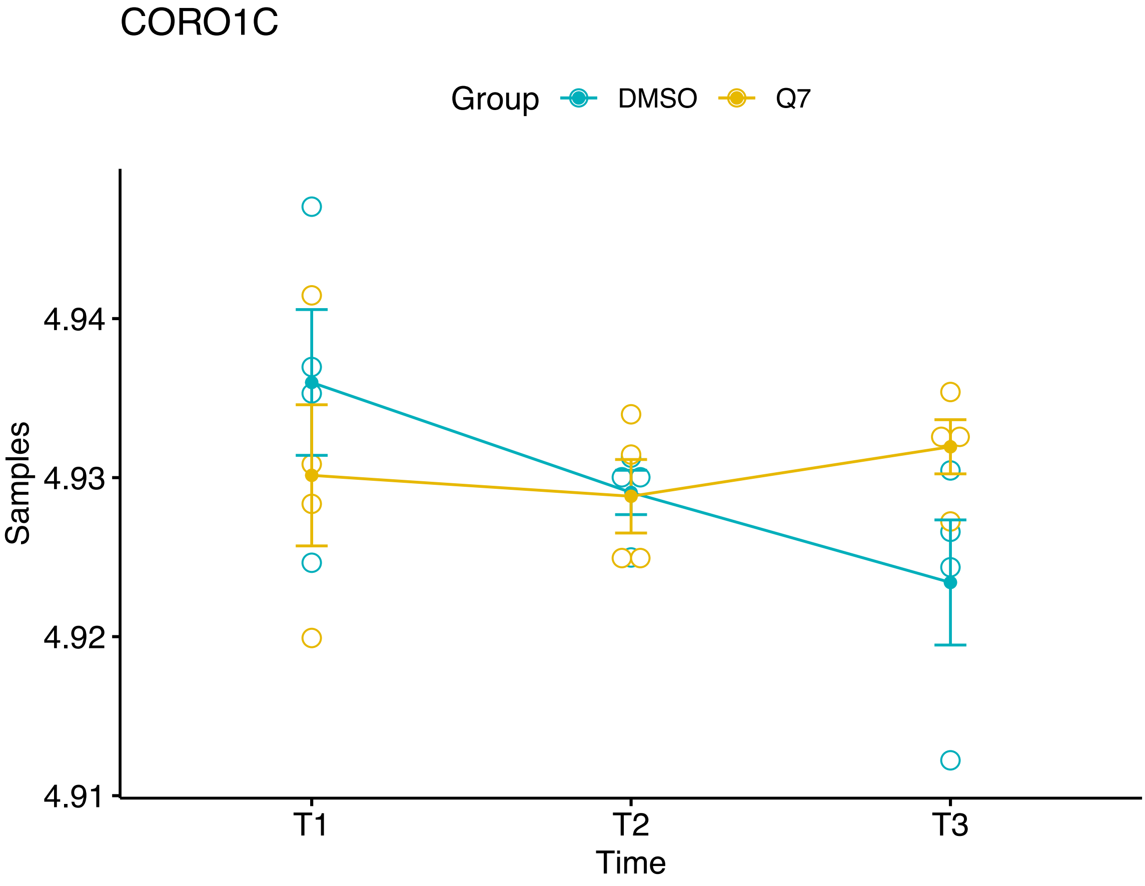

CORO1C
Group
DMSO
Q7
Samples
4.94
4.93
4.92
4.91
T1
T2
T3
Time

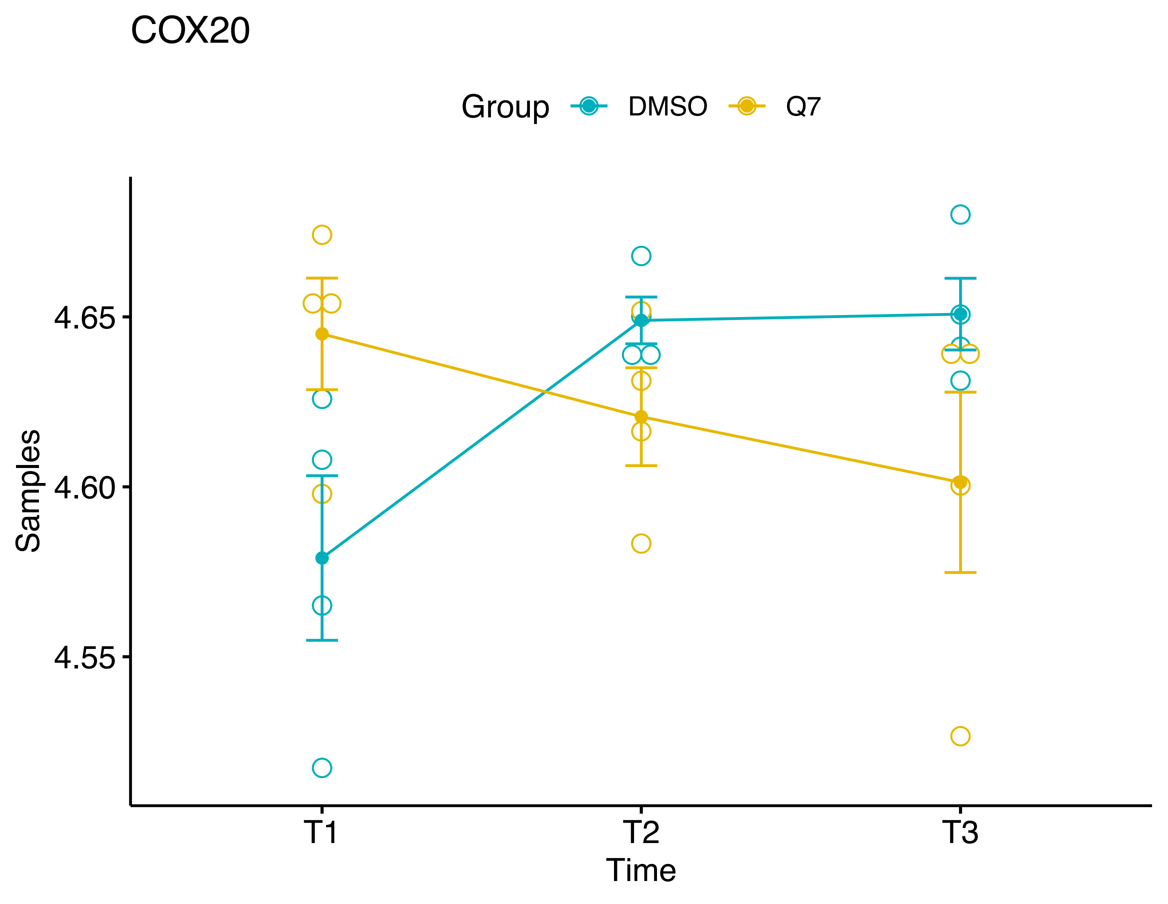
COX20
Group
DMSO
Q7
Samples
4.65
4.60
4.55
T1
T2
T3
Time

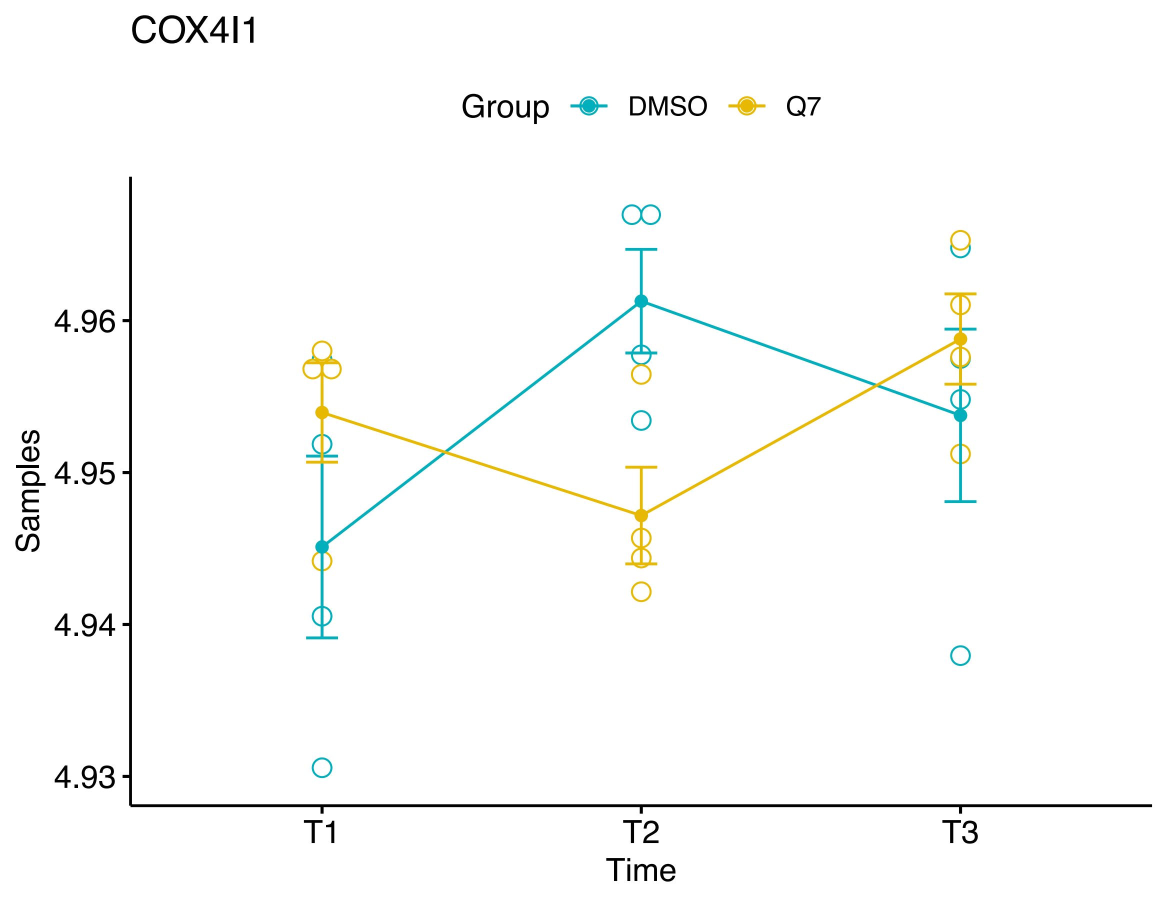
COX4I1
Group
DMSO
Q7
Samples
4.96
4.95
4.94
4.93
T1
T2
T3
Time

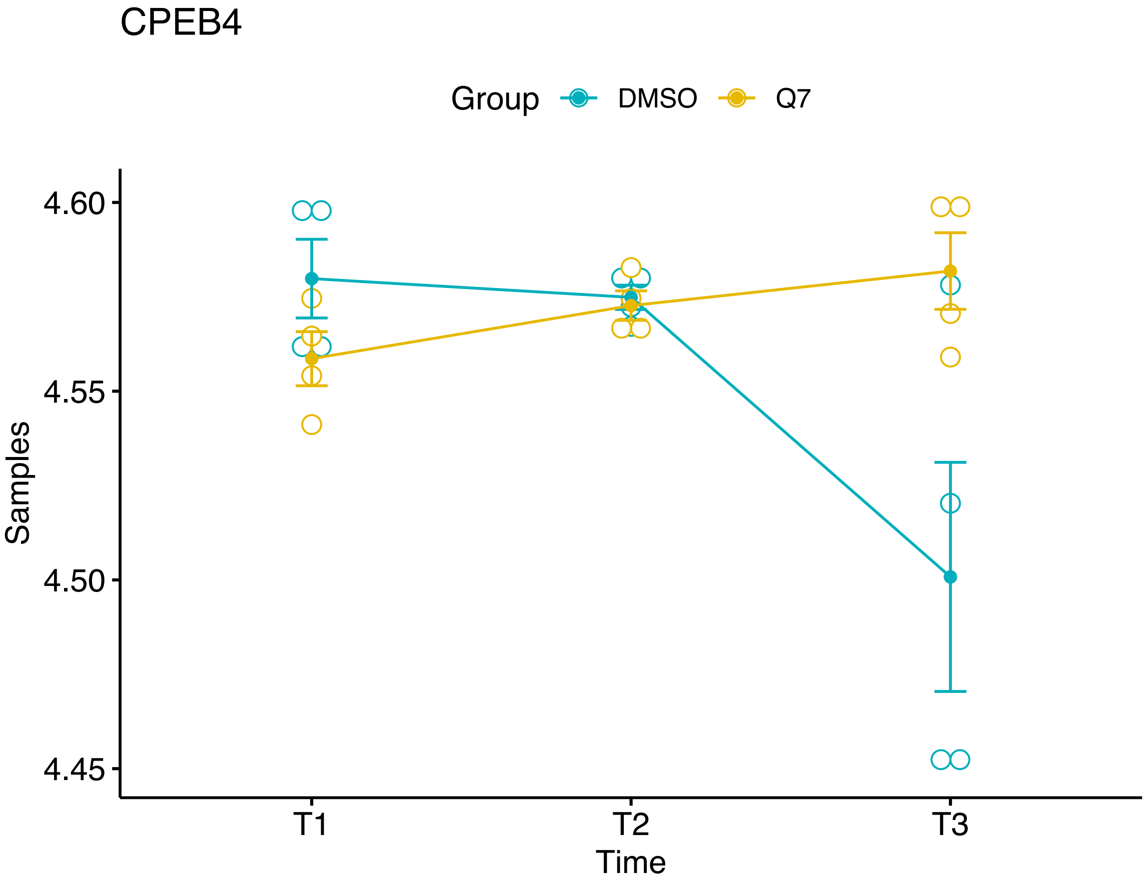

CPEB4
Group
DMSO
Q7
Samples
4.60
4.55
4.50
4.45
T1
T2
T3
Time

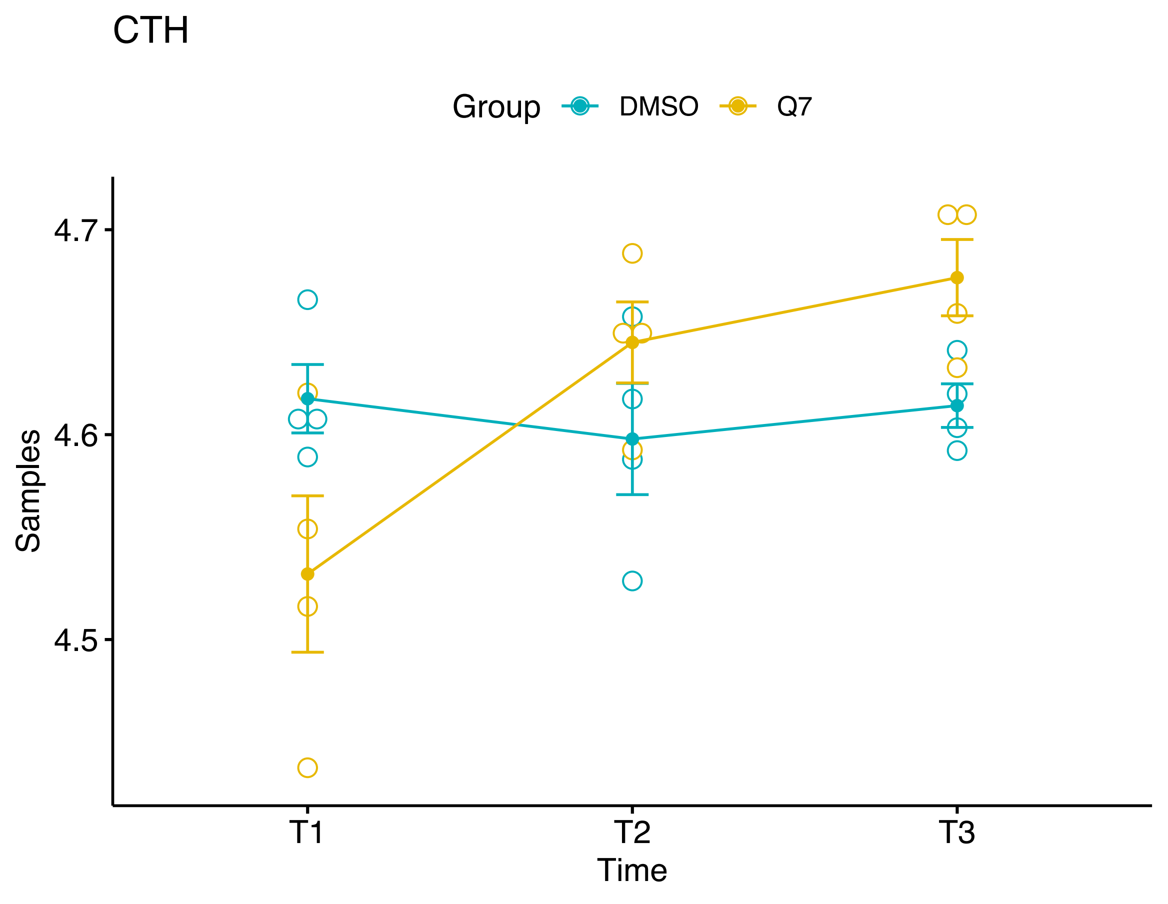
CTH
Group
DMSO
Q7
4.7
4.6
4.5
Samples
T1
T2
T3
Time

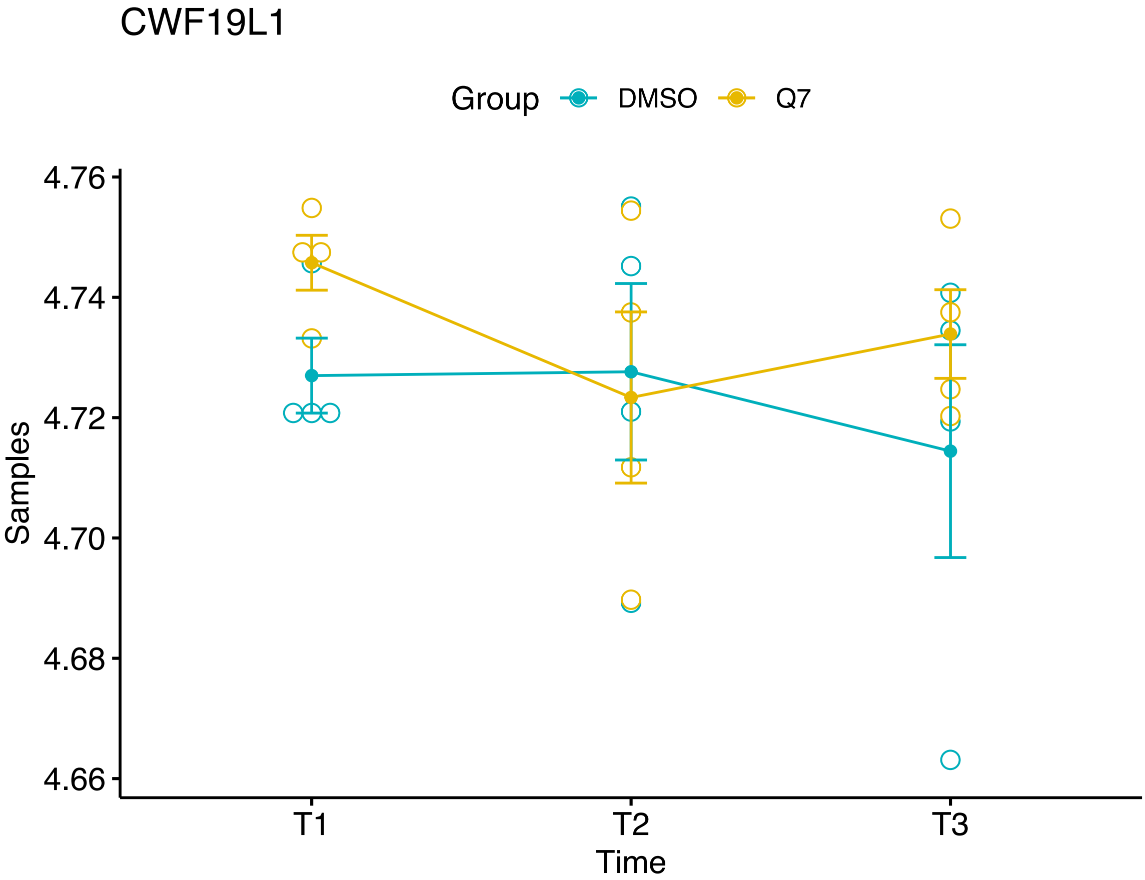

CWF19L1
Group
DMSO
Q7
4.76
4.74
4.72
4.70
4.68
4.66
Samples
T1
T2
T3
Time

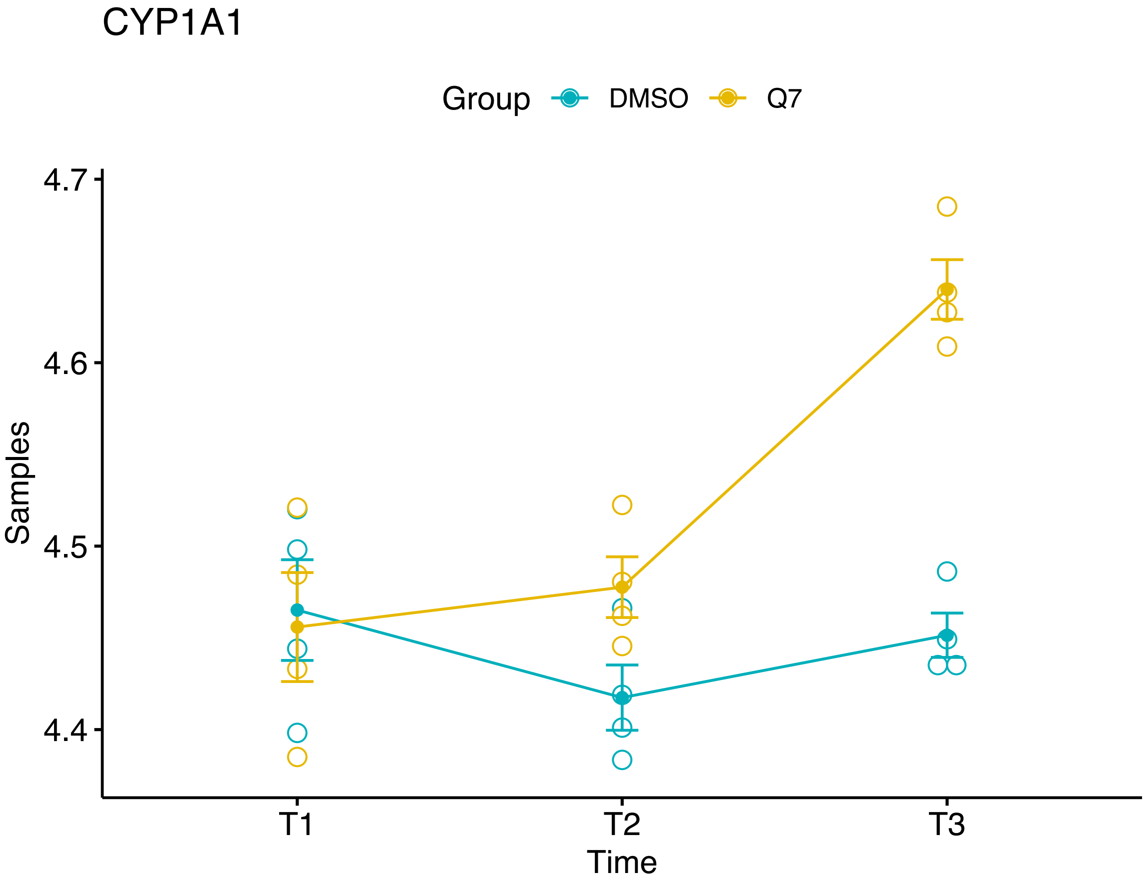
CYP1A1
Group
DMSO
Q7
4.7
4.6
4.5
4.4
Samples
T1
T2
T3
Time

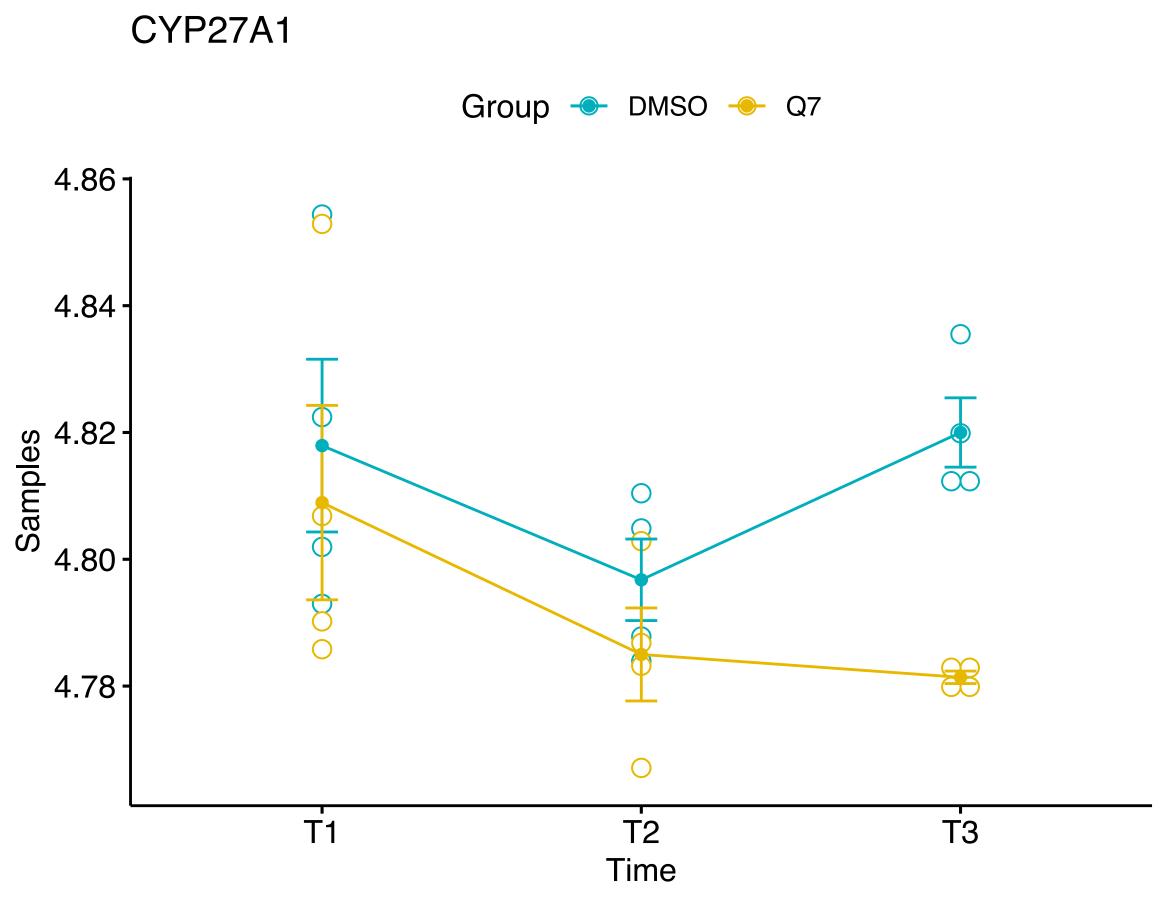

CYP27A1
Group
DMSO
Q7
4.86
4.84
4.82
4.80
4.78
Samples
T1
T2
T3
Time

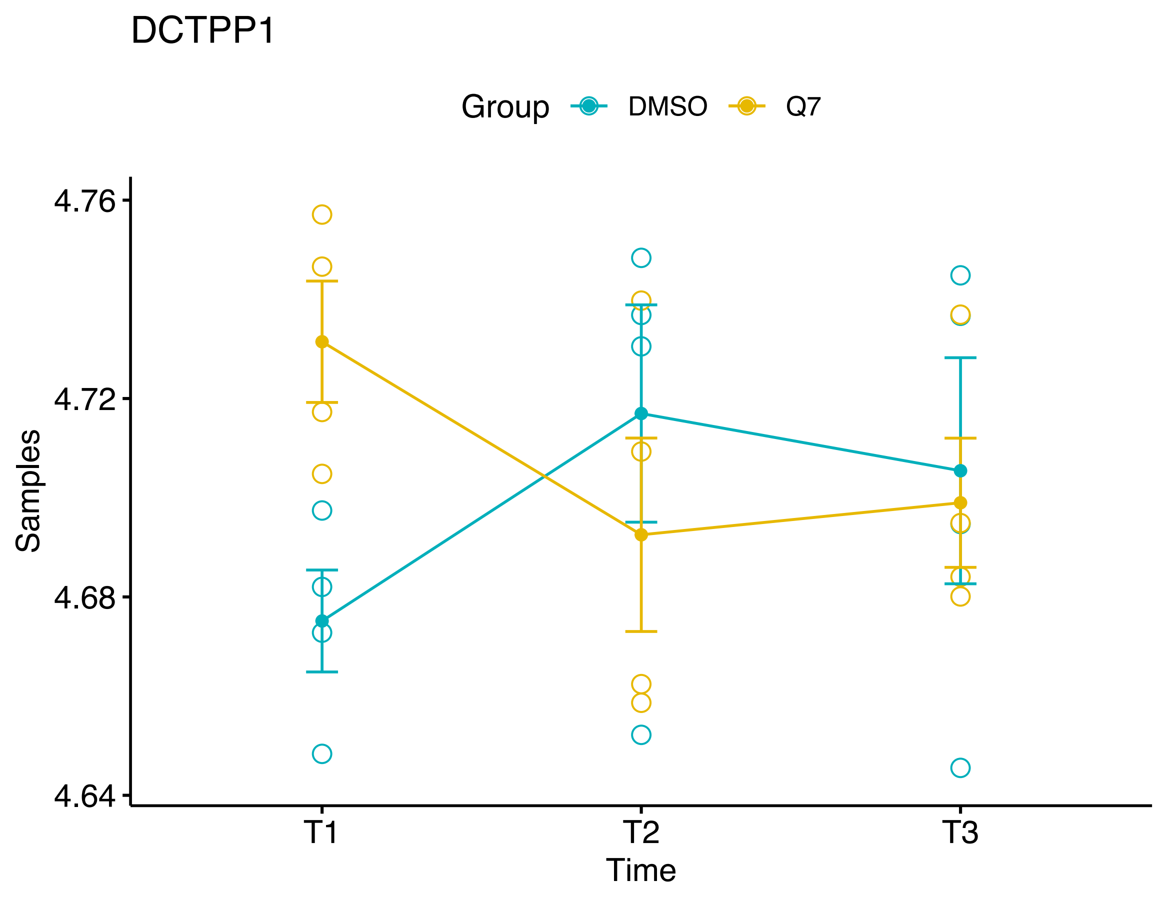
DCTPP1
Group
DMSO
Q7
4.76
4.72
4.68
4.64
Samples
T1
T2
T3
Time

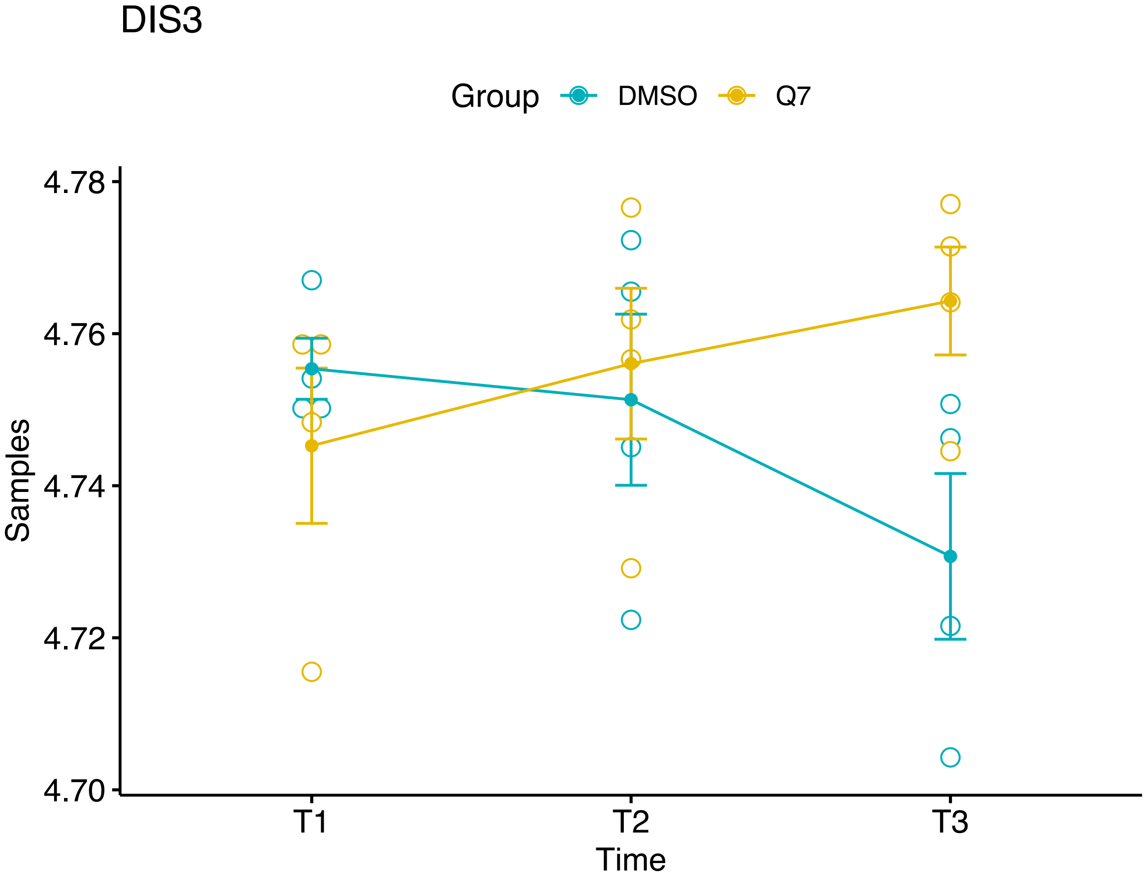

DIS3
Group
DMSO
Q7
4.78
4.76
4.74
4.72
4.70
Samples
T1
T2
T3
Time

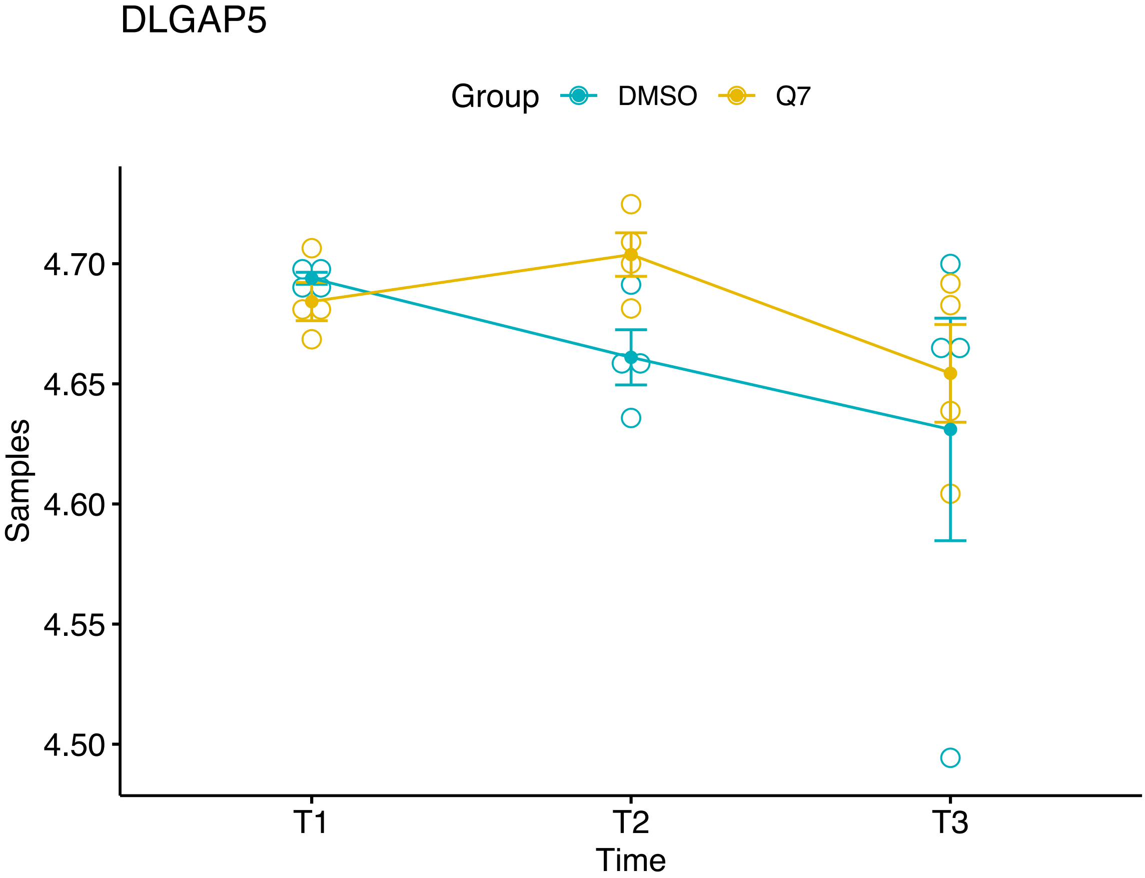
DLGAP5
Group
DMSO
Q7
Samples
4.70
4.65
4.60
4.55
4.50
T1
T2
T3
Time

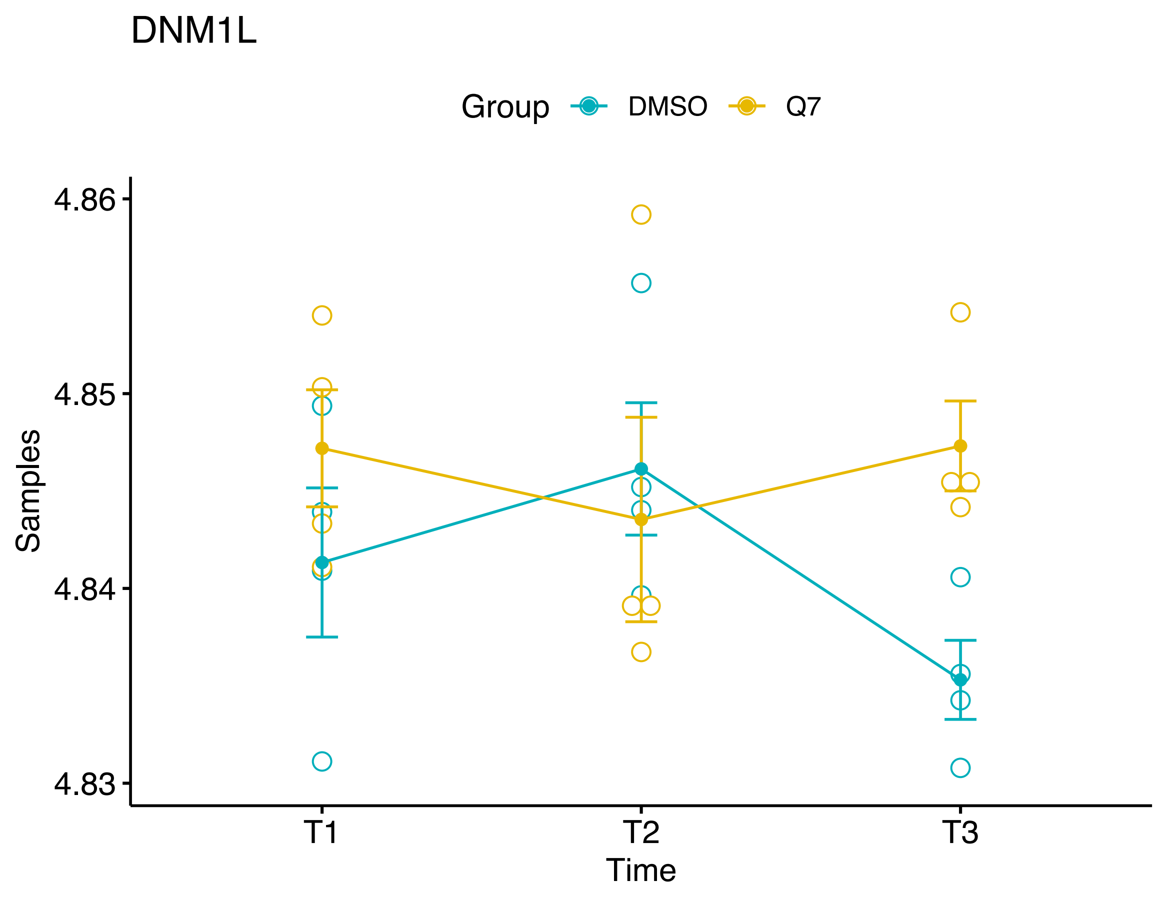
DNM1L
Group
DMSO
Q7
Samples
4.86
4.85
4.84
4.83
T1
T2
T3
Time

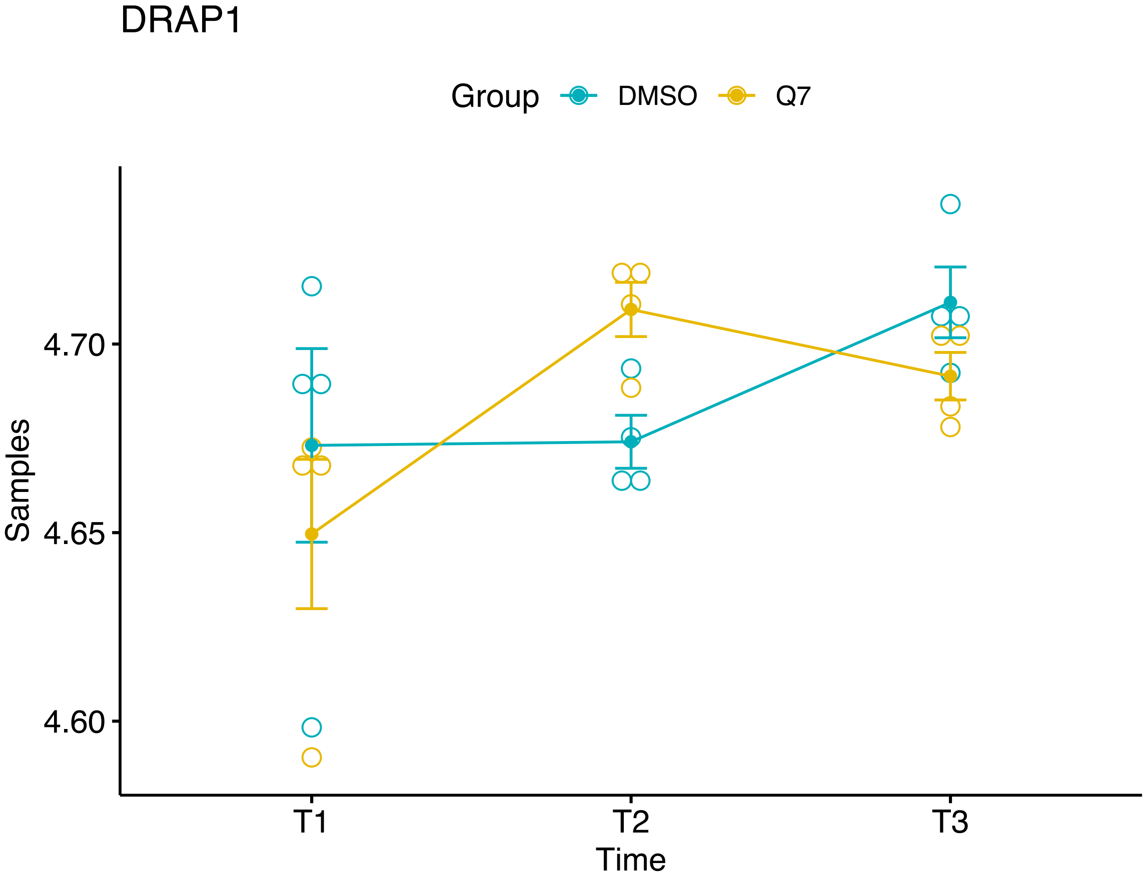
DRAP1
Group
DMSO
Q7
4.70
4.65
4.60
Samples
T1
T2
T3
Time

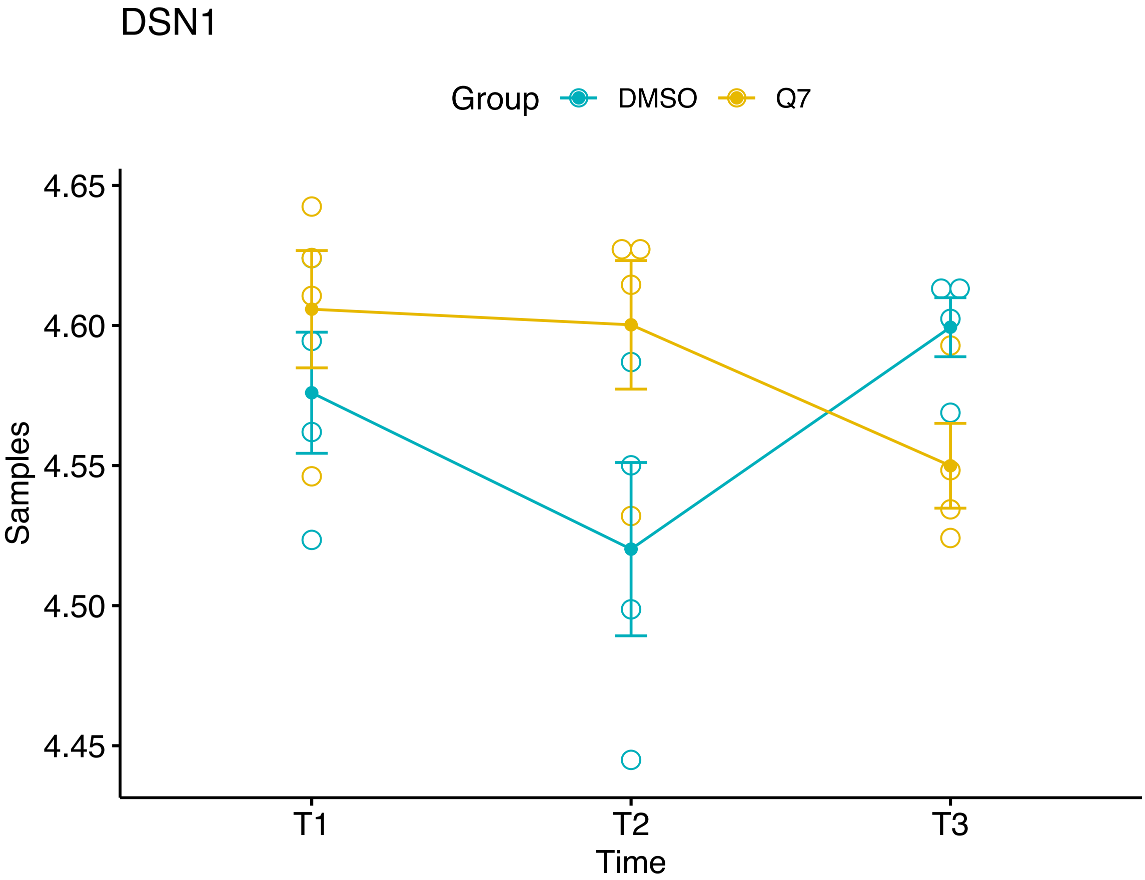
DSN1
Group
DMSO
Q7
4.65
4.60
4.55
4.50
4.45
Samples
T1
T2
T3
Time

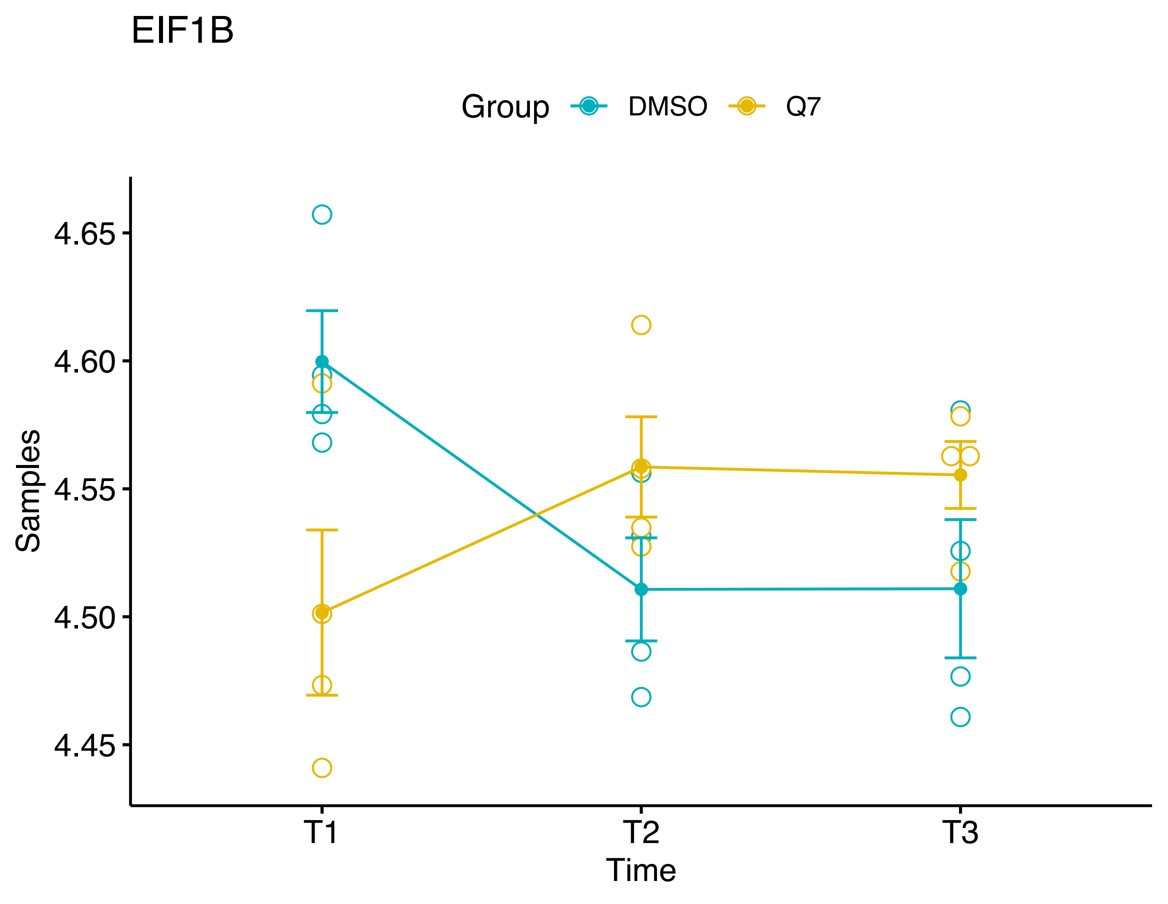

EIF1B
Group
DMSO
Q7
Samples
4.65
4.60
4.55
4.50
4.45
T1
T2
T3
Time

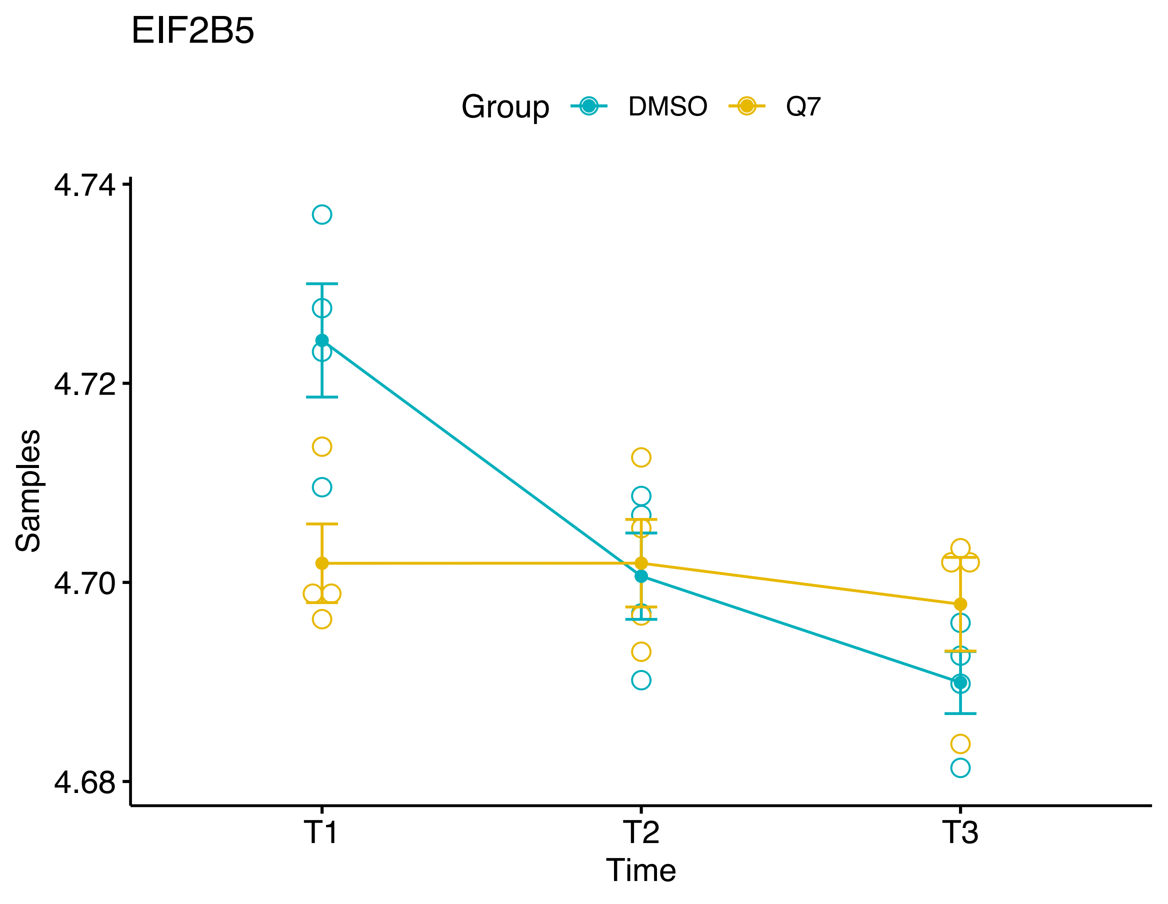

EIF2B5
Group
DMSO
Q7
4.74
4.72
4.70
4.68
Samples
T1
T2
T3
Time

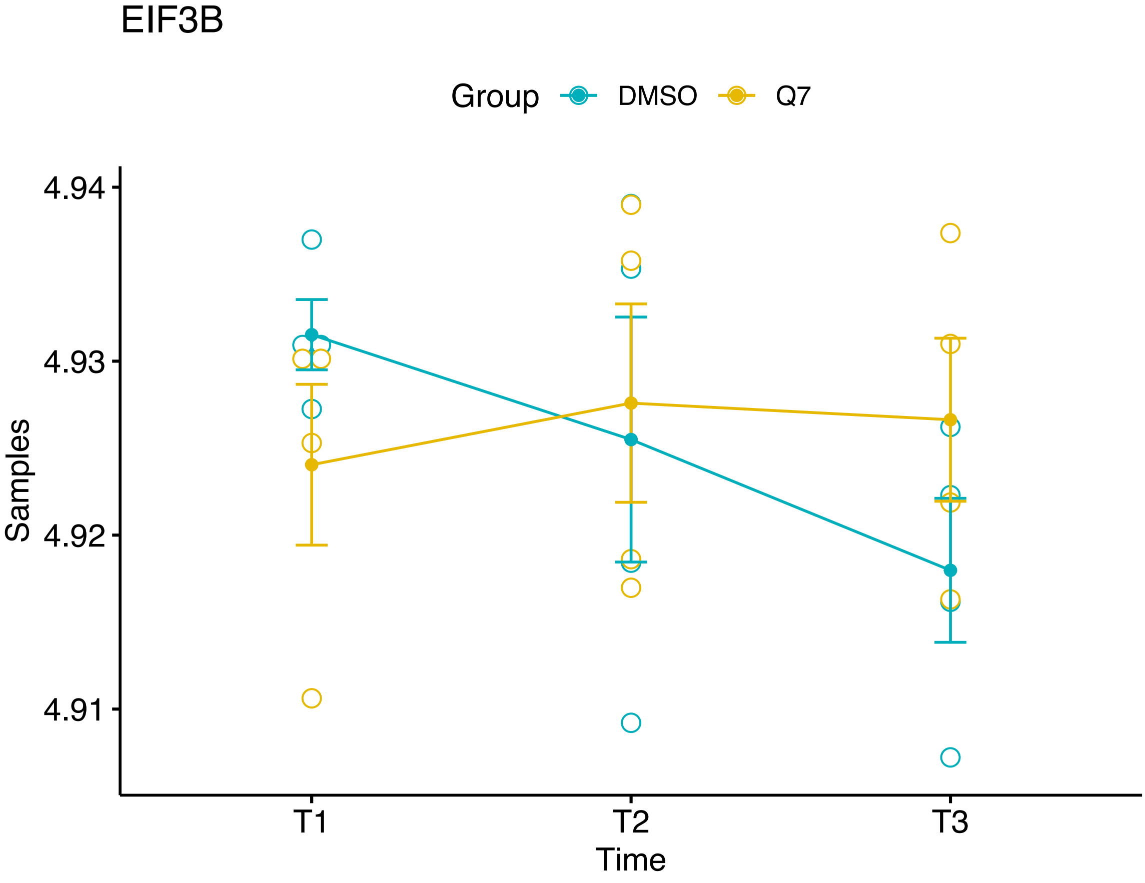
EIF3B
Group
DMSO
Q7
4.94
4.93
4.92
4.91
Samples
T1
T2
T3
Time

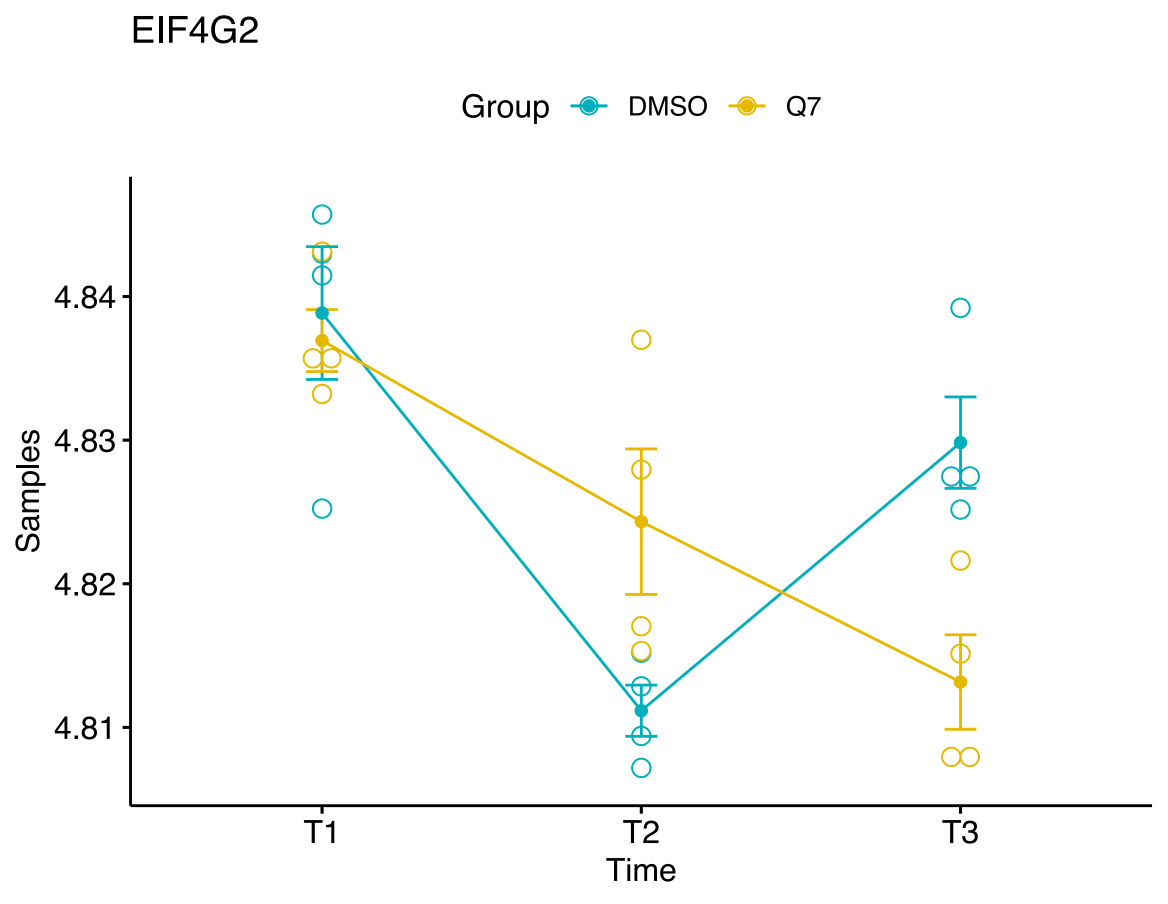
EIF4G2
Group
DMSO
Q7
4.84
4.83
4.82
4.81
Samples
T1
T2
T3
Time

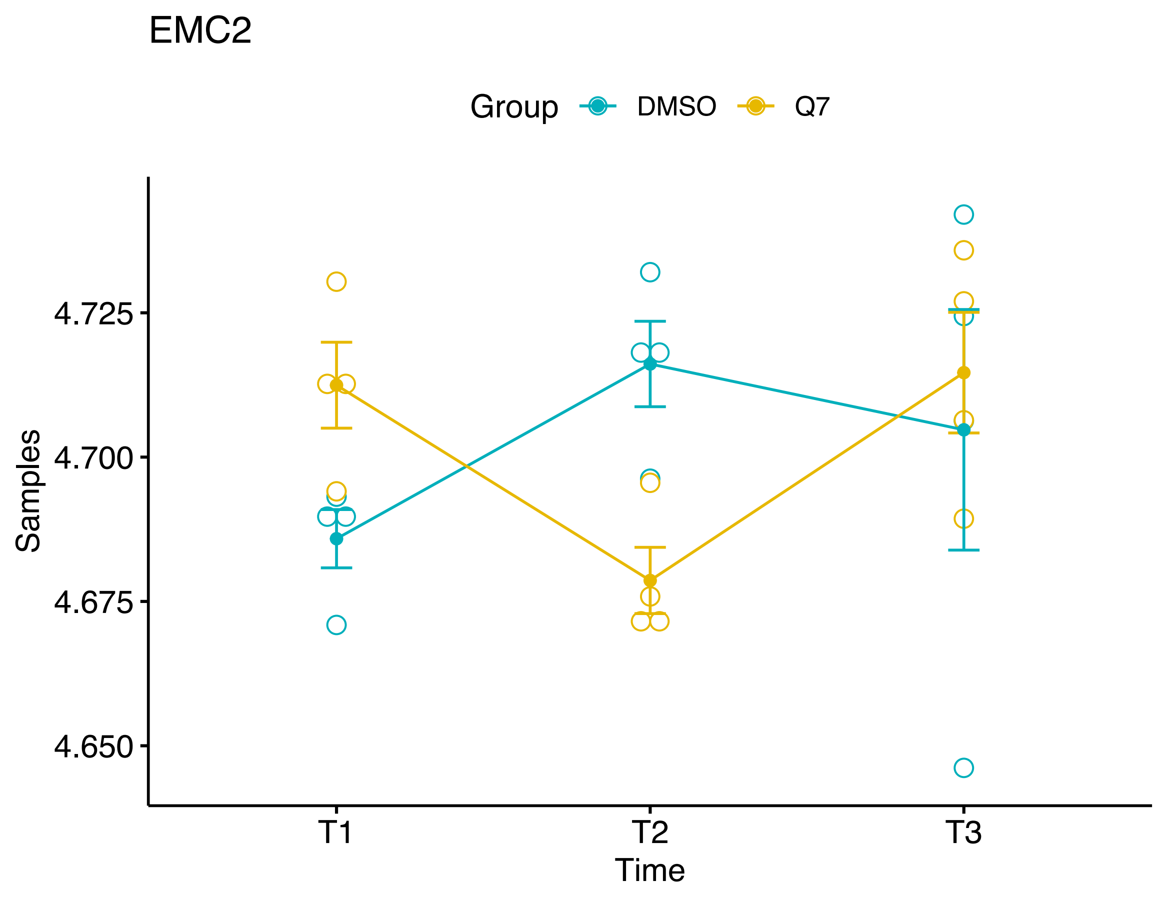
EMC2
Group
DMSO
Q7
4.725
4.700
4.675
4.650
Samples
T1
T2
T3
Time

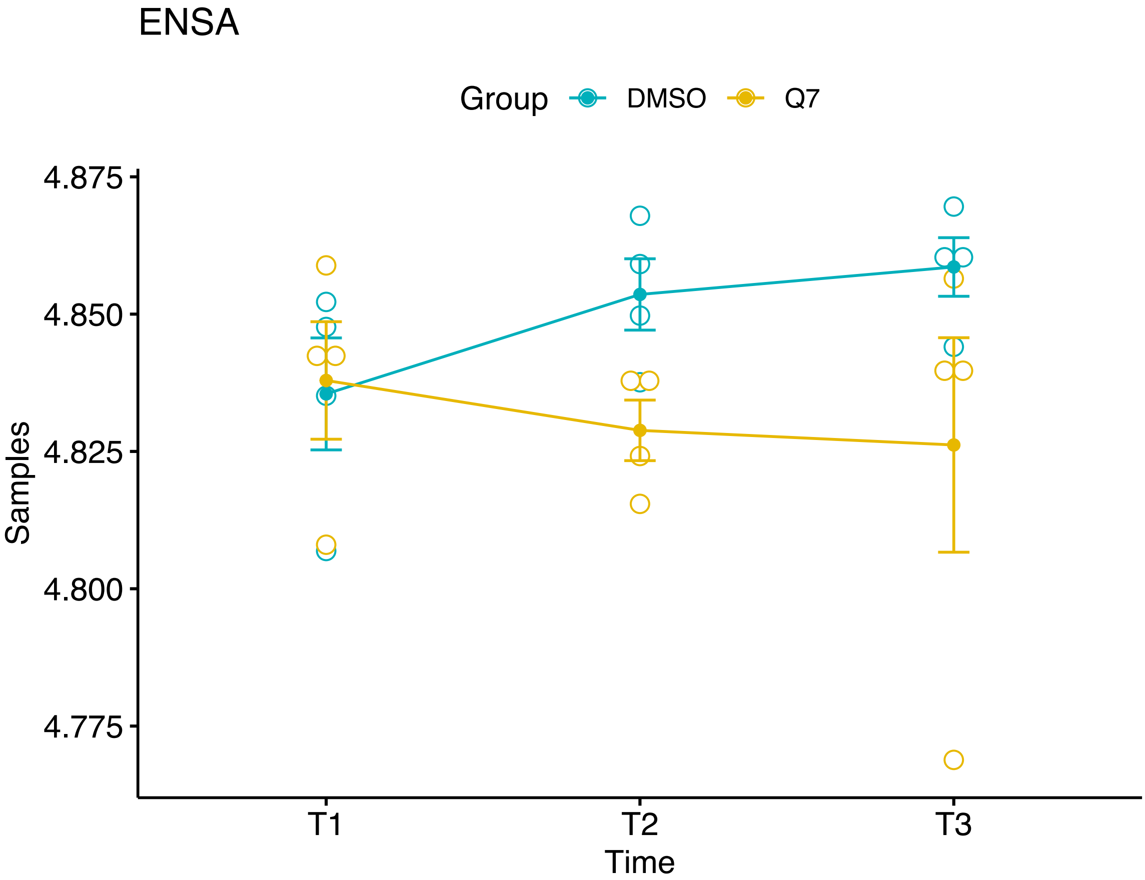

ENSA
Group
DMSO
Q7
4.875
4.850
4.825
4.800
4.775
Samples
T1
T2
T3
Time

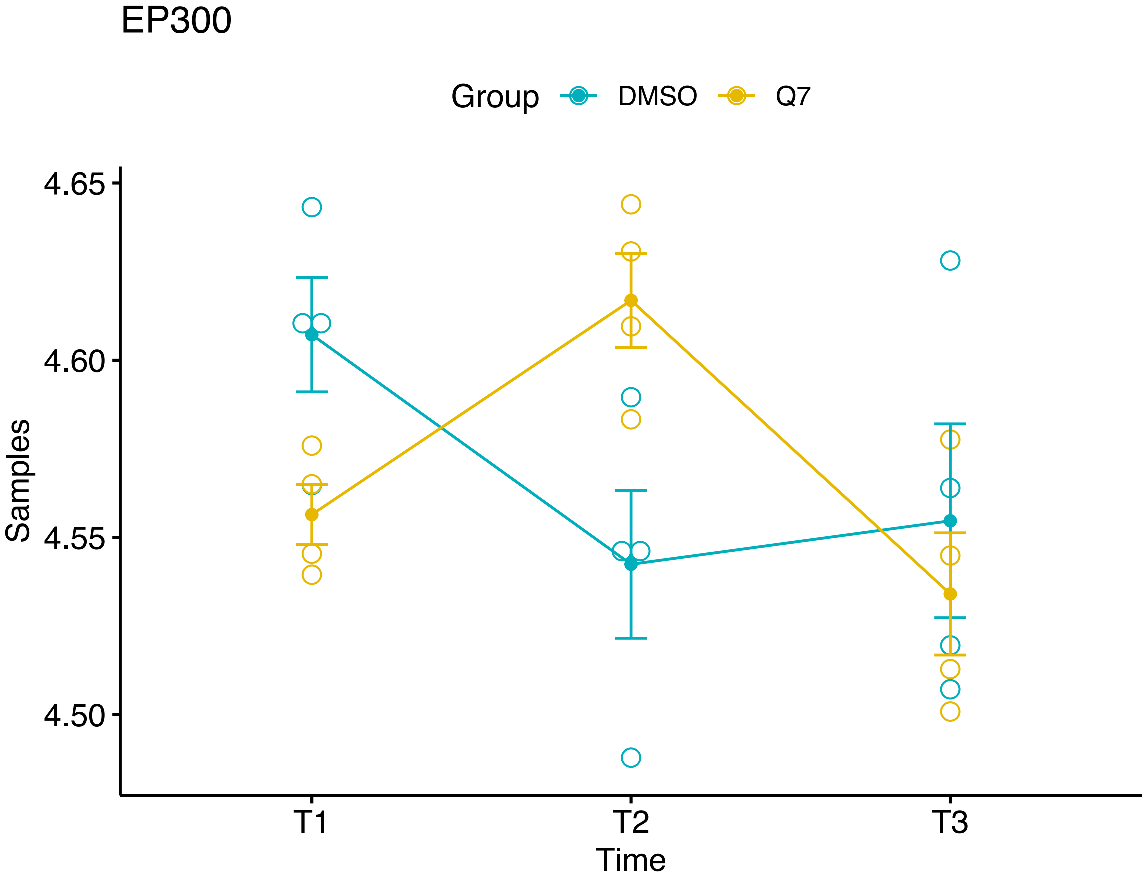
EP300
Group
DMSO
Q7
4.65
4.60
4.55
4.50
Samples
T1
T2
T3
Time

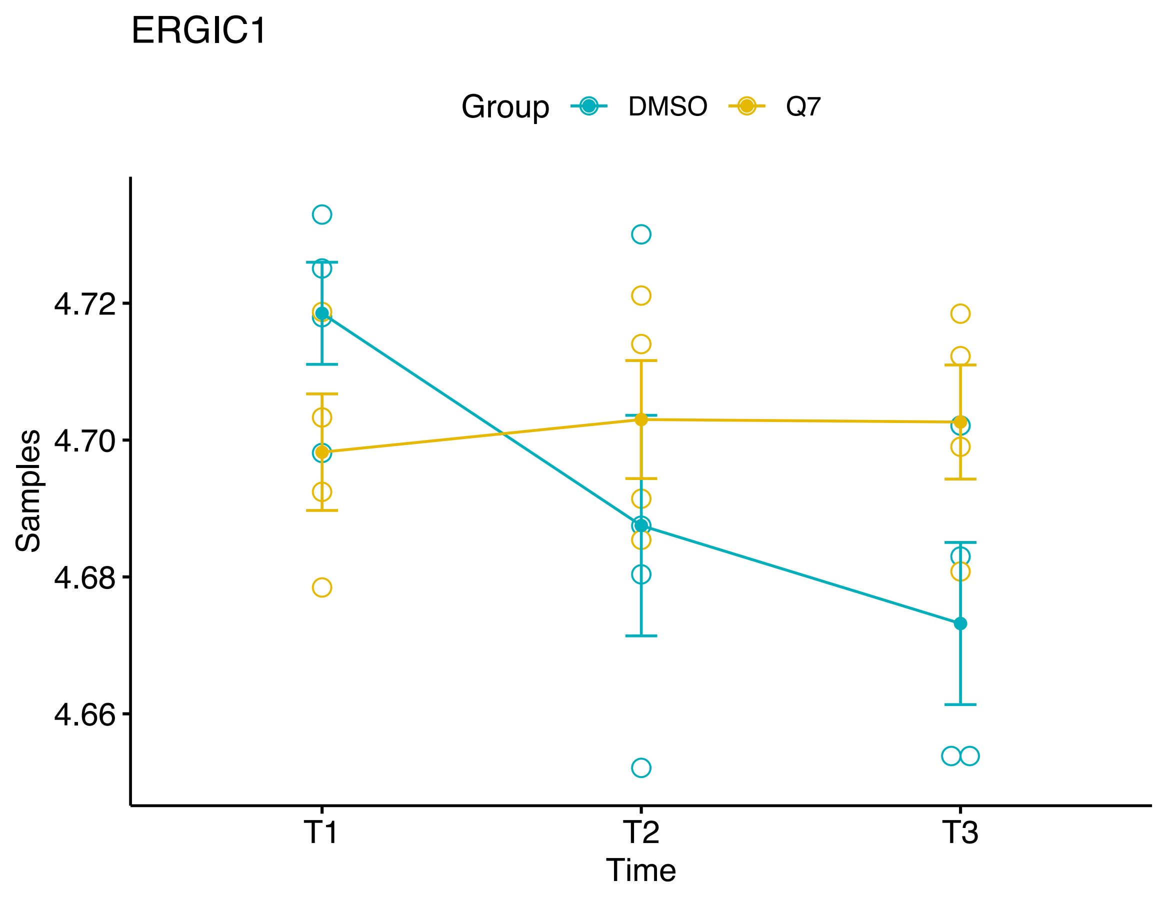

ERGIC1
Group
DMSO
Q7
Samples
4.72
4.70
4.68
4.66
T1
T2
T3
Time

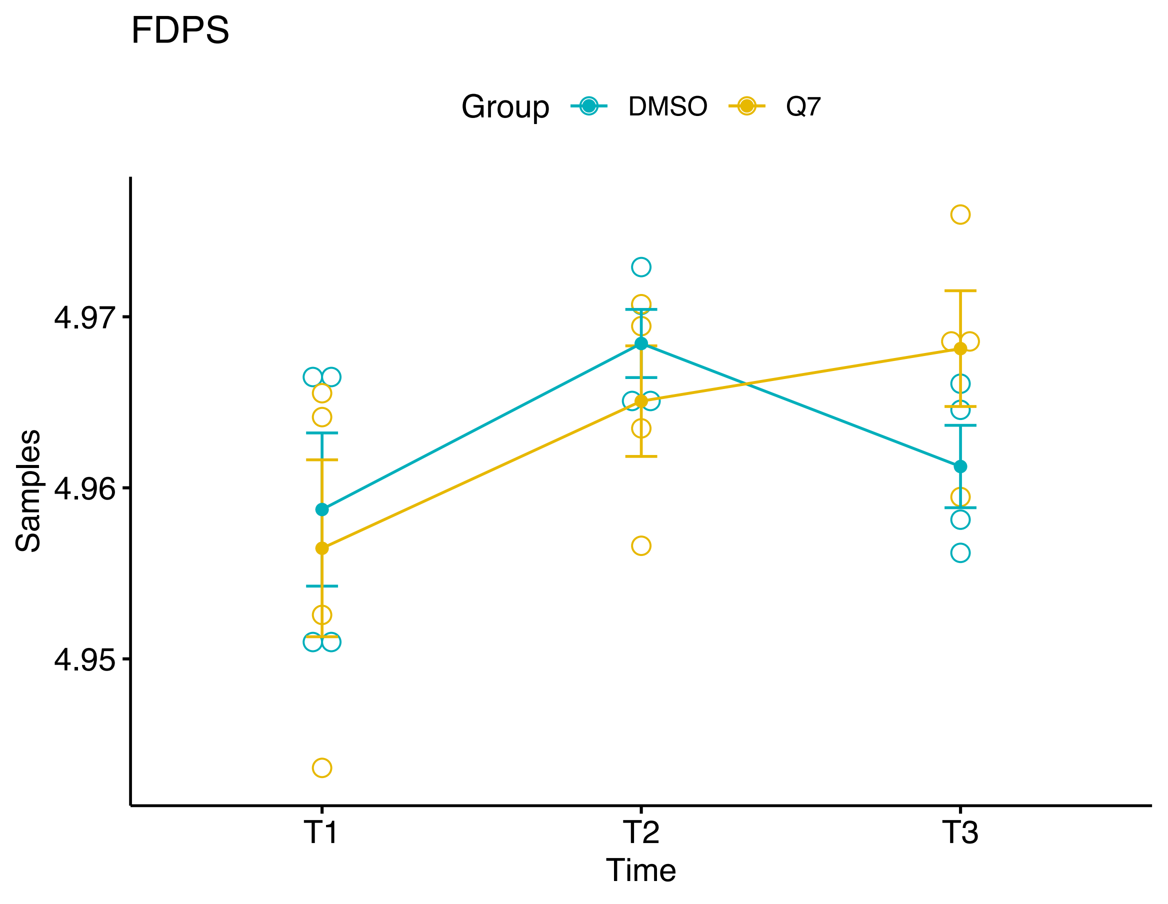
FDPS
Group
DMSO
Q7
Samples
4.97
4.96
4.95
T1
T2
T3
Time

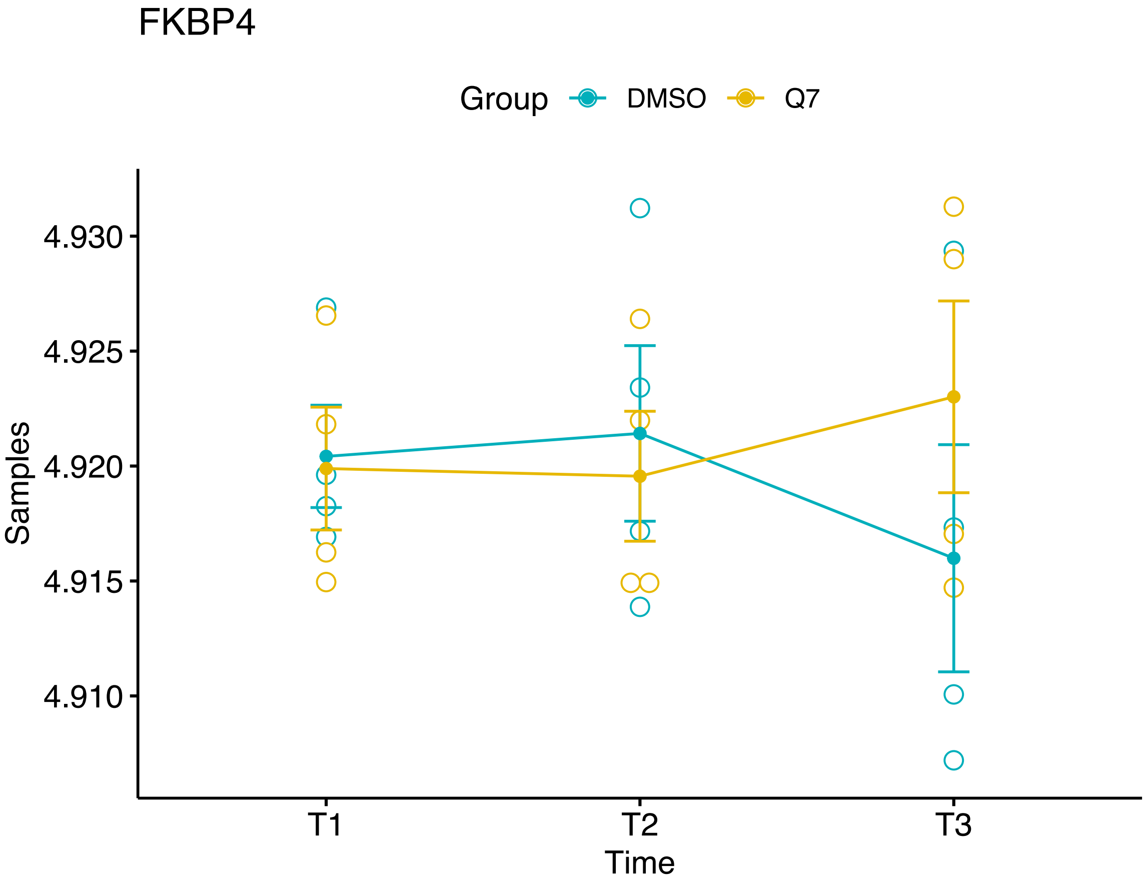
FKBP4
Group
DMSO
Q7
Samples
4.930
4.925
4.920
4.915
4.910
T1
T2
T3
Time

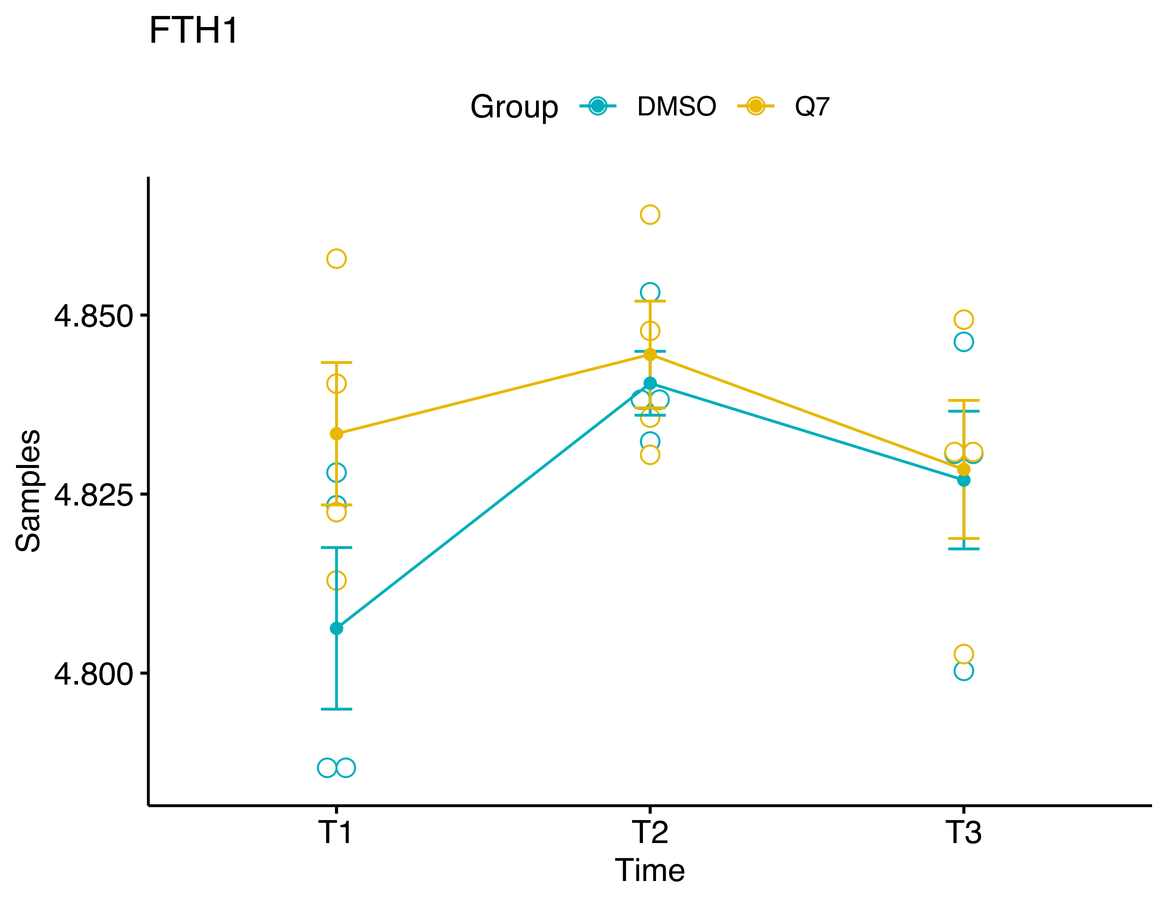

FTH1
Group
DMSO
Q7
Samples
4.850
4.825
4.800
T1
T2
T3
Time

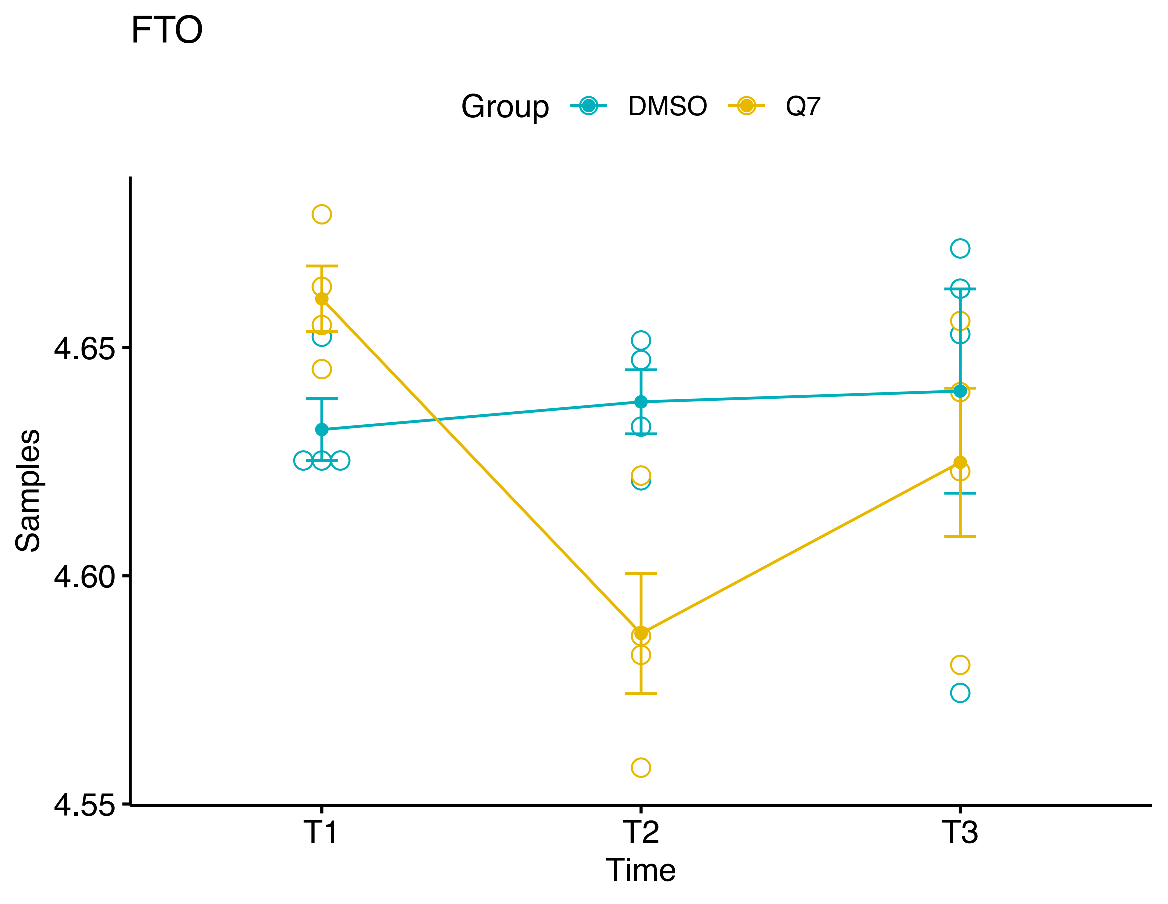

FTO
Group
DMSO
Q7
4.65
4.60
4.55
Samples
T1
T2
T3
Time

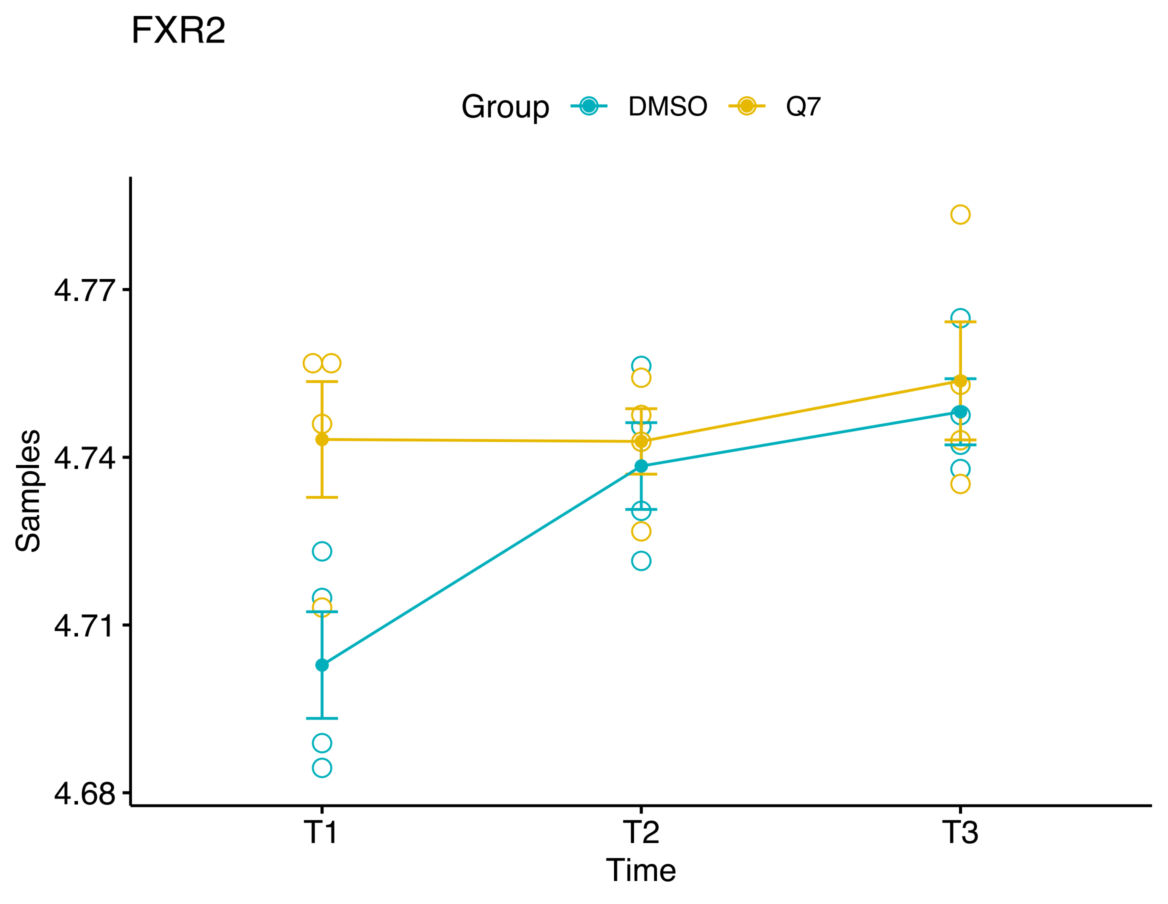

FXR2
Group
DMSO
Q7
4.77
4.74
4.71
4.68
Samples
T1
T2
T3
Time

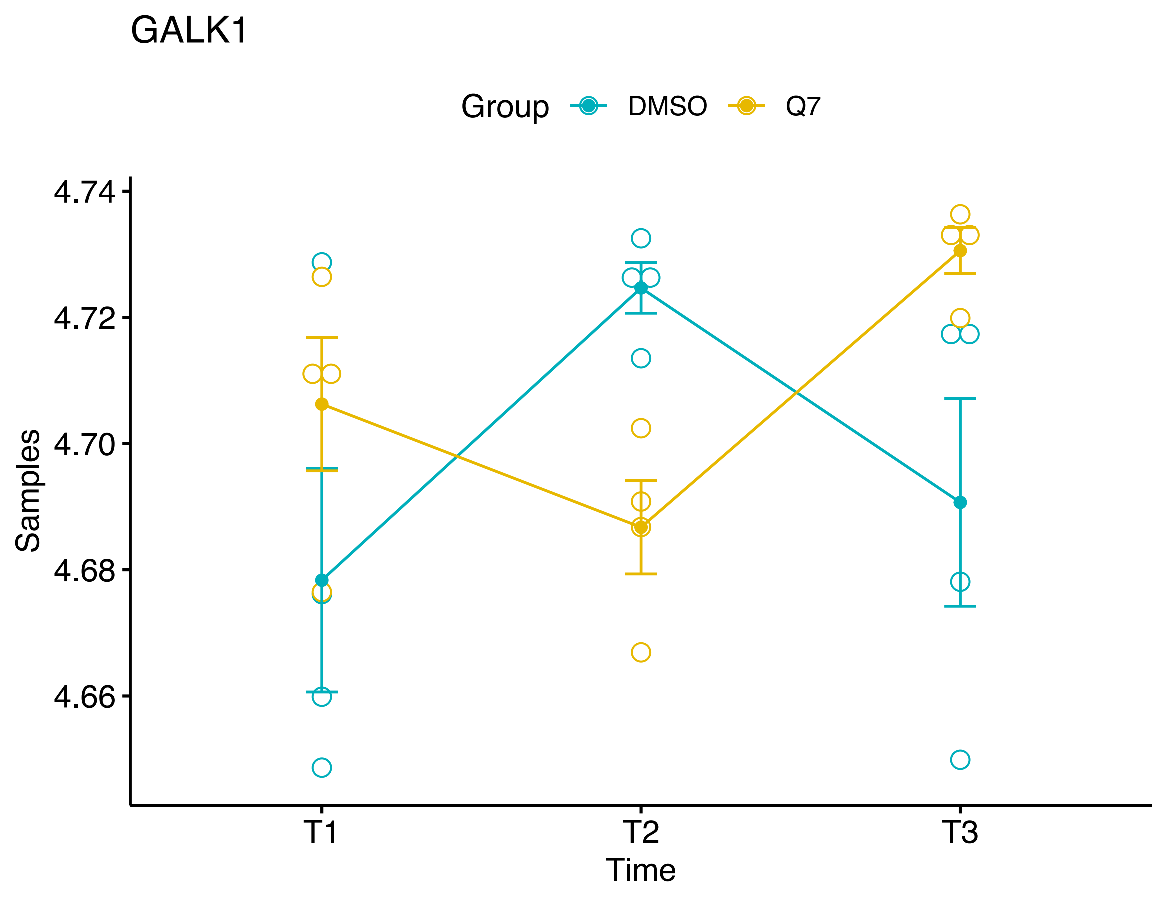
GALK1
Group
DMSO
Q7
4.74
4.72
4.70
4.68
4.66
Samples
T1
T2
T3
Time

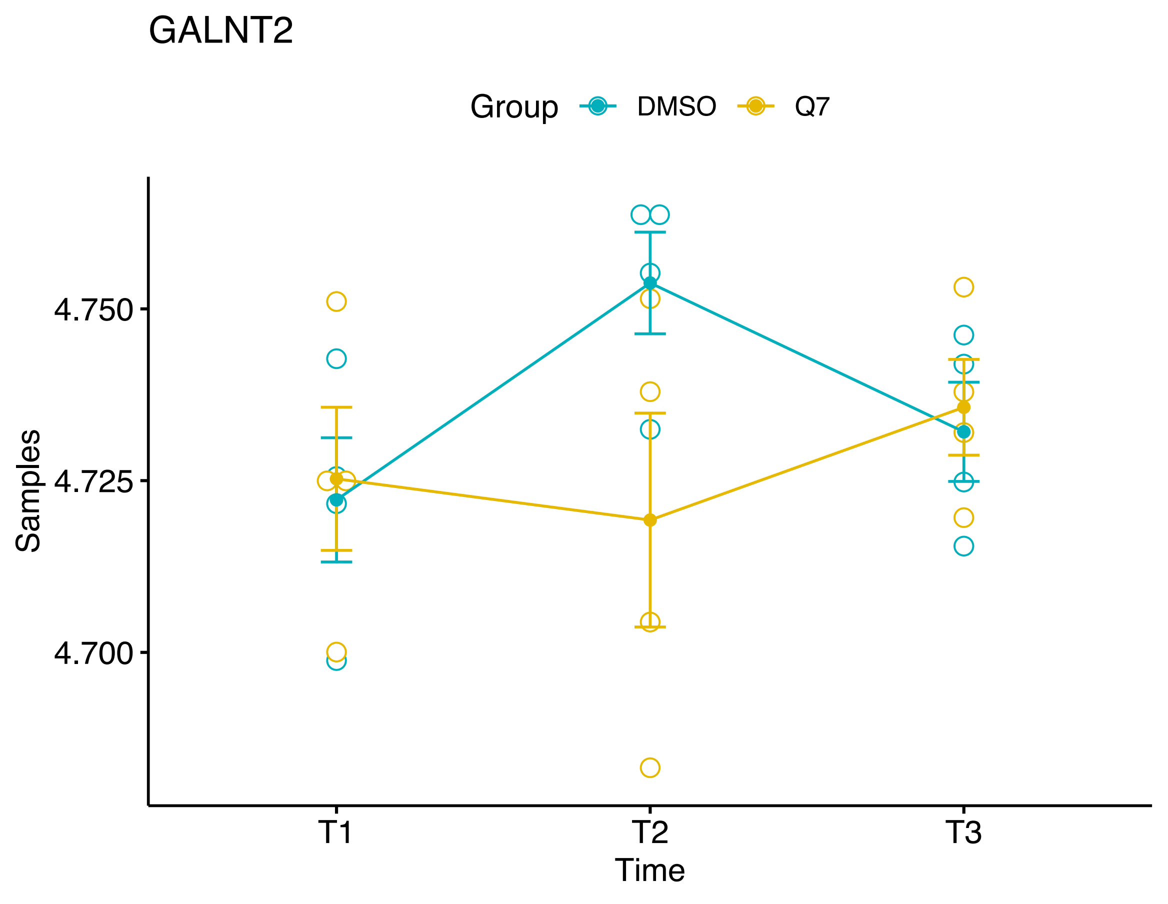

GALNT2
Group
DMSO
Q7
Samples
4.750
4.725
4.700
T1
T2
T3
Time

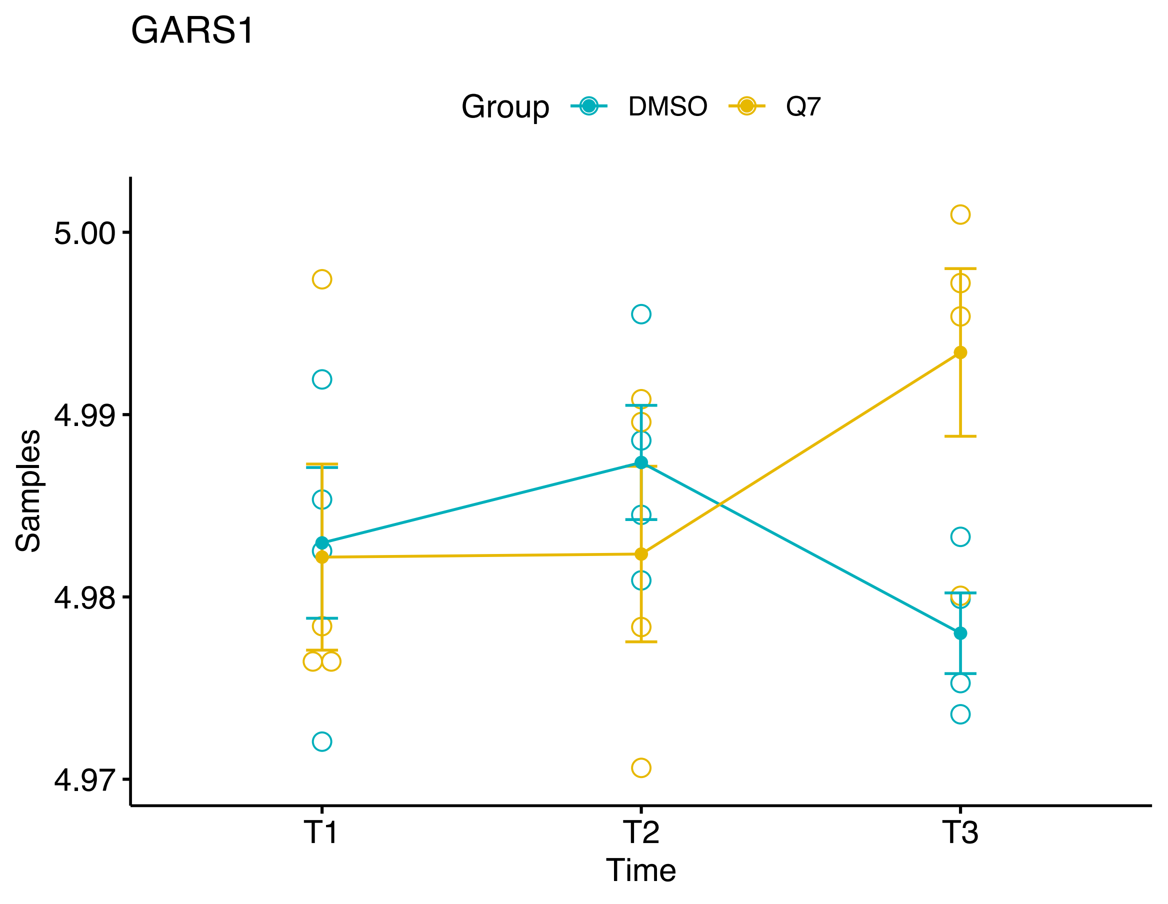
GARS1
Group
DMSO
Q7
Samples
5.00
4.99
4.98
4.97
T1
T2
T3
Time

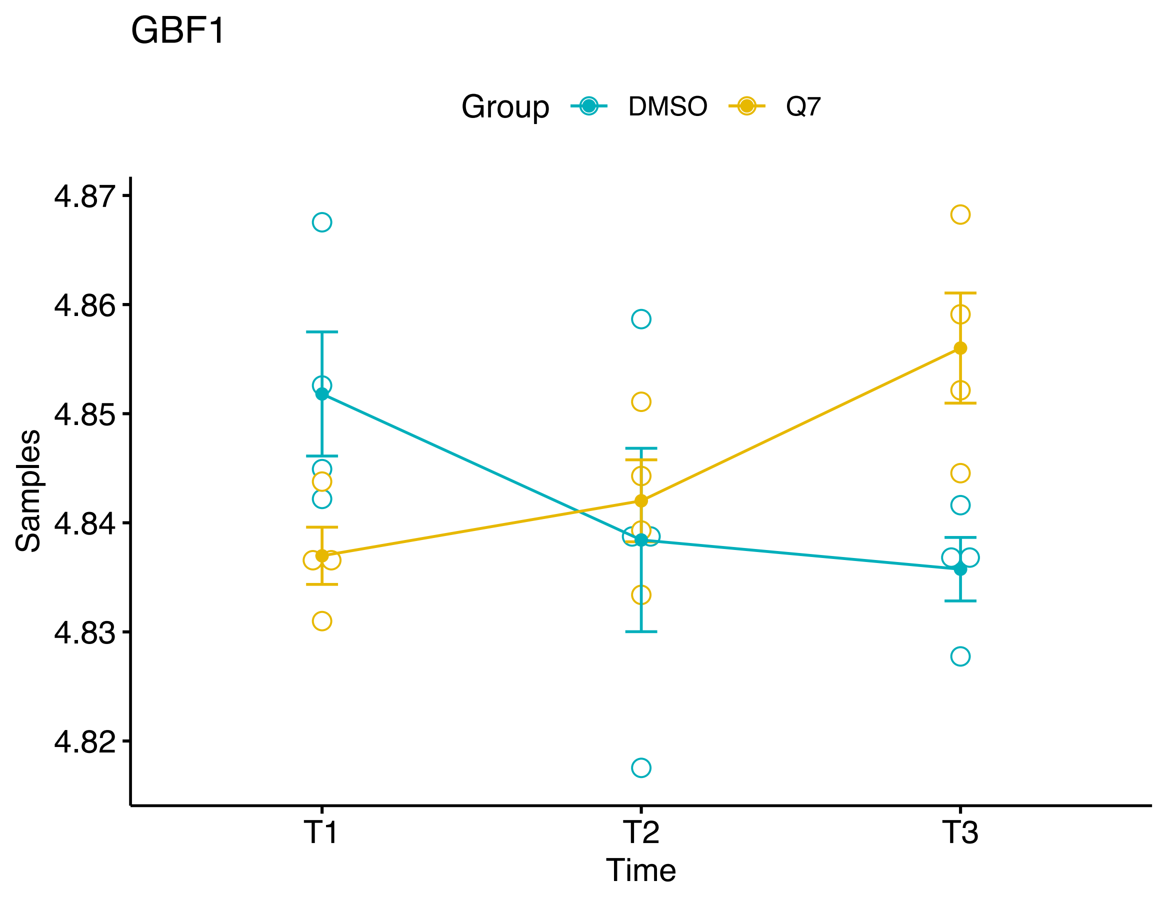
GBF1
Group
DMSO
Q7
4.87
4.86
4.85
4.84
4.83
4.82
Samples
T1
T2
T3
Time

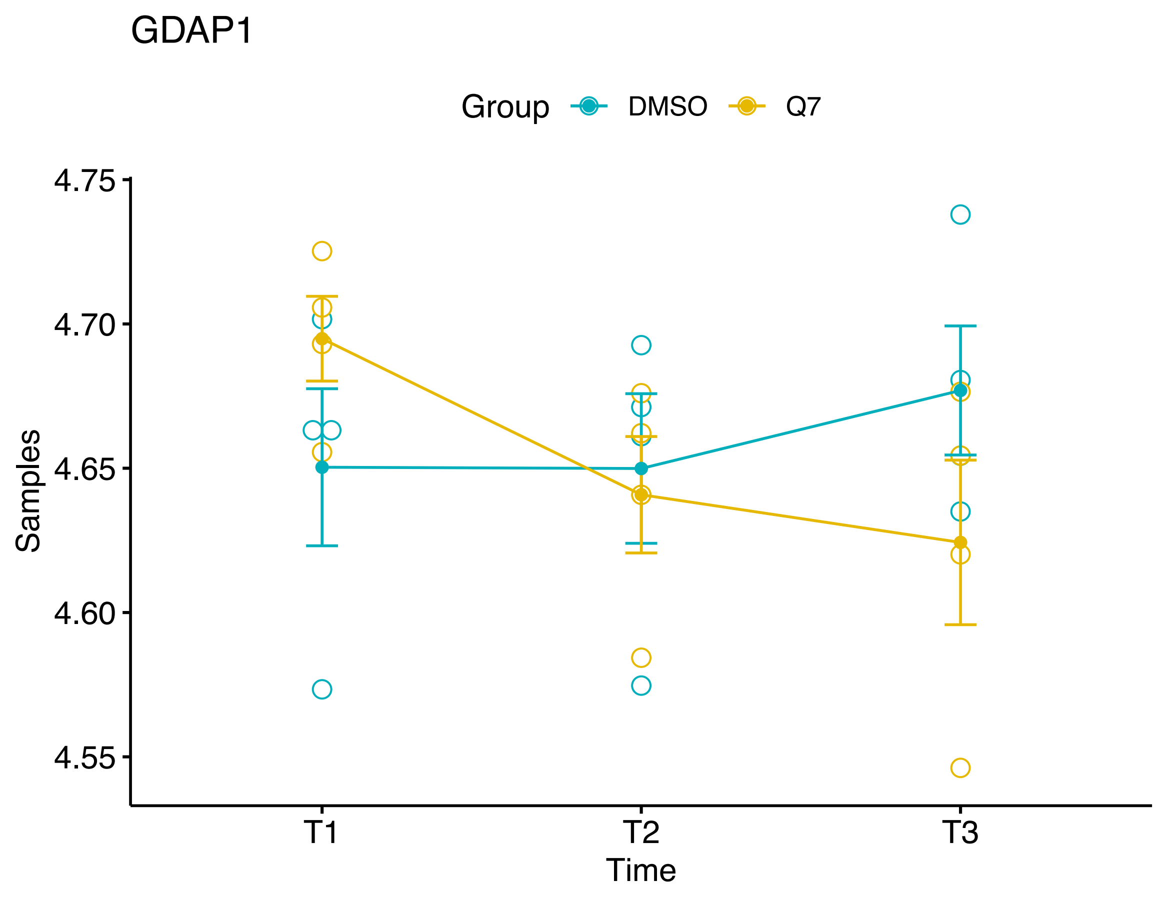

GDAP1
Group
DMSO
Q7
4.75
4.70
4.65
4.60
4.55
Samples
T1
T2
T3
Time

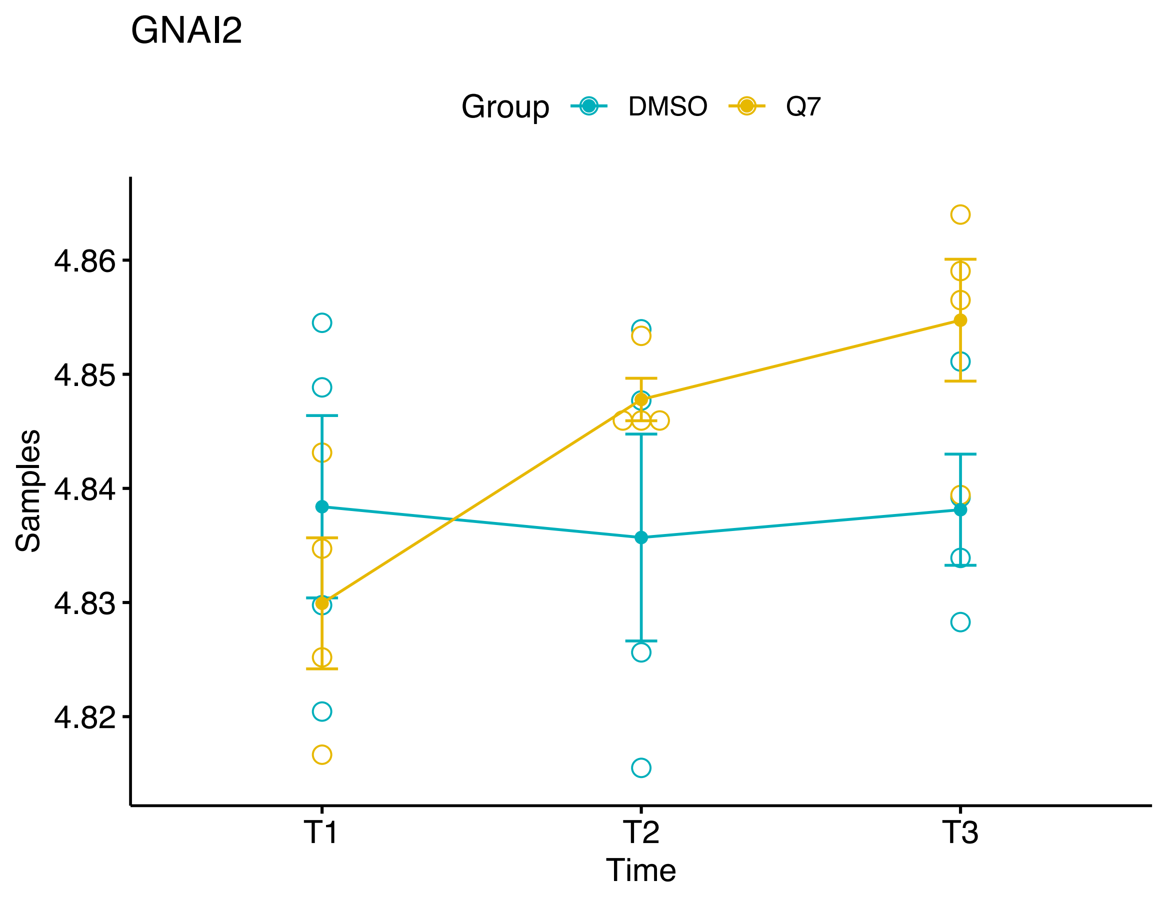
GNAI2
Group
DMSO
Q7
Samples
4.86
4.85
4.84
4.83
4.82
T1
T2
T3
Time

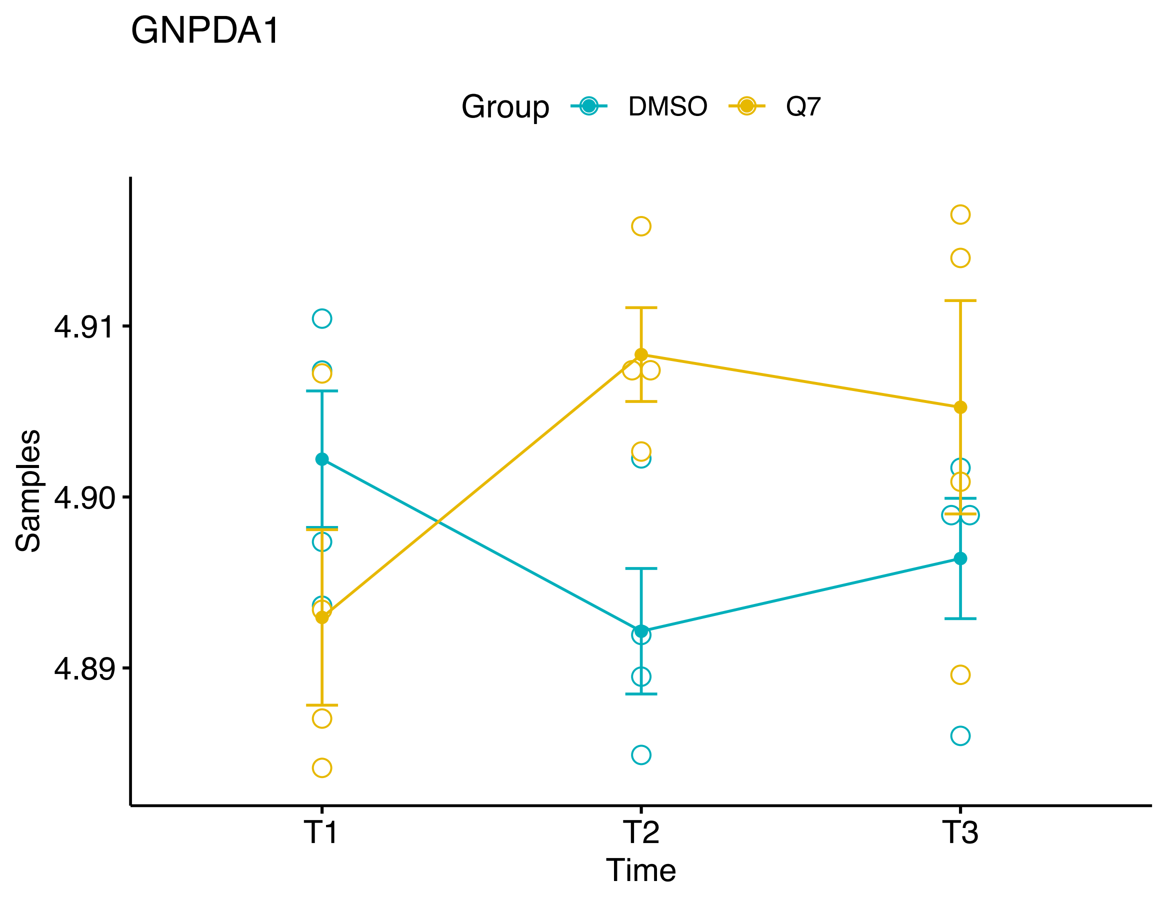

GNPDA1
Group
DMSO
Q7
Samples
4.91
4.90
4.89
T1
T2
T3
Time

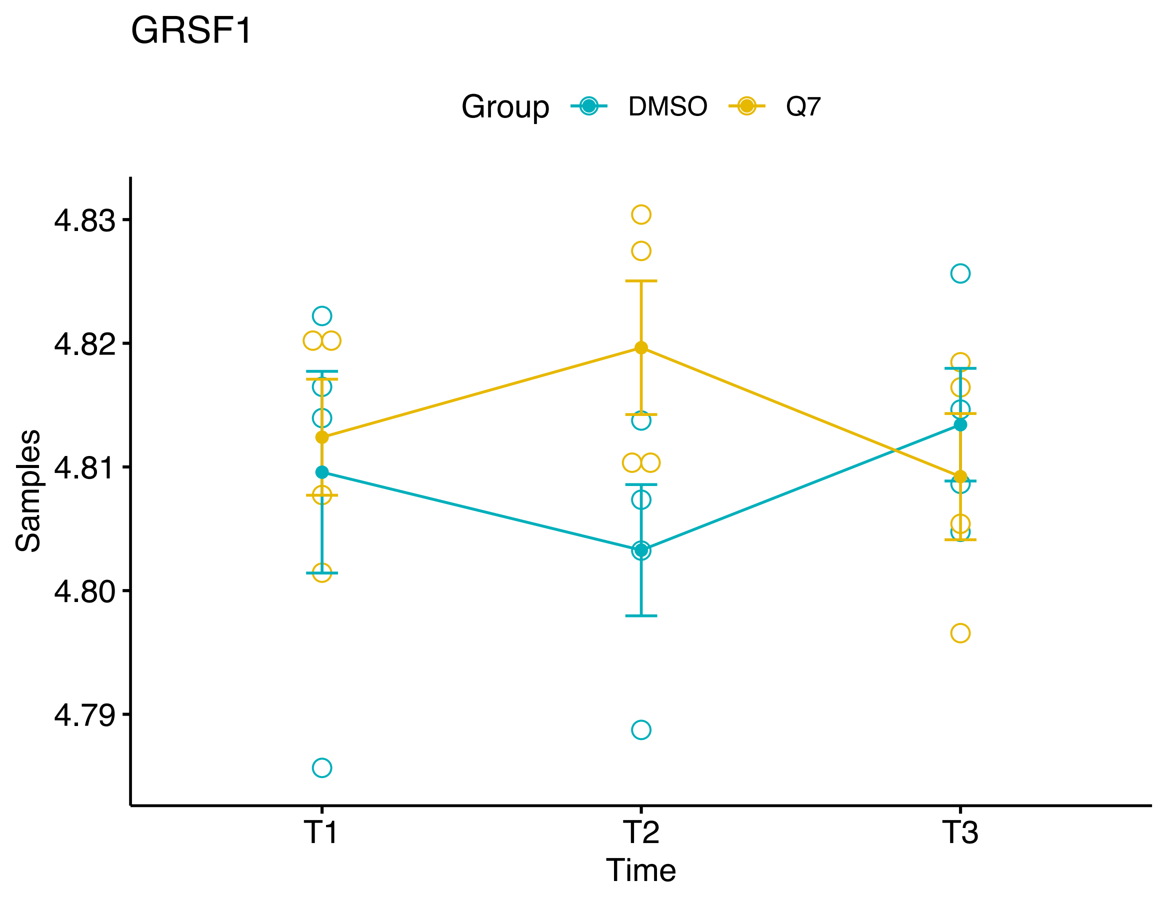

GRSF1
Group
DMSO
Q7
4.83
4.82
4.81
4.80
4.79
Samples
T1
T2
T3
Time

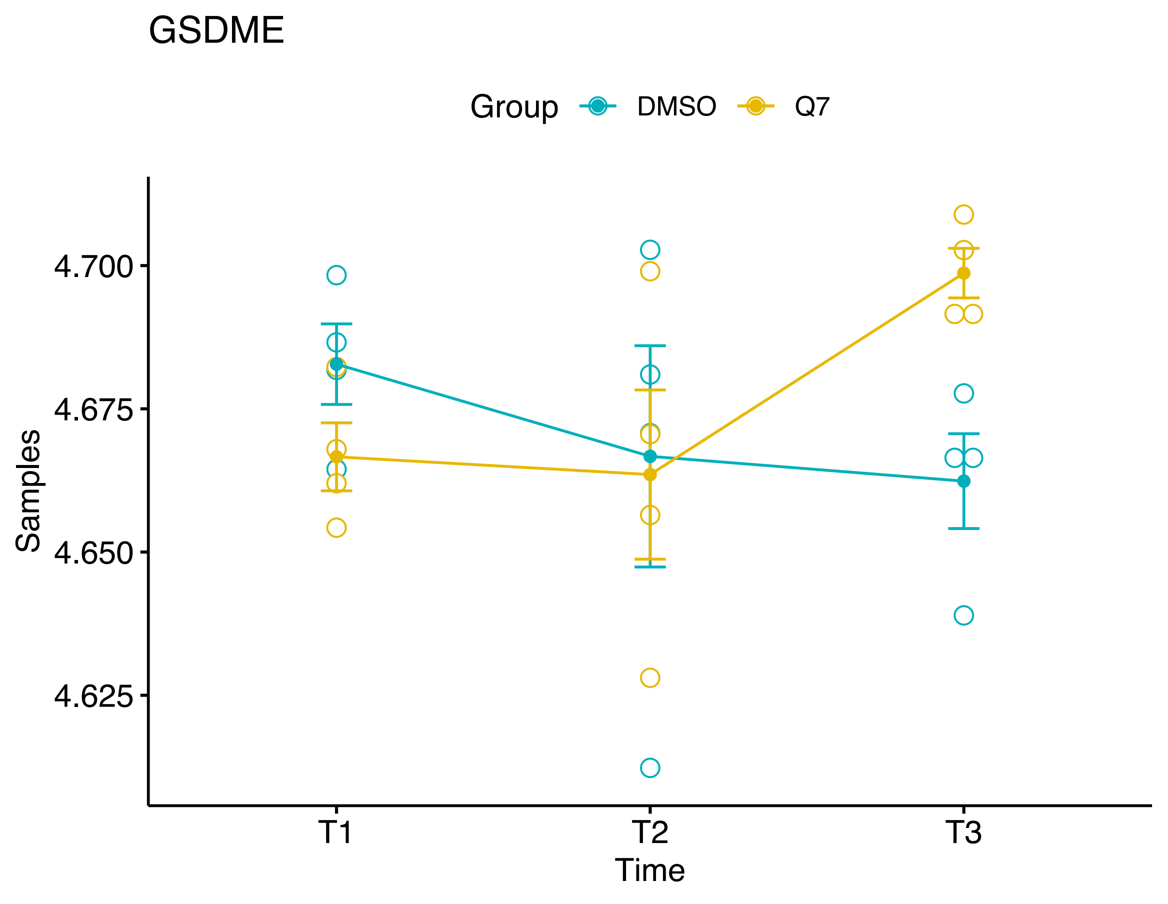

GSDME
Group
DMSO
Q7
4.700
4.675
4.650
4.625
Samples
T1
T2
T3
Time

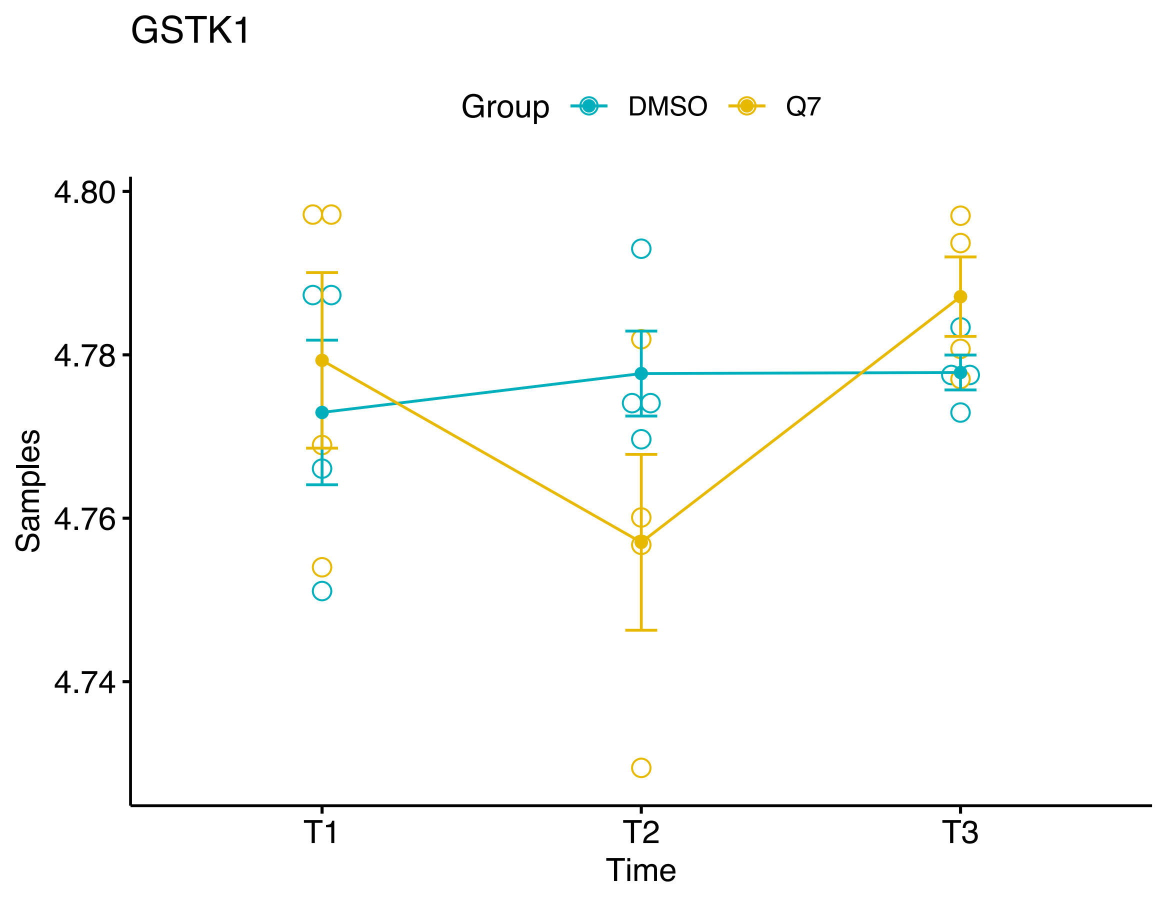
GSTK1
Group
DMSO
Q7
4.80
4.78
4.76
4.74
Samples
T1
T2
T3
Time

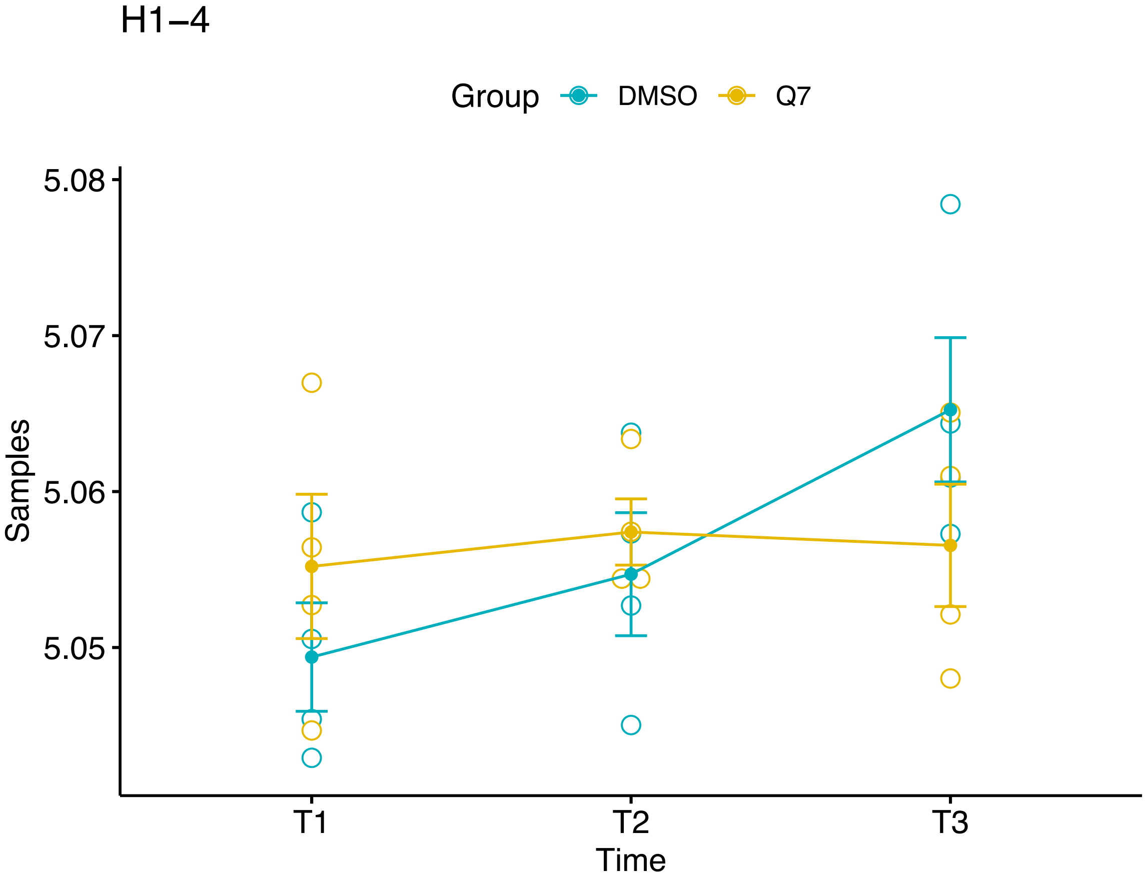
H1−4
Group
DMSO
Q7
5.08
5.07
5.06
5.05
Samples
T1
T2
T3
Time

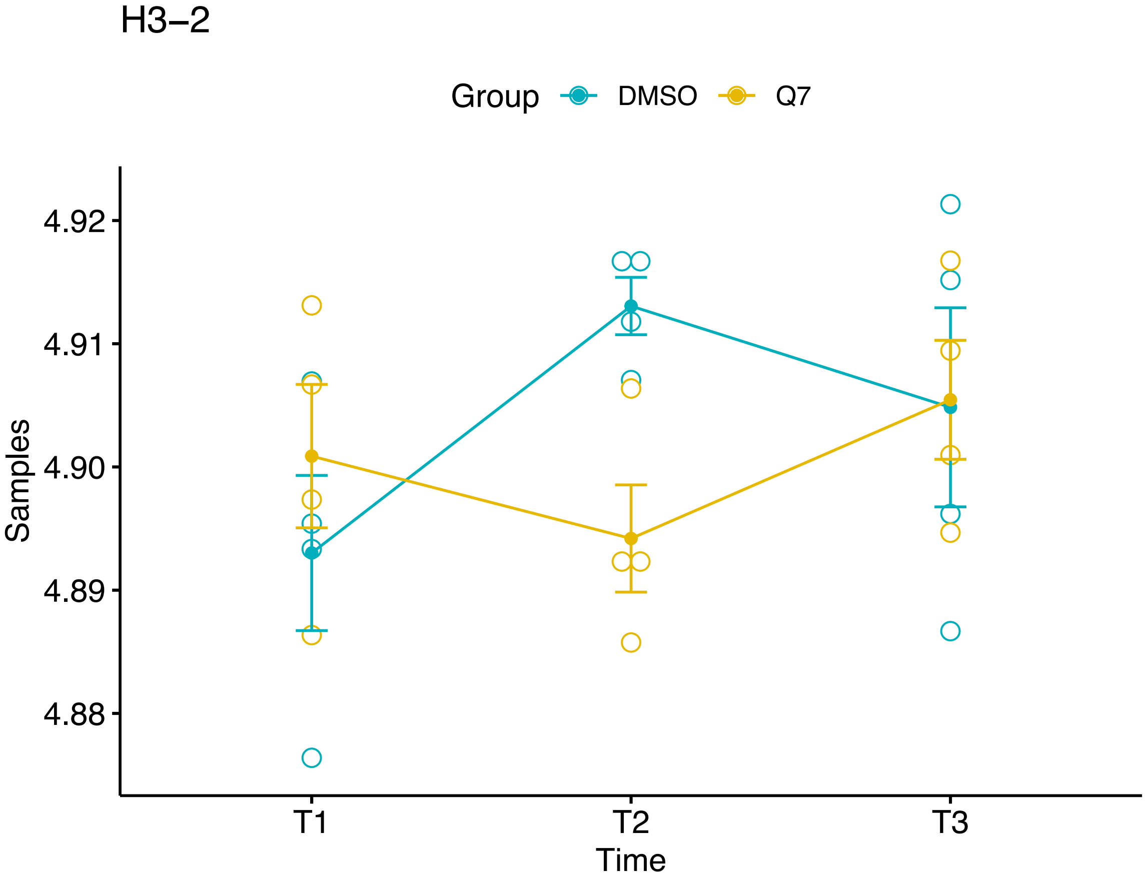

H3–2
Group
DMSO
Q7
4.92
4.91
4.90
4.89
4.88
Samples
T1
T2
T3
Time

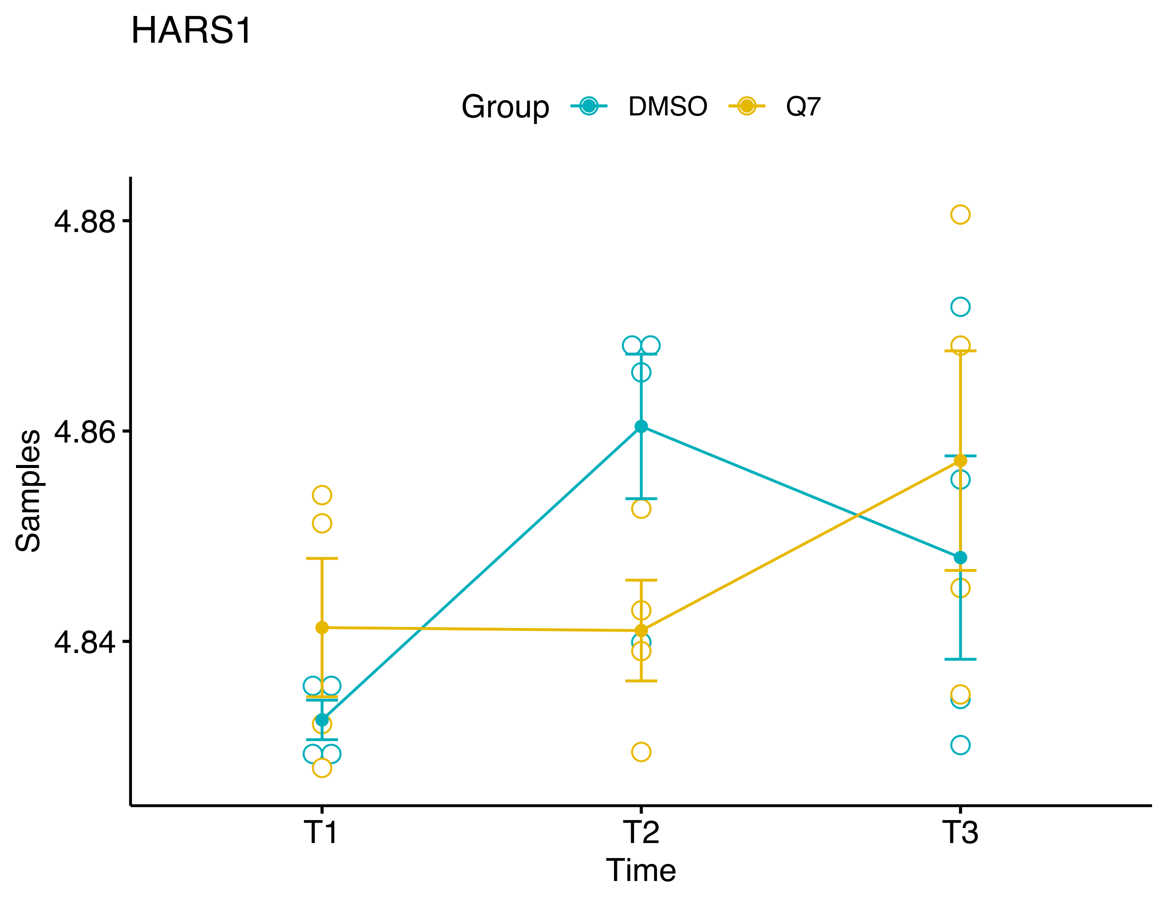
HARS1
Group
DMSO
Q7
4.88
4.86
4.84
Samples
T1
T2
T3
Time

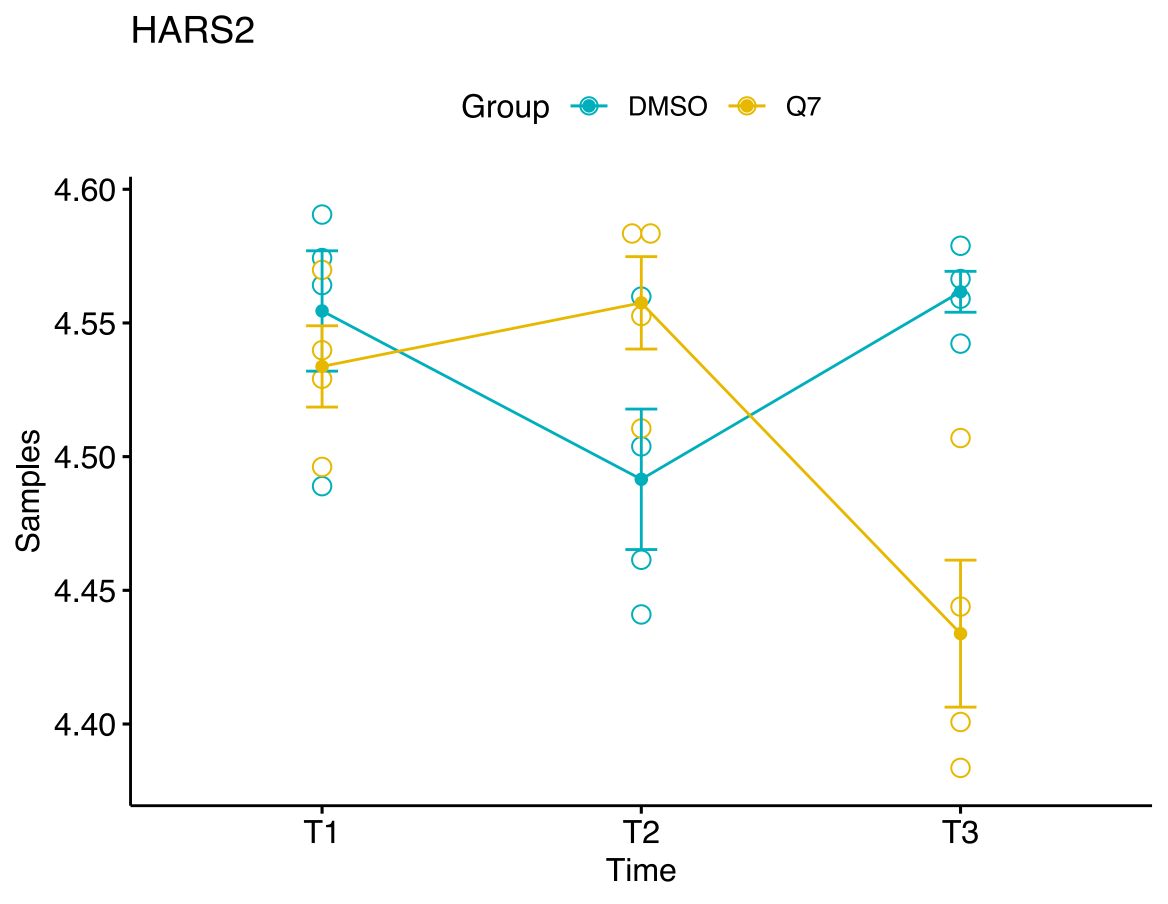
HARS2
Group
DMSO
Q7
4.60
4.55
4.50
4.45
4.40
Samples
T1
T2
T3
Time

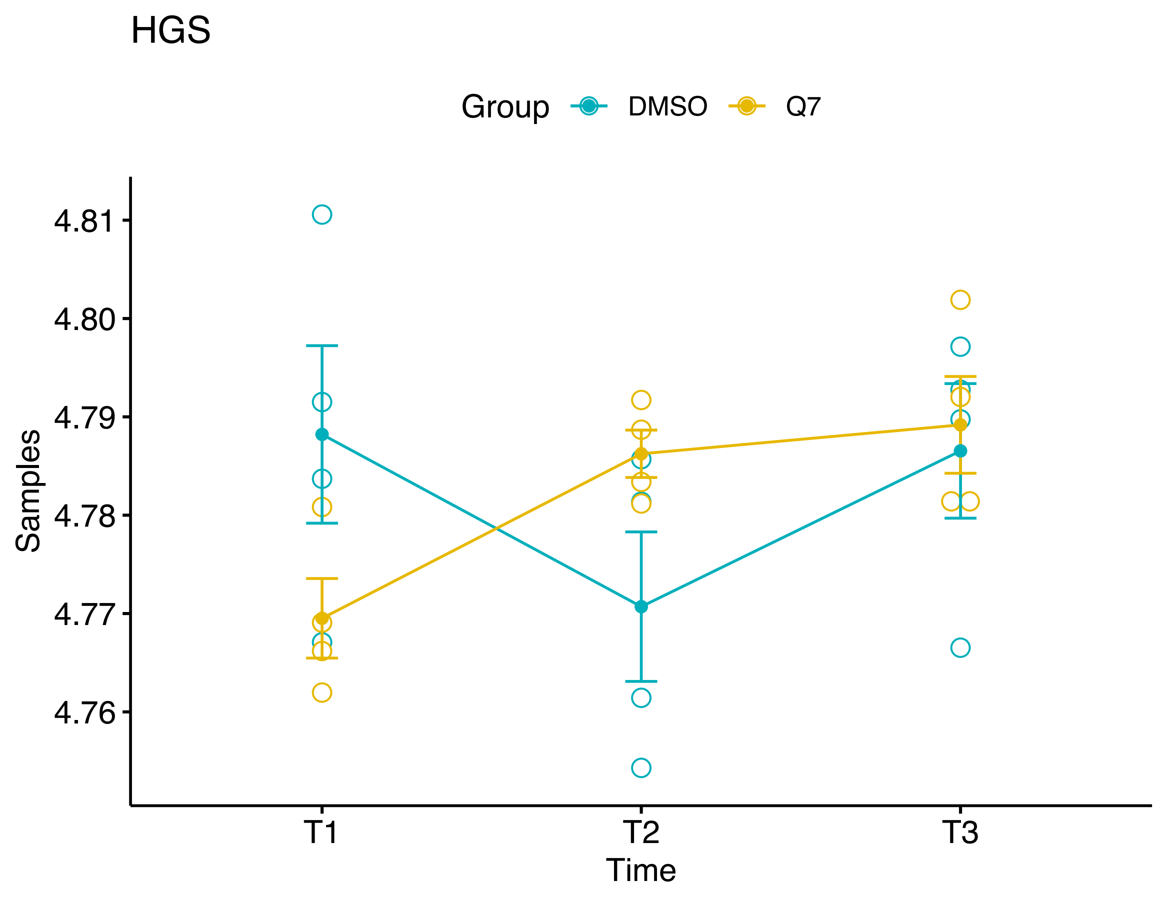
HGS
Group
DMSO
Q7
Samples
4.81
4.80
4.79
4.78
4.77
4.76
T1
T2
T3
Time

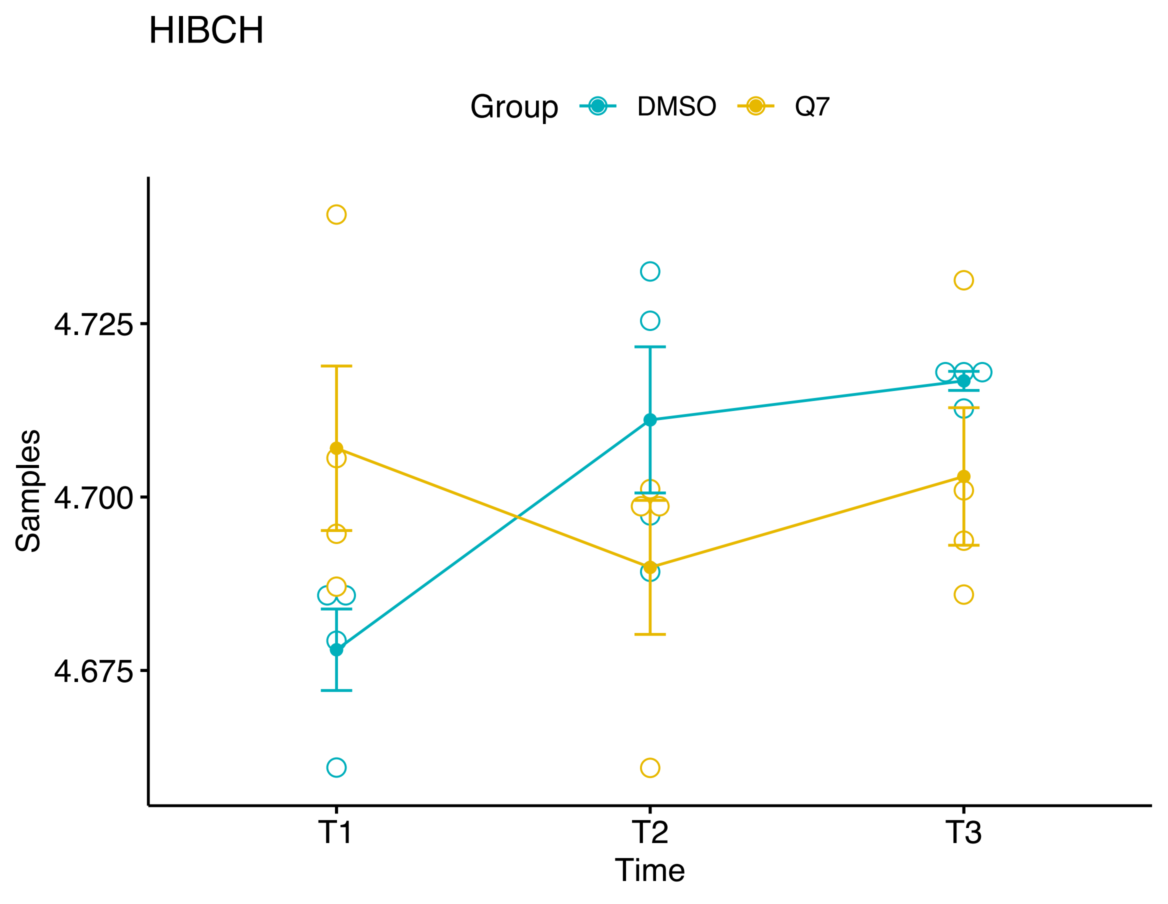

HIBCH
Group
DMSO
Q7
Samples
4.725
4.700
4.675
T1
T2
T3
Time

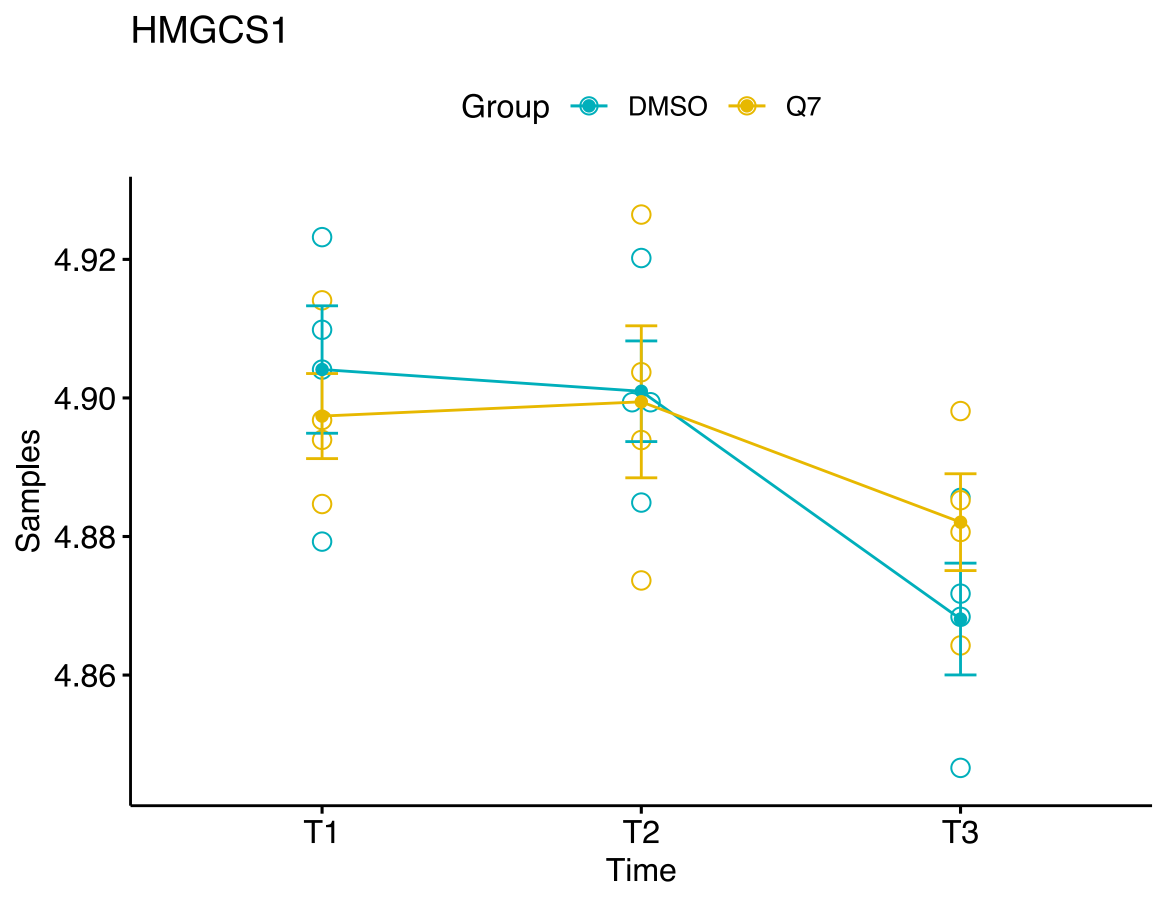
HMGCS1
Group
DMSO
Q7
4.92
4.90
4.88
4.86
Samples
T1
T2
T3
Time

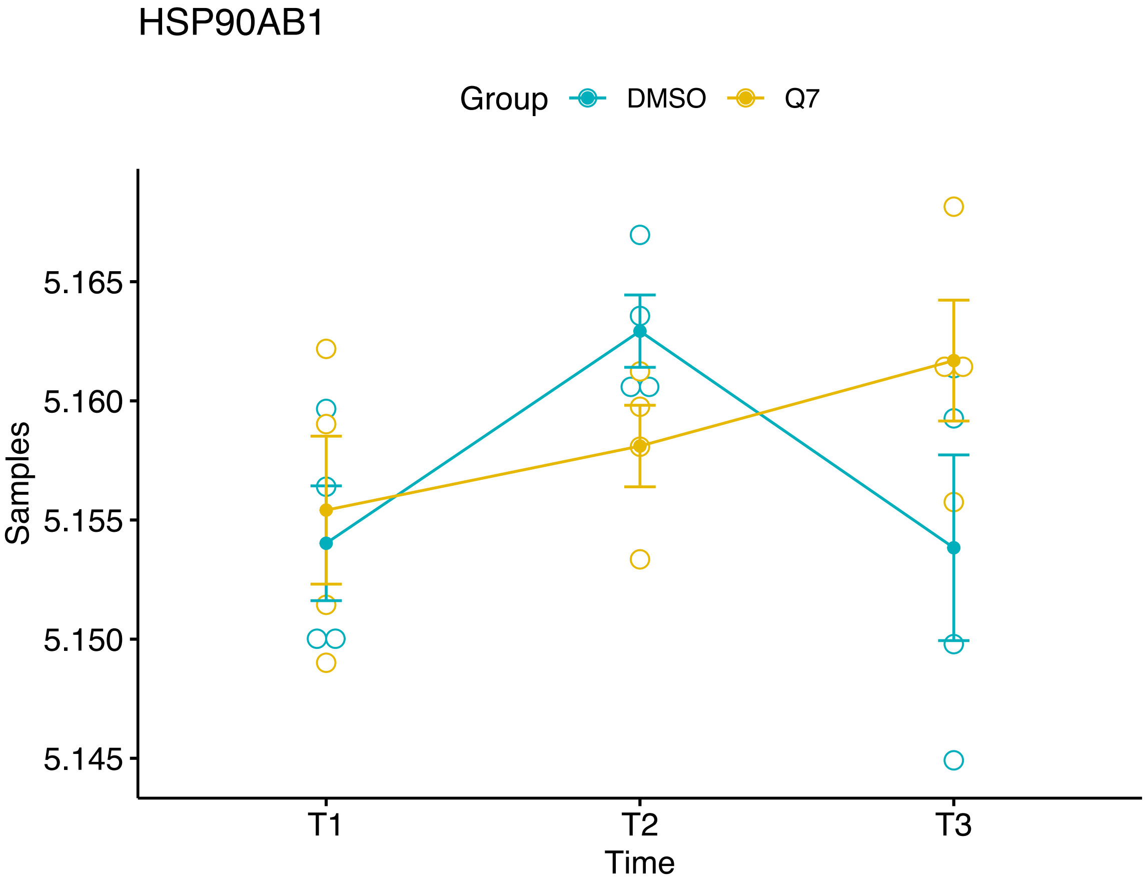

HSP90AB1
Group
DMSO
Q7
Samples
5.165
5.160
5.155
5.150
5.145
T1
T2
T3
Time

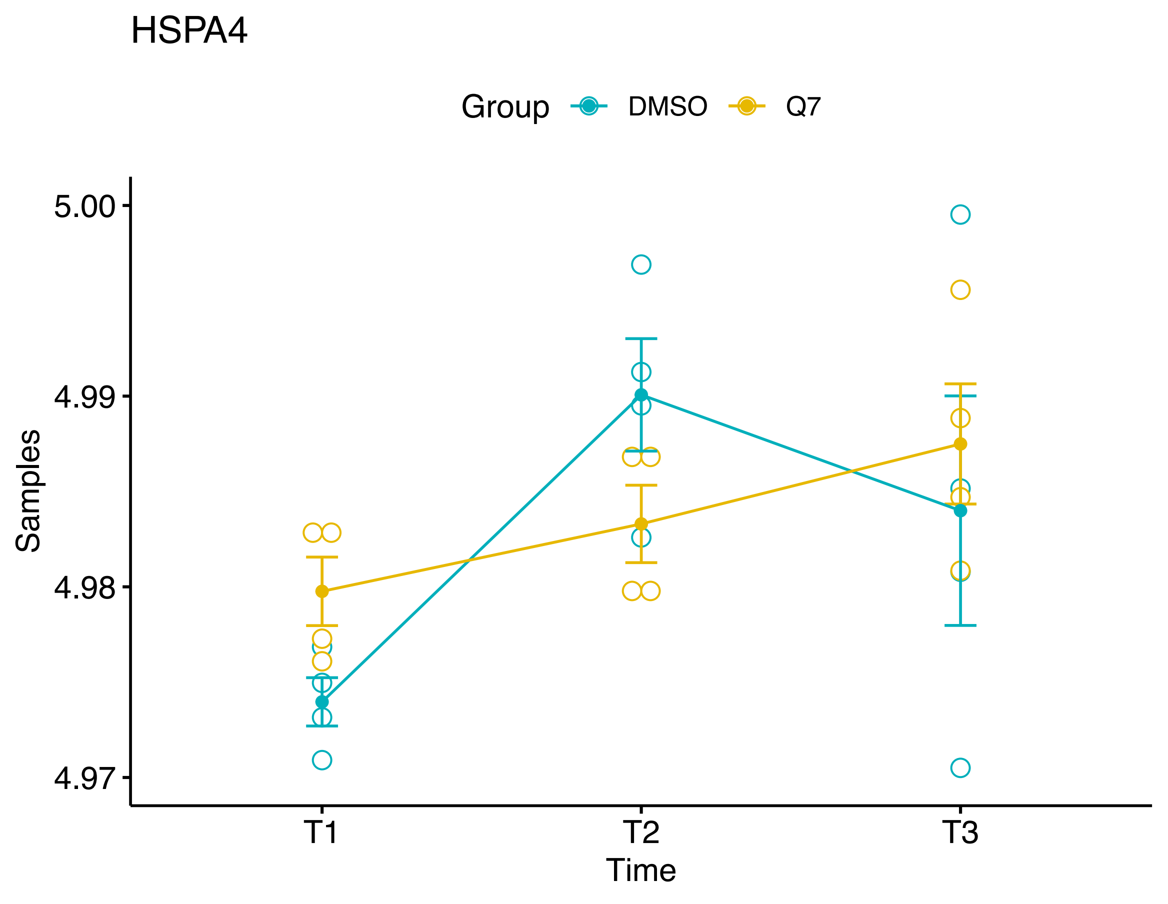

HSPA4
Group
DMSO
Q7
Samples
5.00
4.99
4.98
4.97
T1
T2
T3
Time

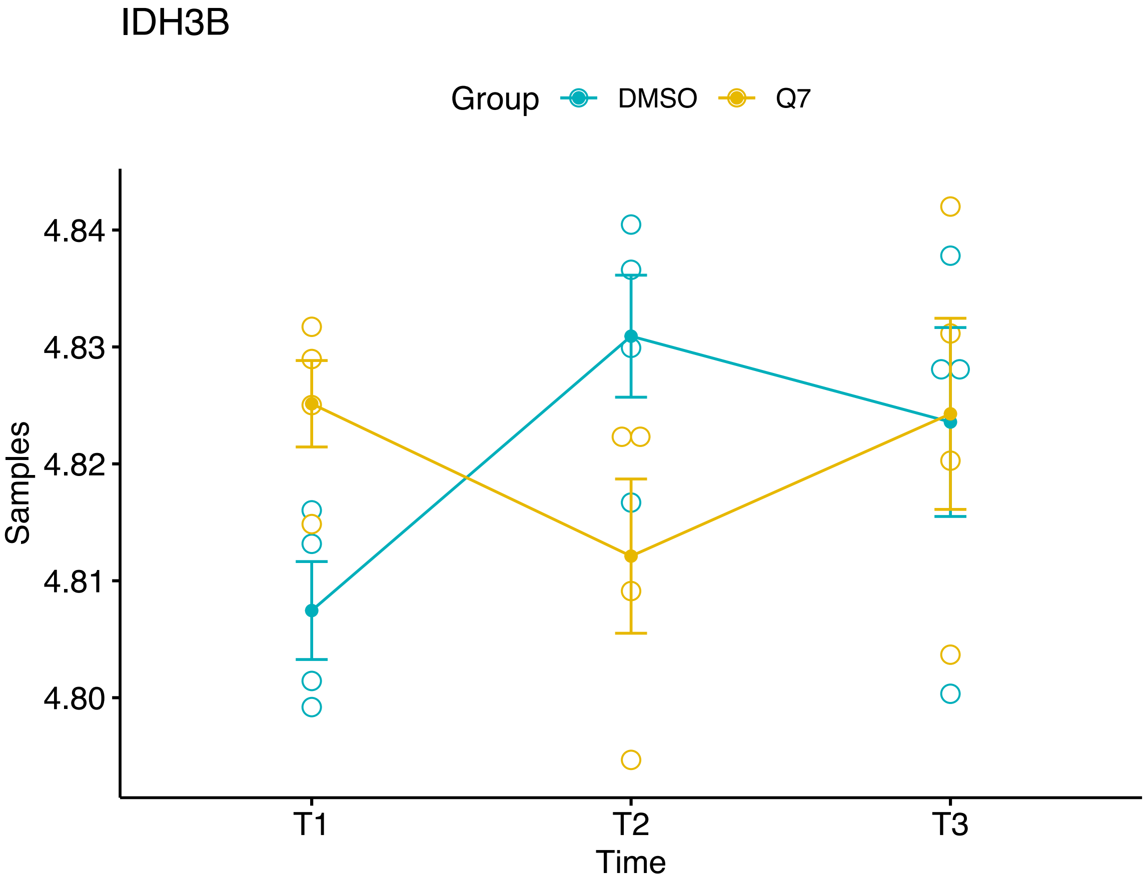
IDH3B
Group
DMSO
Q7
Samples
4.84
4.83
4.82
4.81
4.80
T1
T2
T3
Time

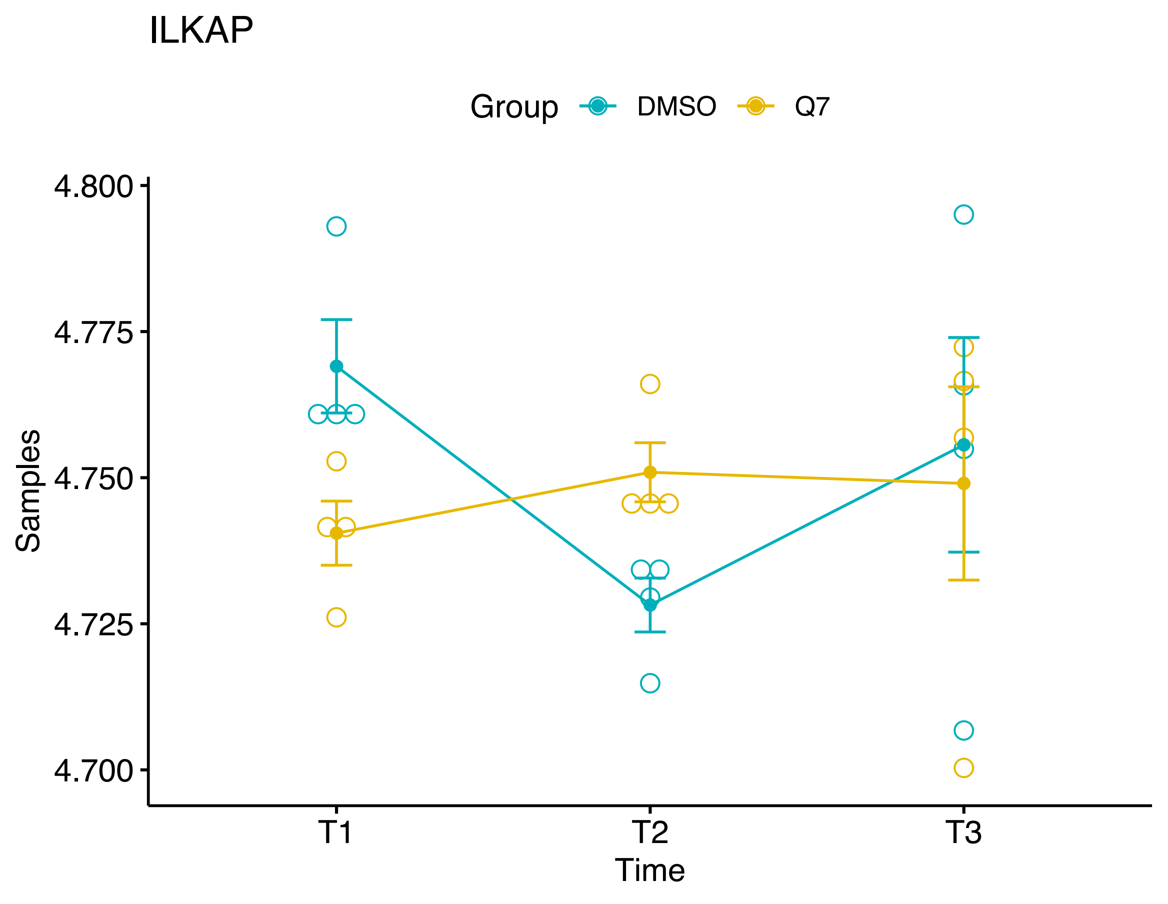

ILKAP
Group
DMSO
Q7
4.800
4.775
4.750
4.725
4.700
Samples
T1
T2
T3
Time

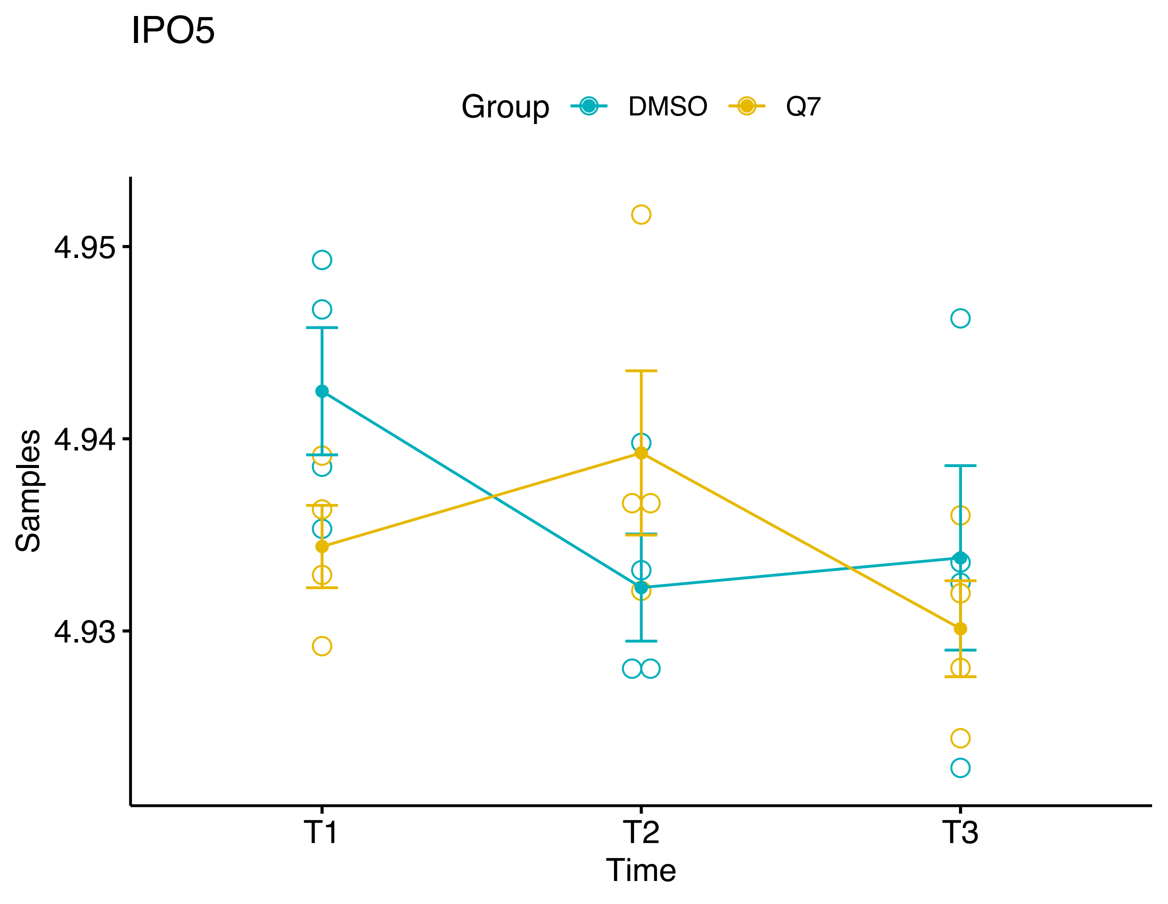
IPO5
Group
DMSO
Q7
Samples
4.95
4.94
4.93
T1
T2
T3
Time

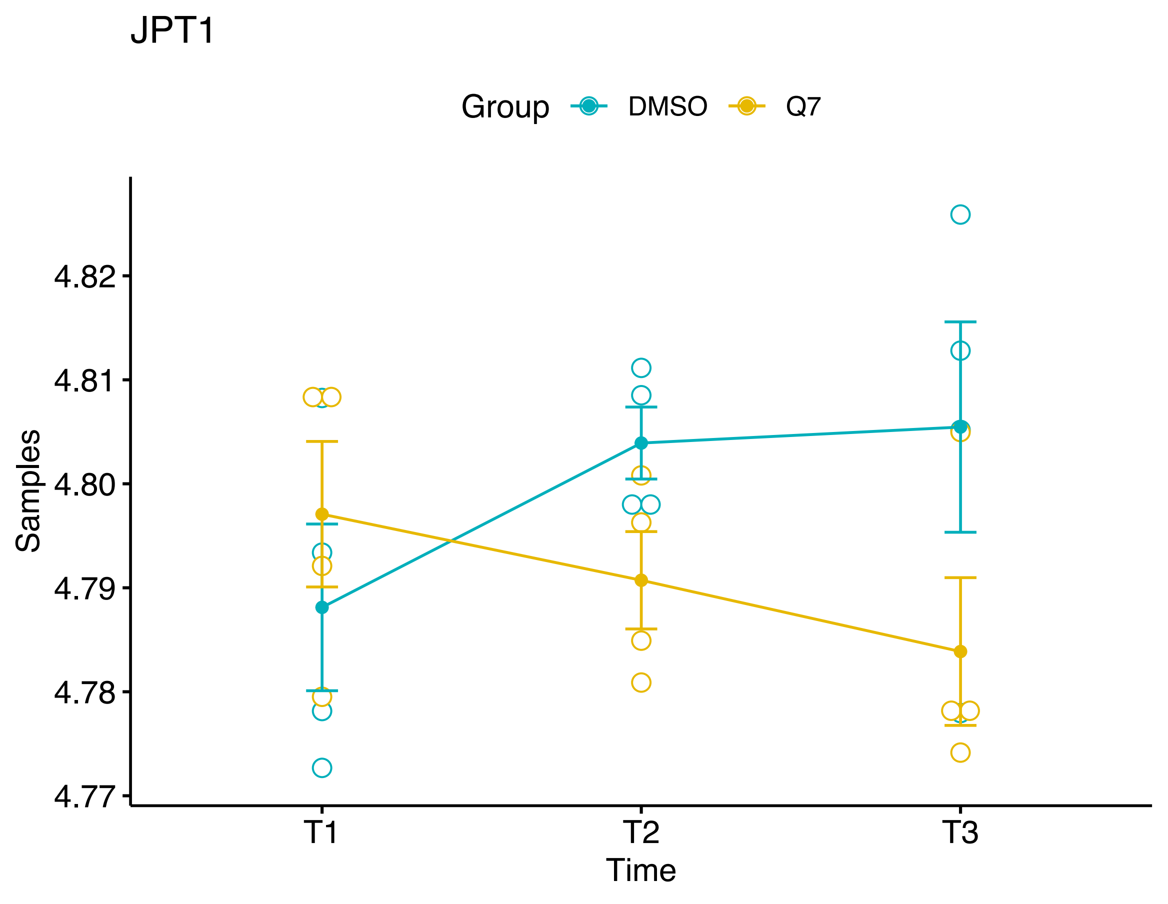
JPT1
Group
DMSO
Q7
Samples
4.82
4.81
4.80
4.79
4.78
4.77
T1
T2
T3
Time

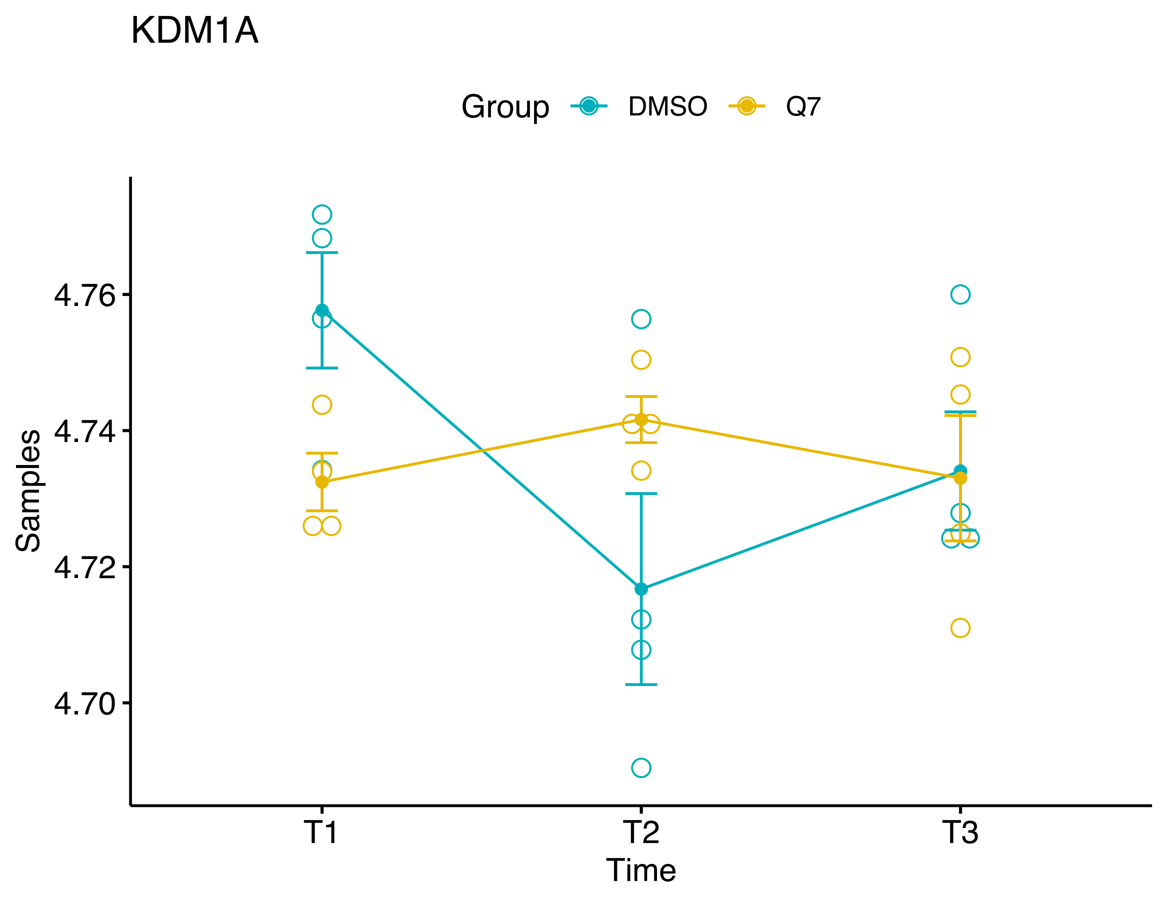

KDM1A
Group
DMSO
Q7
Samples
4.76
4.74
4.72
4.70
T1
T2
T3
Time

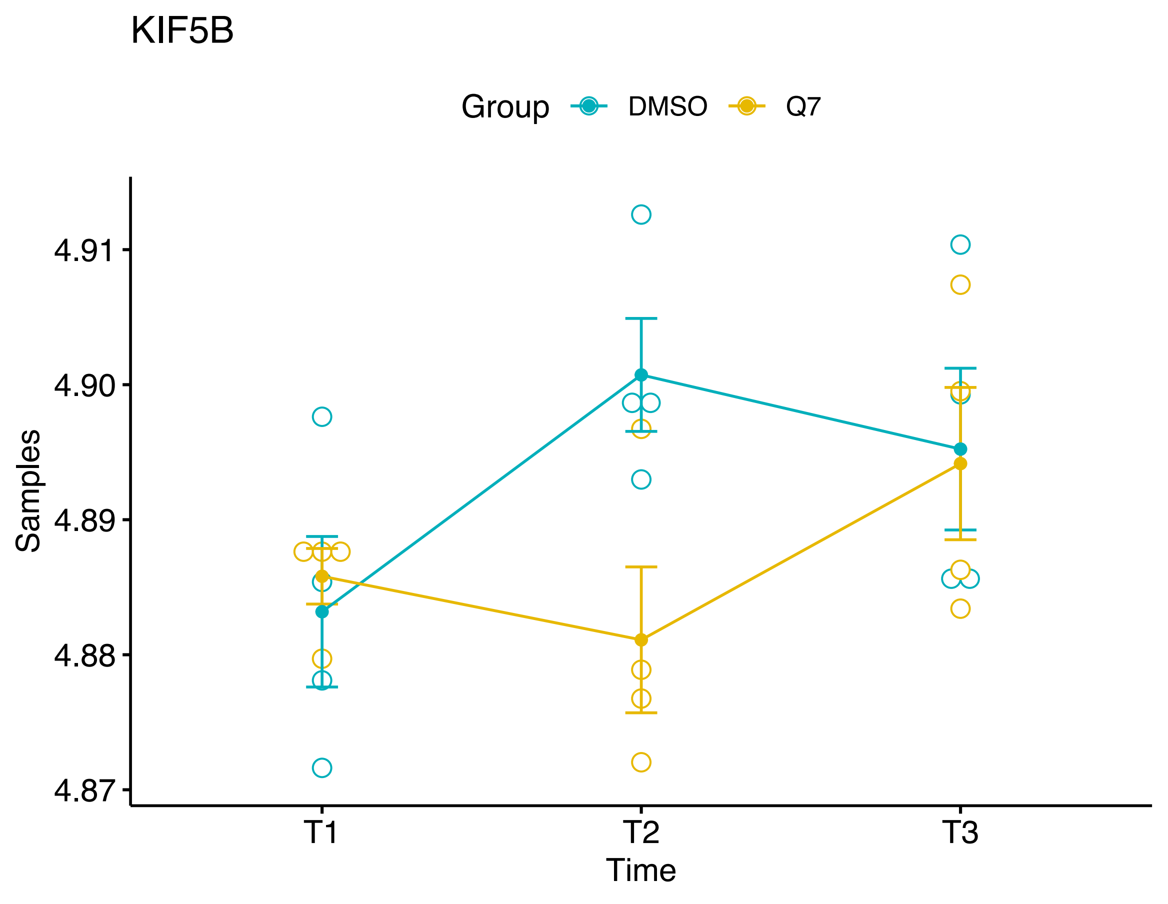

KIF5B
Group
DMSO
Q7
Samples
4.91
4.90
4.89
4.88
4.87
T1
T2
T3
Time

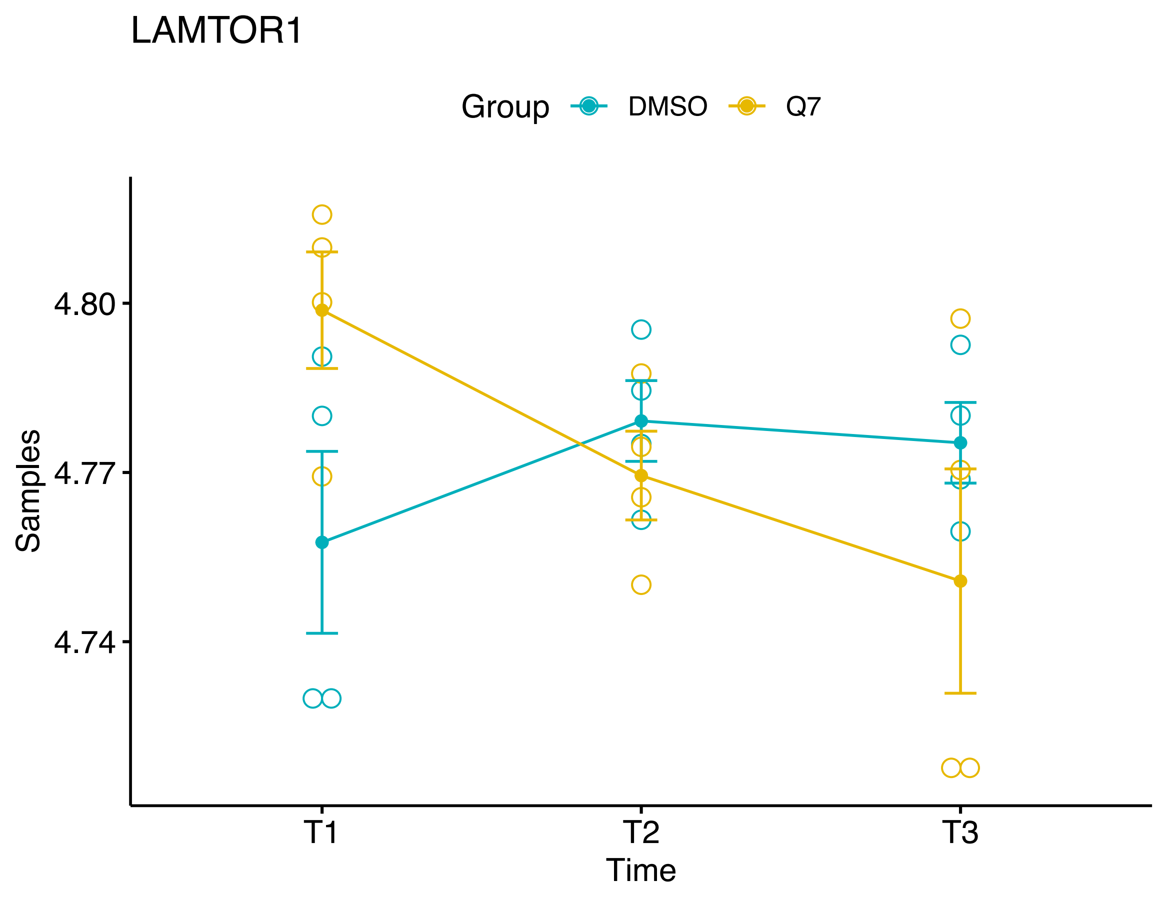

LAMTOR1
Group
DMSO
Q7
Samples
4.80
4.77
4.74
T1
T2
T3
Time

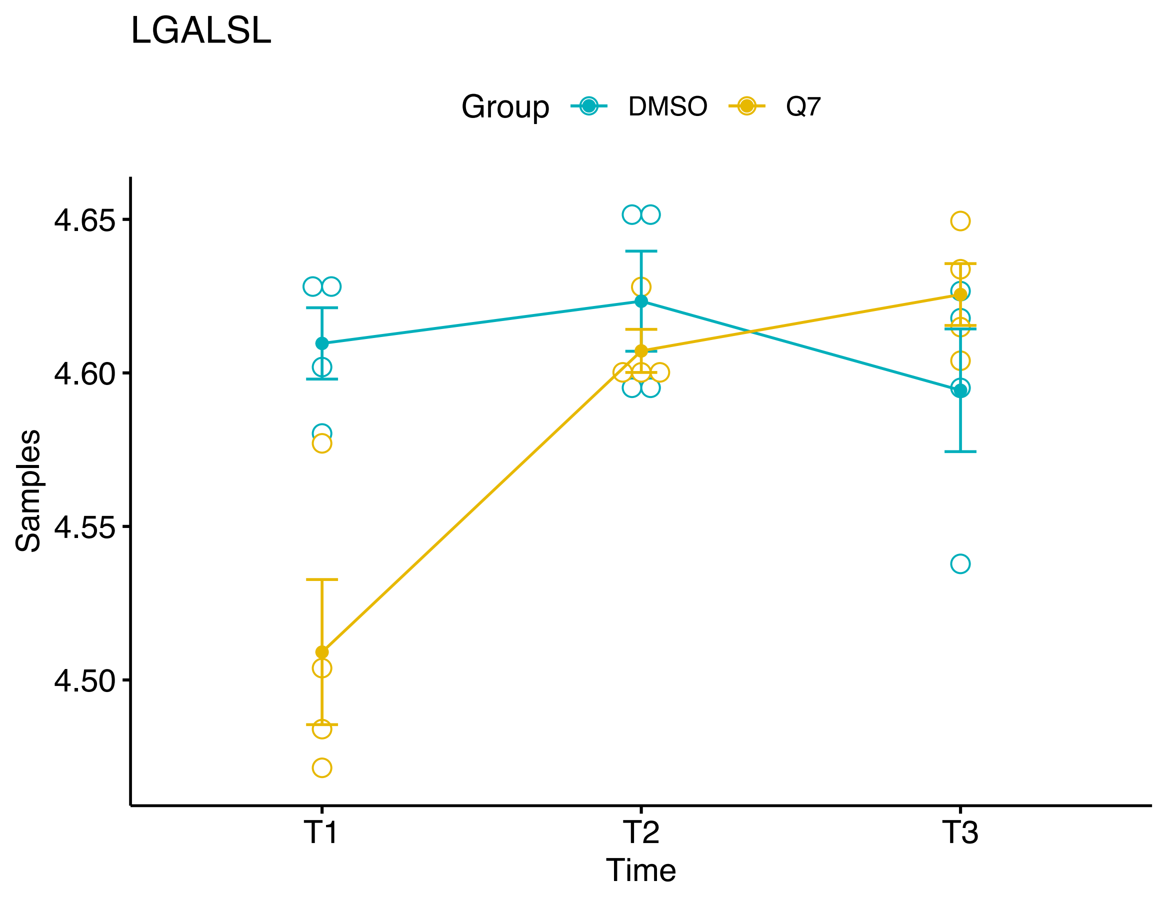

LGALSL
Group
DMSO
Q7
4.65
4.60
4.55
4.50
Samples
T1
T2
T3
Time

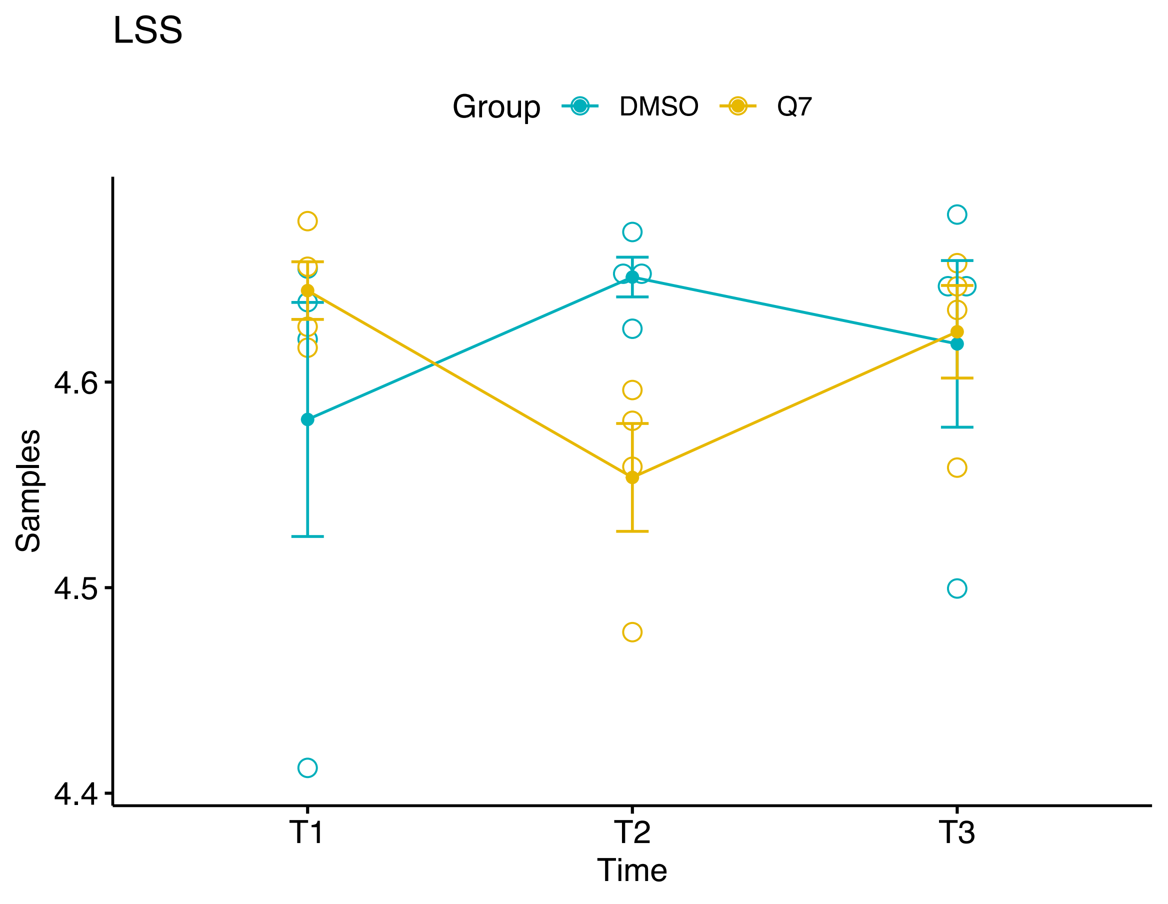

LSS
Group
DMSO
Q7
Samples
4.6
4.5
4.4
T1
T2
T3
Time

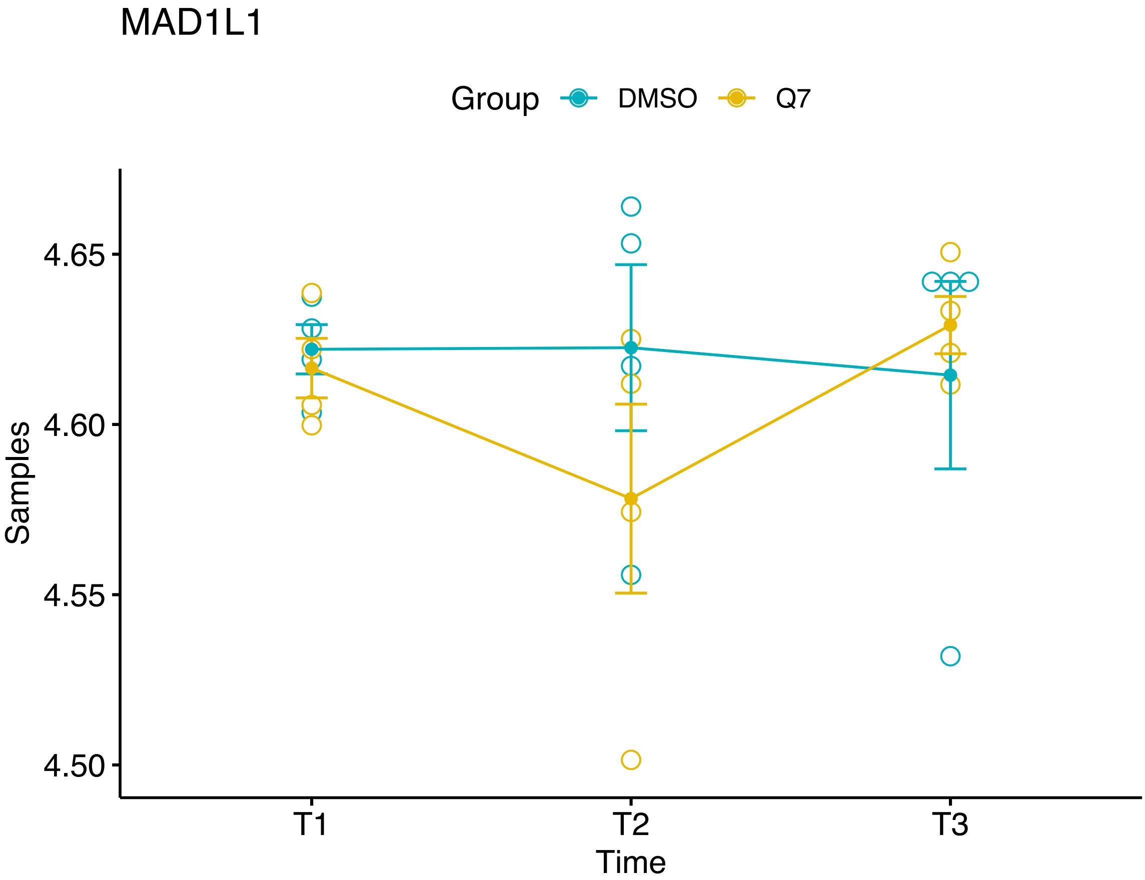
MAD1L1
Group
DMSO
Q7
Samples
4.65
4.60
4.55
4.50
T1
T2
T3
Time

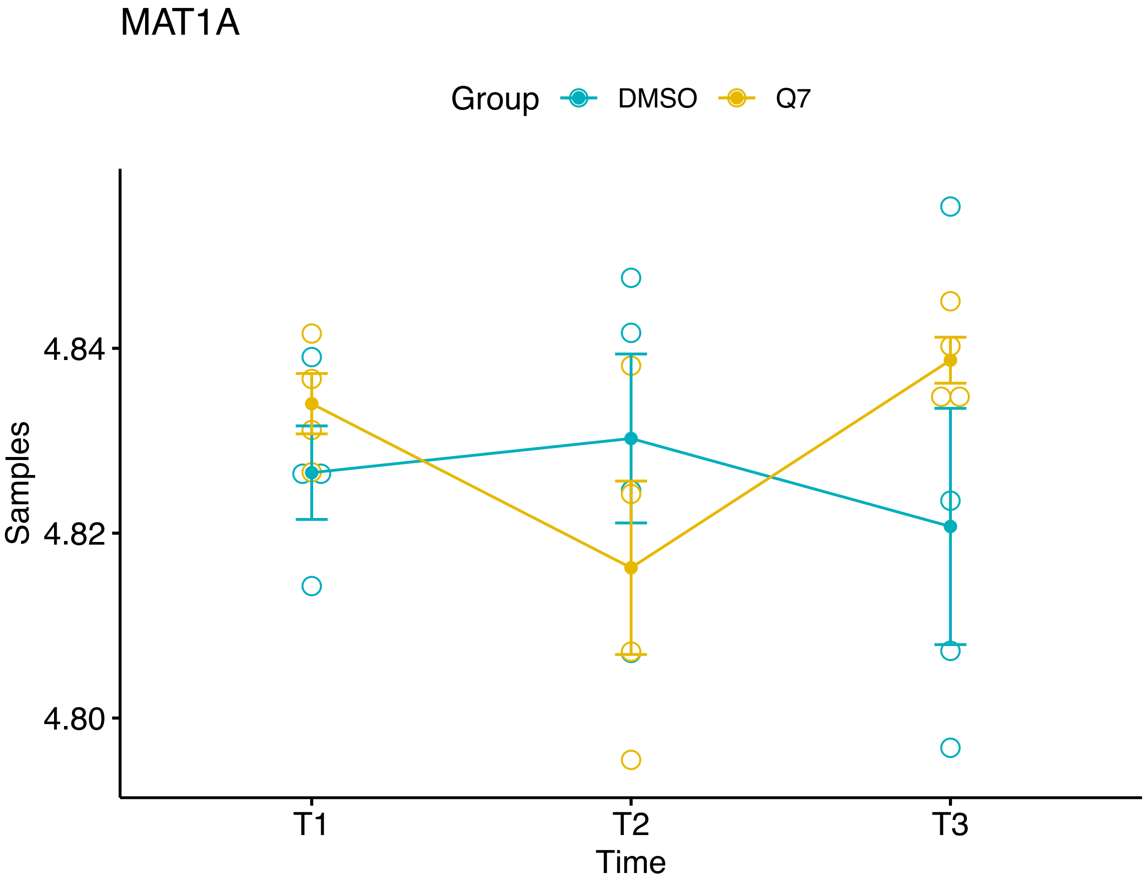
MAT1A
Group
DMSO
Q7
Samples
4.84
4.82
4.80
T1
T2
T3
Time

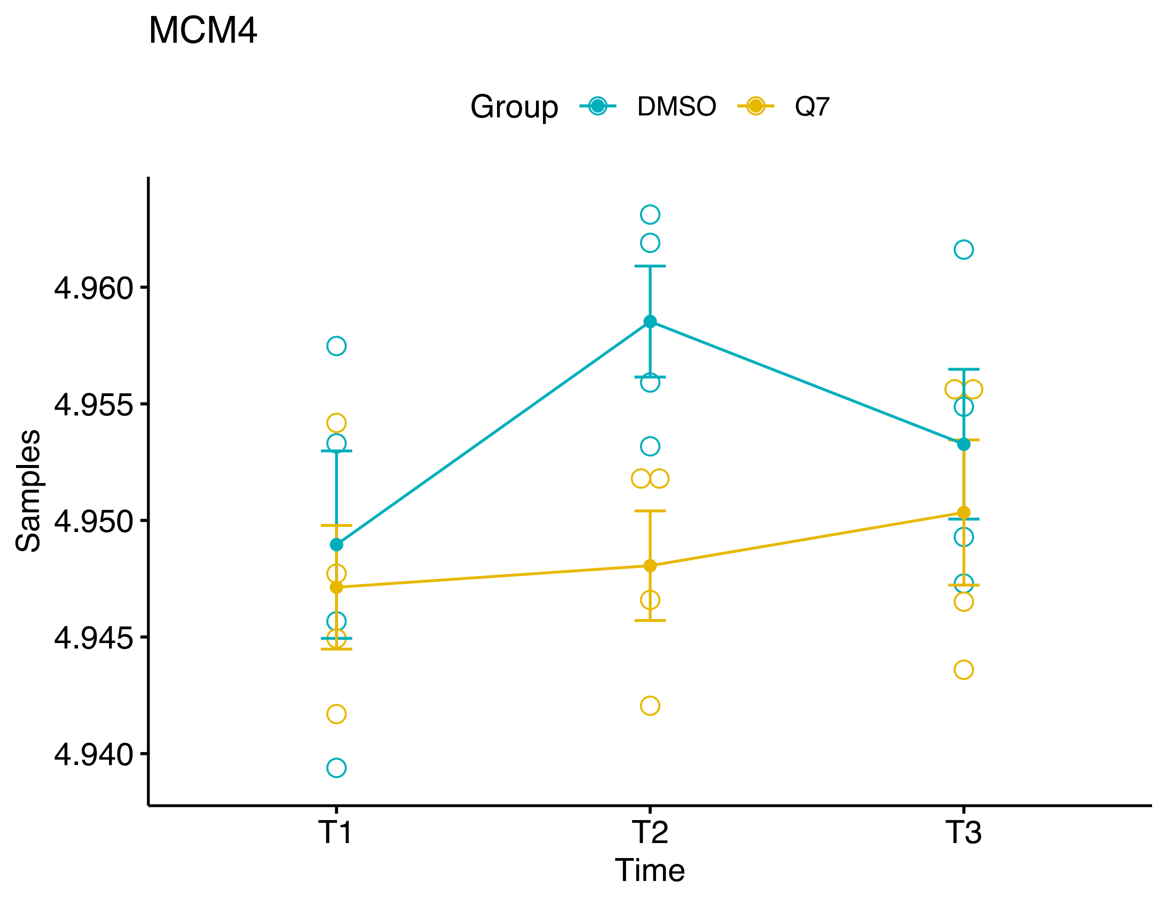

MCM4
Group
DMSO
Q7
Samples
4.960
4.955
4.950
4.945
4.940
T1
T2
T3
Time

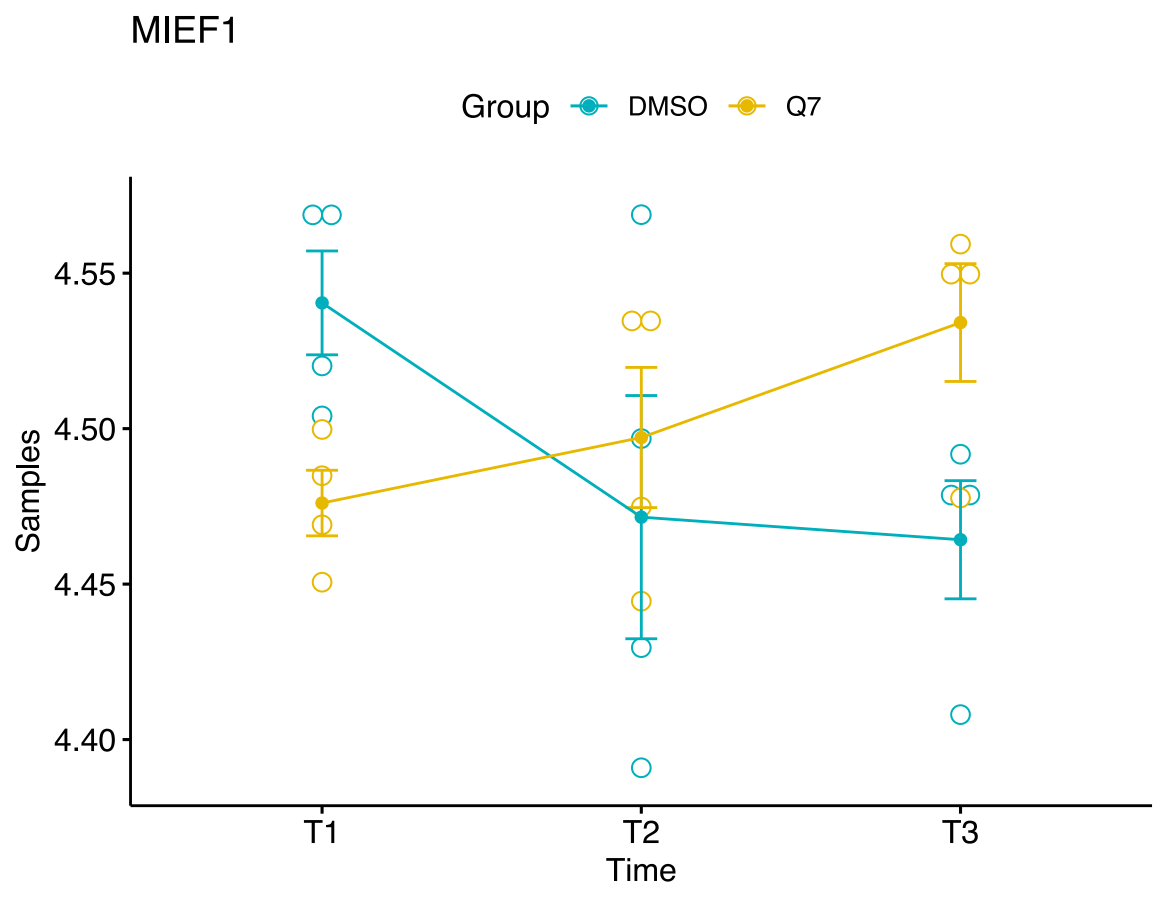
MIEF1
Group
DMSO
Q7
Samples
4.55
4.50
4.45
4.40
T1
T2
T3
Time

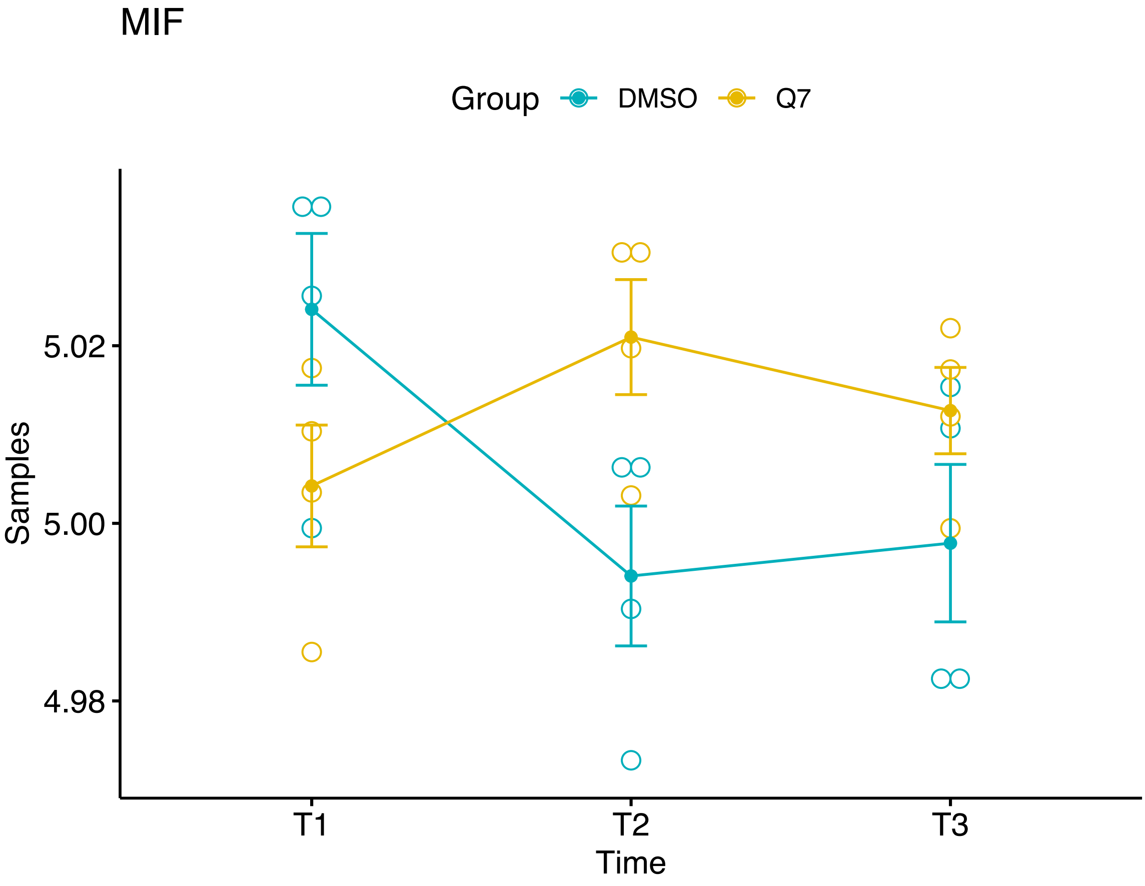

MIF
Group
DMSO
Q7
Samples
5.02
5.00
4.98
T1
T2
T3
Time

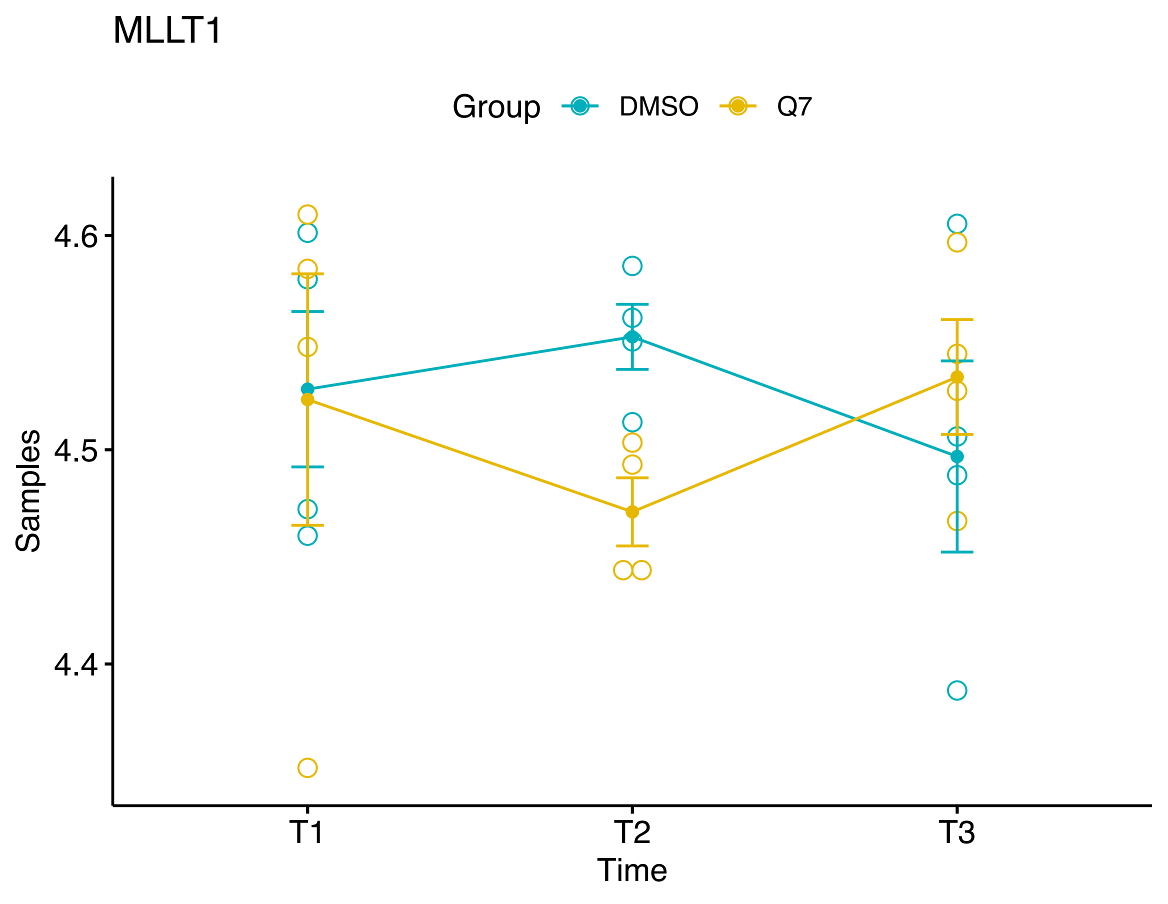

MLLT1
Group
DMSO
Q7
Samples
4.6
4.5
4.4
T1
T2
T3
Time

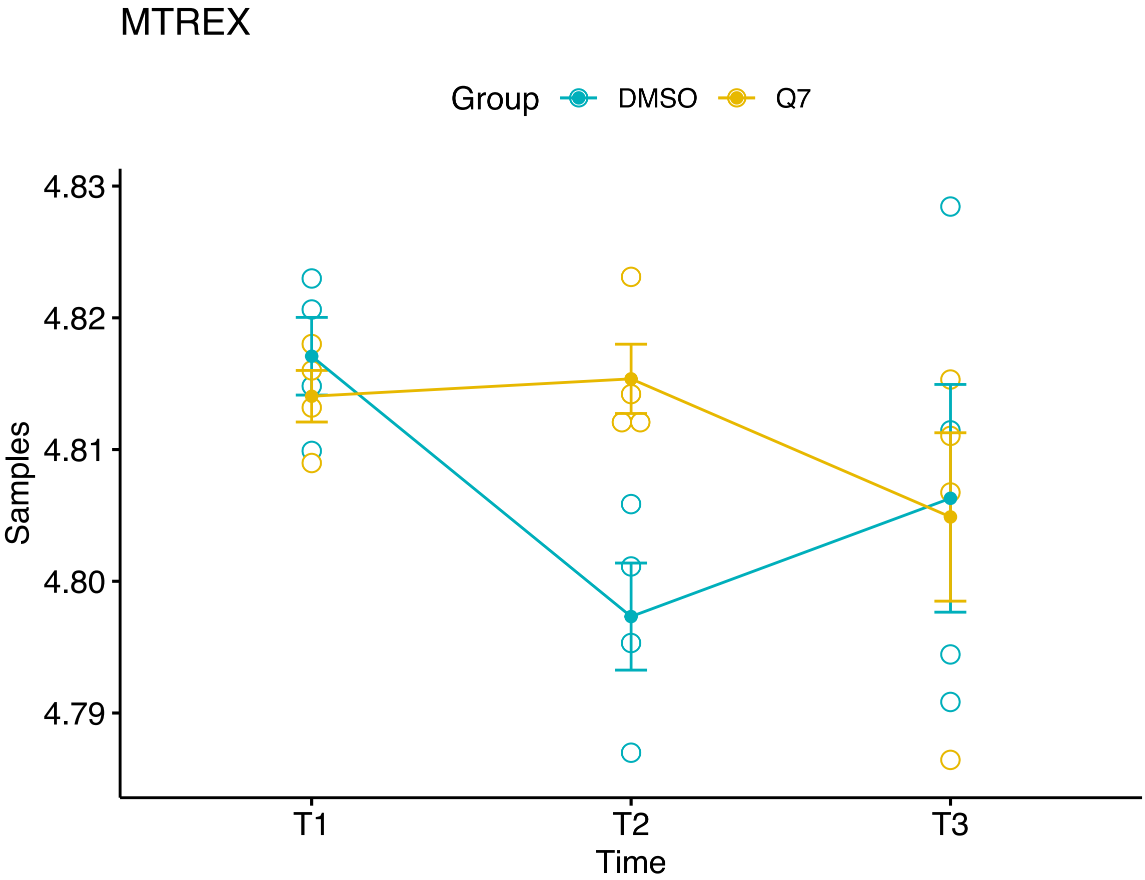

MTREX
Group
DMSO
Q7
4.83
4.82
4.81
4.80
4.79
Samples
T1
T2
T3
Time

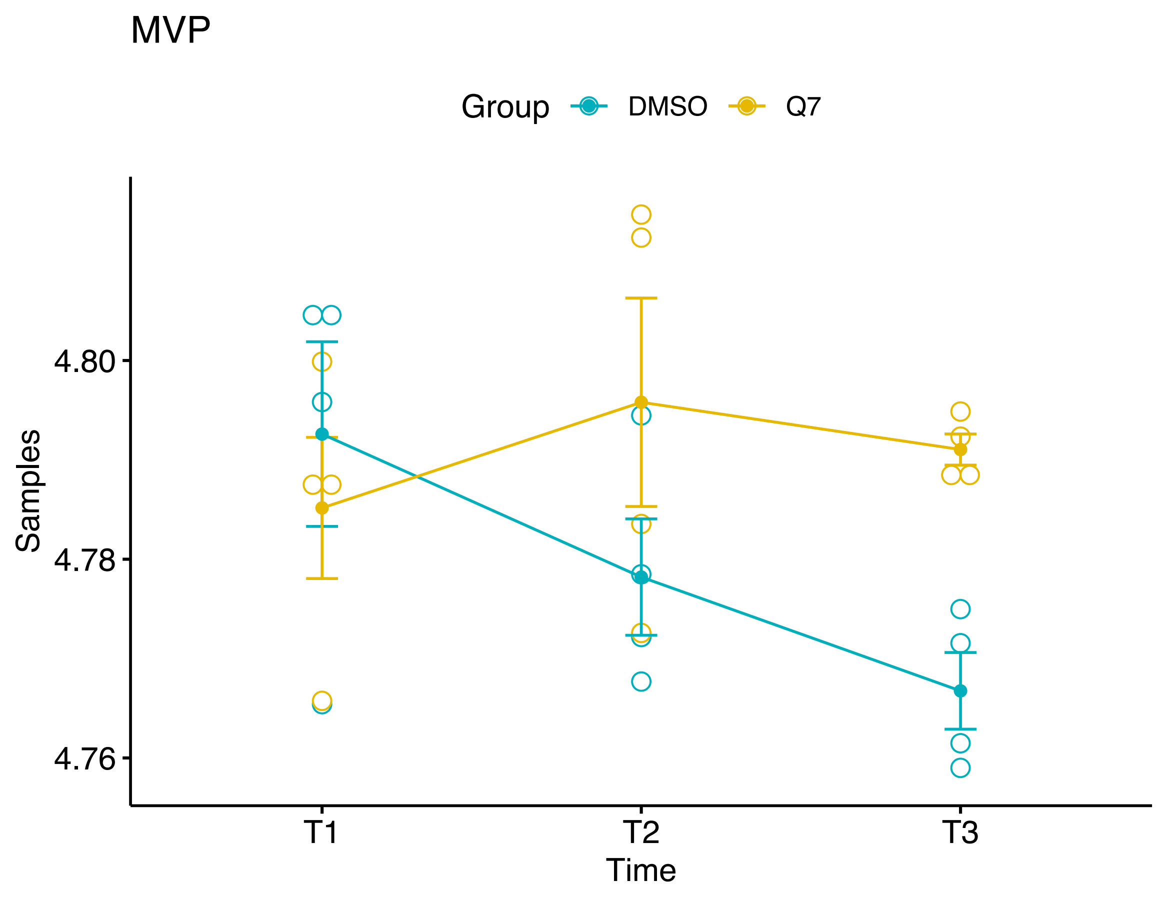
MVP
Group
DMSO
Q7
Samples
4.80
4.78
4.76
T1
T2
T3
Time

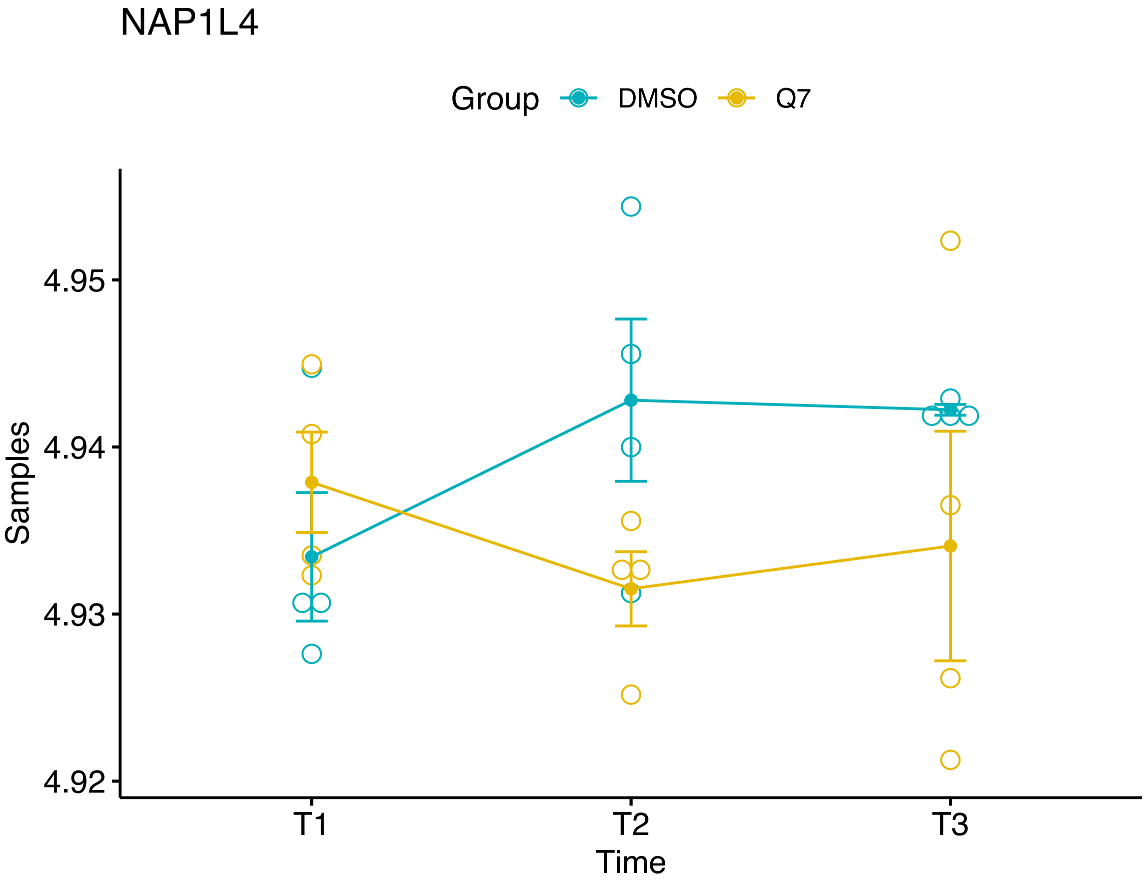

NAP1L4
Group
DMSO
Q7
Samples
4.95
4.94
4.93
4.92
T1
T2
T3
Time

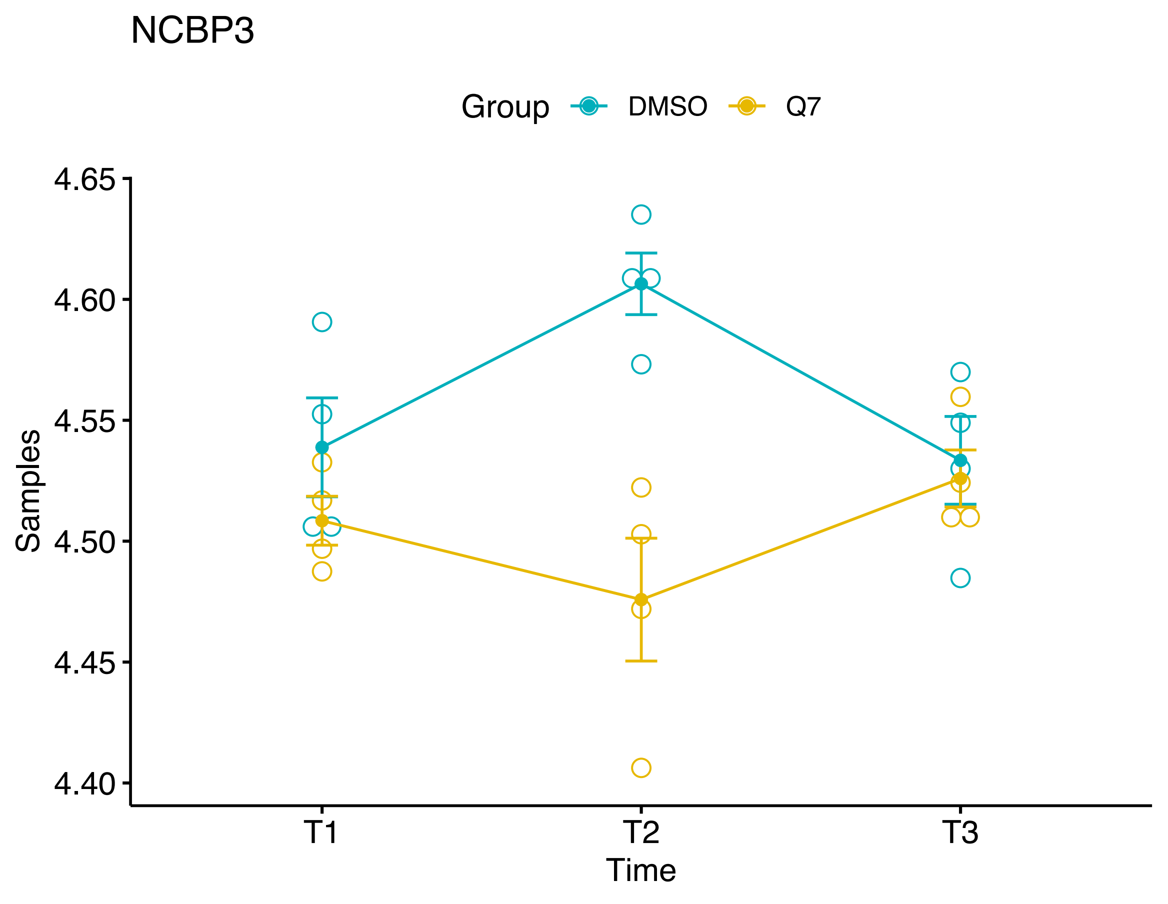

NCBP3
Group
DMSO
Q7
Samples
4.65
4.60
4.55
4.50
4.45
4.40
T1
T2
T3
Time

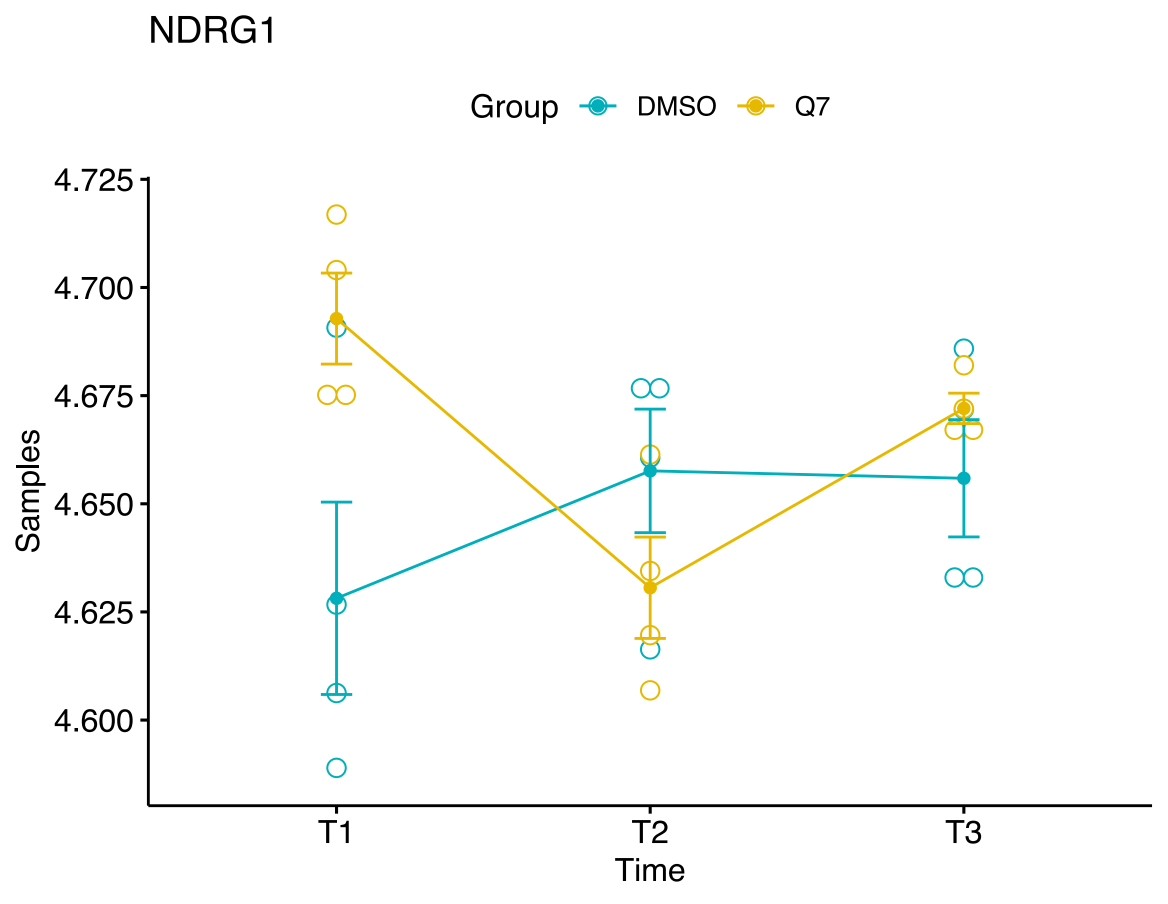

NDRG1
Group
DMSO
Q7
4.725
4.700
4.675
4.650
4.625
4.600
Samples
T1
T2
T3
Time

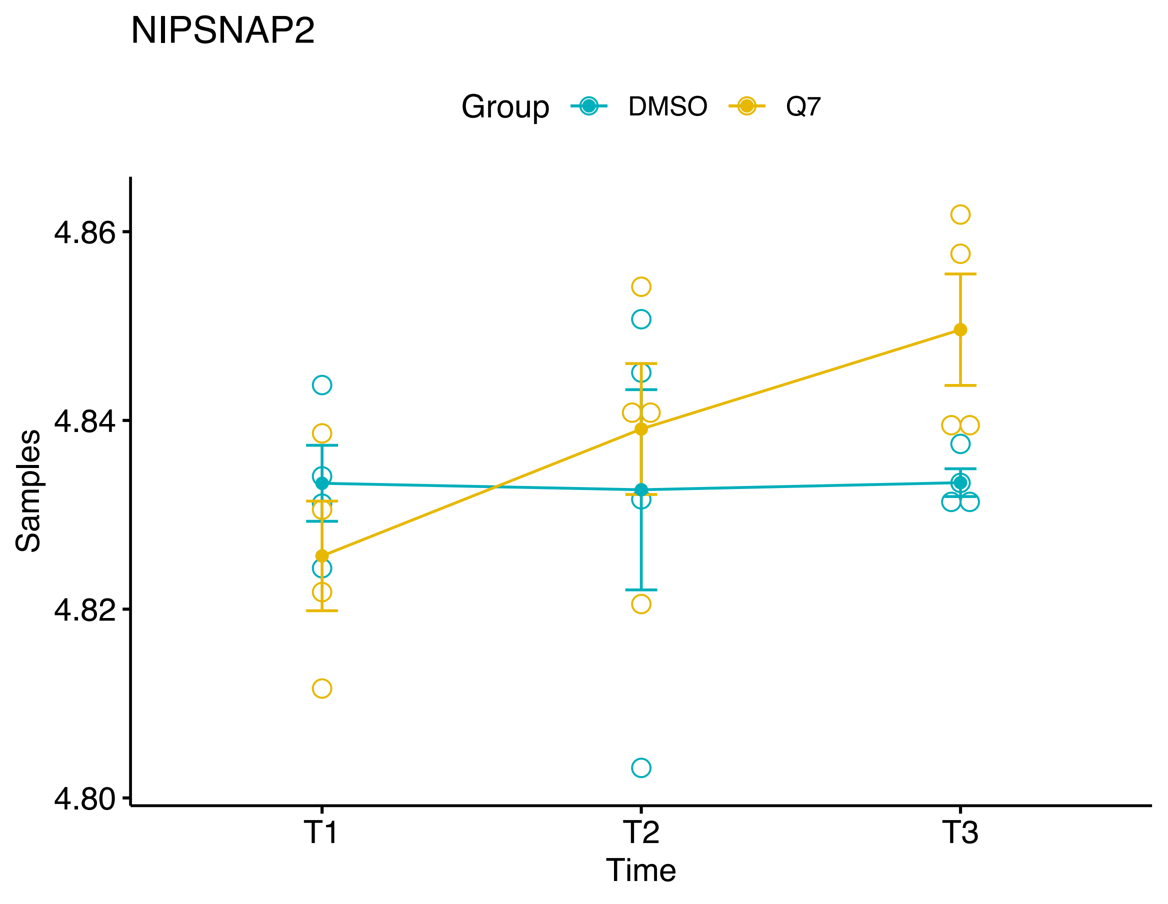
NIPSNAP2
Group
DMSO
Q7
4.86
4.84
4.82
4.80
Samples
T1
T2
T3
Time

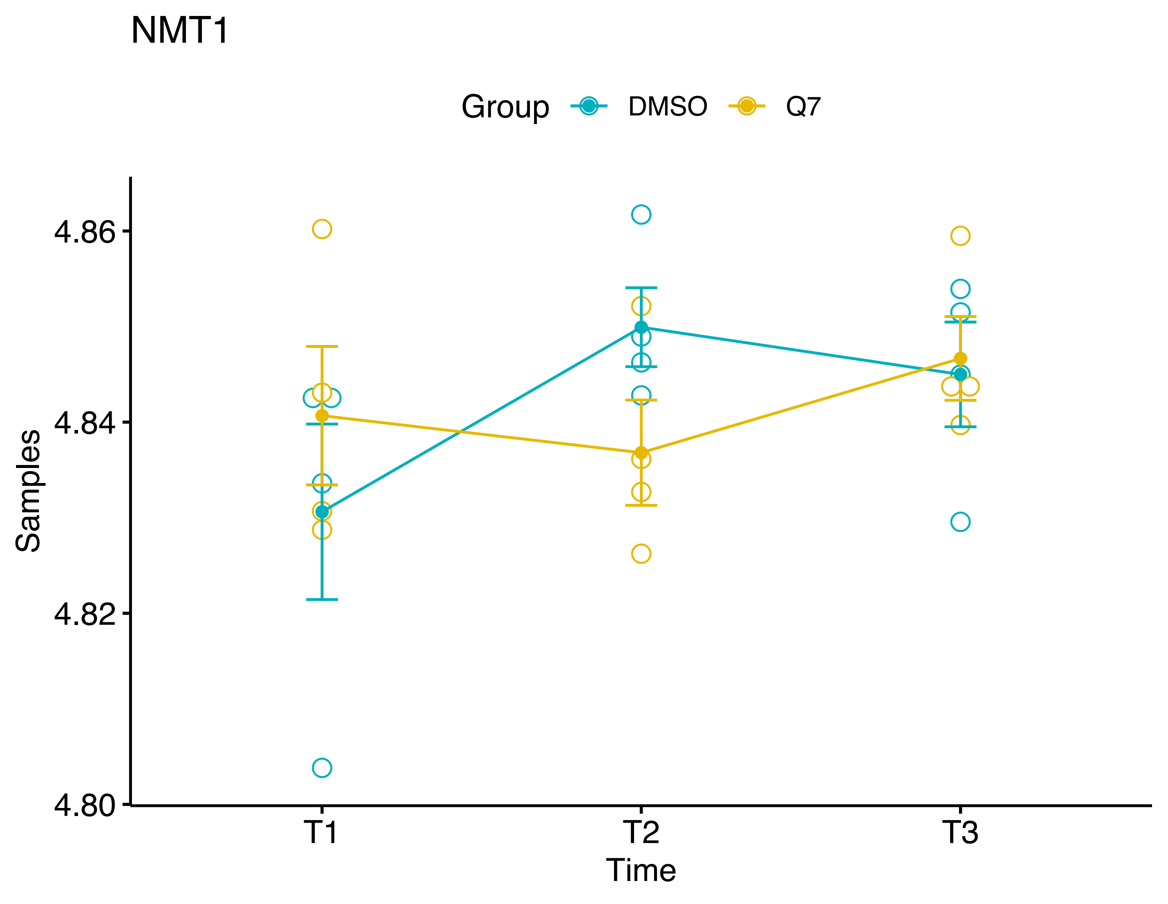

NMT1
Group
DMSO
Q7
4.86
4.84
4.82
4.80
Samples
T1
T2
T3
Time

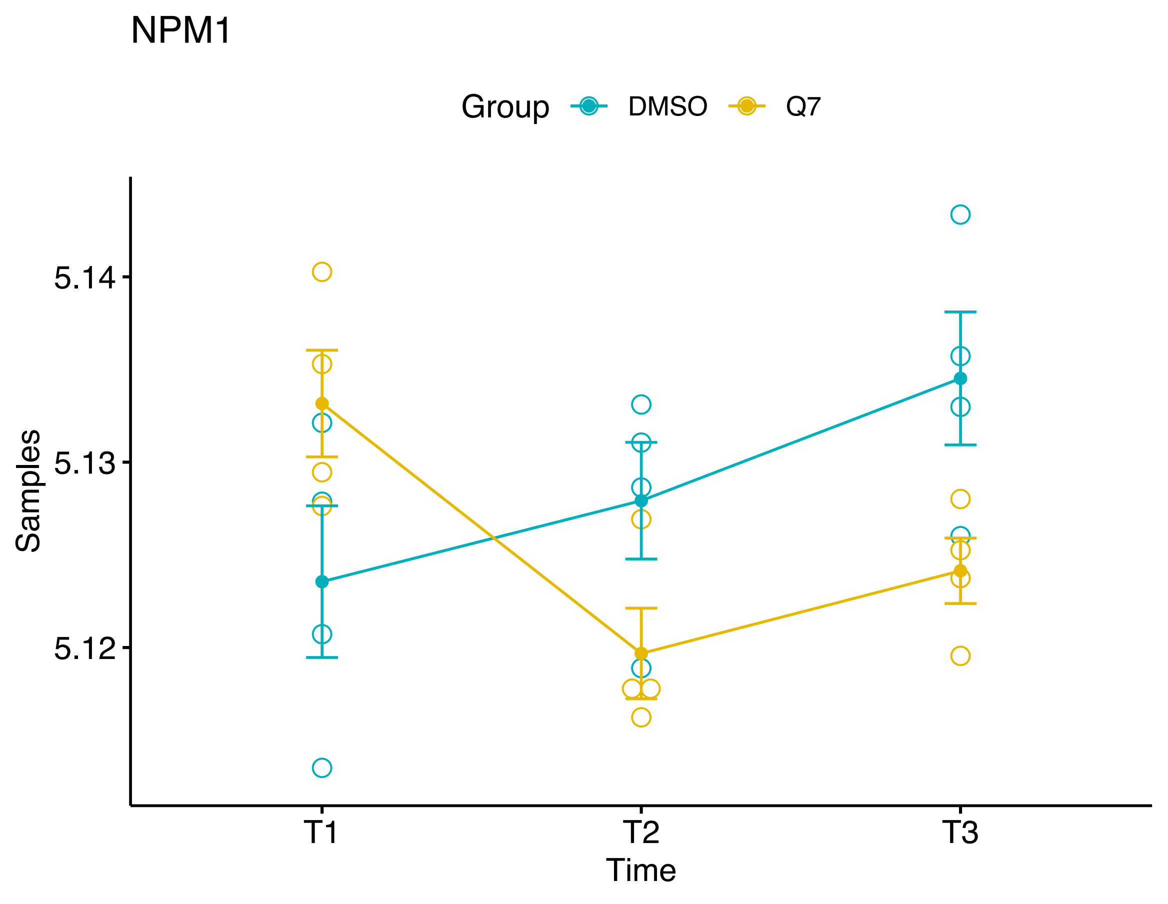

NPM1
Group
DMSO
Q7
Samples
5.14
5.13
5.12
T1
T2
T3
Time

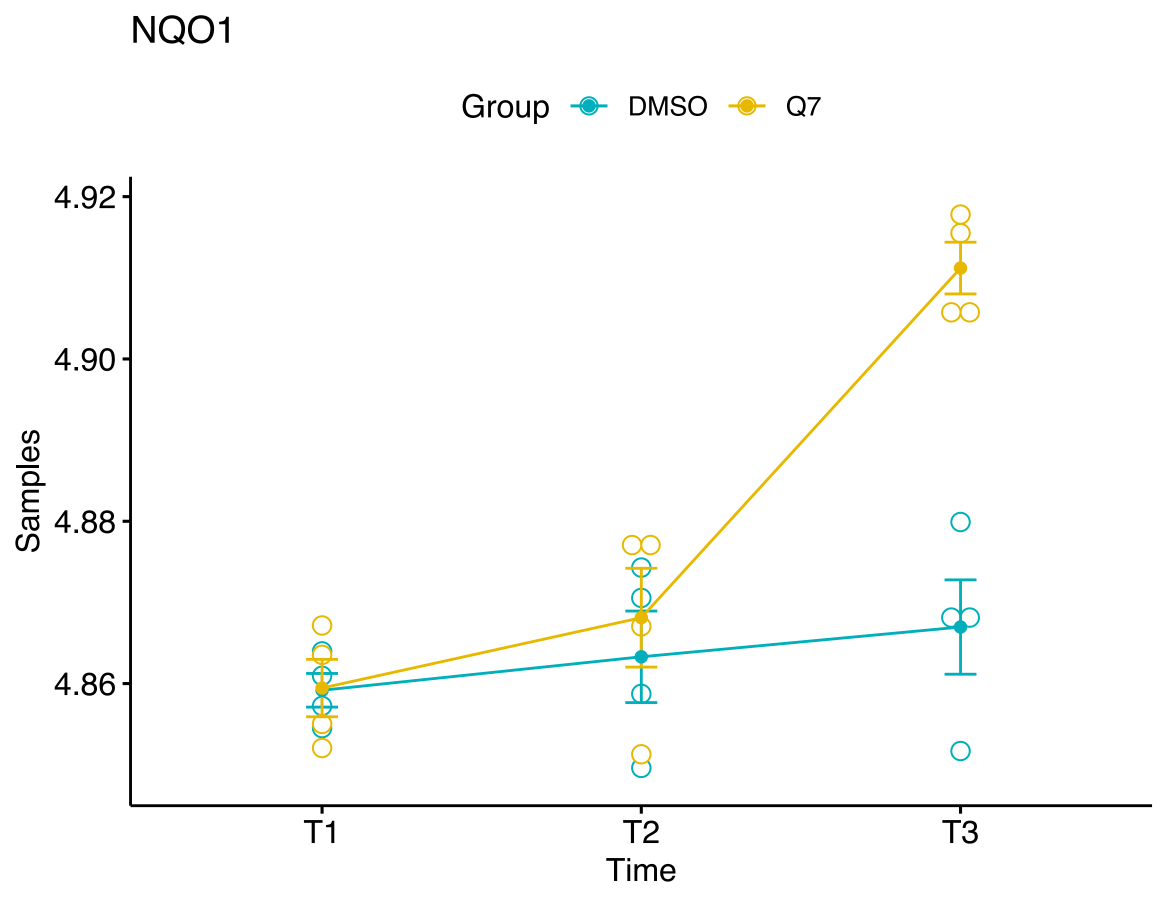

NQO1
Group
DMSO
Q7
Samples
4.92
4.90
4.88
4.86
T1
T2
T3
Time

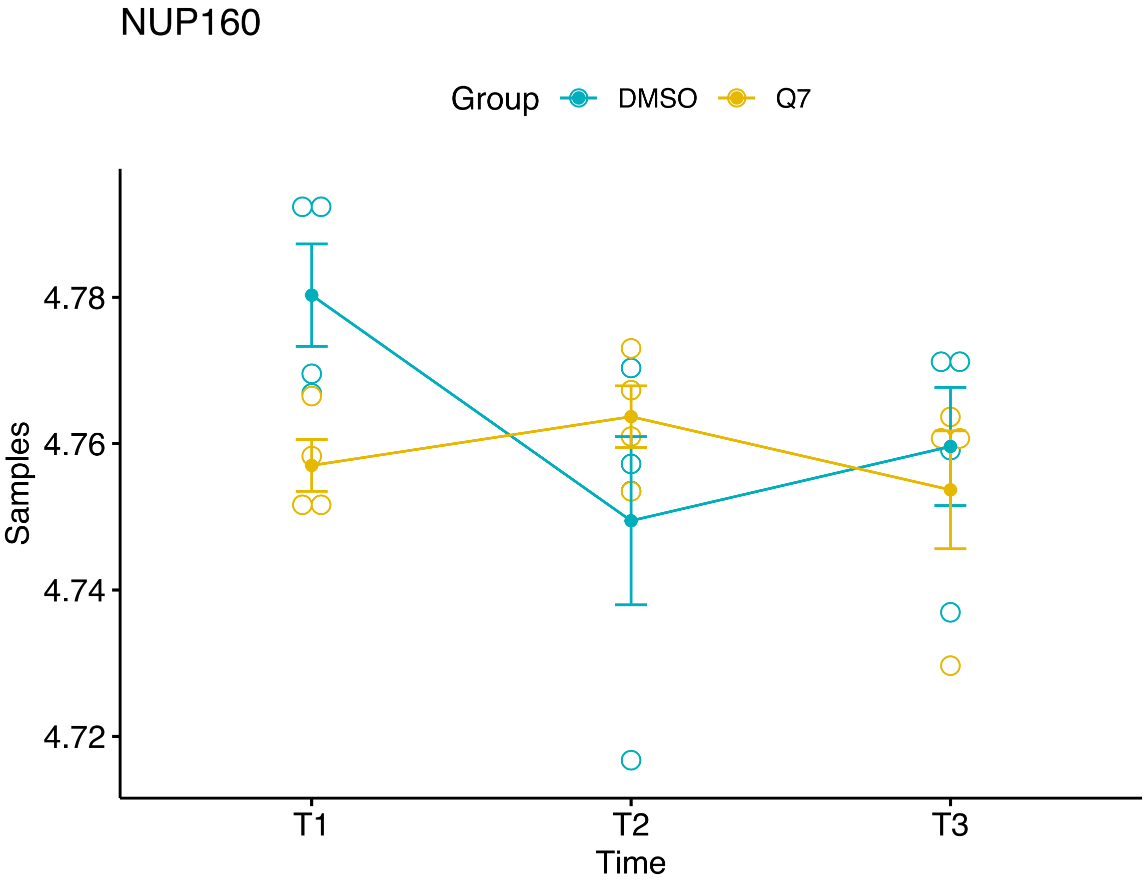
NUP160
Group
DMSO
Q7
4.78
4.76
4.74
4.72
Samples
T1
T2
T3
Time

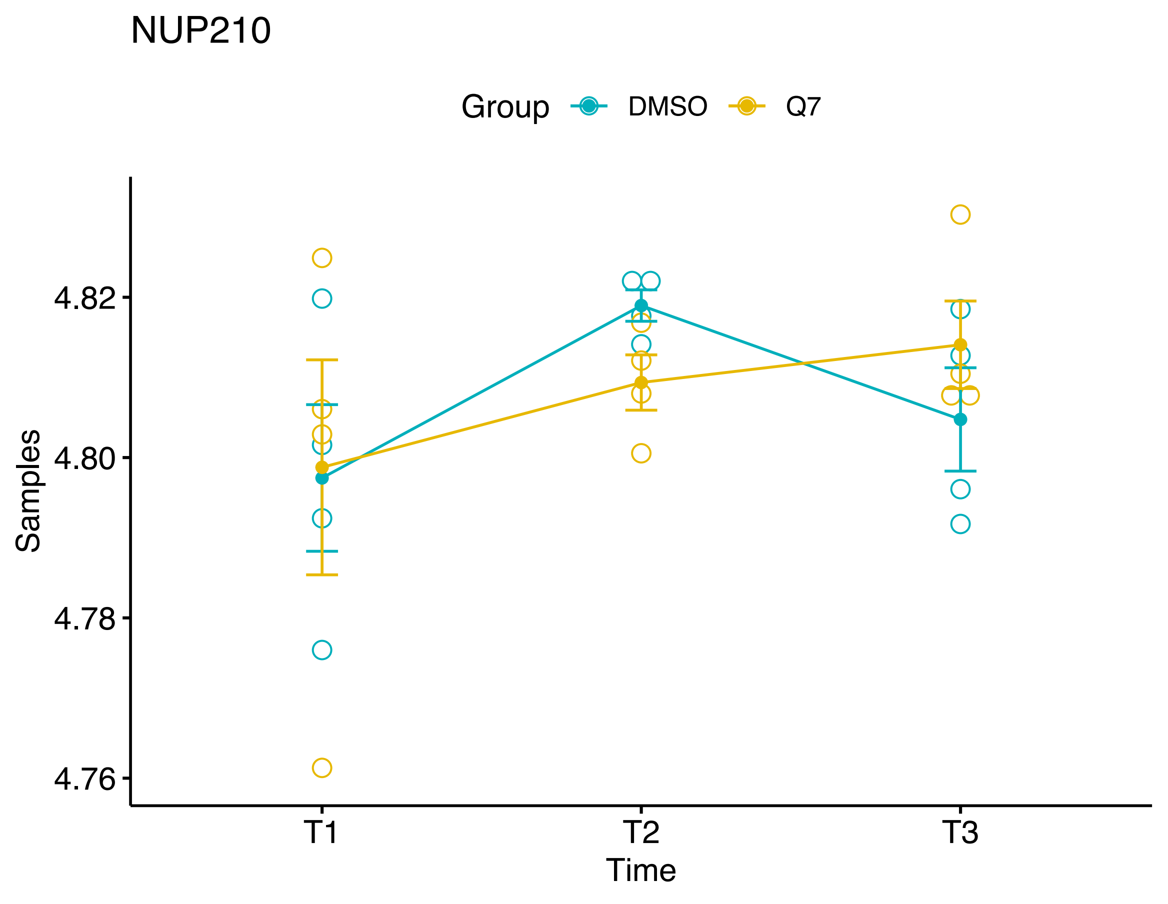
NUP210
Group
DMSO
Q7
4.82
4.80
4.78
4.76
Samples
T1
T2
T3
Time

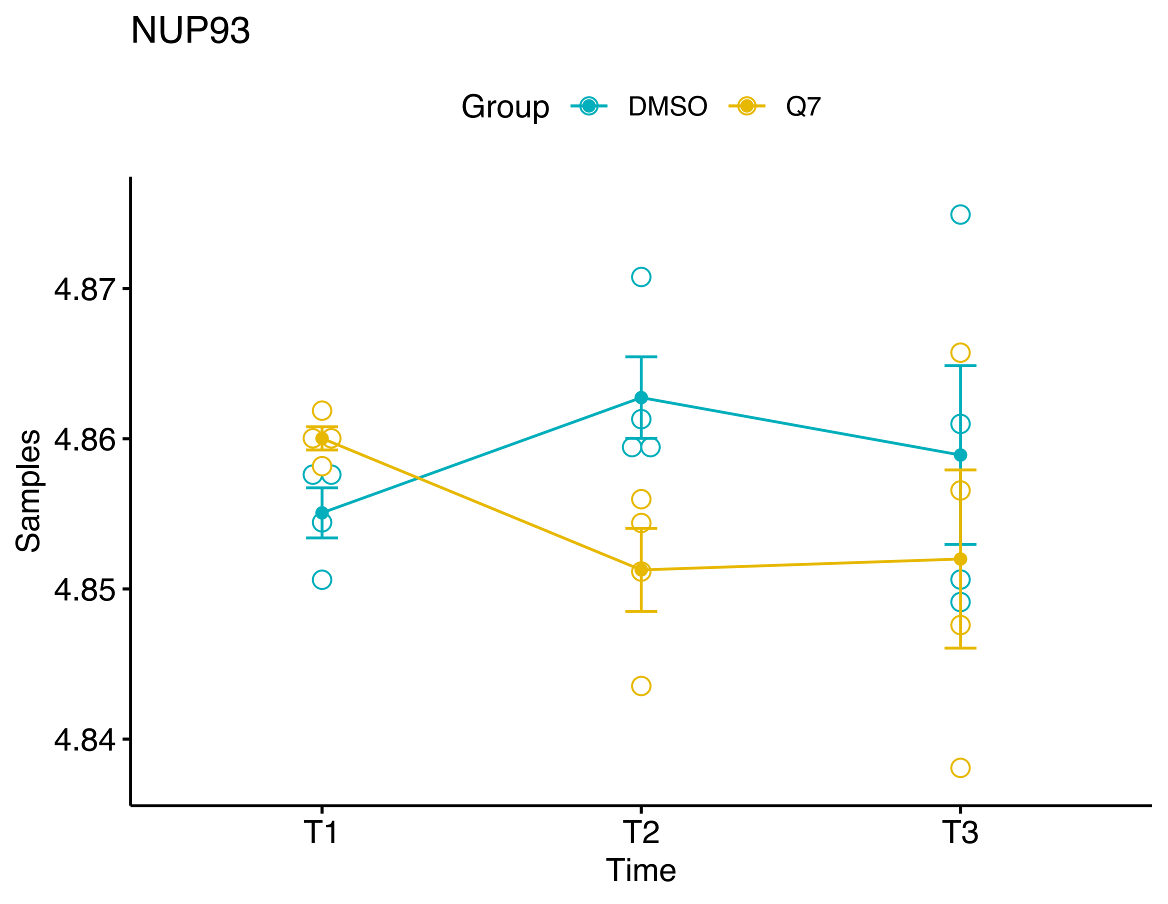

NUP93
Group
DMSO
Q7
Samples
4.87
4.86
4.85
4.84
T1
T2
T3
Time

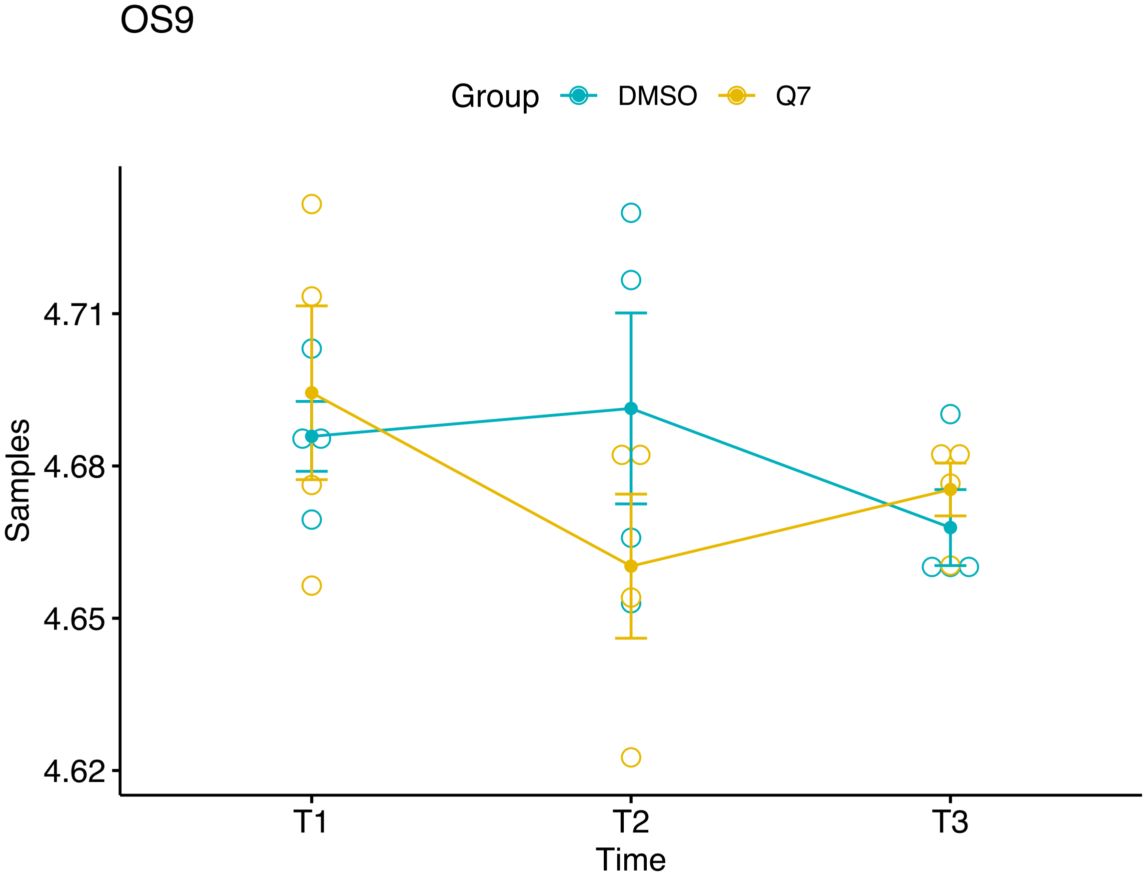

OS9
Group
DMSO
Q7
4.71
4.68
4.65
4.62
Samples
T1
T2
T3
Time

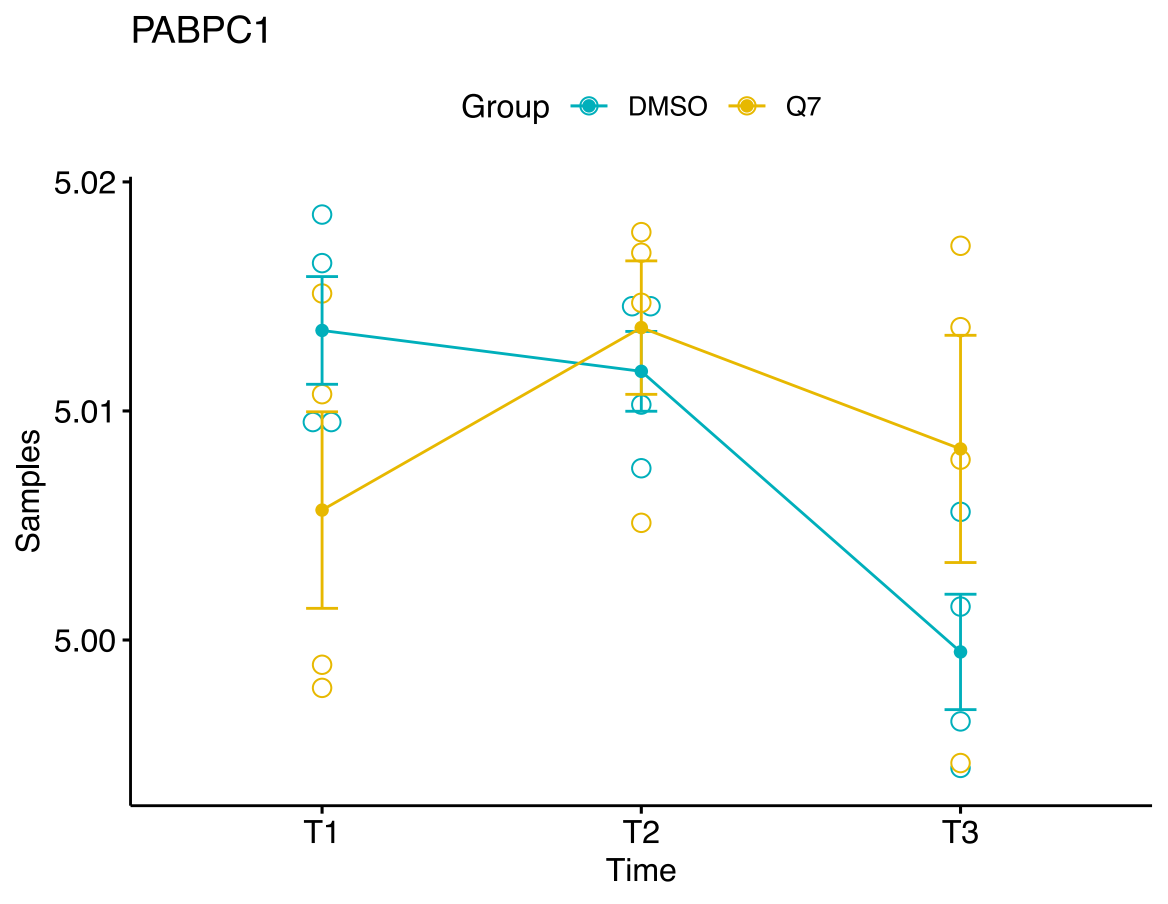
PABPC1
Group
DMSO
Q7
5.02
5.01
5.00
Samples
T1
T2
T3
Time

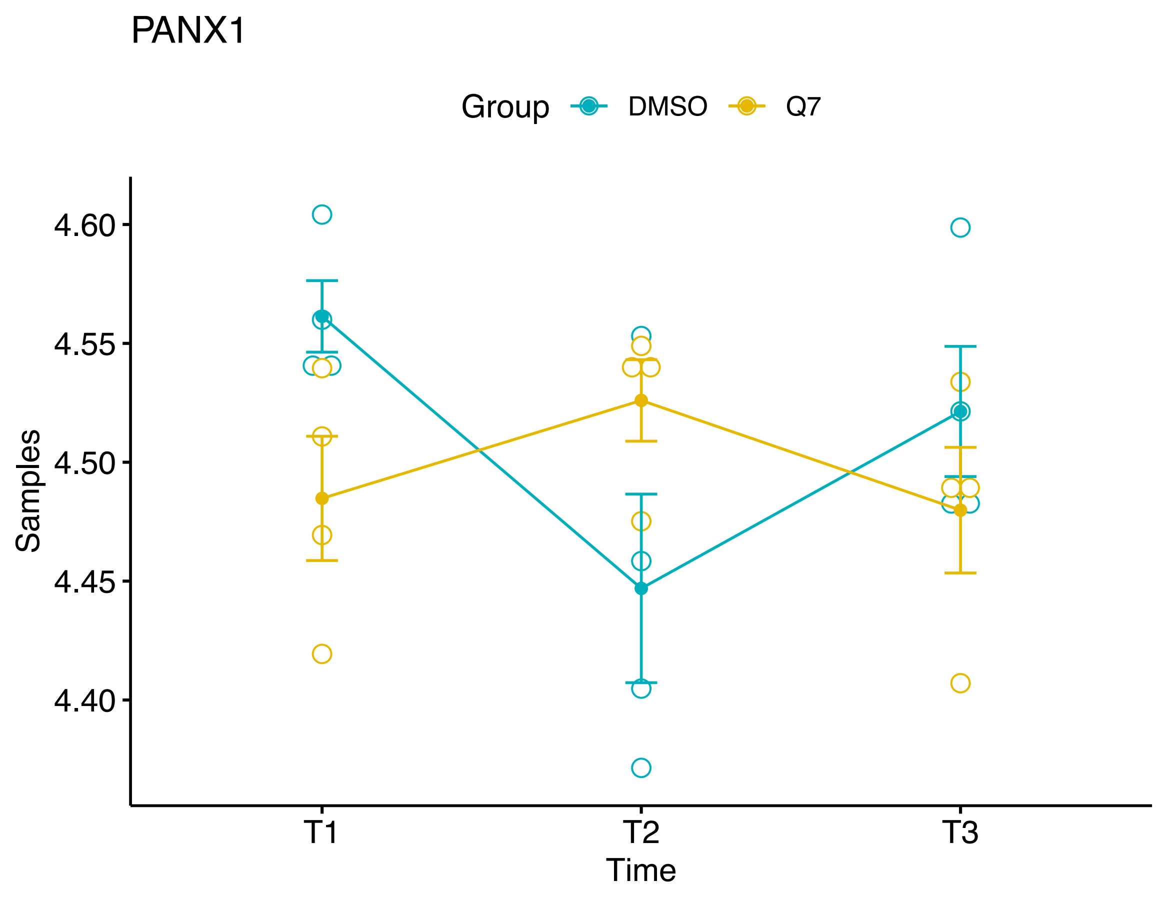

PANX1
Group
DMSO
Q7
Samples
4.60
4.55
4.50
4.45
4.40
T1
T2
T3
Time

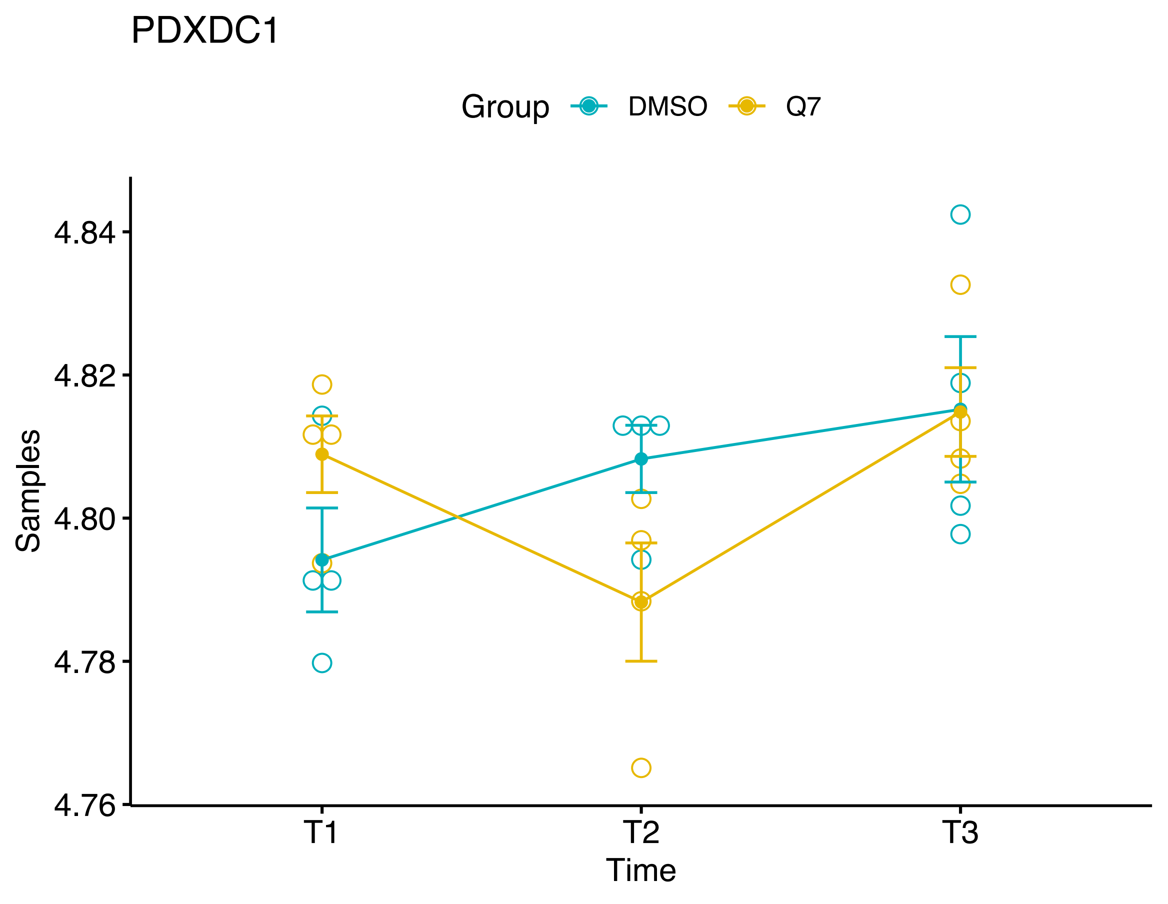

PDXDC1
Group
DMSO
Q7
4.84
4.82
4.80
4.78
4.76
Samples
T1
T2
T3
Time

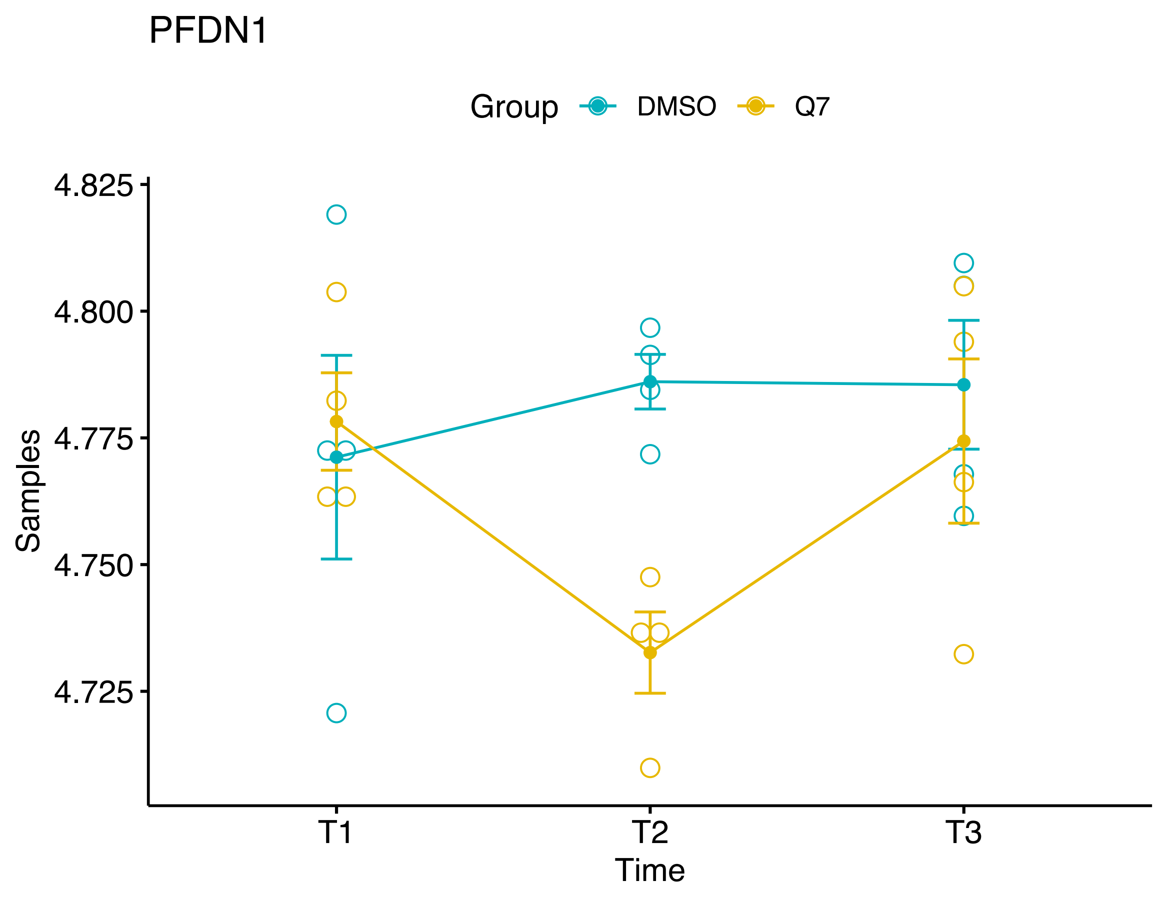

PFDN1
Group
DMSO
Q7
4.825
4.800
4.775
4.750
4.725
Samples
T1
T2
T3
Time

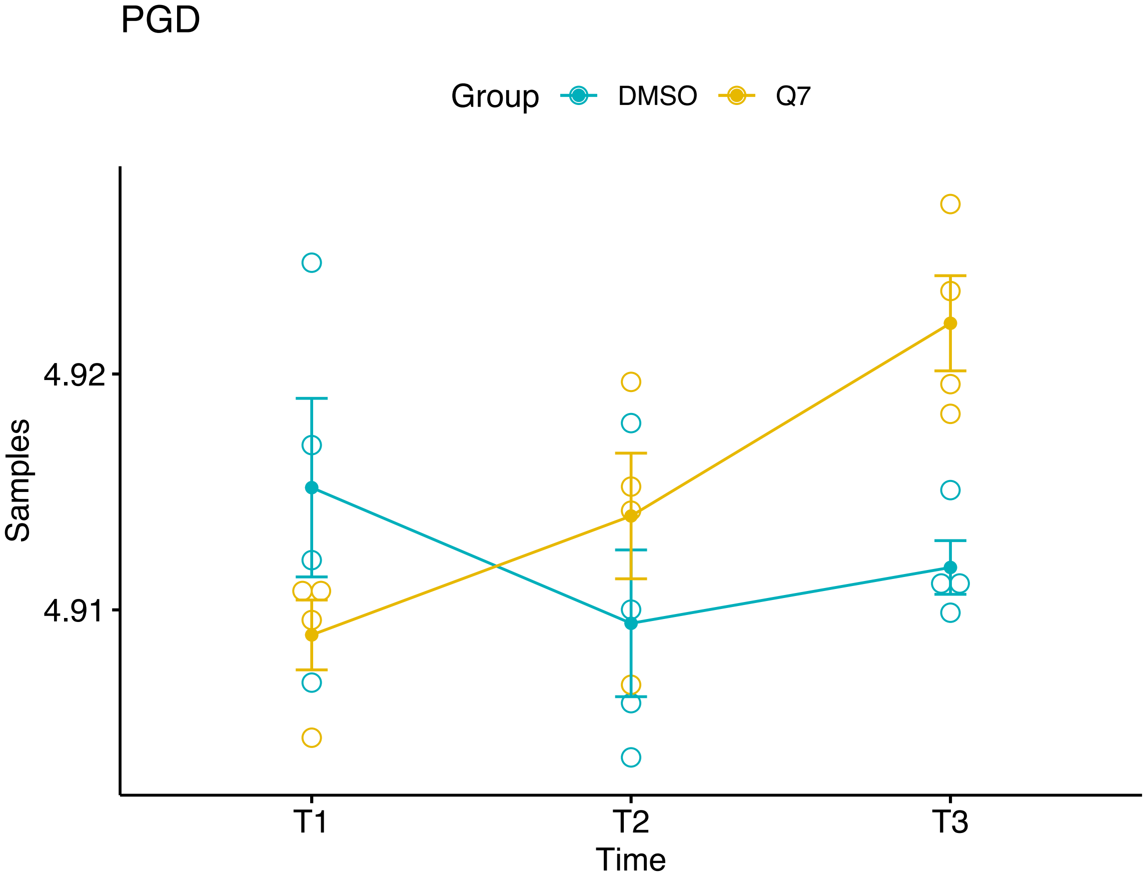
PGD
Group
DMSO
Q7
Samples
4.92
4.91
T1
T2
T3
Time

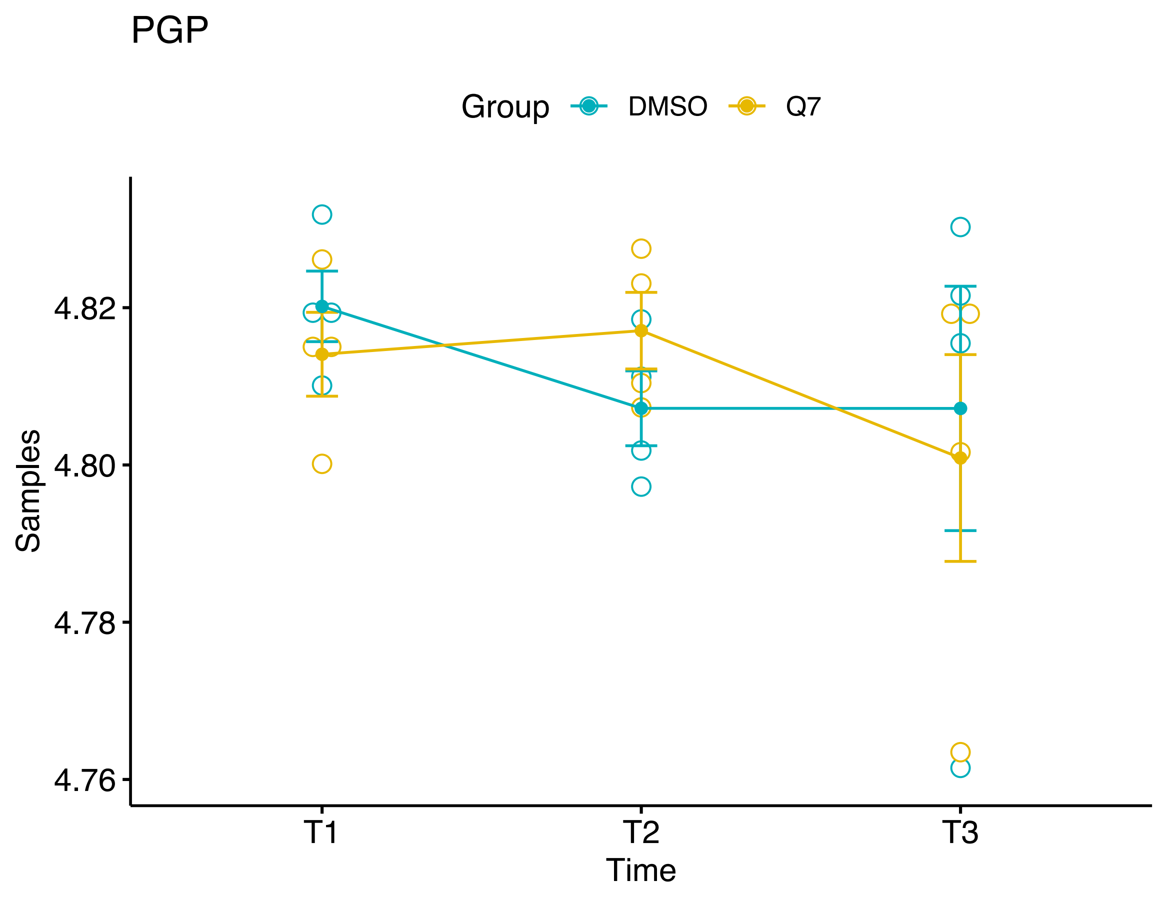
PGP
Group
DMSO
Q7
Samples
4.82
4.80
4.78
4.76
T1
T2
T3
Time

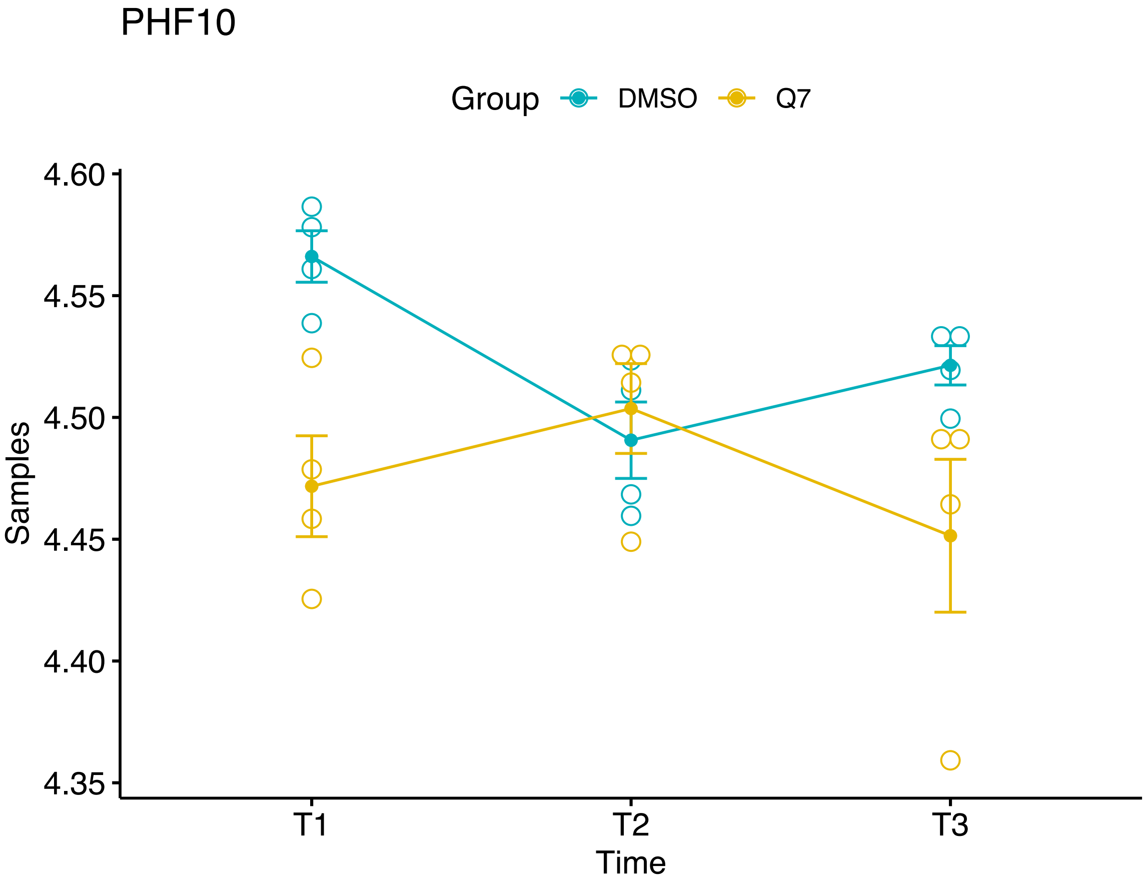

PHF10
Group
DMSO
Q7
4.60
4.55
4.50
4.45
4.40
4.35
Samples
T1
T2
T3
Time

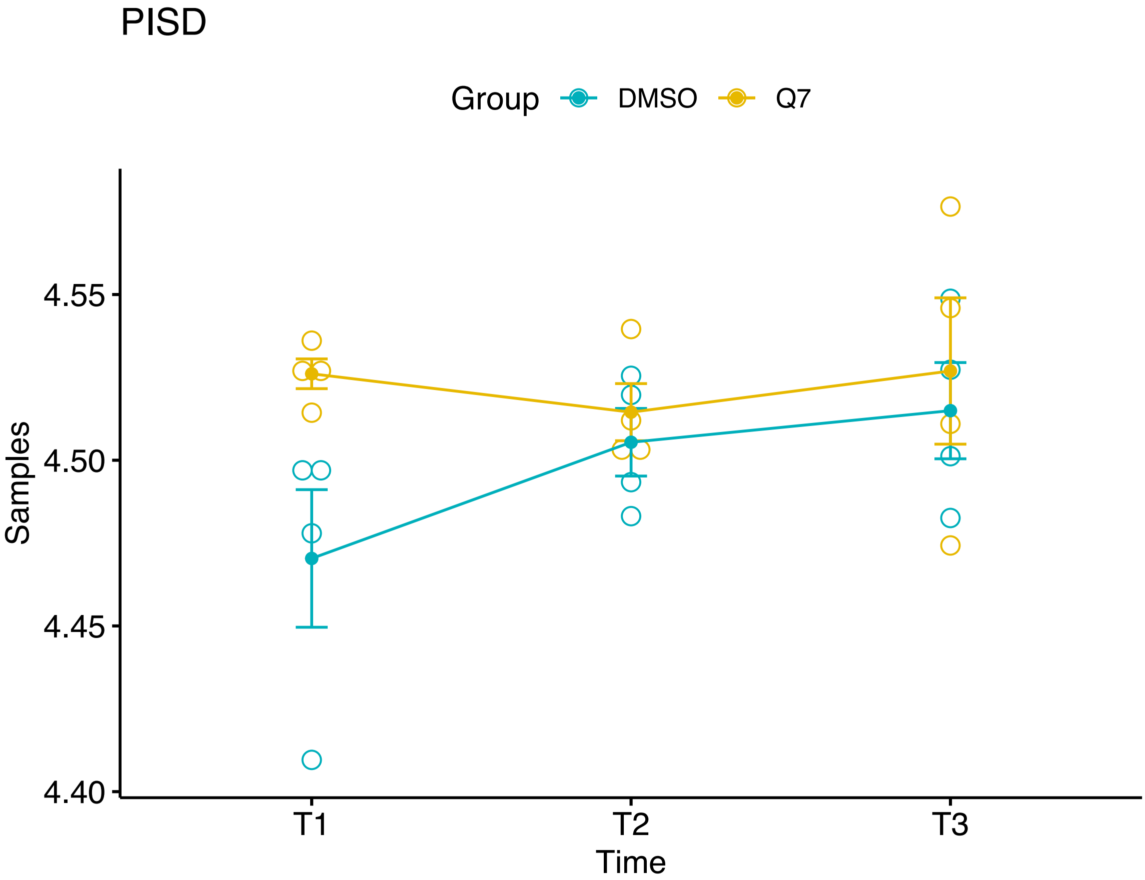
PISD
Group
DMSO
Q7
Samples
4.55
4.50
4.45
4.40
T1
T2
T3
Time

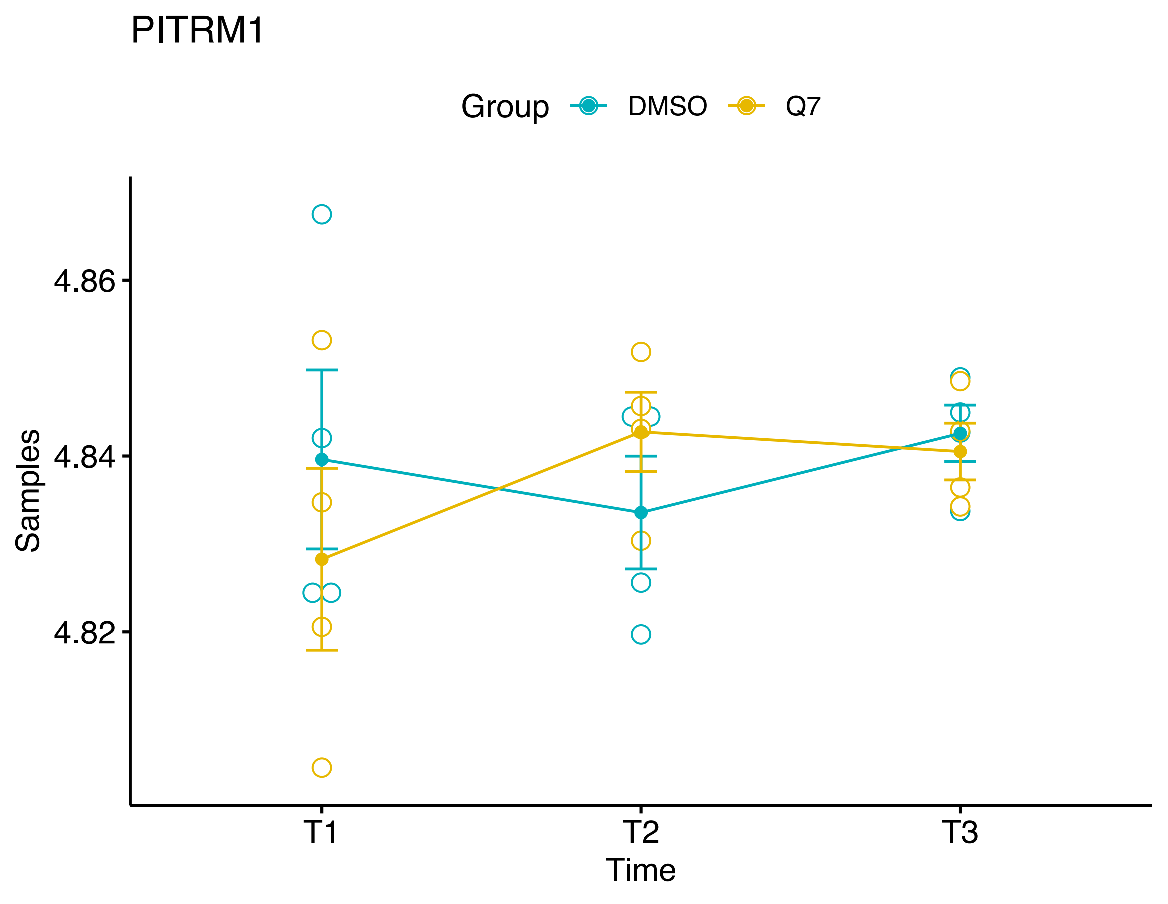

PITRM1
Group
DMSO
Q7
Samples
4.86
4.84
4.82
T1
T2
T3
Time

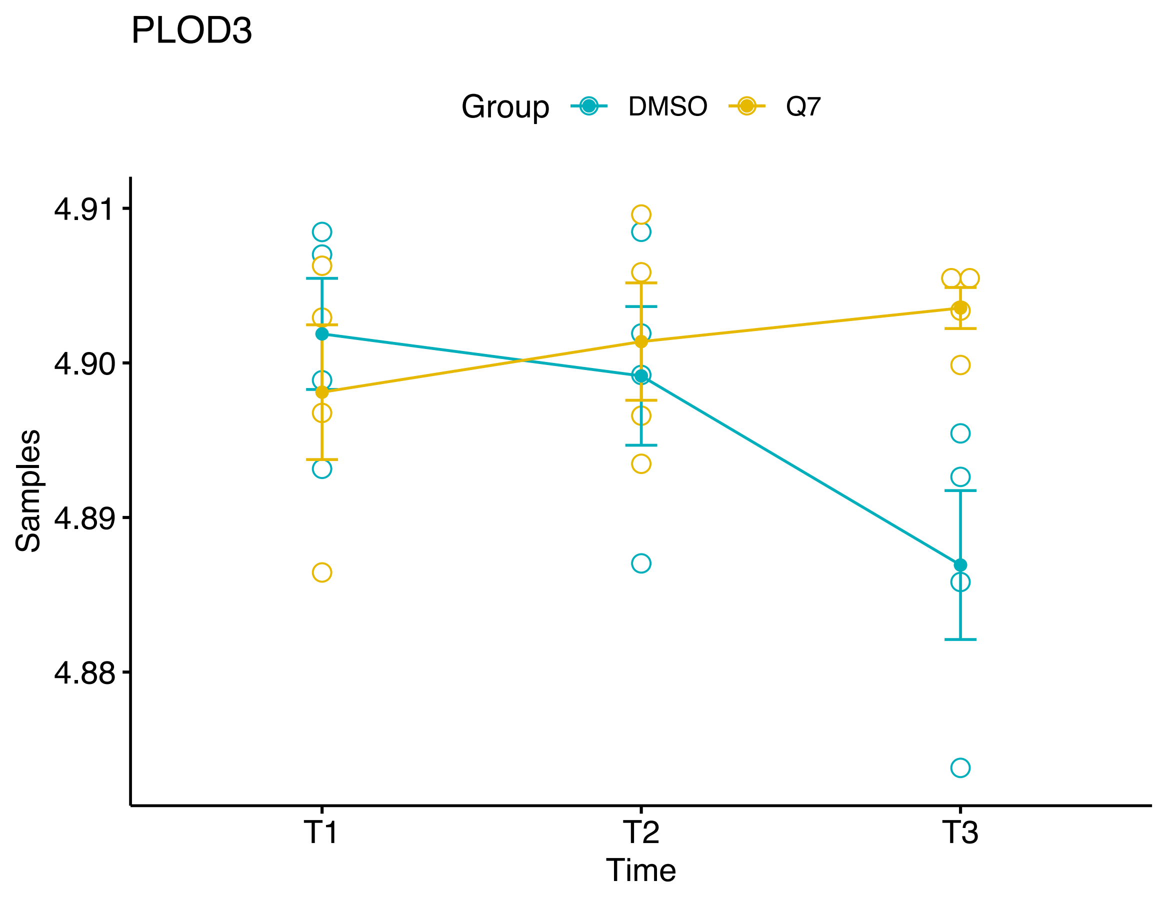
PLOD3
Group
DMSO
Q7
4.91
4.90
4.89
4.88
Samples
T1
T2
T3
Time

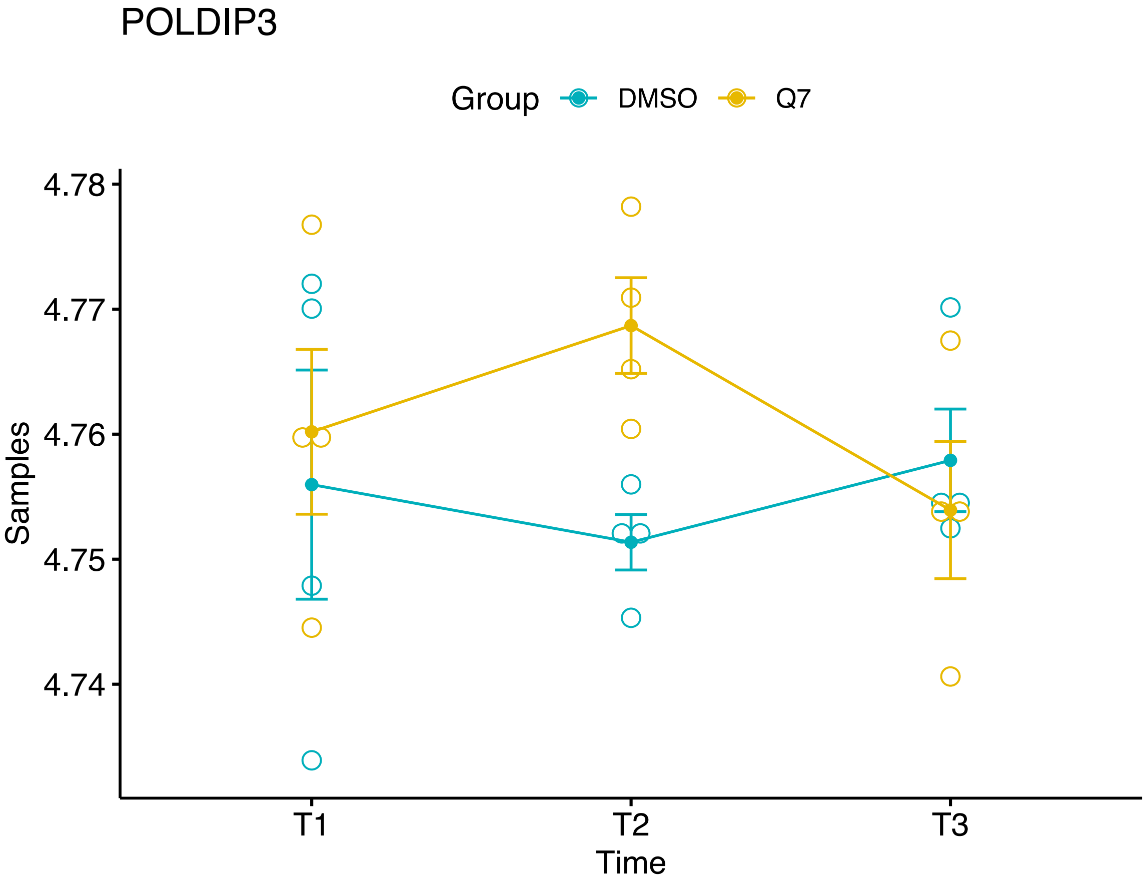

POLDIP3
Group
DMSO
Q7
Samples
4.78
4.77
4.76
4.75
4.74
T1
T2
T3
Time

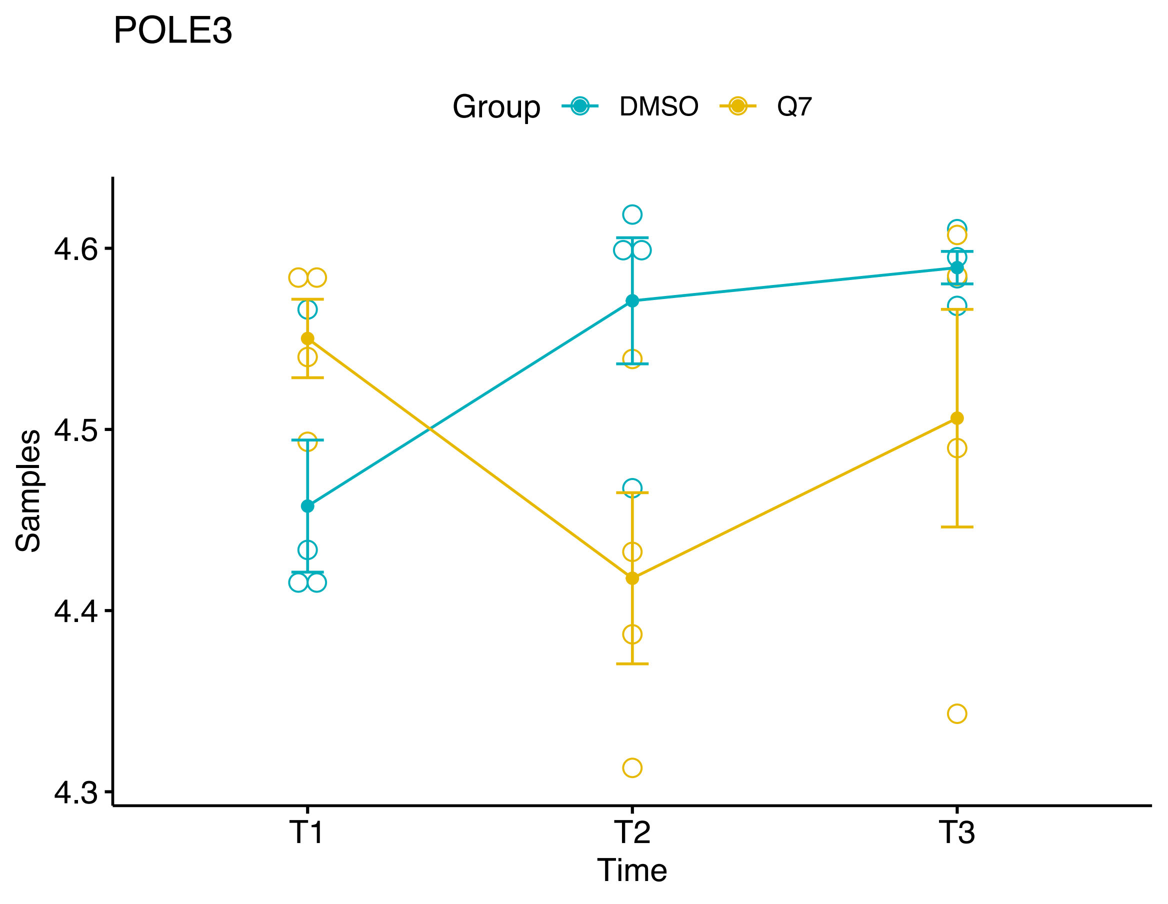

POLE3
Group
DMSO
Q7
Samples
4.6
4.5
4.4
4.3
T1
T2
T3
Time

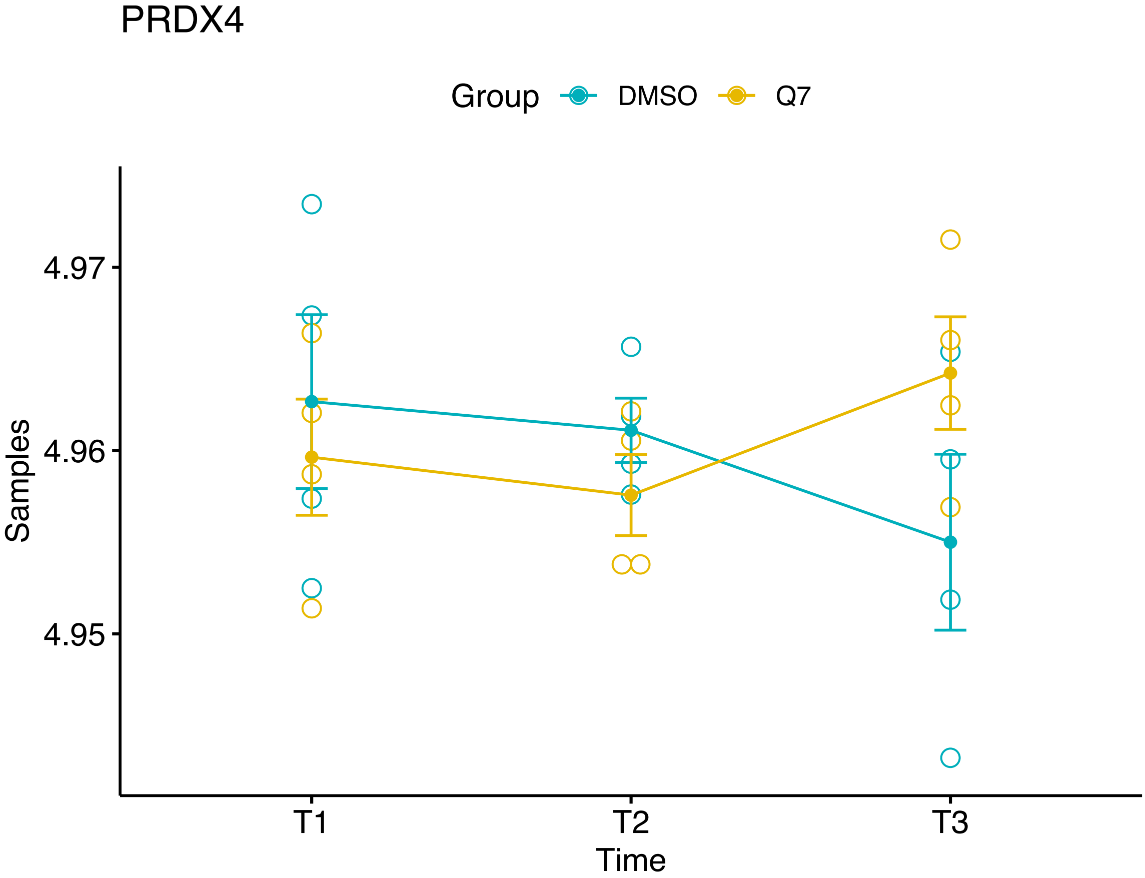
PRDX4
Group
DMSO
Q7
4.97
4.96
4.95
Samples
T1
T2
T3
Time

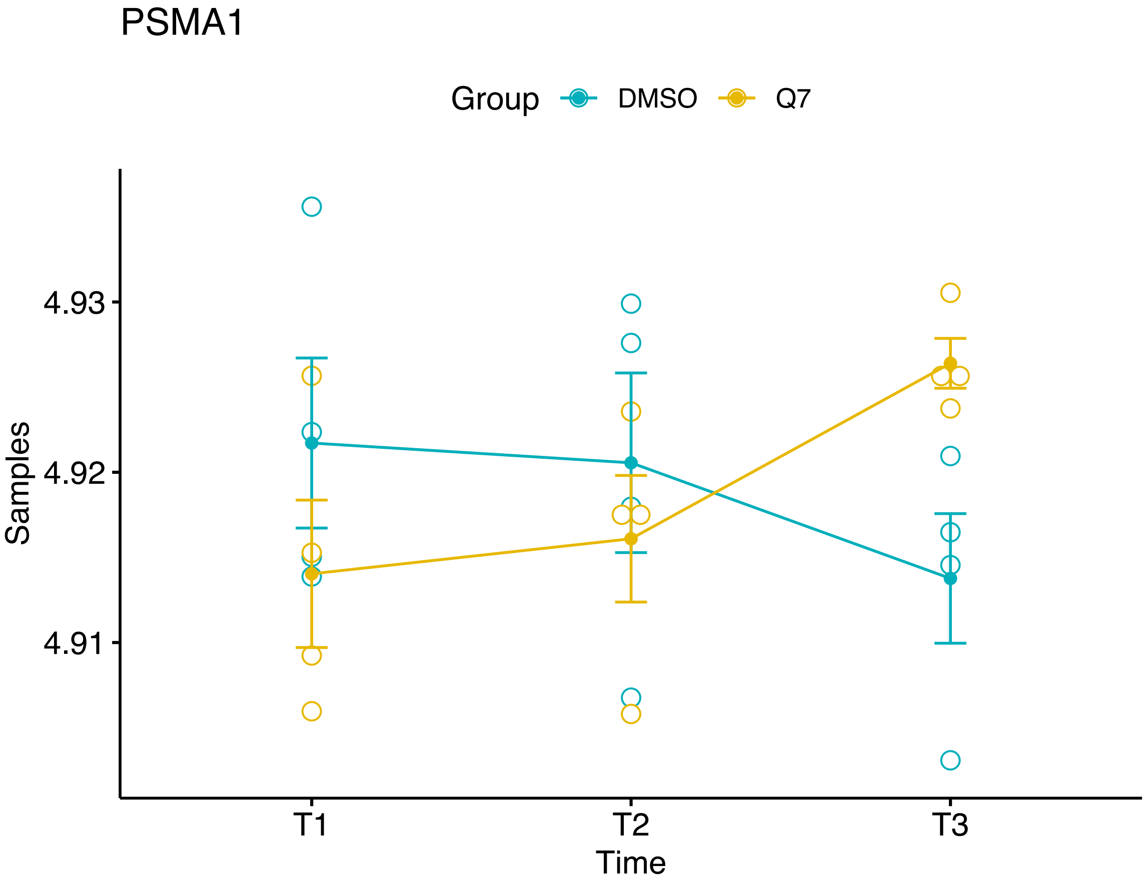

PSMA1
Group
DMSO
Q7
Samples
4.93
4.92
4.91
T1
T2
T3
Time

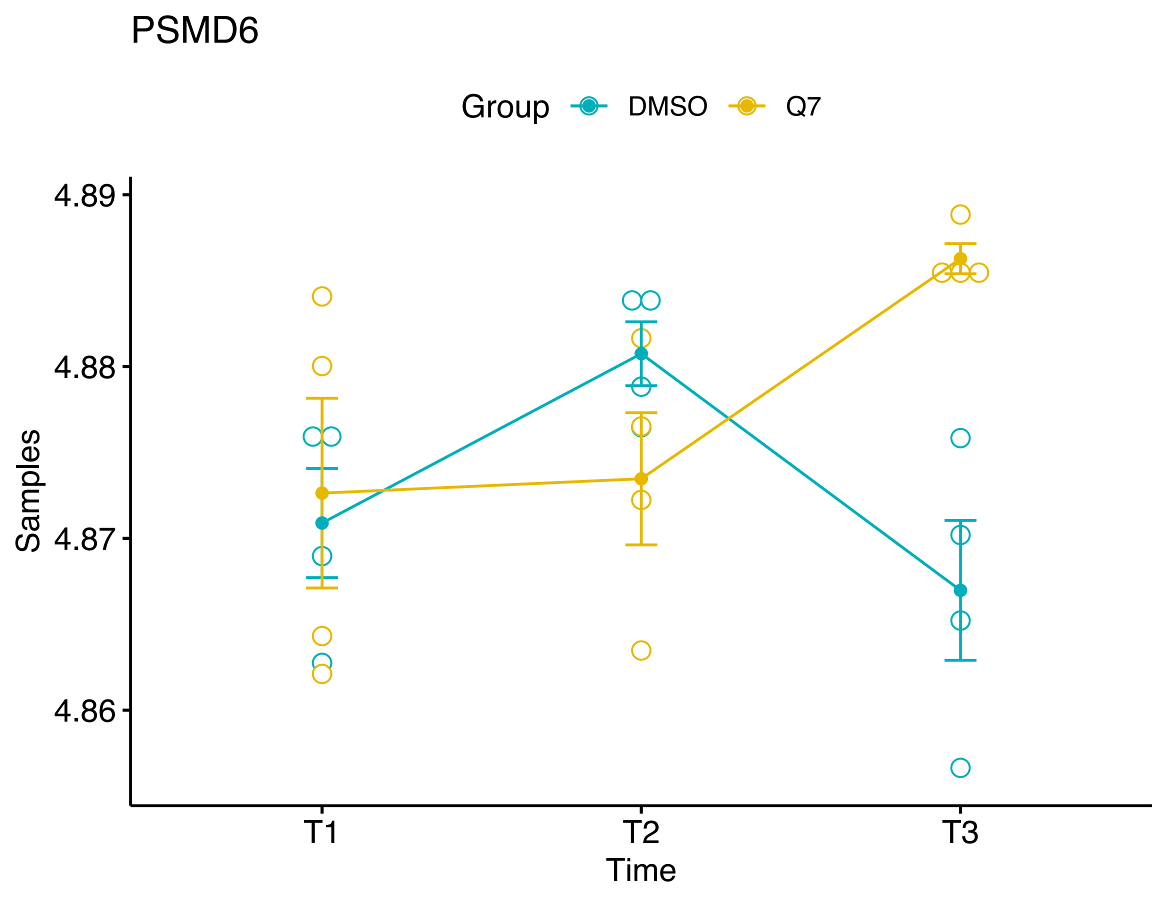
PSMD6
Group
DMSO
Q7
4.89
4.88
4.87
4.86
Samples
T1
T2
T3
Time

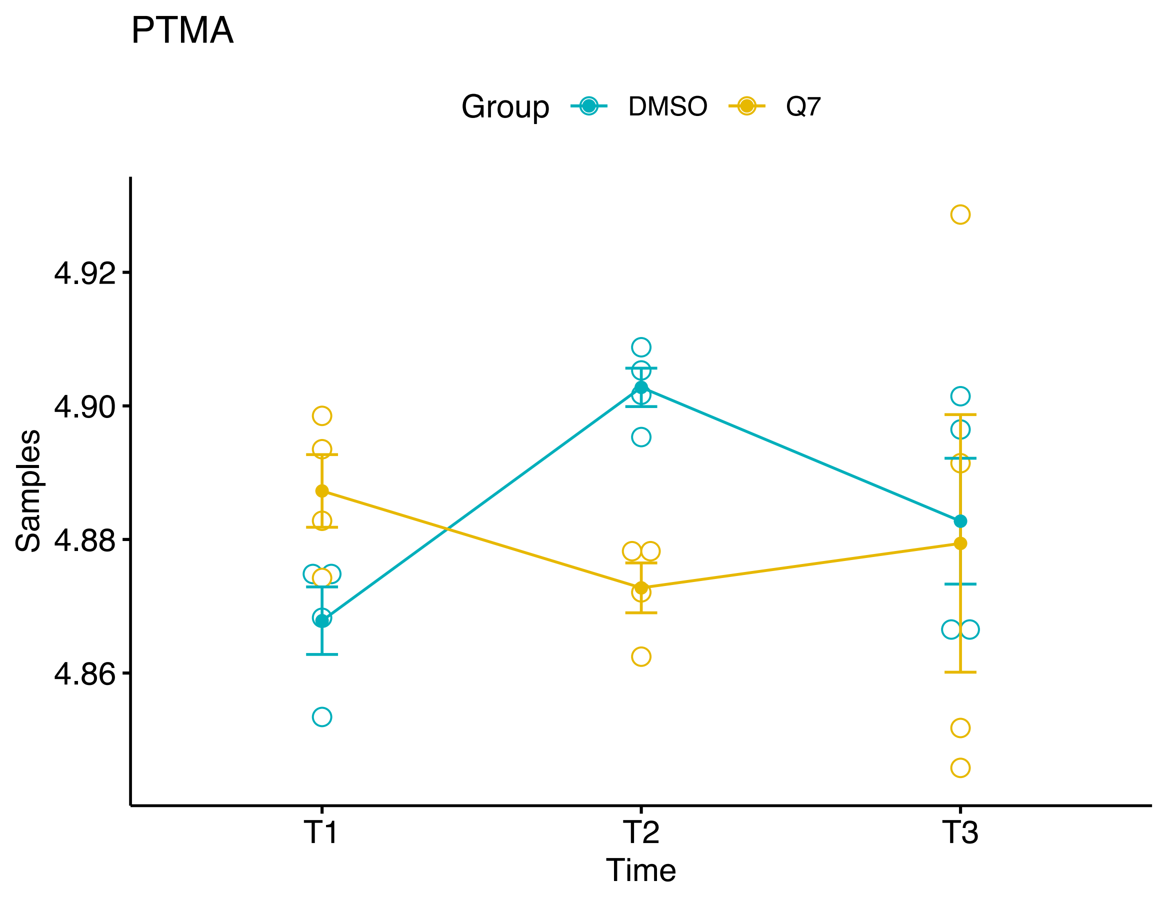

PTMA
Group
DMSO
Q7
Samples
4.92
4.90
4.88
4.86
T1
T2
T3
Time

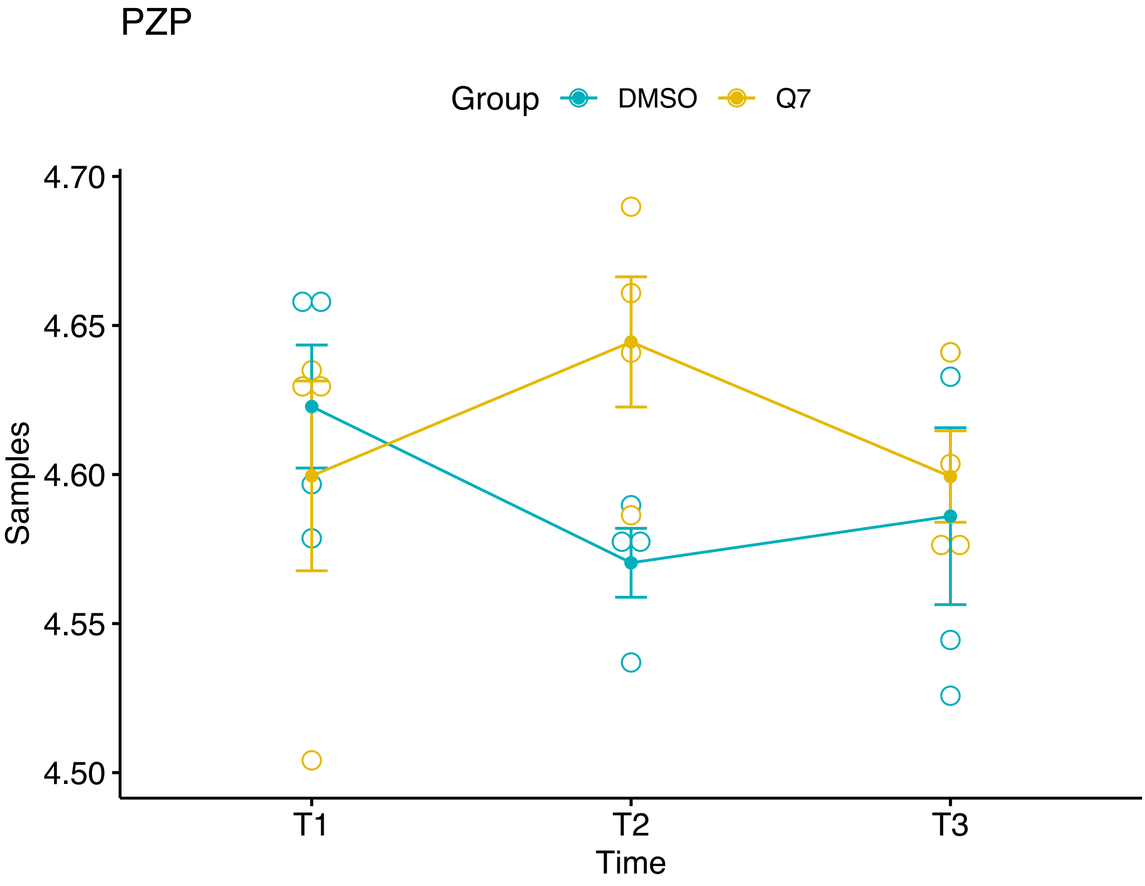
PZP
Group
DMSO
Q7
Samples
4.70
4.65
4.60
4.55
4.50
T1
T2
T3
Time

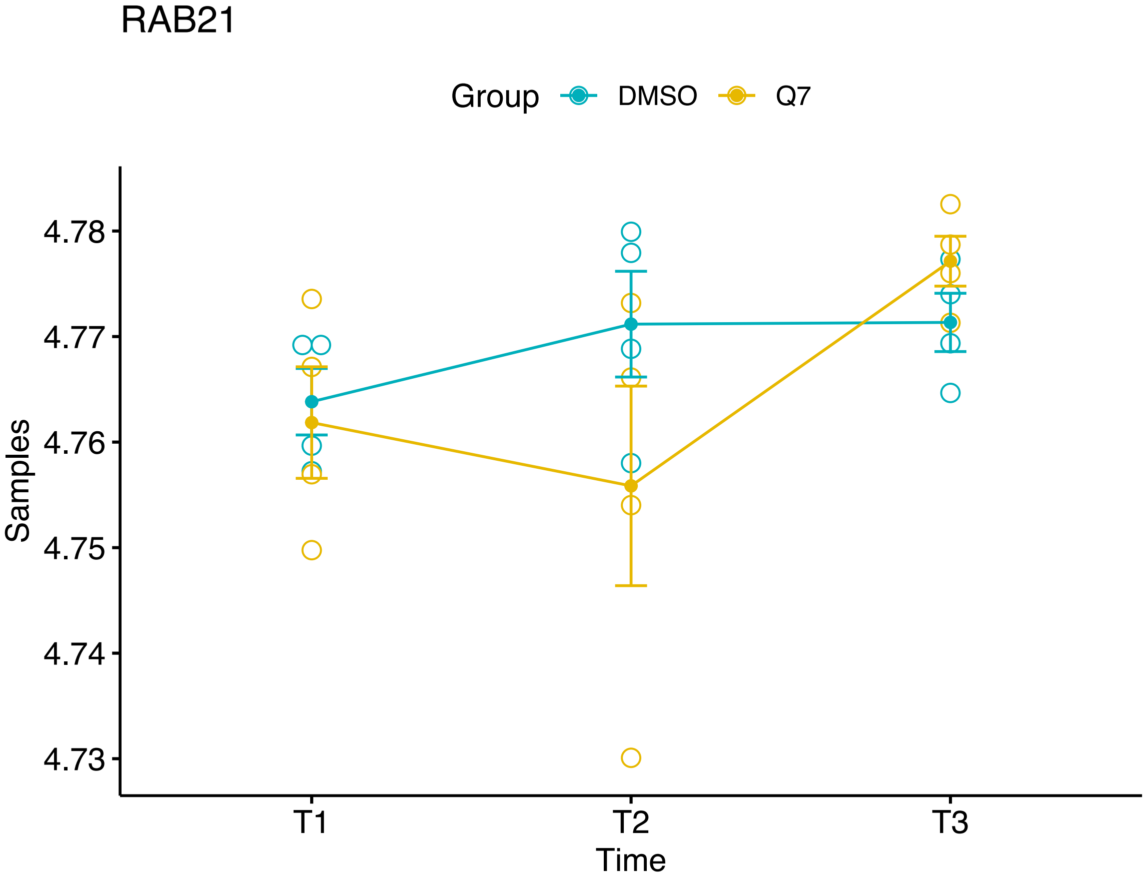
RAB21
Group
DMSO
Q7
4.78
4.77
4.76
4.75
4.74
4.73
Samples
T1
T2
T3
Time

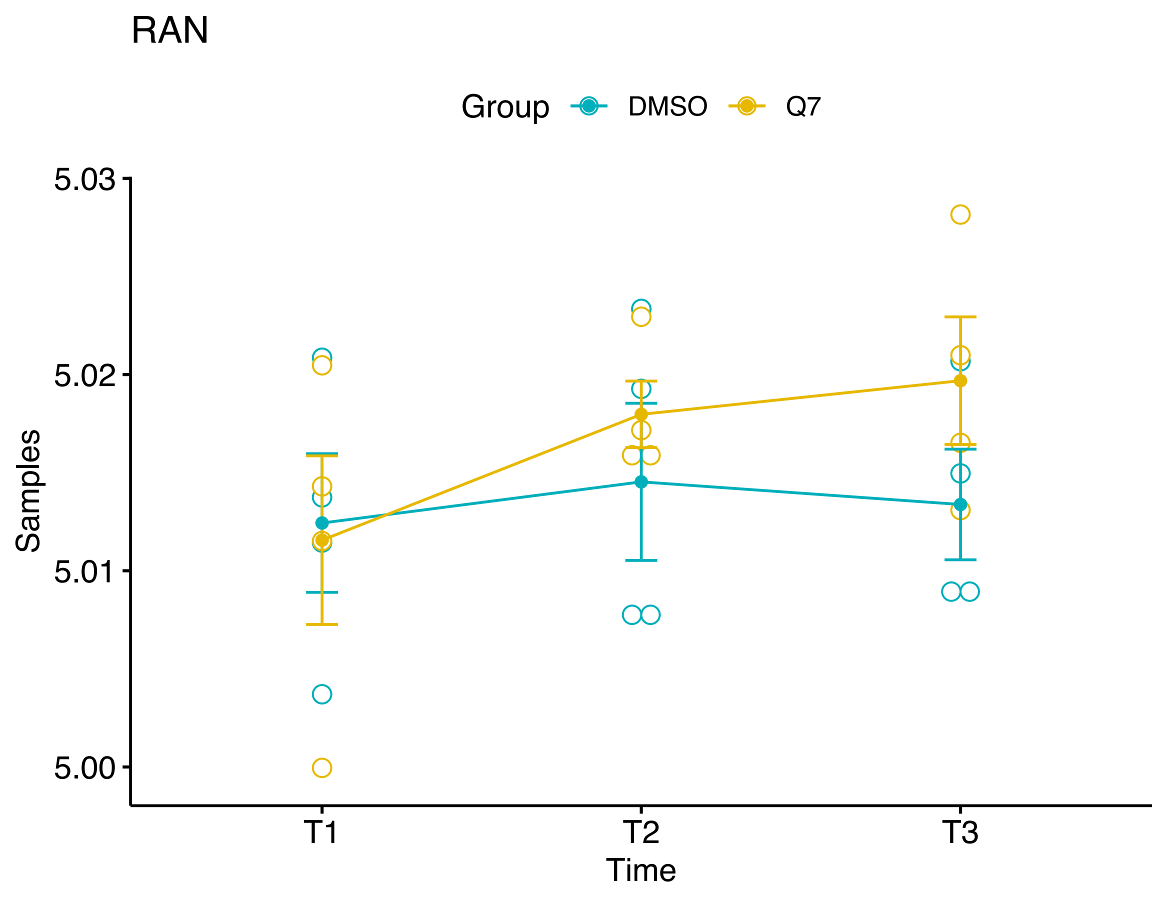

RAN
Group
DMSO
Q7
5.03
5.02
5.01
5.00
Samples
T1
T2
T3
Time

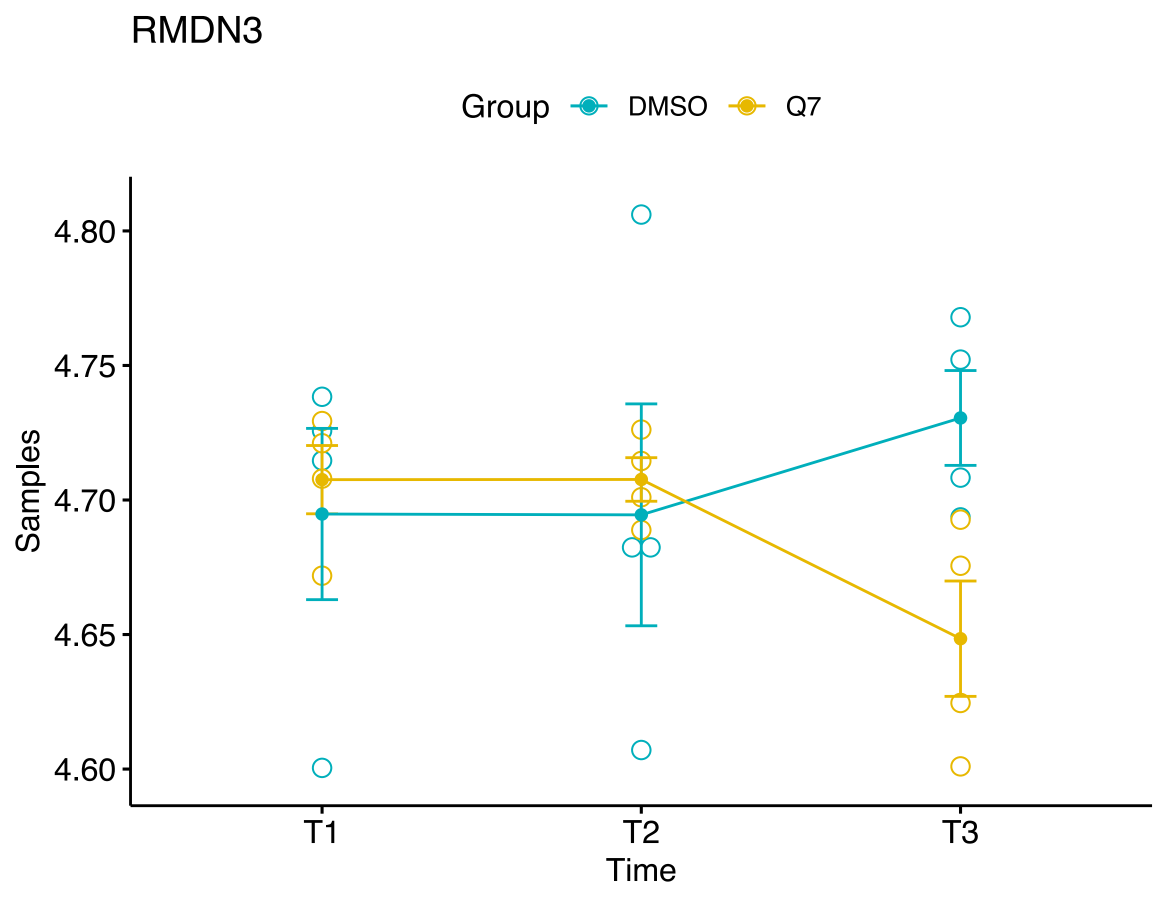

RMDN3
Group
DMSO
Q7
Samples
4.80
4.75
4.70
4.65
4.60
T1
T2
T3
Time

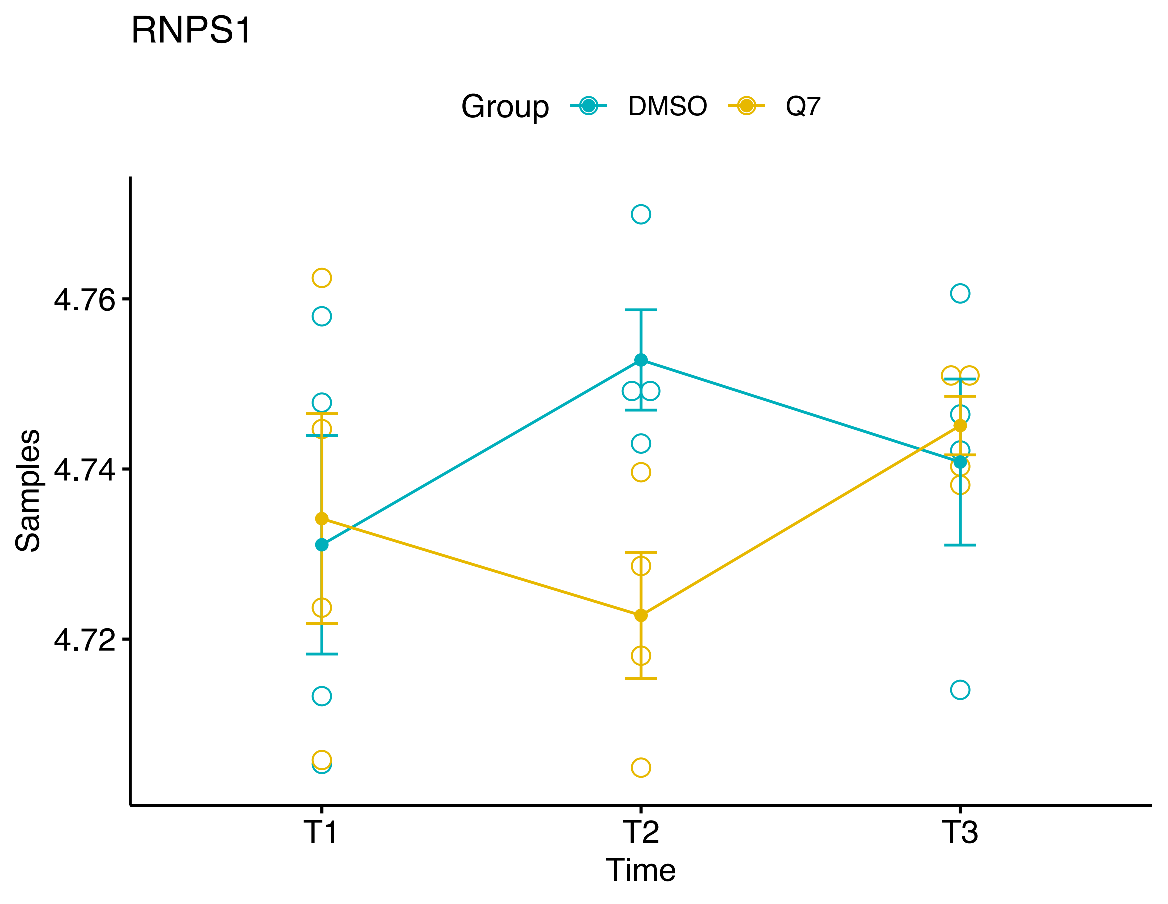
RNPS1
Group
DMSO
Q7
Samples
4.76
4.74
4.72
T1
T2
T3
Time

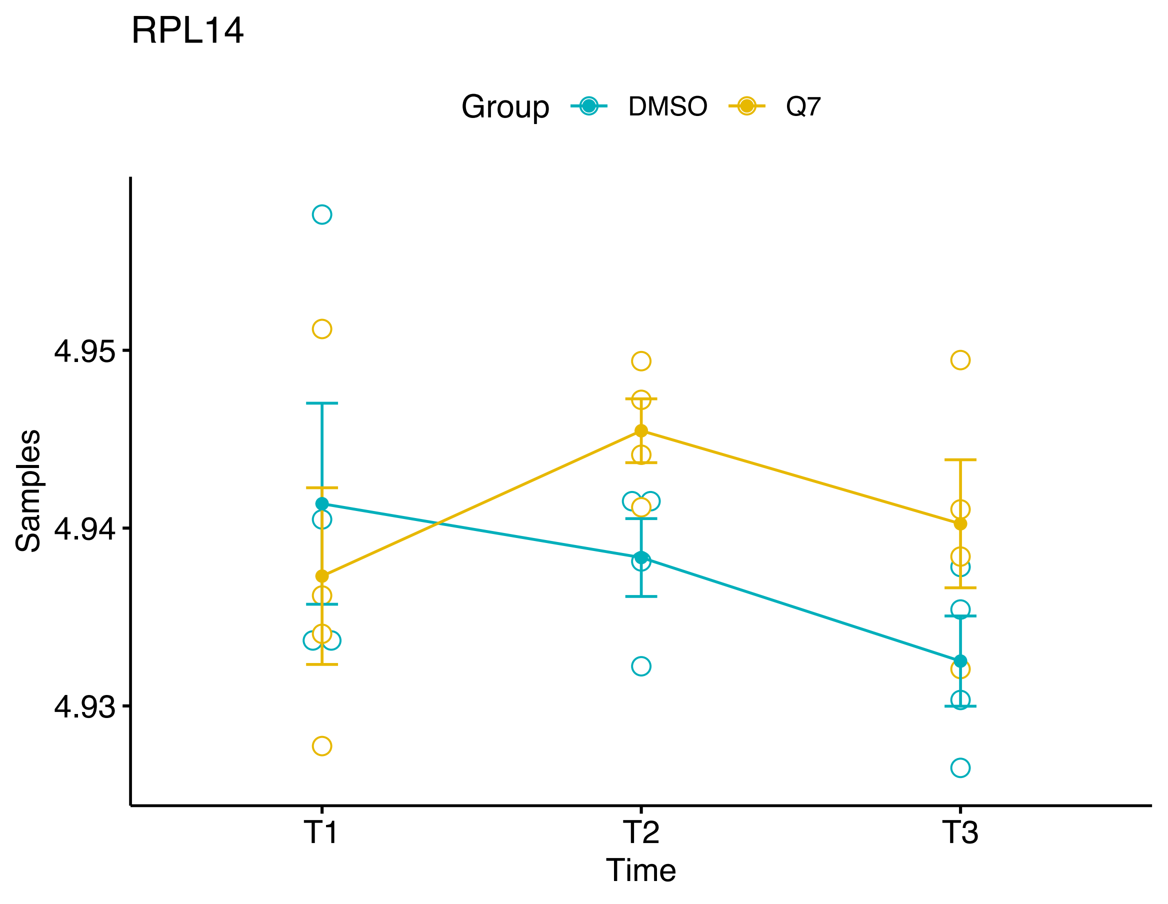

RPL14
Group
DMSO
Q7
Samples
4.95
4.94
4.93
T1
T2
T3
Time

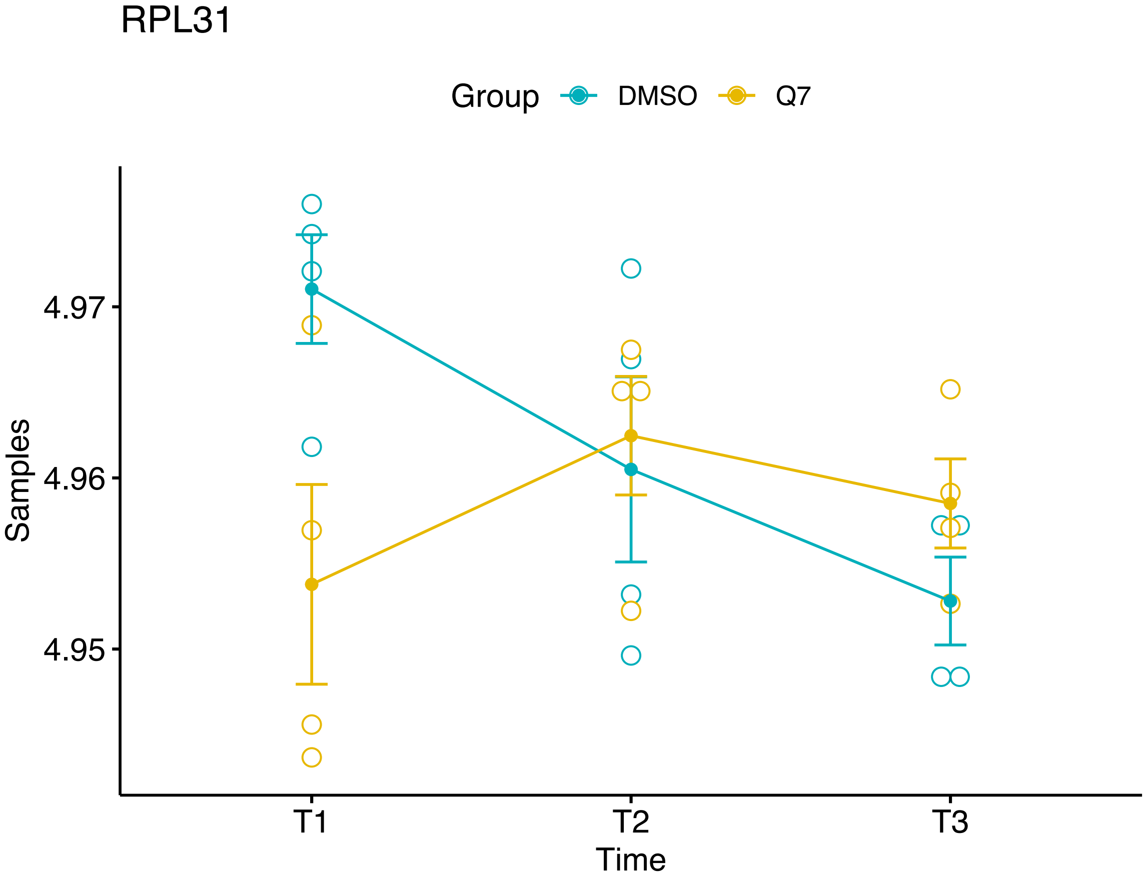

RPL31
Group
DMSO
Q7
4.97
4.96
4.95
Samples
T1
T2
T3
Time

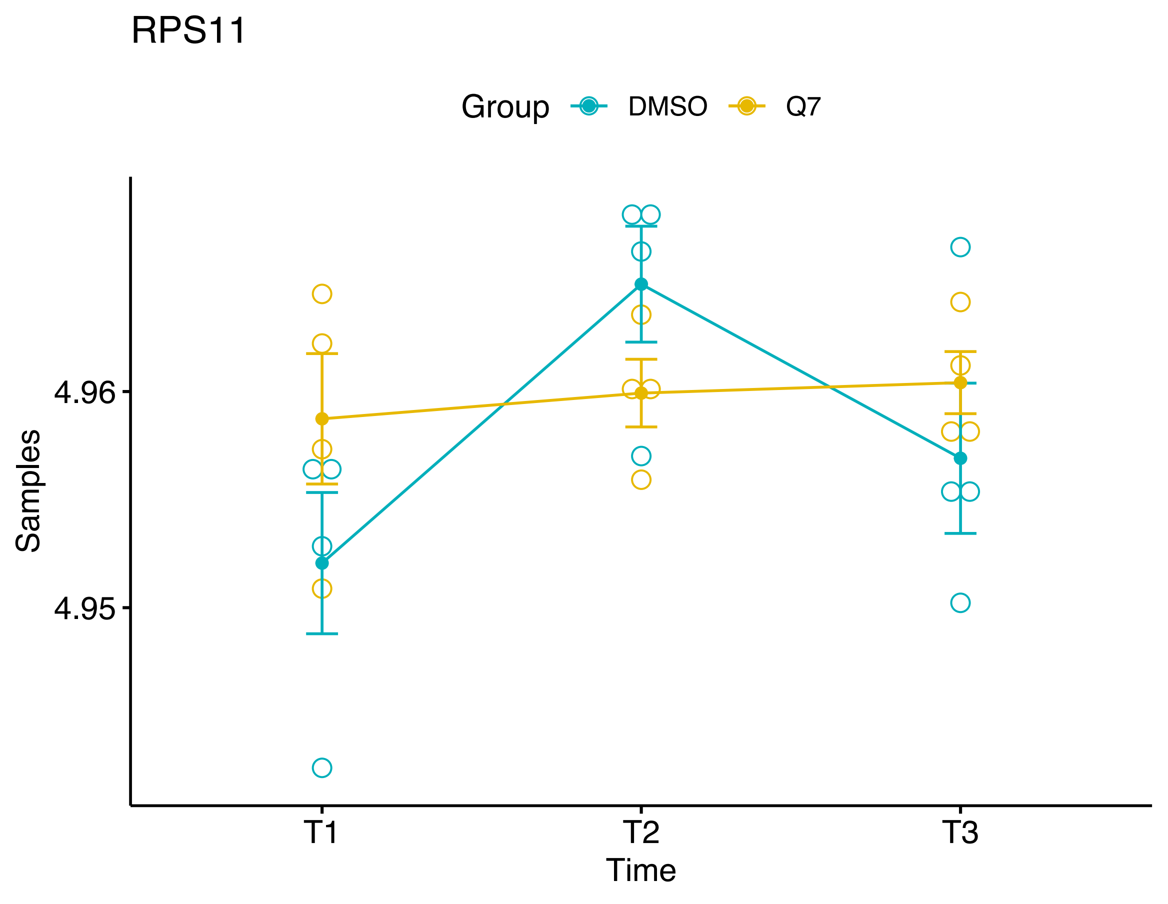
RPS11
Group
DMSO
Q7
4.96
4.95
Samples
T1
T2
T3
Time

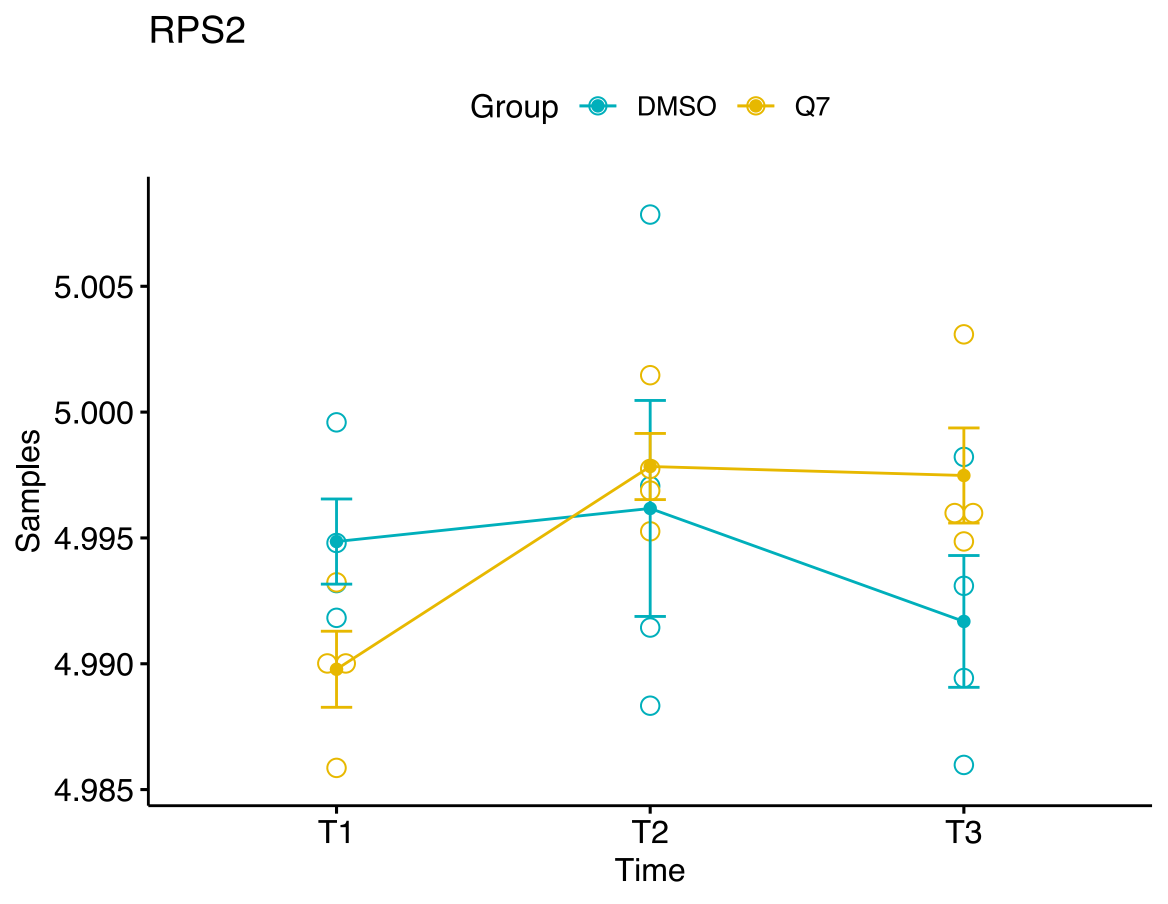
RPS2
Group
DMSO
Q7
Samples
5.005
5.000
4.995
4.990
4.985
T1
T2
T3
Time

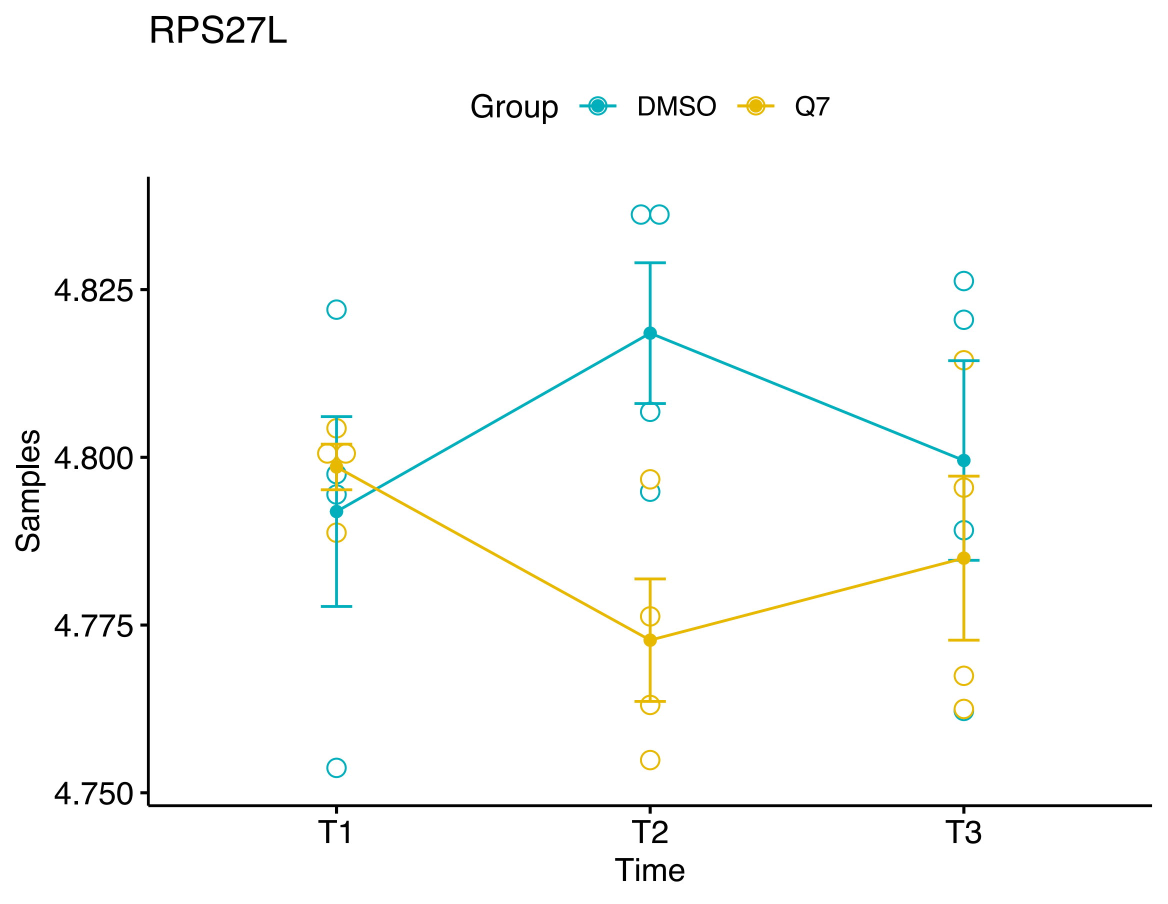

RPS27L
Group
DMSO
Q7
Samples
4.825
4.800
4.775
4.750
T1
T2
T3
Time

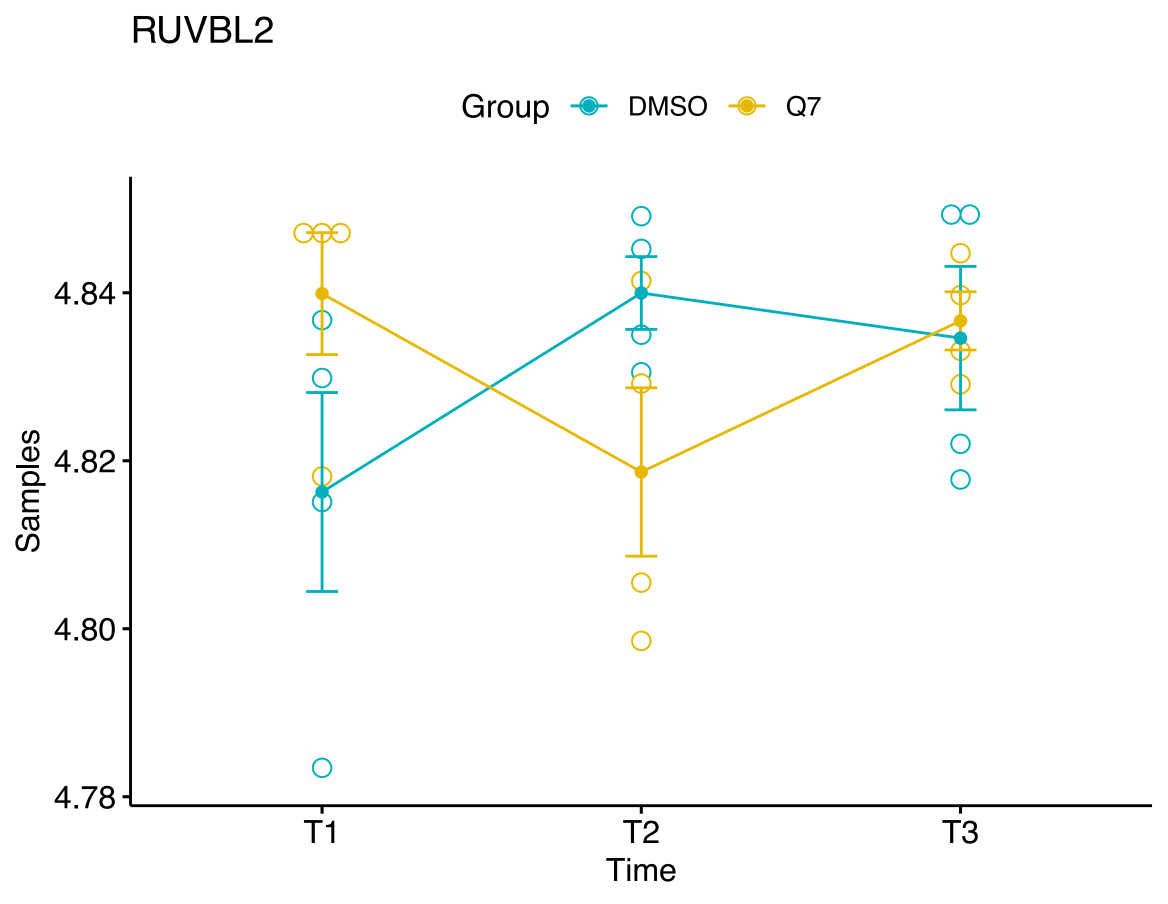
RUVBL2
Group
DMSO
Q7
4.84
4.82
4.80
4.78
Samples
T1
T2
T3
Time

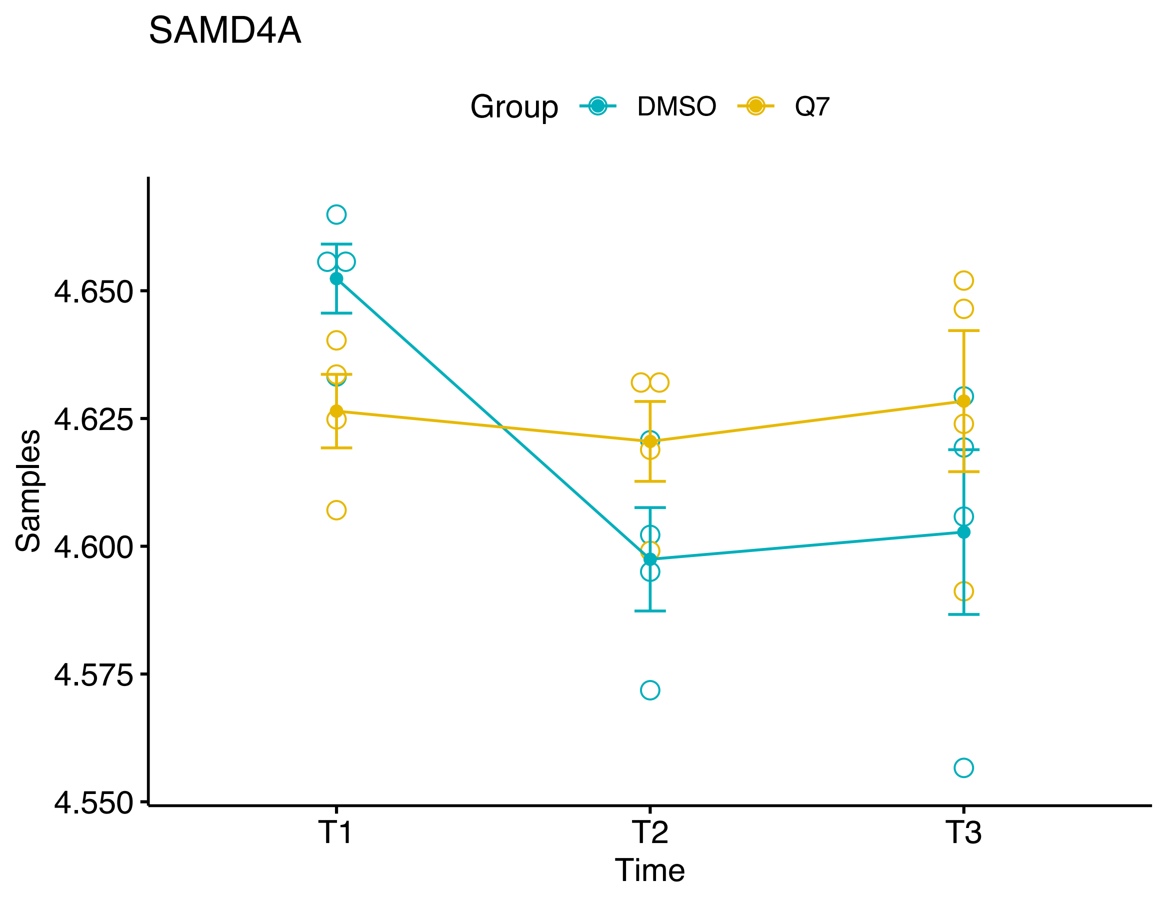

SAMD4A
Group
DMSO
Q7
Samples
4.650
4.625
4.600
4.575
4.550
T1
T2
T3
Time

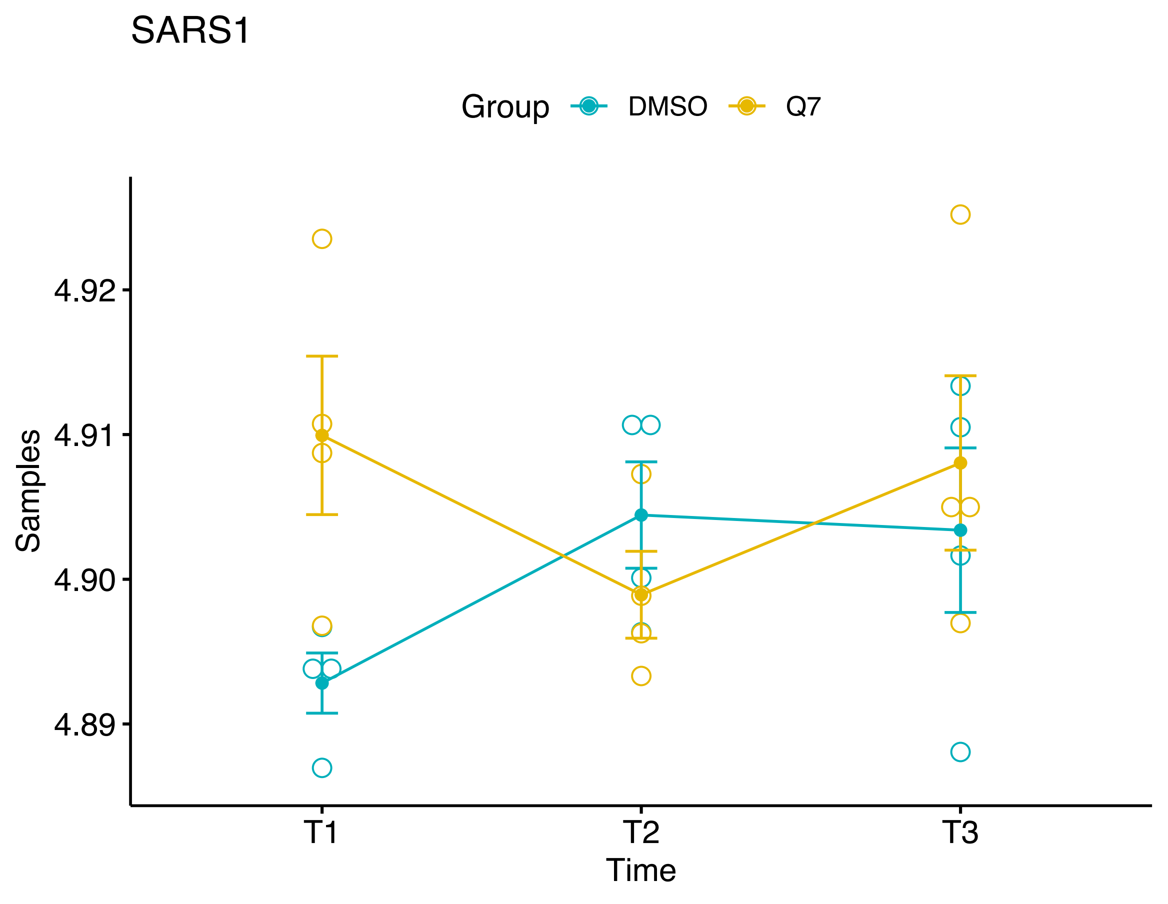
SARS1
Group
DMSO
Q7
Samples
4.92
4.91
4.90
4.89
T1
T2
T3
Time

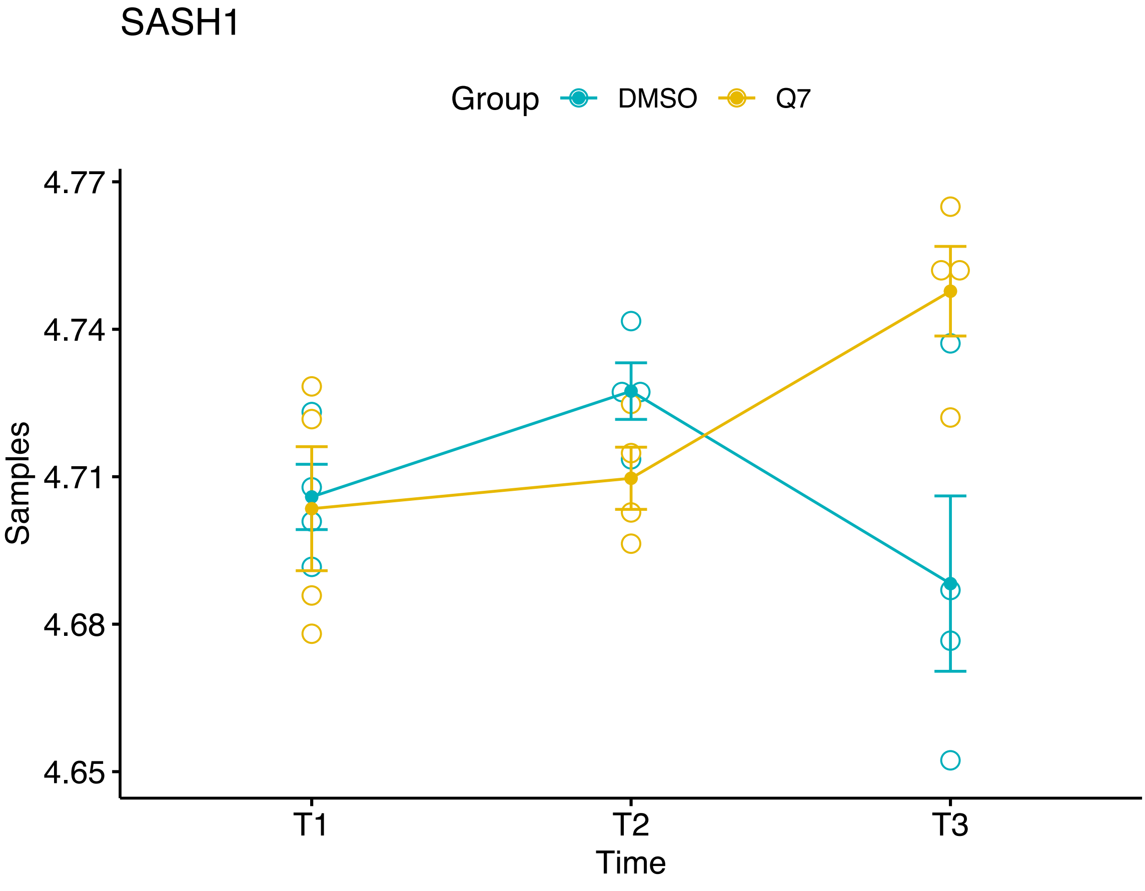
SASH1
Group
DMSO
Q7
4.77
4.74
4.71
4.68
4.65
Samples
T1
T2
T3
Time

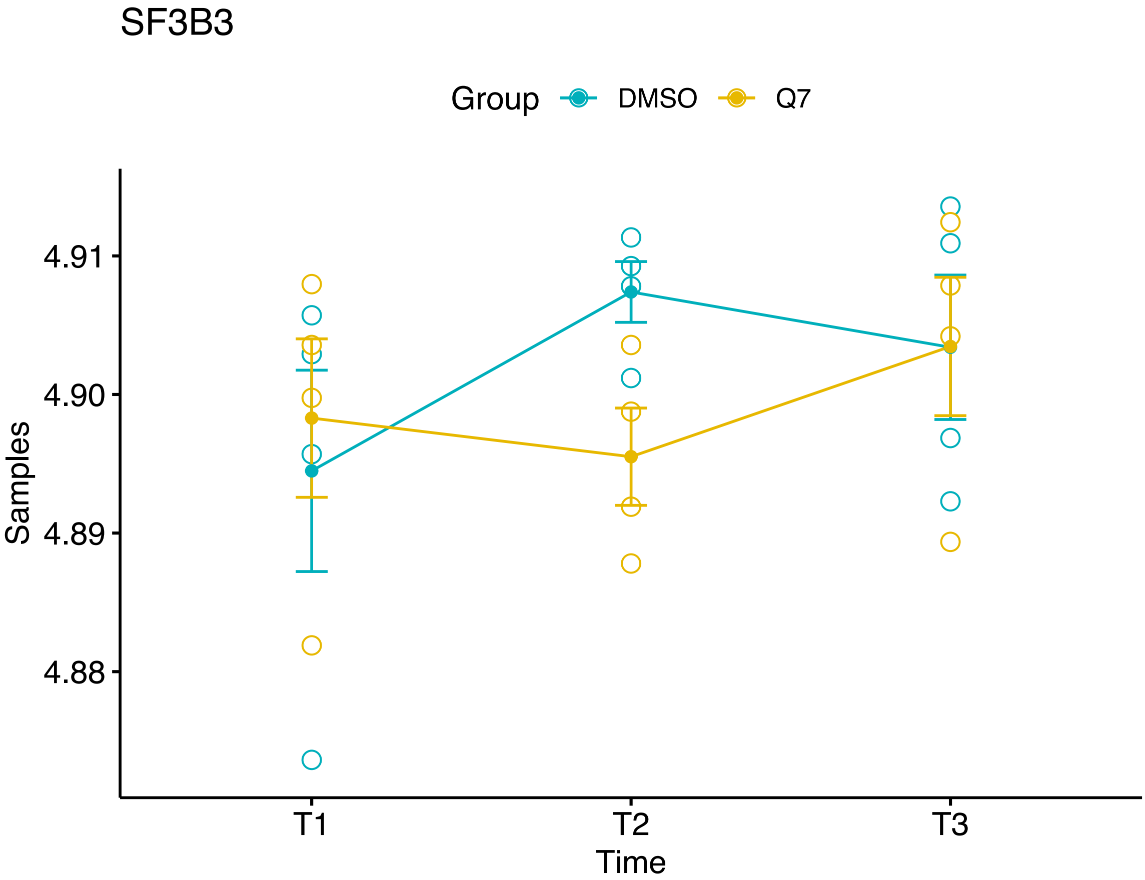
SF3B3
Group
DMSO
Q7
4.91
4.90
4.89
4.88
Samples
T1
T2
T3
Time

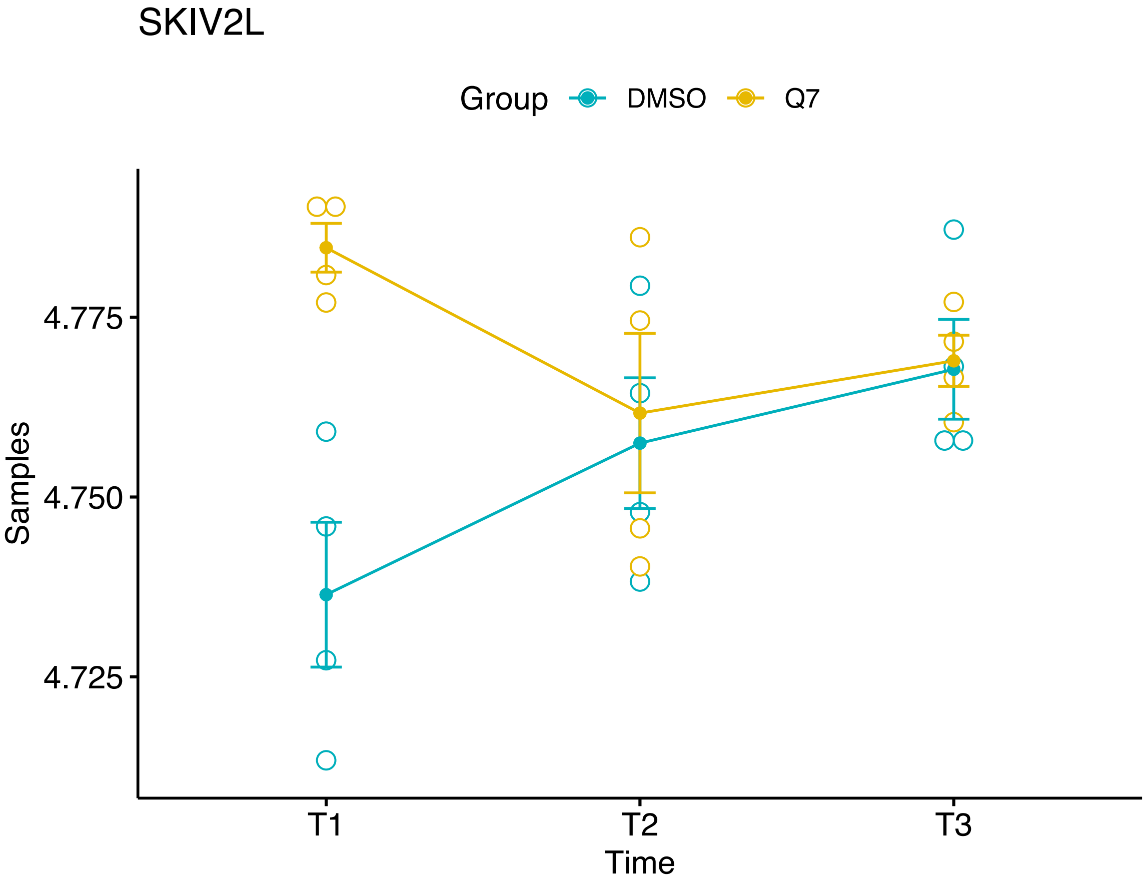

SKIV2L
Group
DMSO
Q7
Samples
4.775
4.750
4.725
T1
T2
T3
Time

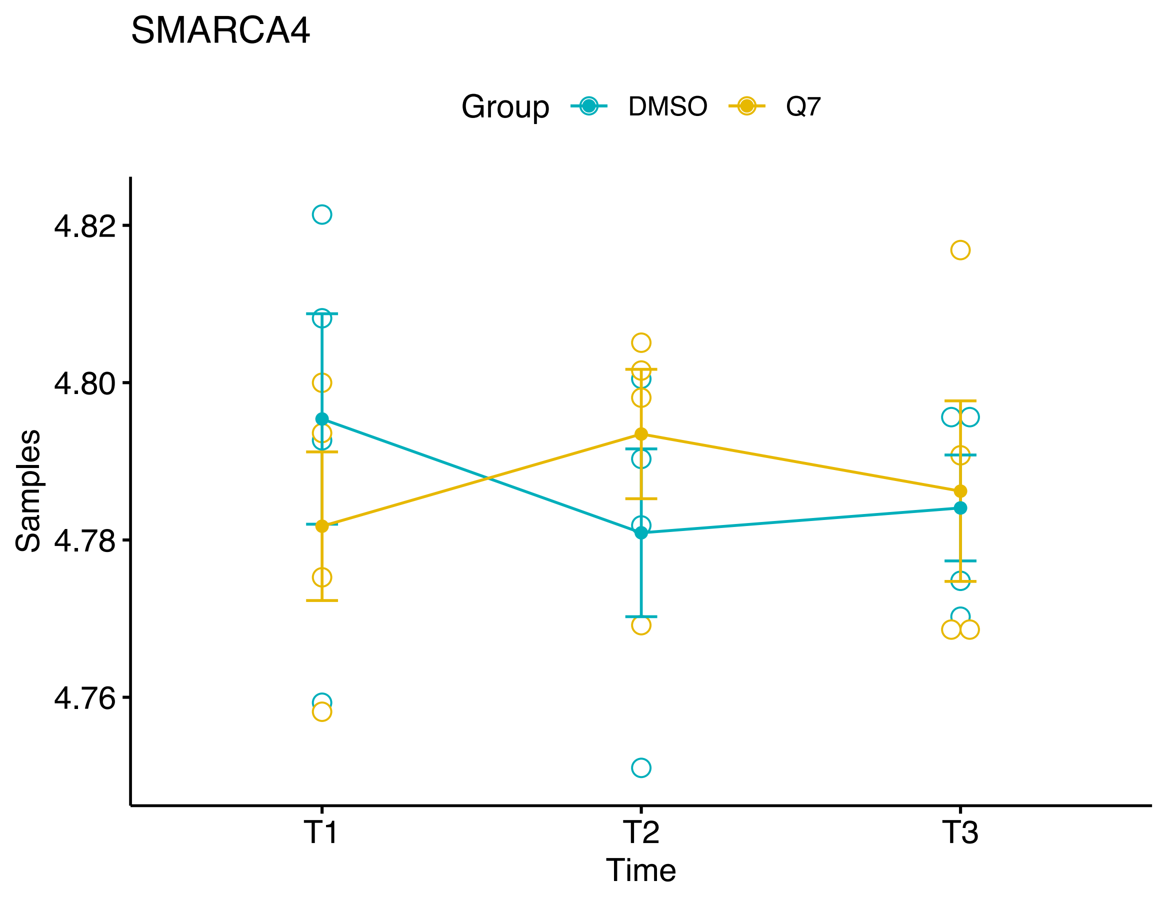
SMARCA4
Group
DMSO
Q7
Samples
4.82
4.80
4.78
4.76
T1
T2
T3
Time

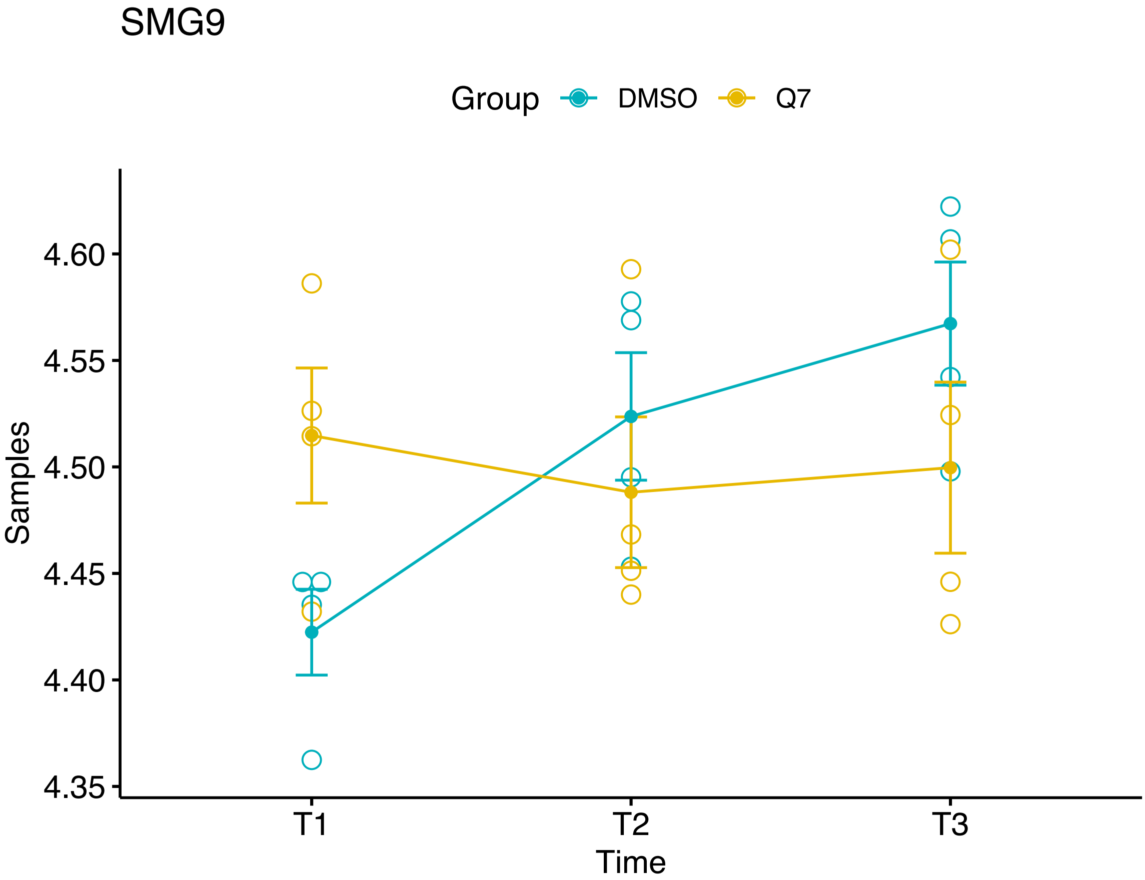

SMG9
Group
DMSO
Q7
Samples
4.60
4.55
4.50
4.45
4.40
4.35
T1
T2
T3
Time

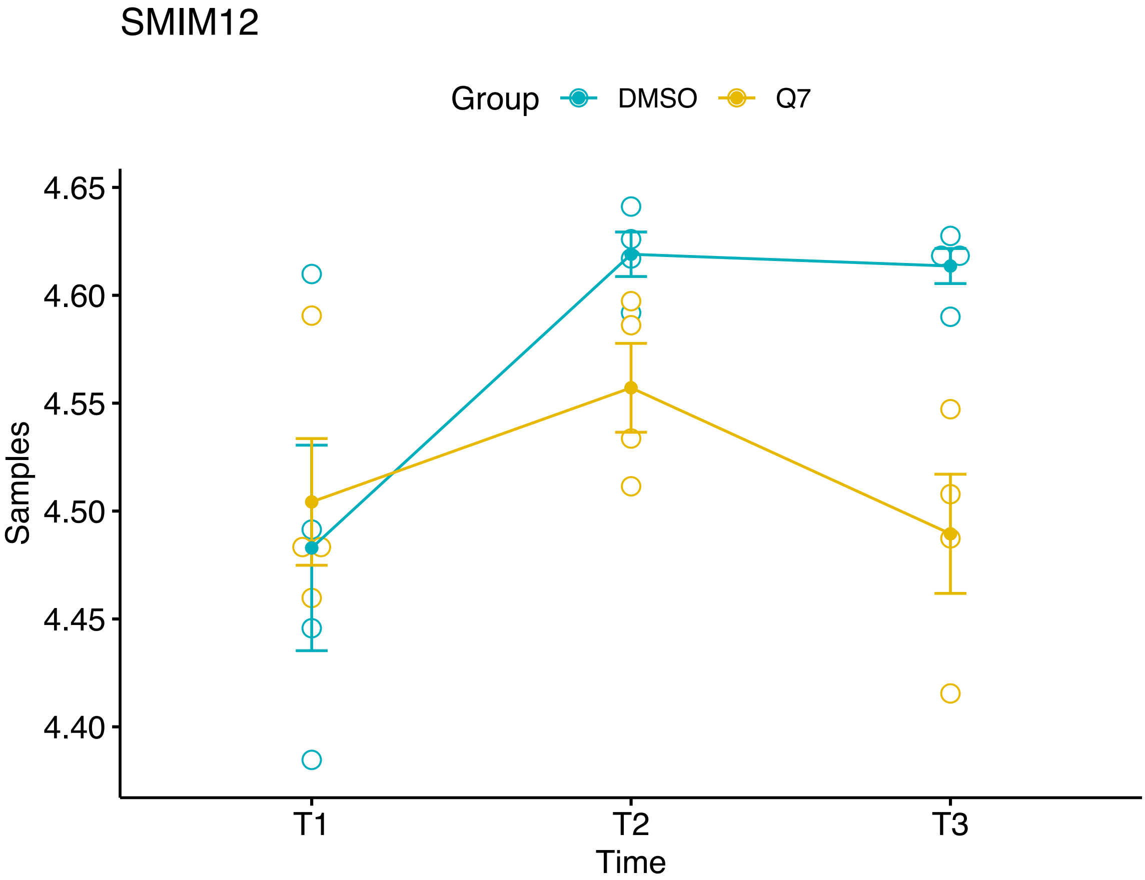

SMIM12
Group
DMSO
Q7
Samples
4.65
4.60
4.55
4.50
4.45
4.40
T1
T2
T3
Time

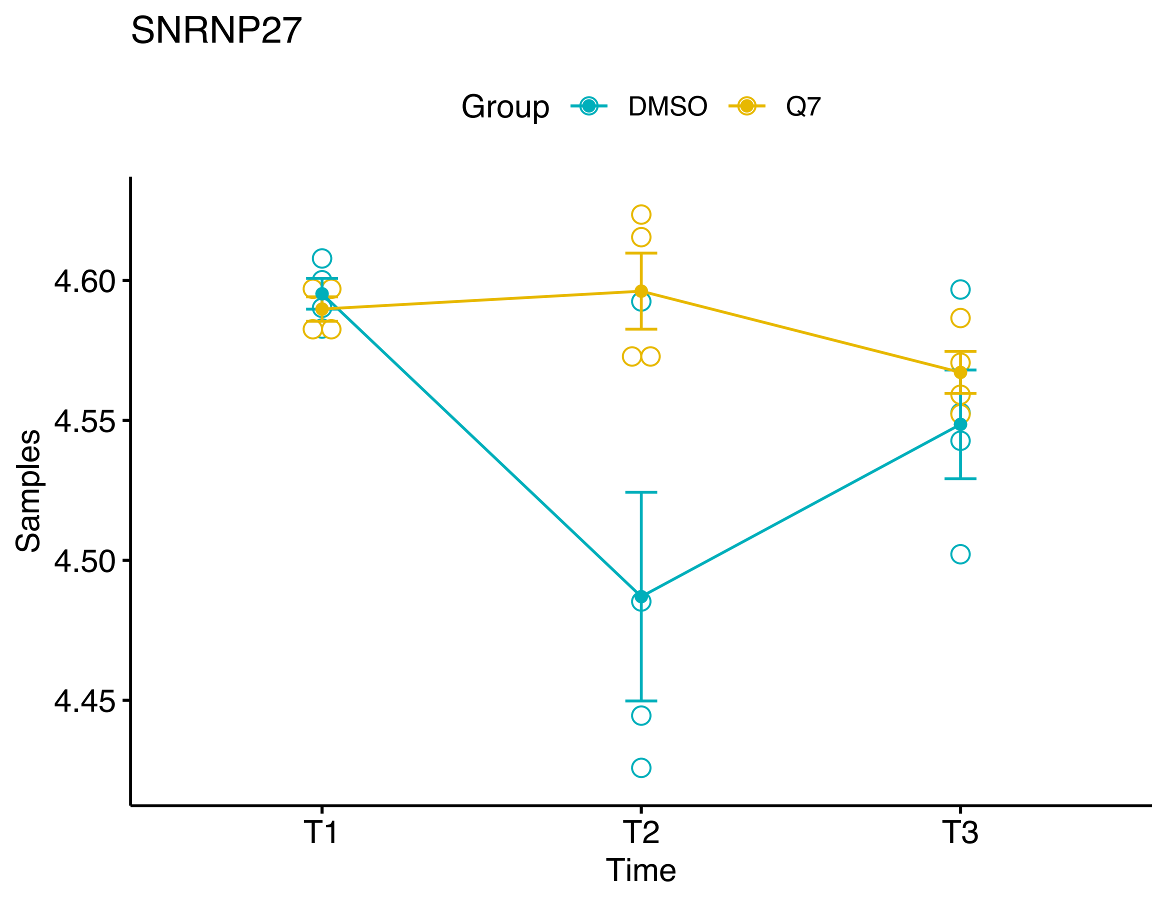

SNRNP27
Group
DMSO
Q7
4.60
4.55
4.50
4.45
Samples
T1
T2
T3
Time

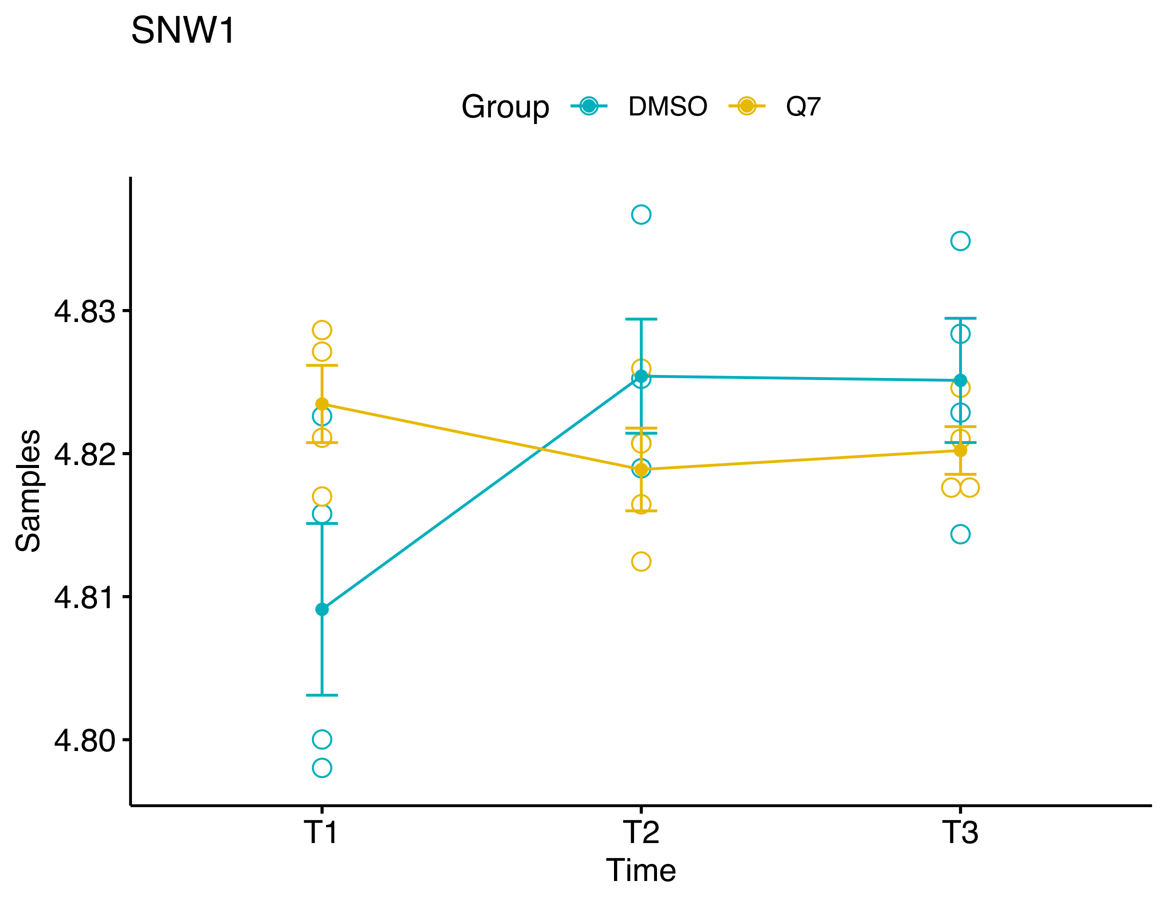

SNW1
Group
DMSO
Q7
Samples
4.83
4.82
4.81
4.80
T1
T2
T3
Time

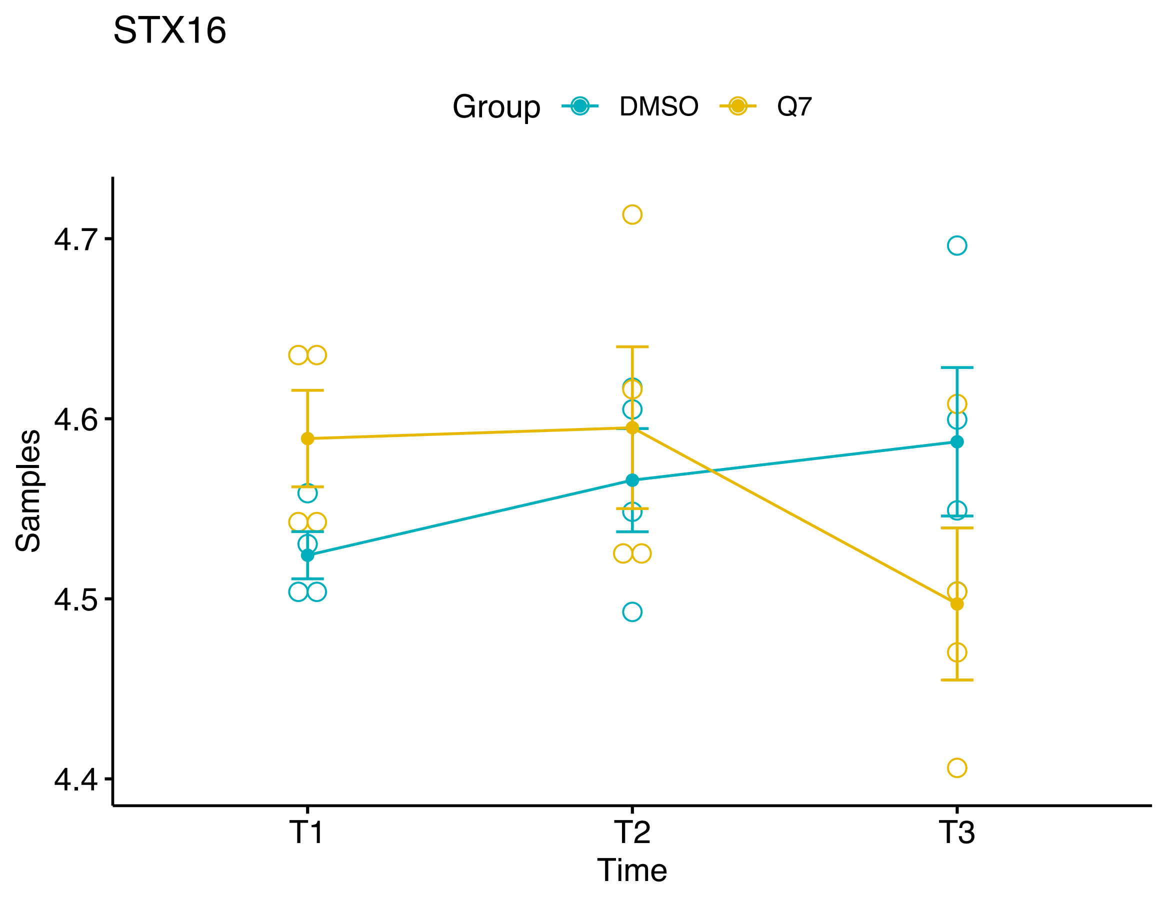
STX16
Group
DMSO
Q7
Samples
4.7
4.6
4.5
4.4
T1
T2
T3
Time

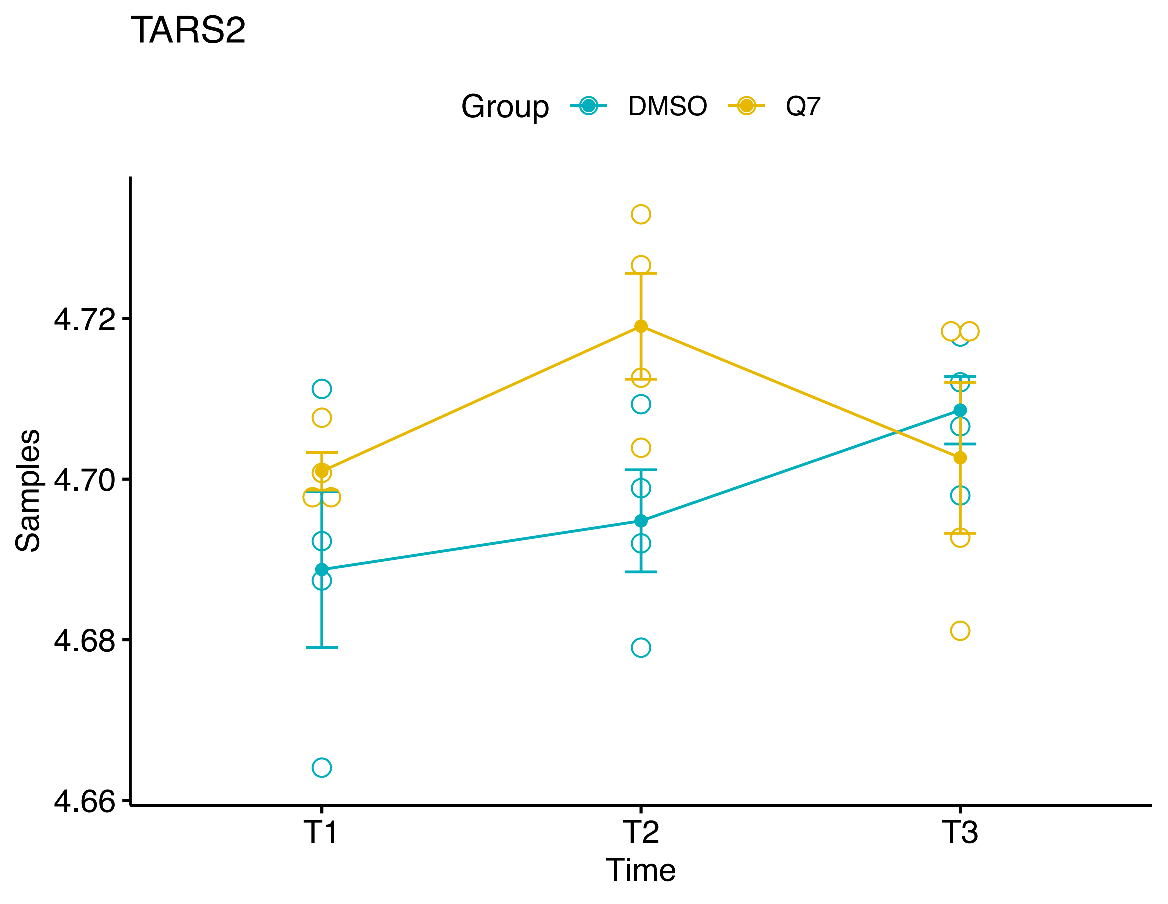

TARS2
Group
DMSO
Q7
Samples
4.72
4.70
4.68
4.66
T1
T2
T3
Time

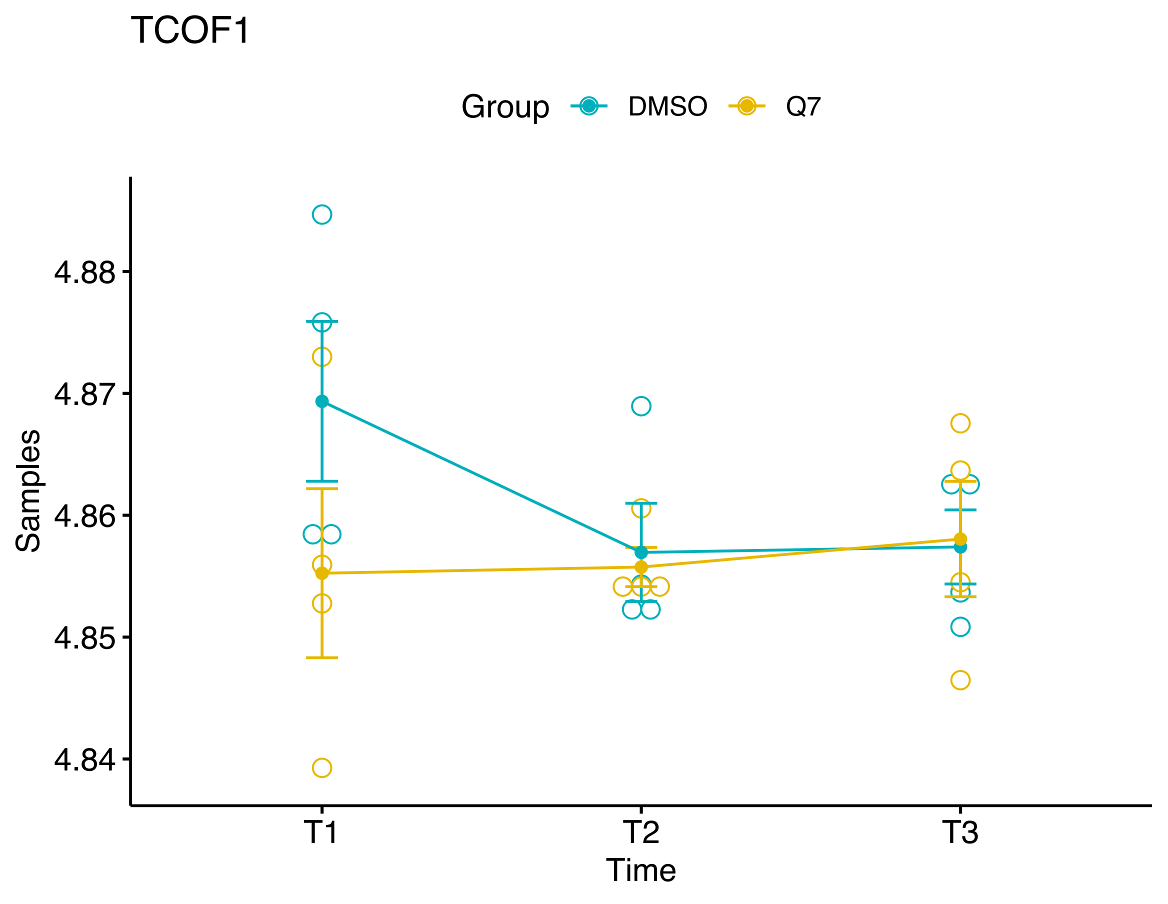

TCOF1
Group
DMSO
Q7
Samples
4.88
4.87
4.86
4.85
4.84
T1
T2
T3
Time

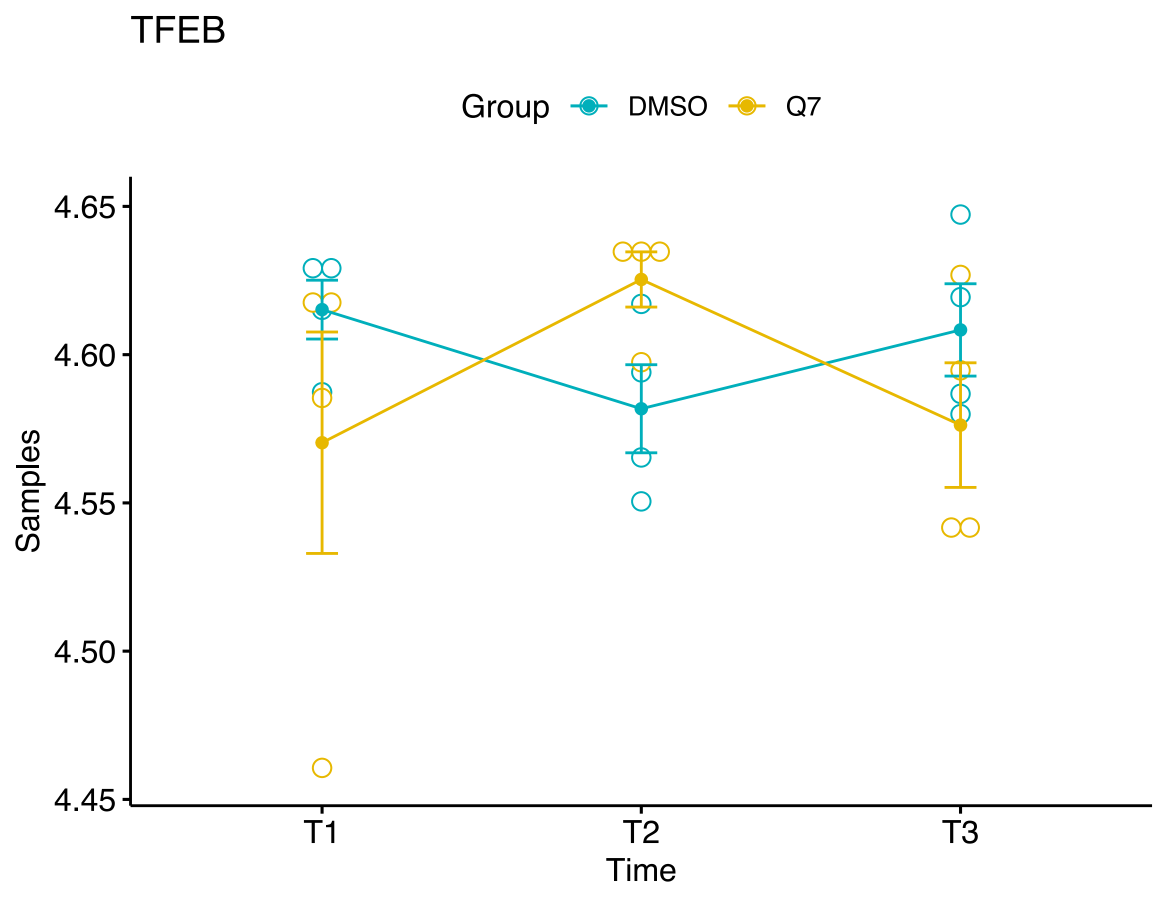

TFEB
Group
DMSO
Q7
4.65
4.60
4.55
4.50
4.45
Samples
T1
T2
T3
Time

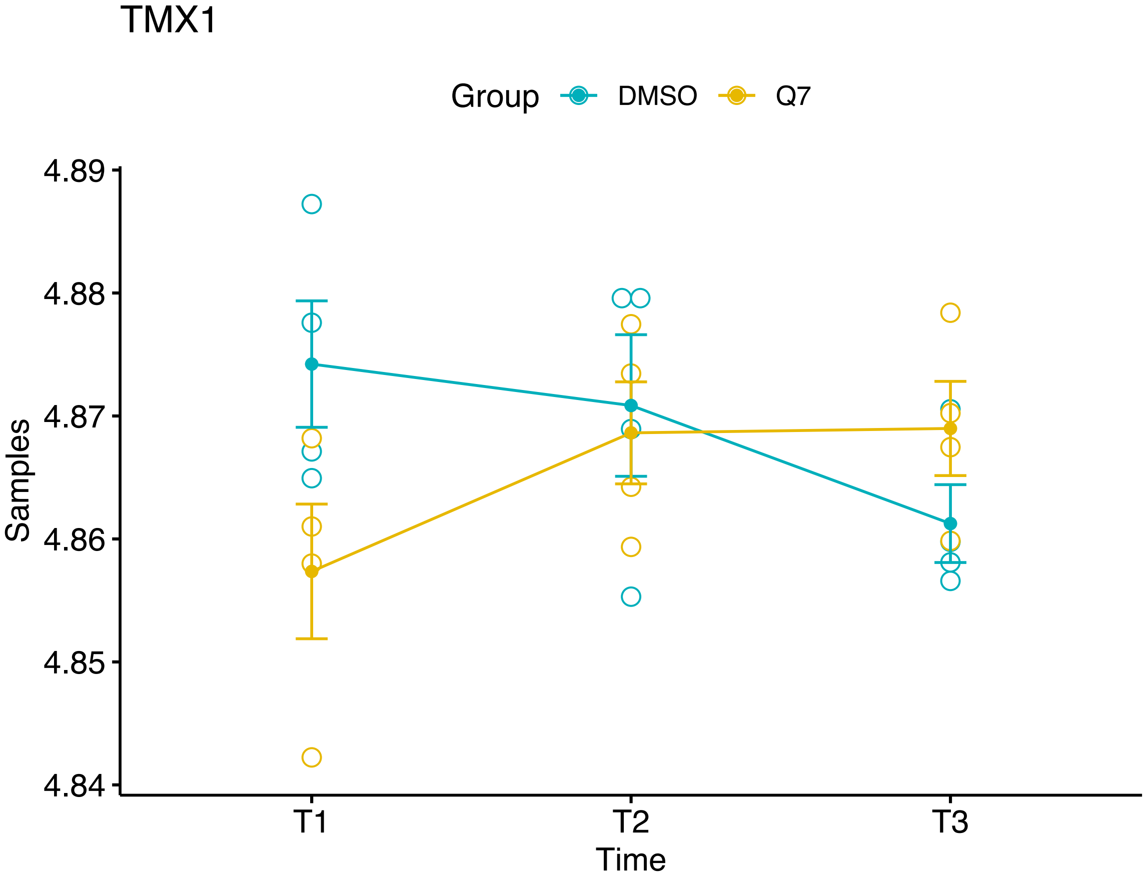
TMX1
Group
DMSO
Q7
4.89
4.88
4.87
4.86
4.85
4.84
Samples
T1
T2
T3
Time

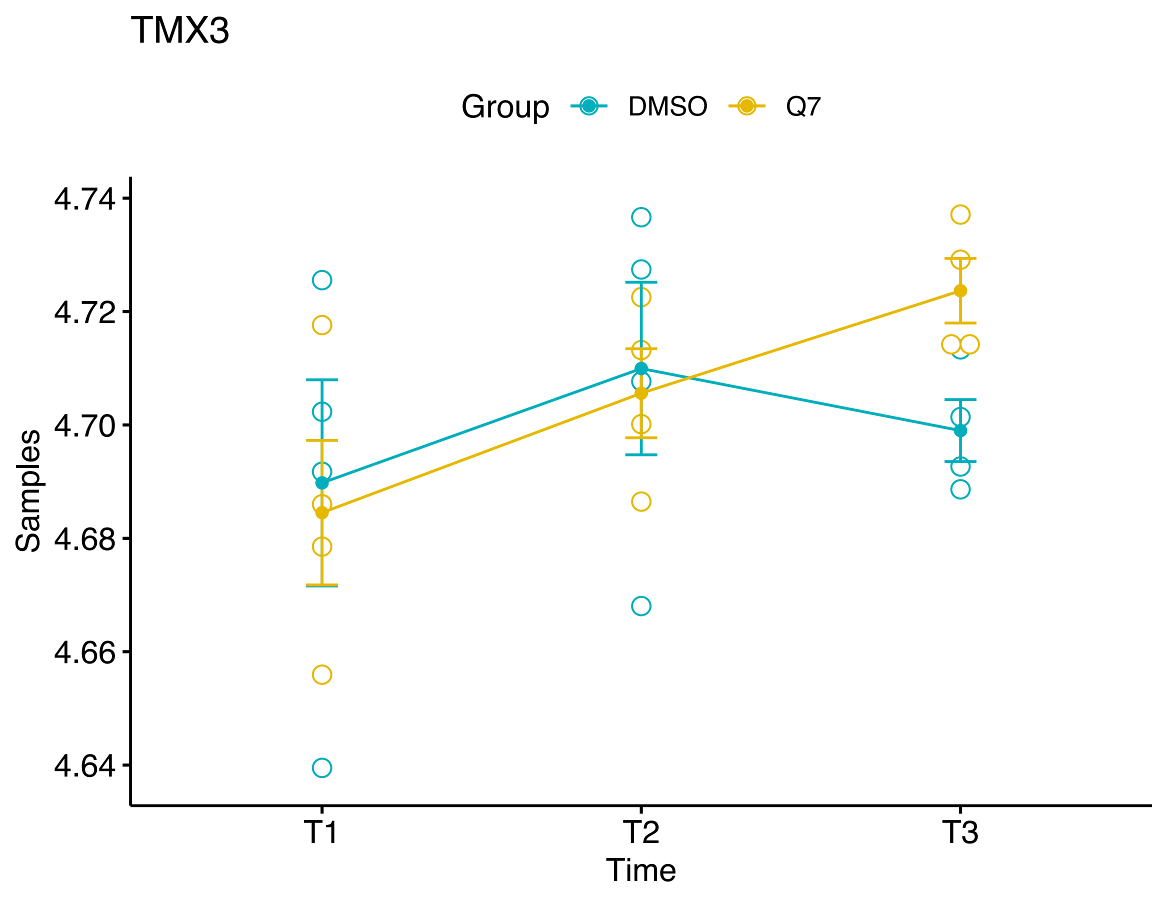
TMX3
Group
DMSO
Q7
4.74
4.72
4.70
4.68
4.66
4.64
Samples
T1
T2
T3
Time

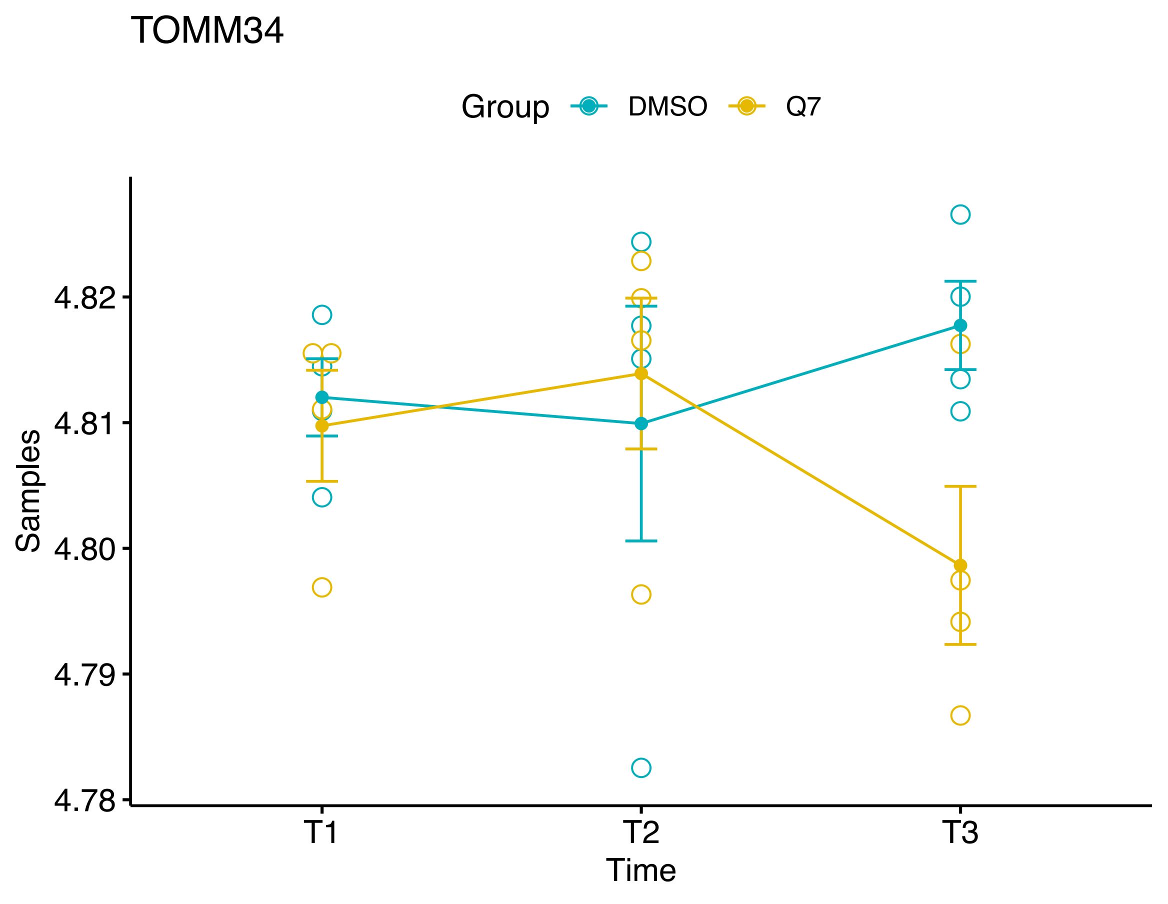
TOMM34
Group
DMSO
Q7
Samples
4.82
4.81
4.80
4.79
4.78
T1
T2
T3
Time

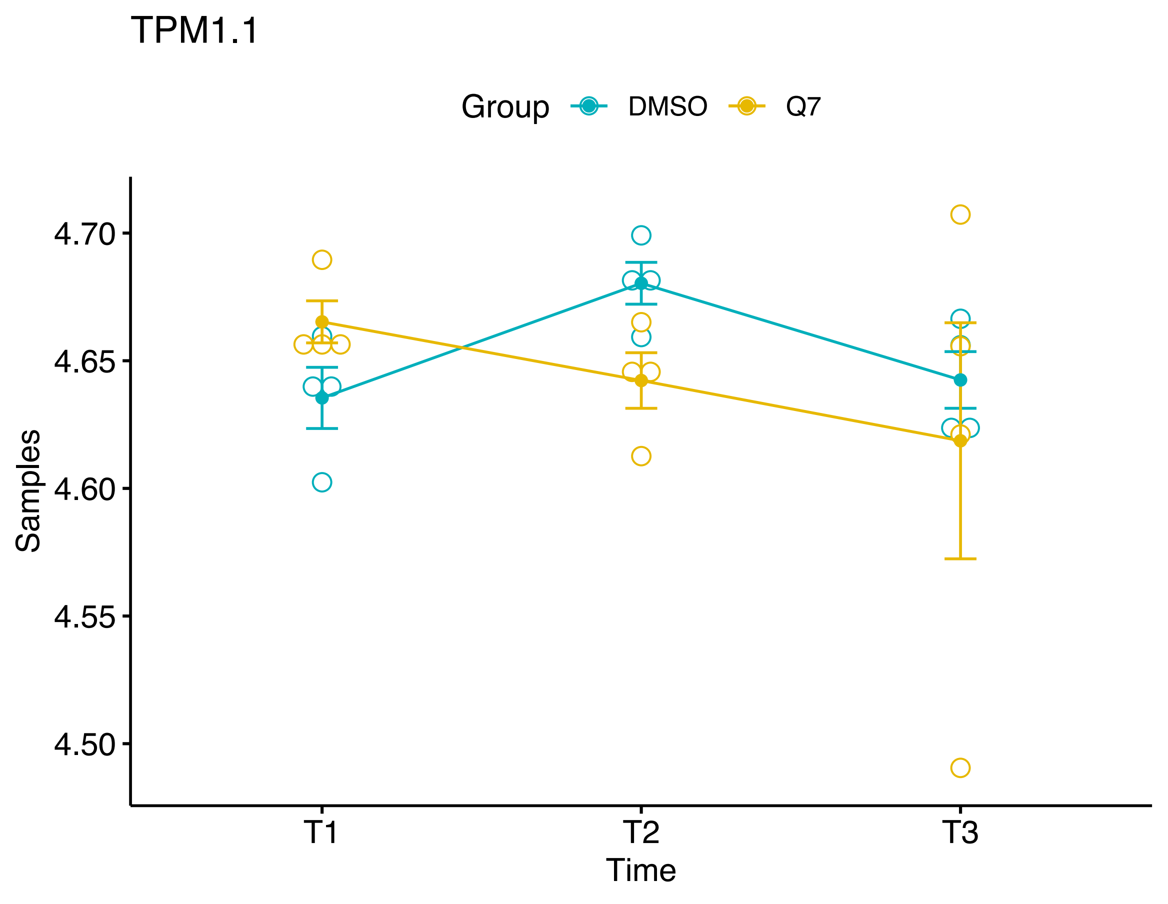
TPM1.1
Group
DMSO
Q7
4.70
4.65
4.60
4.55
4.50
Samples
T1
T2
T3
Time

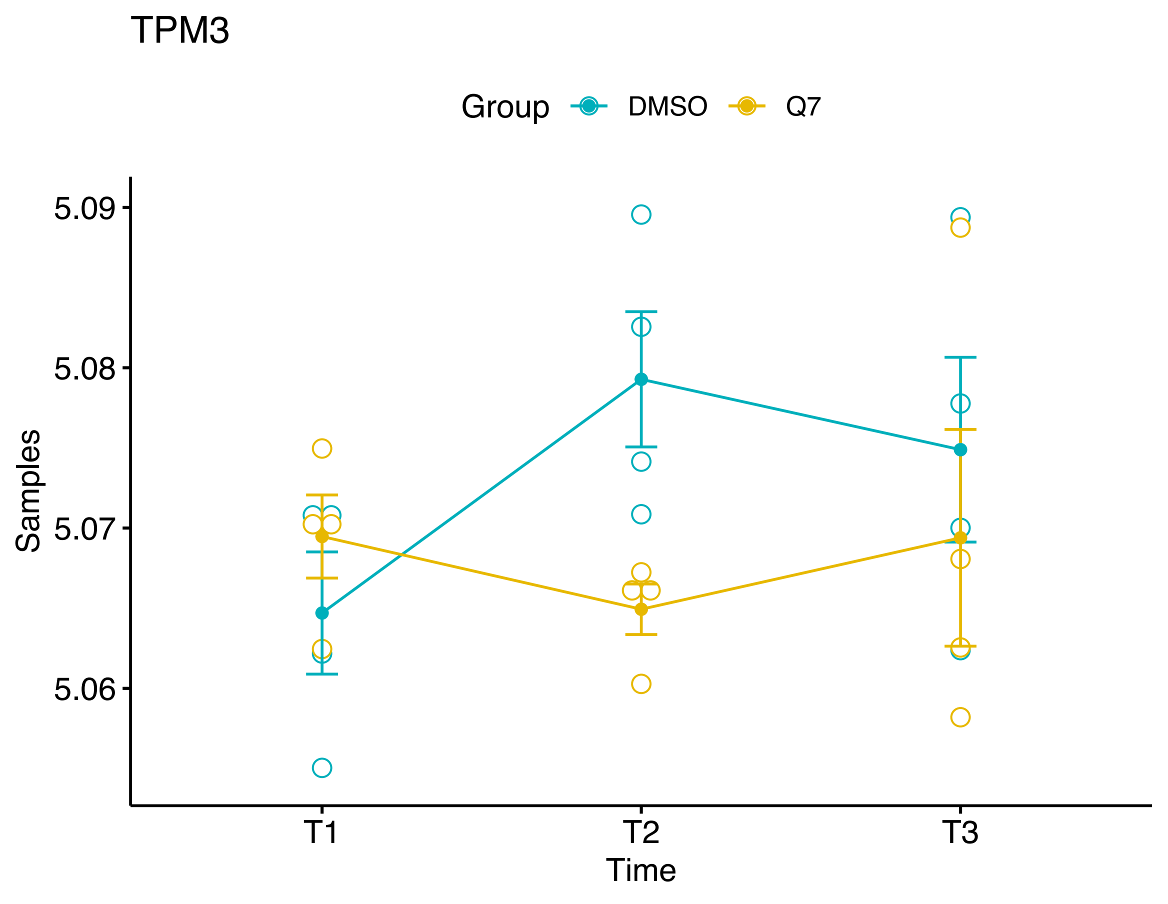

TPM3
Group
DMSO
Q7
Samples
5.09
5.08
5.07
5.06
T1
T2
T3
Time

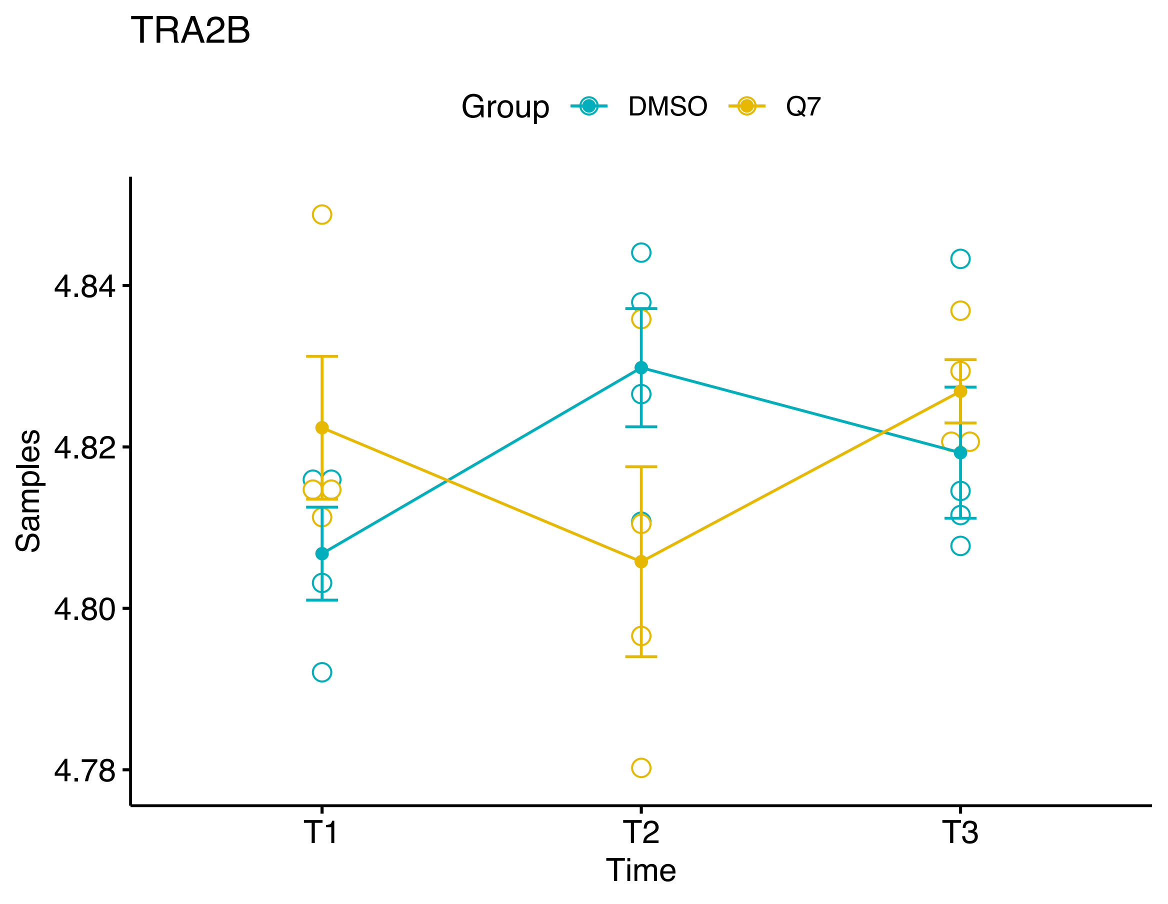
TRA2B
Group
DMSO
Q7
Samples
4.84
4.82
4.80
4.78
T1
T2
T3
Time

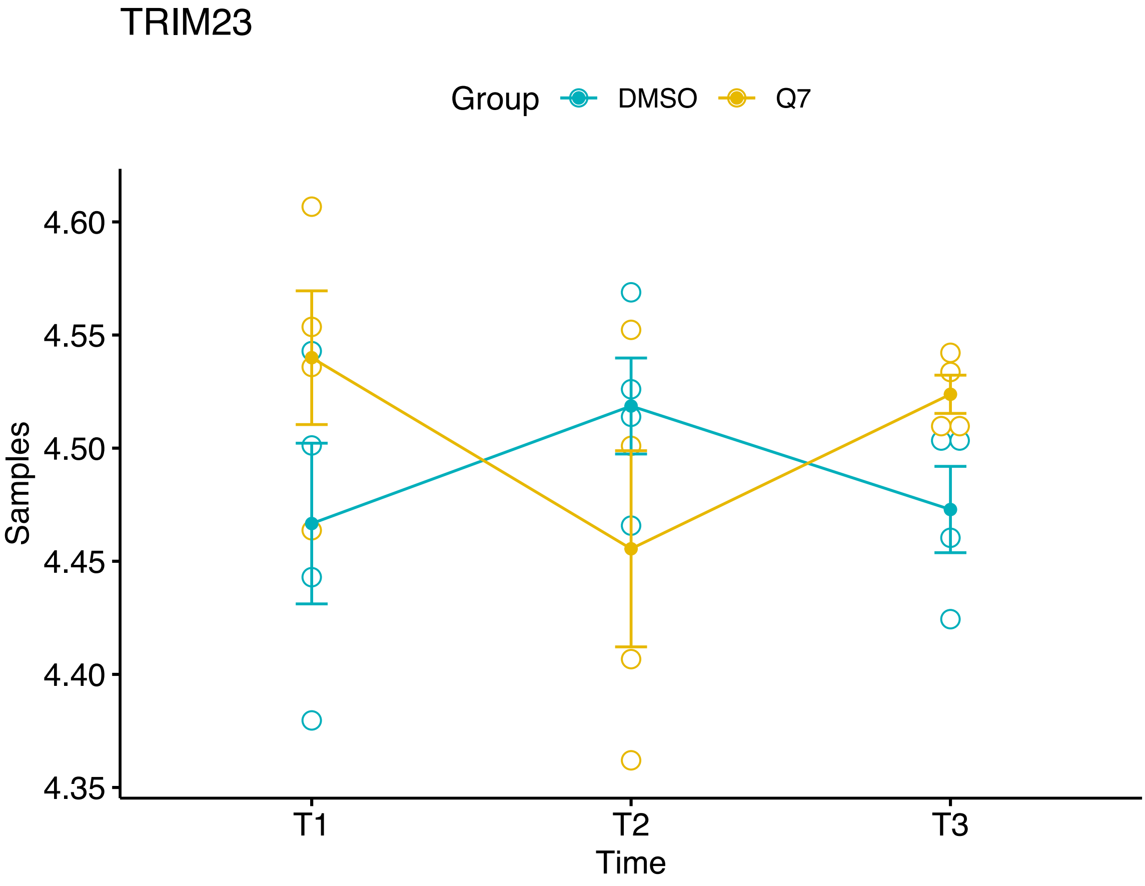
TRIM23
Group
DMSO
Q7
Samples
4.60
4.55
4.50
4.45
4.40
4.35
T1
T2
T3
Time

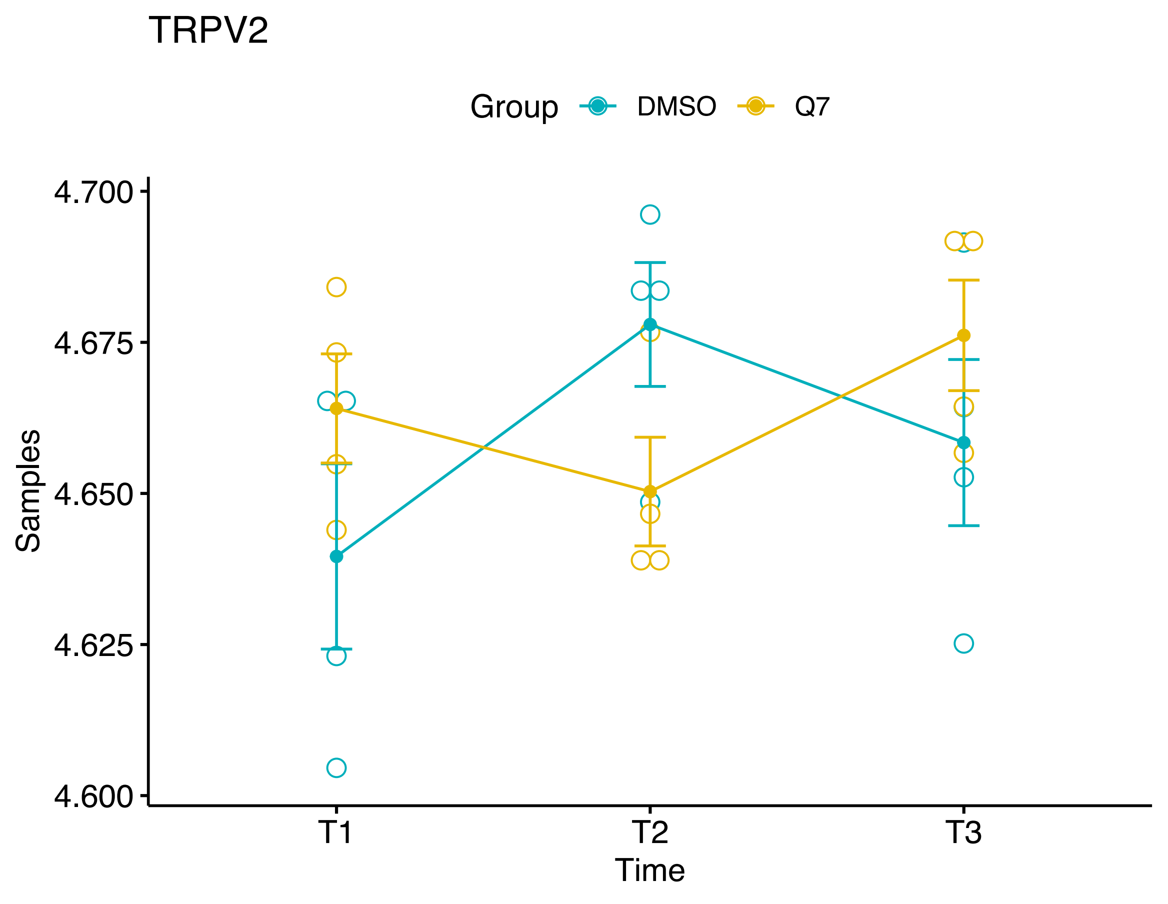
TRPV2
Group
DMSO
Q7
4.700
4.675
4.650
4.625
4.600
Samples
T1
T2
T3
Time

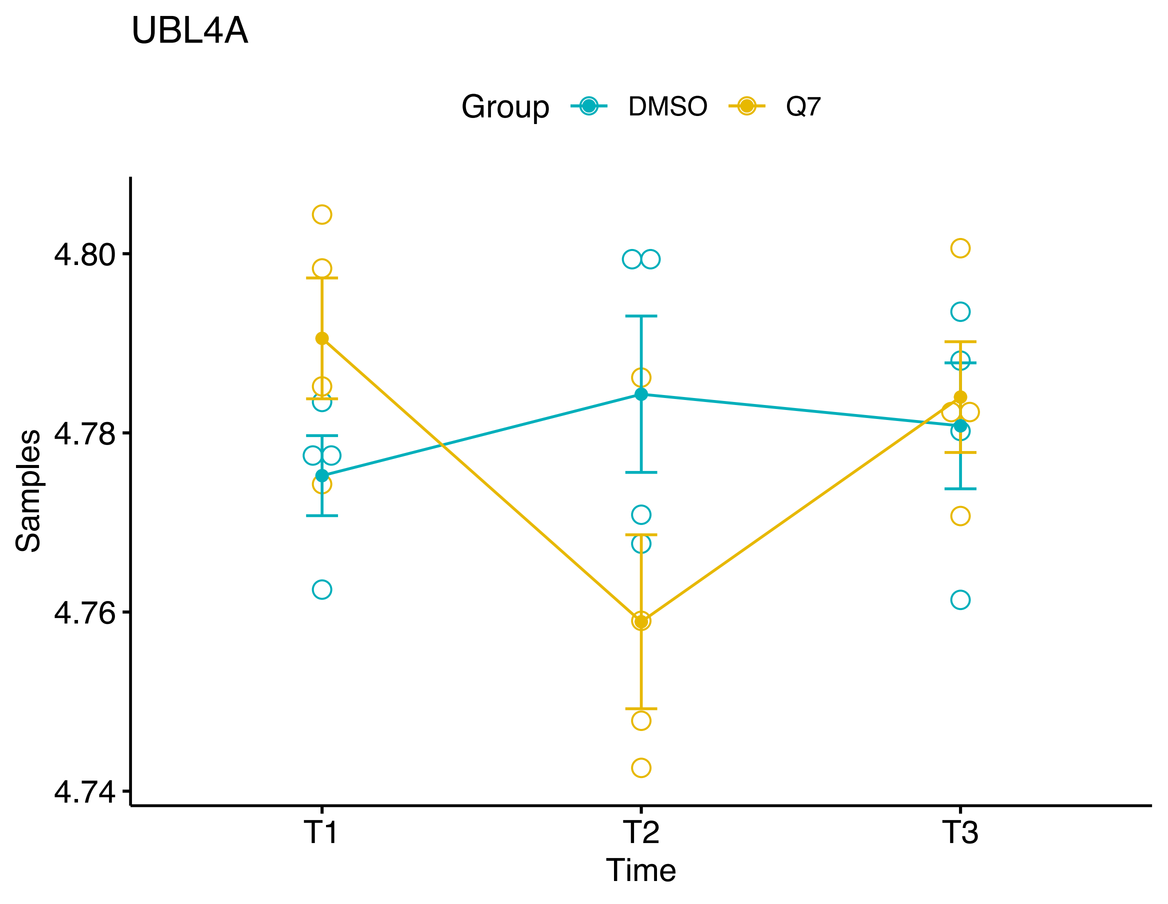
UBL4A
Group
DMSO
Q7
Samples
4.80
4.78
4.76
4.74
T1
T2
T3
Time

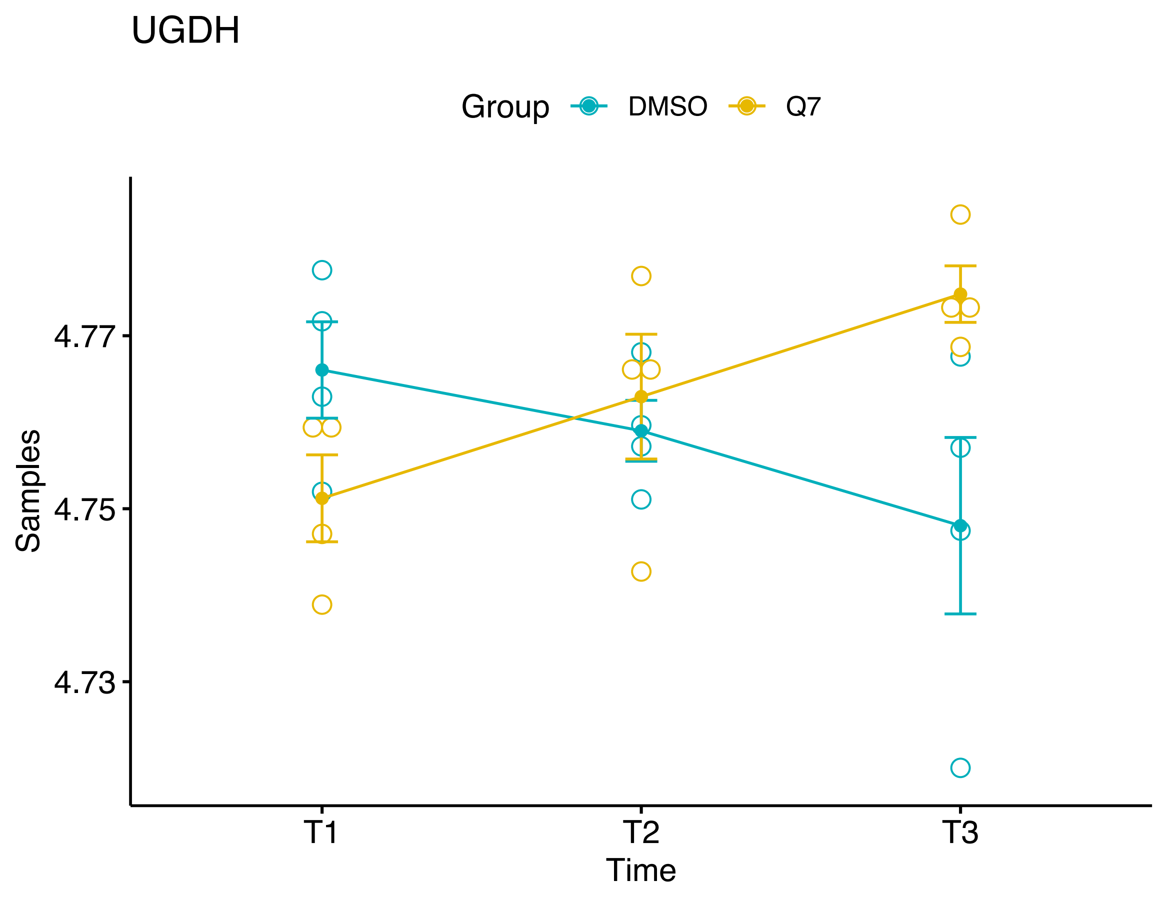
UGDH
Group
DMSO
Q7
4.77
4.75
4.73
Samples
T1
T2
T3
Time

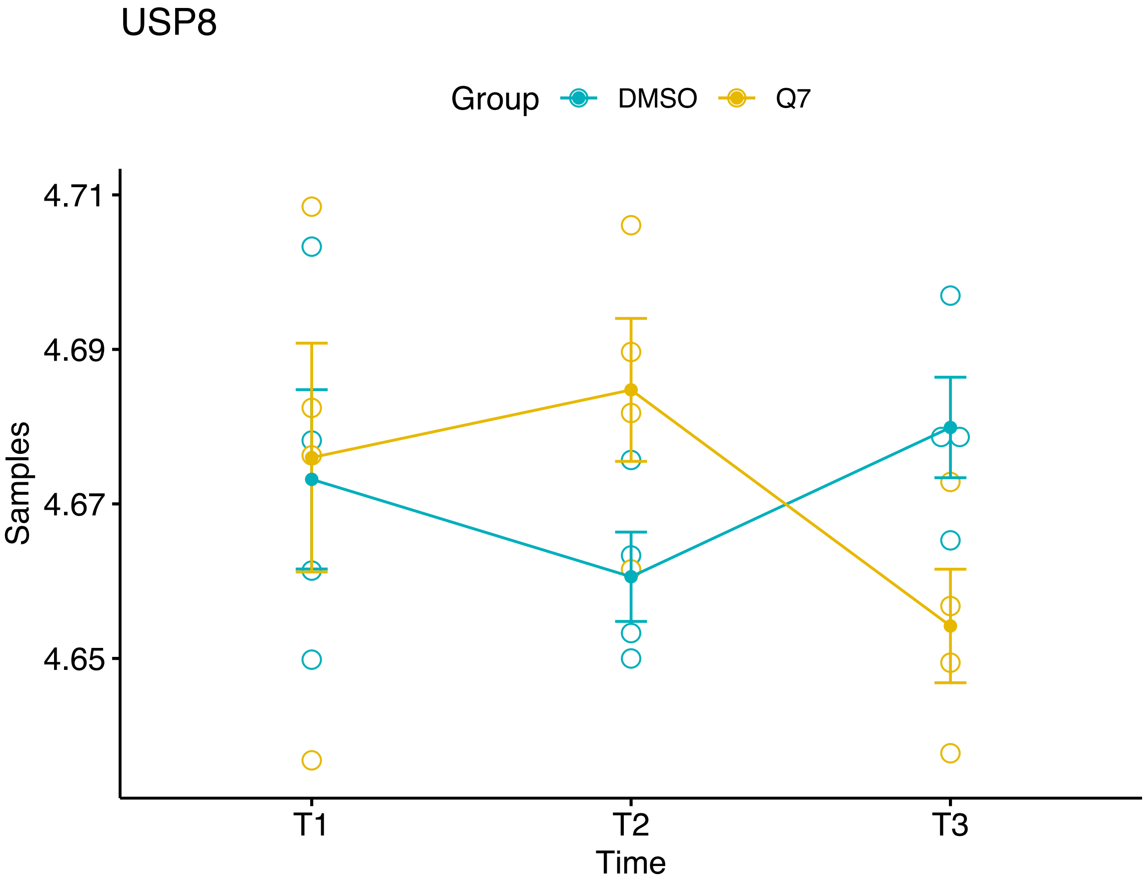

USP8
Group
DMSO
Q7
4.71
4.69
4.67
4.65
Samples
T1
T2
T3
Time

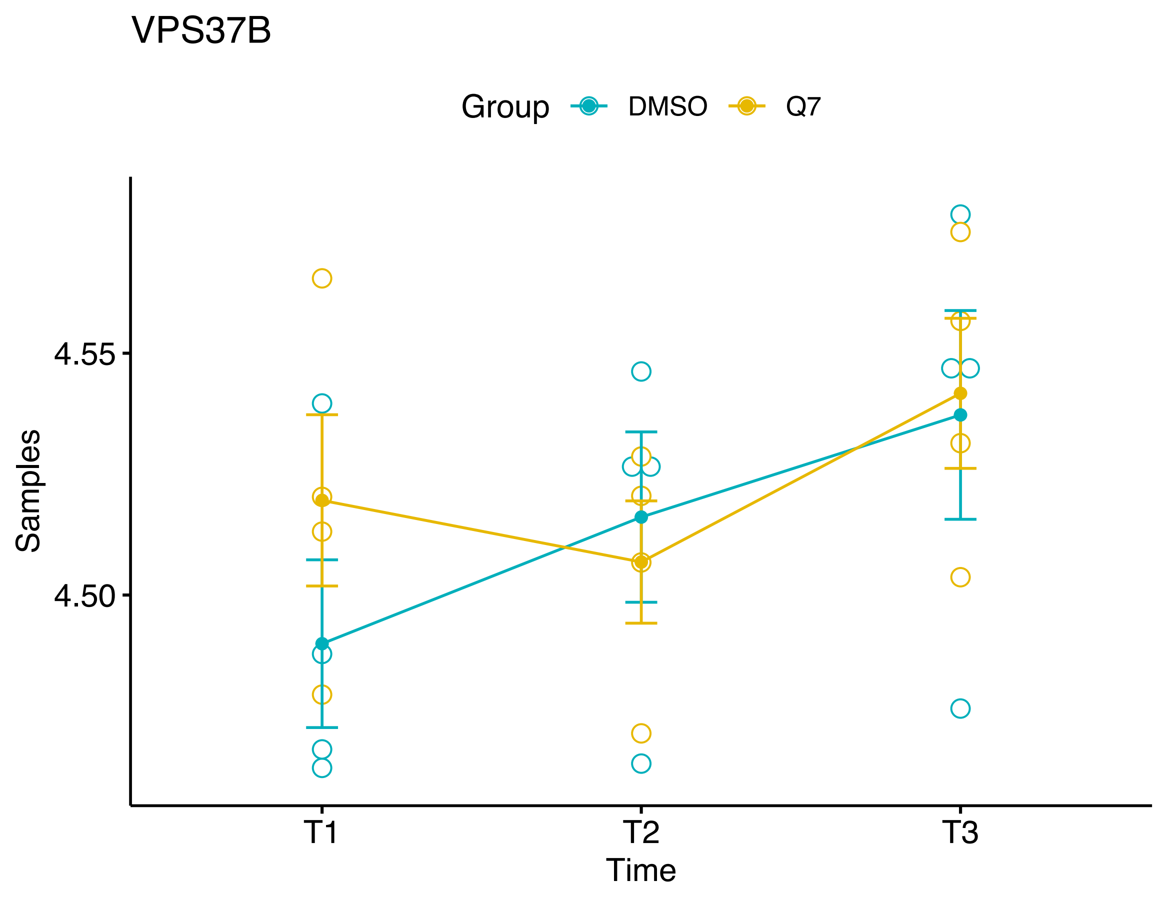

VPS37B
Group
DMSO
Q7
4.55
4.50
Samples
T1
T2
T3
Time

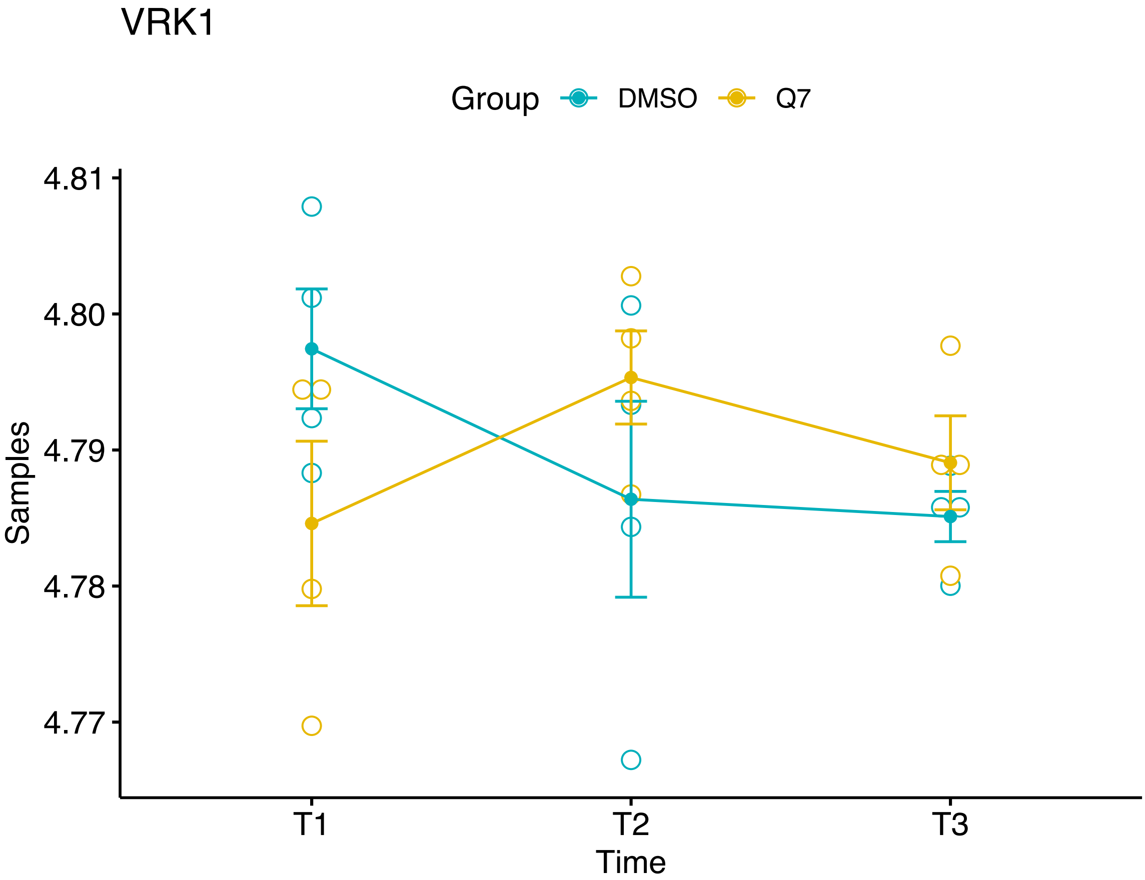
VRK1
Group
DMSO
Q7
Samples
4.81
4.80
4.79
4.78
4.77
T1
T2
T3
Time

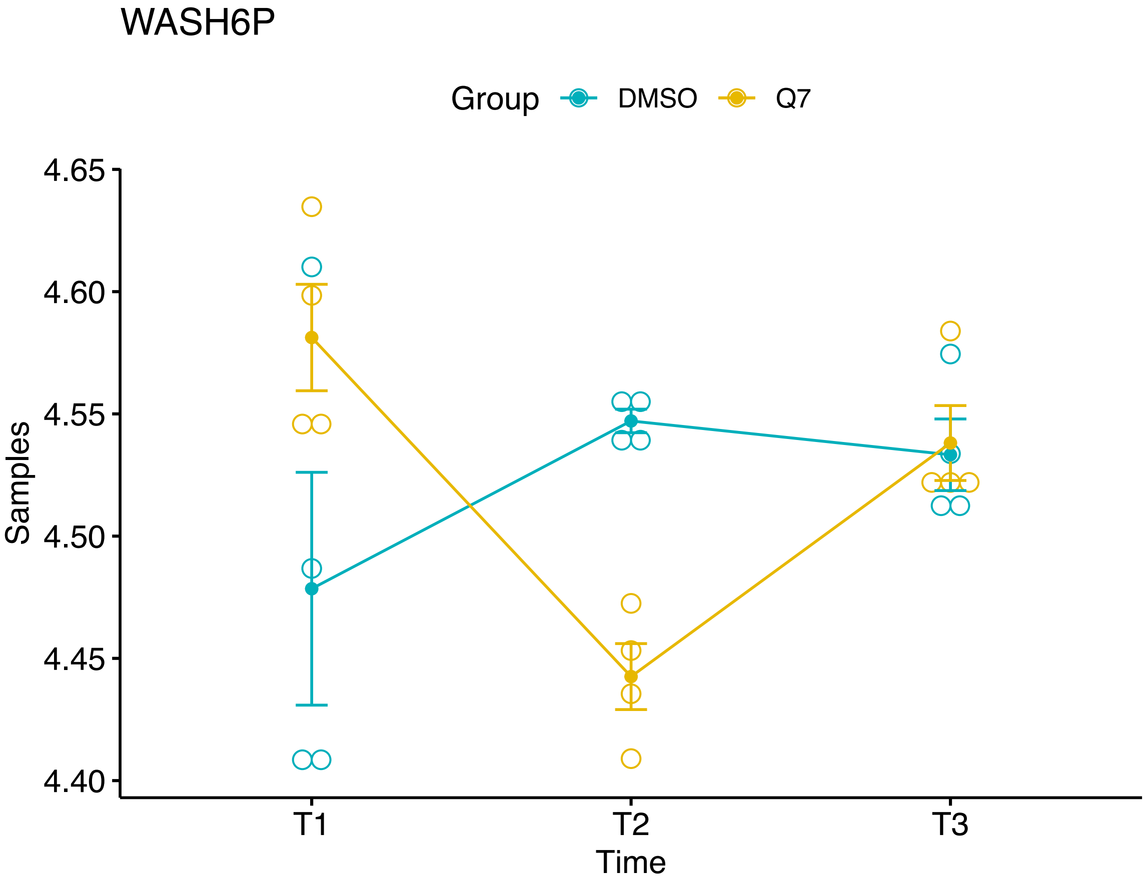

WASH6P
Group
DMSO
Q7
4.65
4.60
4.55
4.50
4.45
4.40
Samples
T1
T2
T3
Time

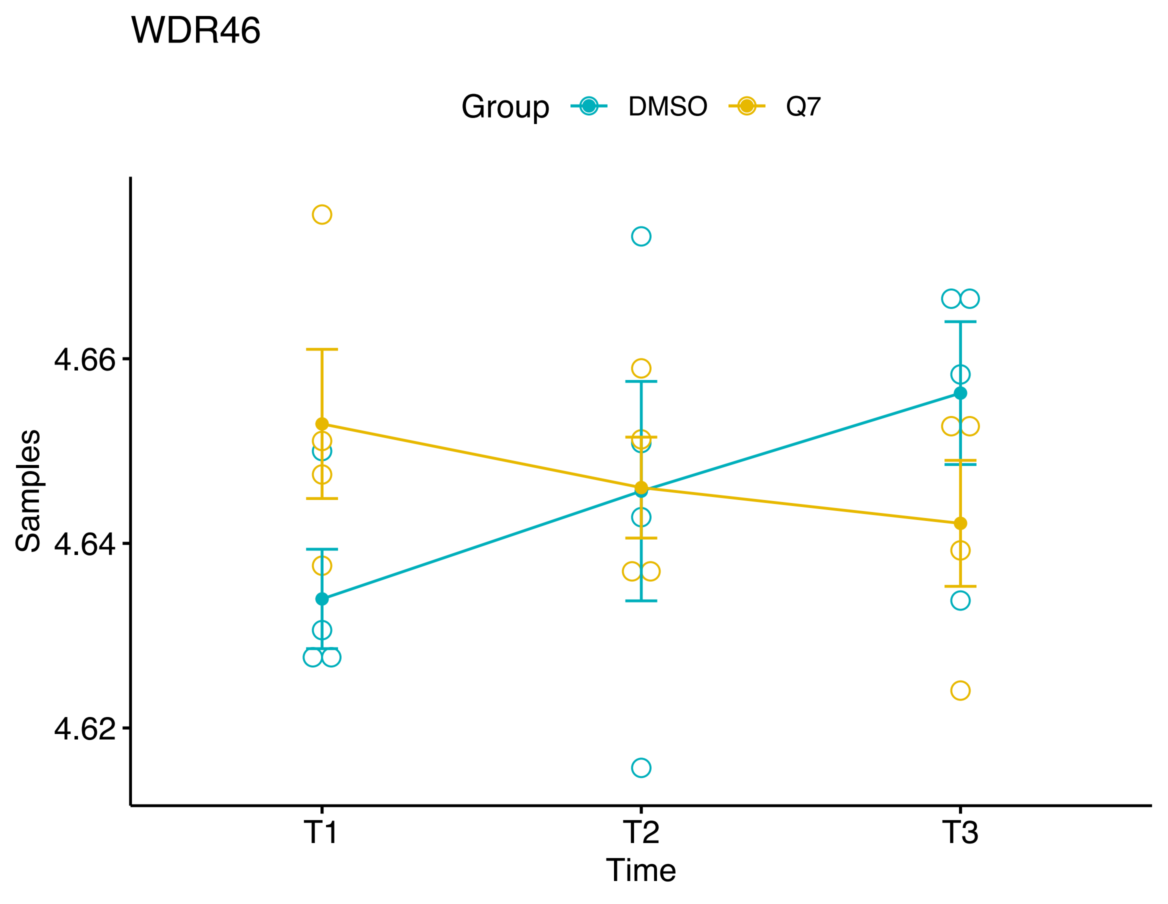

WDR46
Group
DMSO
Q7
4.66
4.64
4.62
Samples
T1
T2
T3
Time

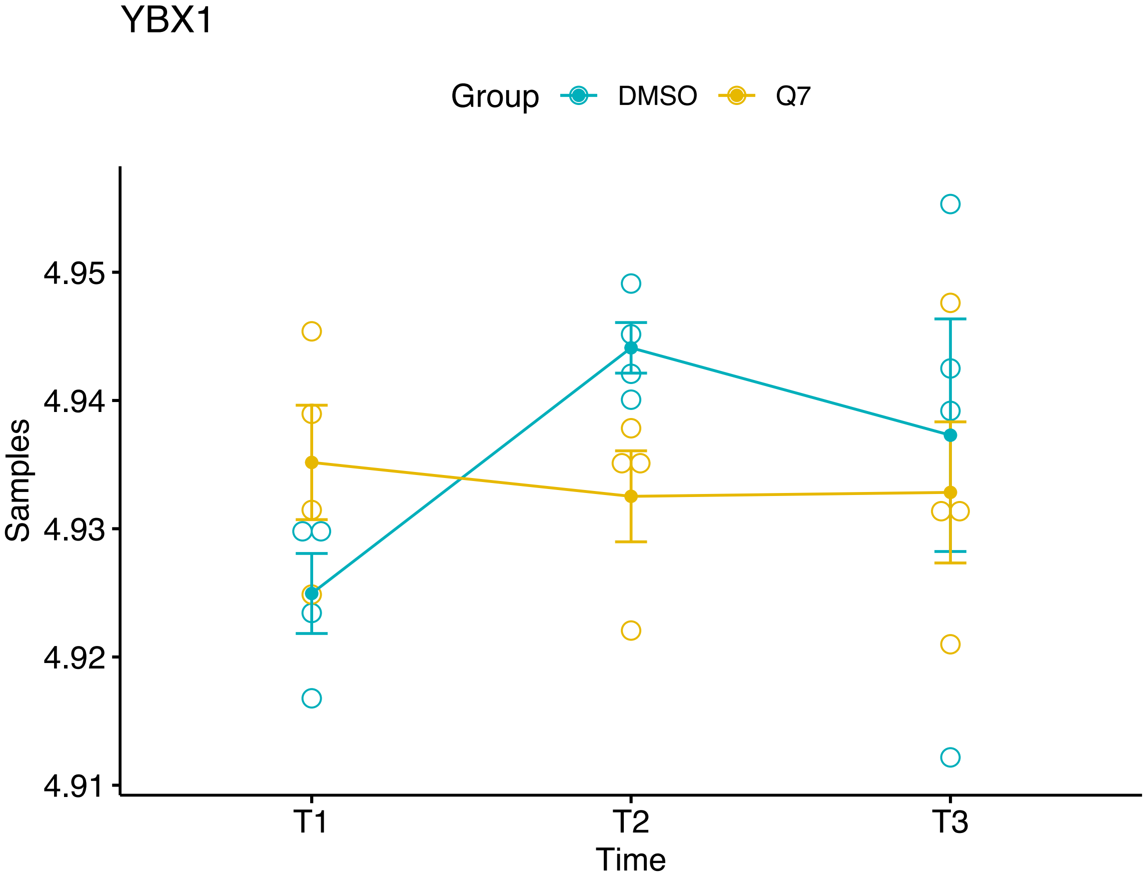

YBX1
Group
DMSO
Q7
Samples
4.95
4.94
4.93
4.92
4.91
T1
T2
T3
Time

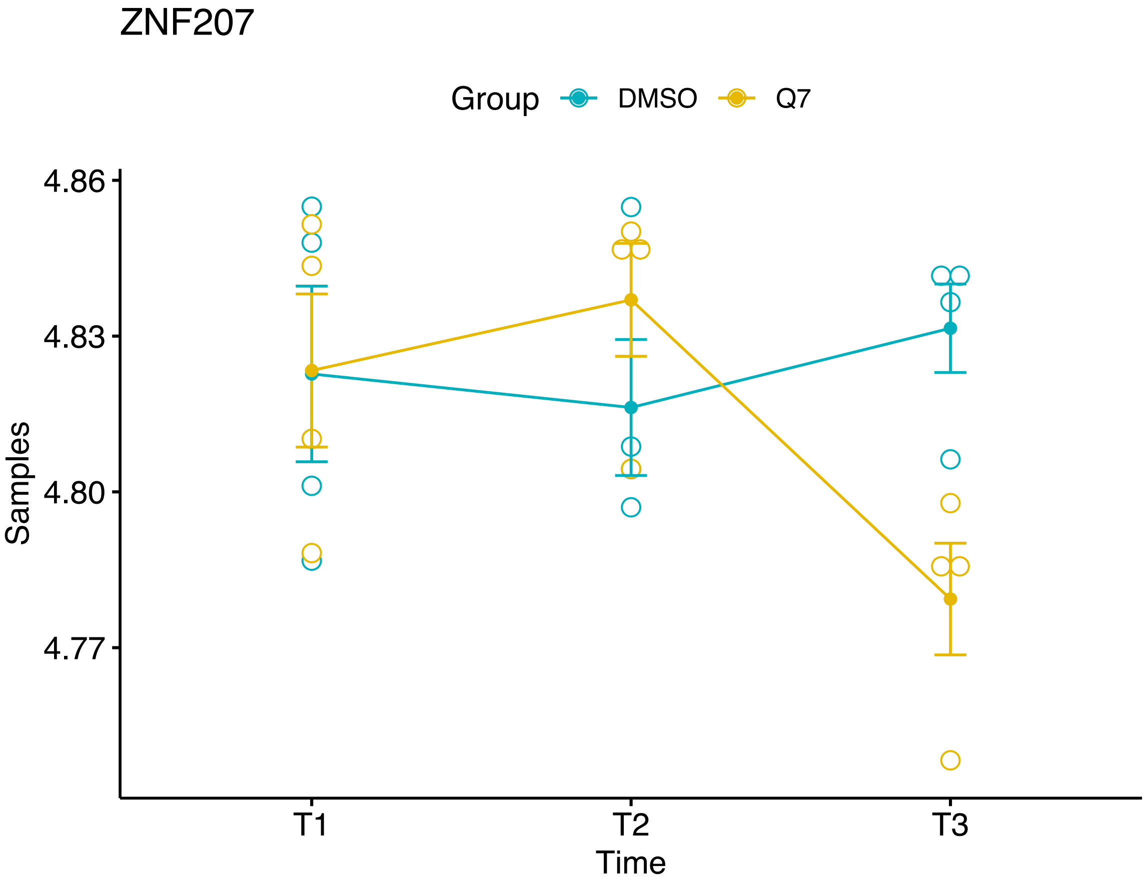

ZNF207
Group
DMSO
Q7
4.86
4.83
4.80
4.77
Samples
T1
T2
T3
Time

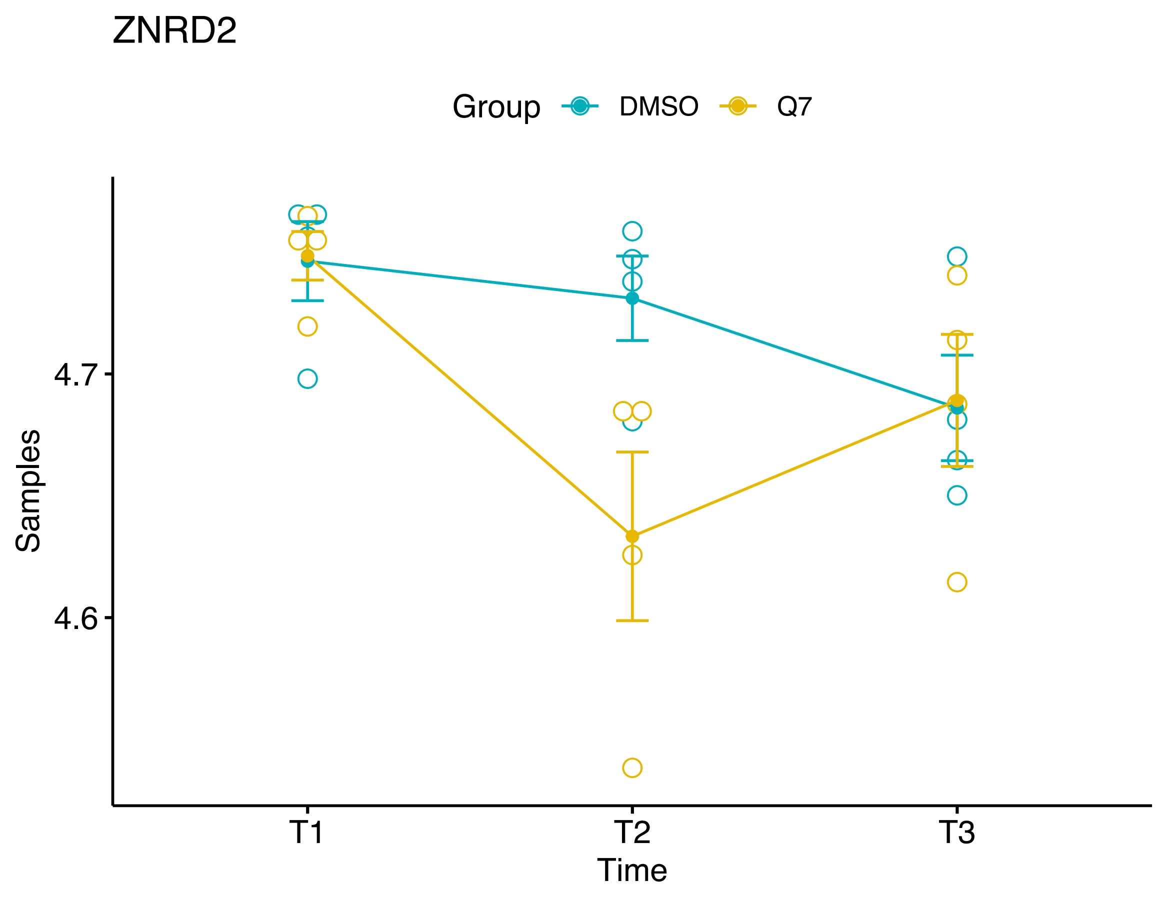
ZNRD2
Group
DMSO
Q7
4.7
4.6
Samples
T1
T2
T3
Time

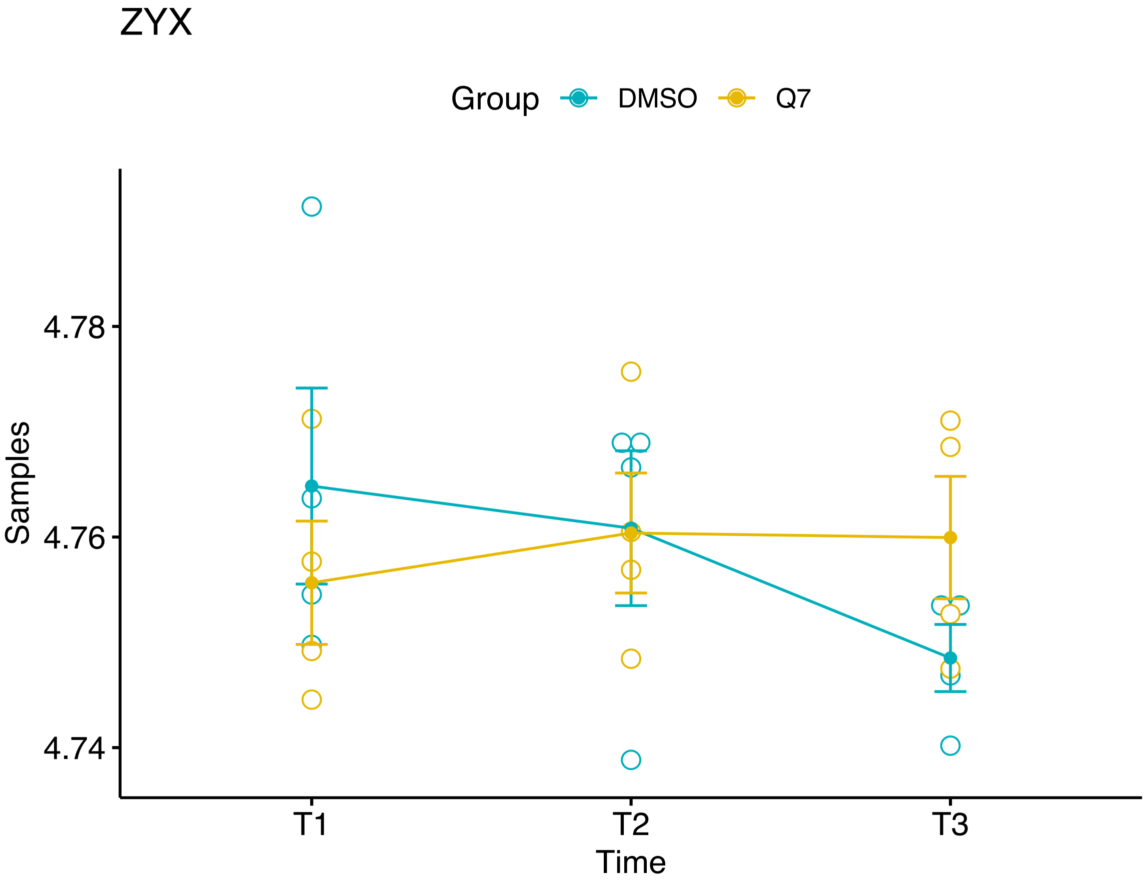

ZYX
Group
DMSO
Q7
Samples
4.78
4.76
4.74
T1
T2
T3
Time
