# Supplementary figures and images for: 1,4-dihydroxy quininib activates ferroptosis pathways in metastatic uveal melanoma and reveals a novel prognostic biomarker signature
Source: Cell Death Discov. 2024 Feb 10;10:70. doi: 10.1038/s41420-023-01773-8 (PMC10858877; doi:10.1038/s41420-023-01773-8)

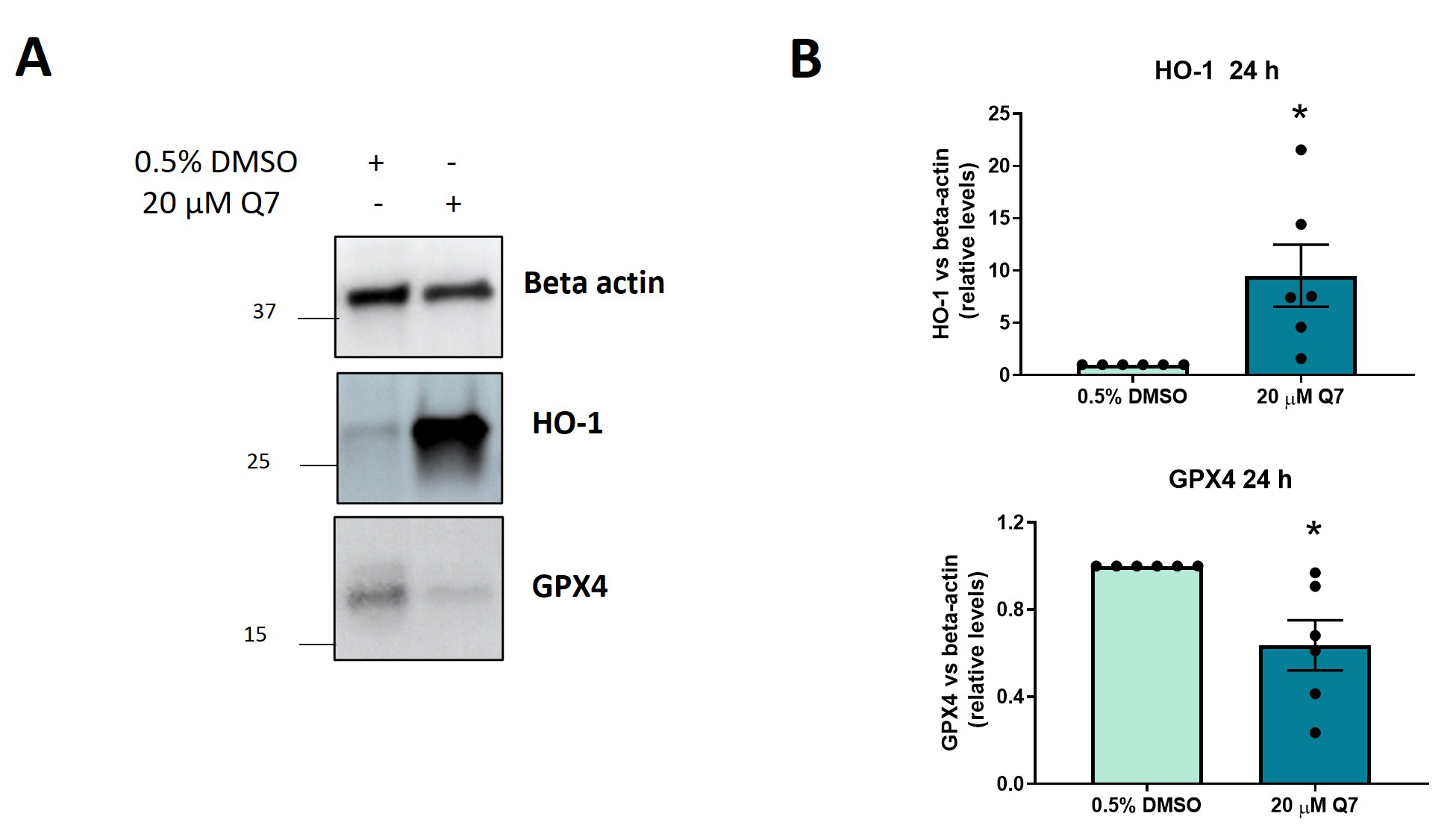

Supplement: Supplementary file 3 — Supplementary Figure 2 [file 41420_2023_1773_MOESM3_ESM.png]

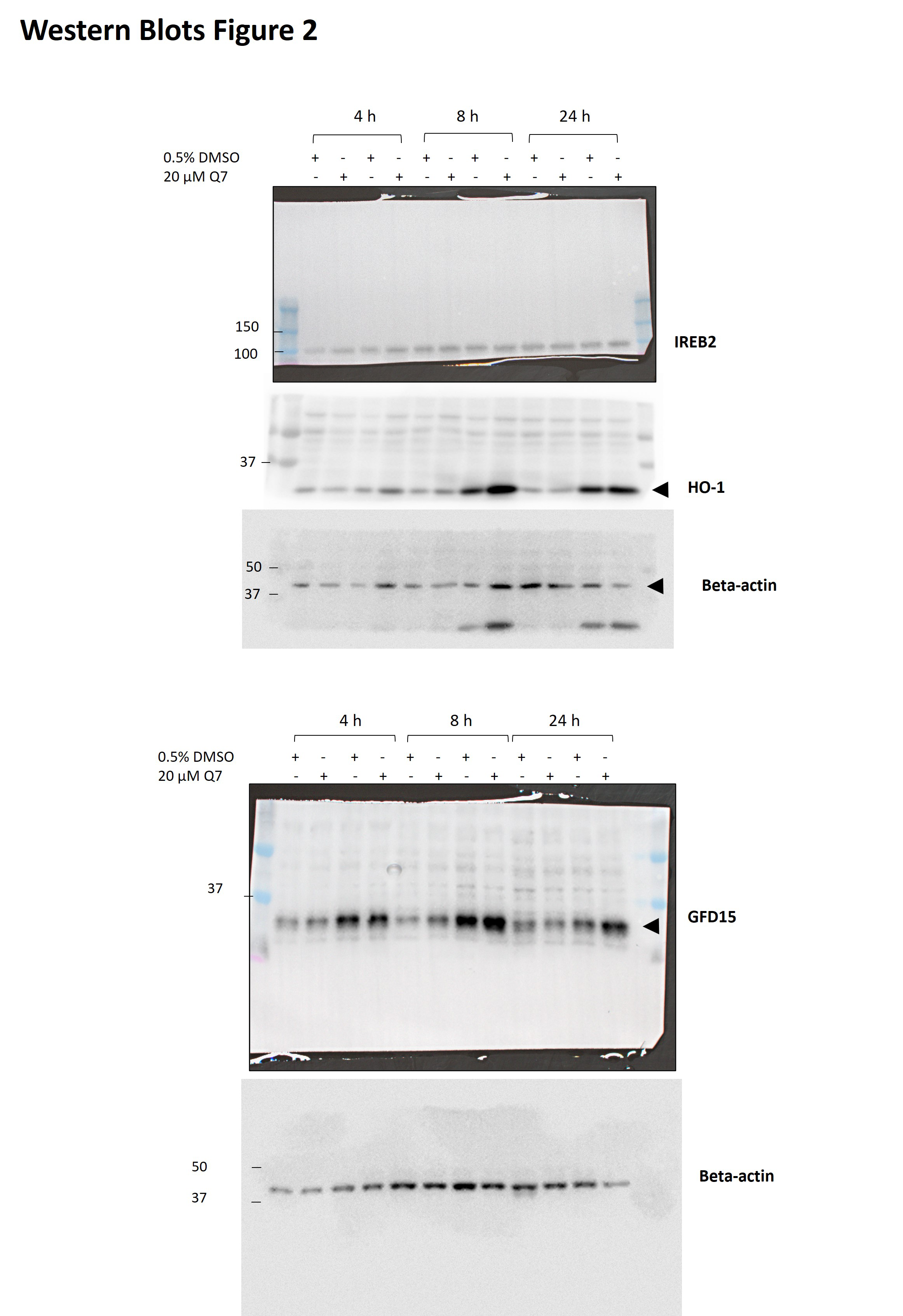

Supplement: Supplementary file 6 — Original Western Blot Figure 2 [file 41420_2023_1773_MOESM6_ESM.png]

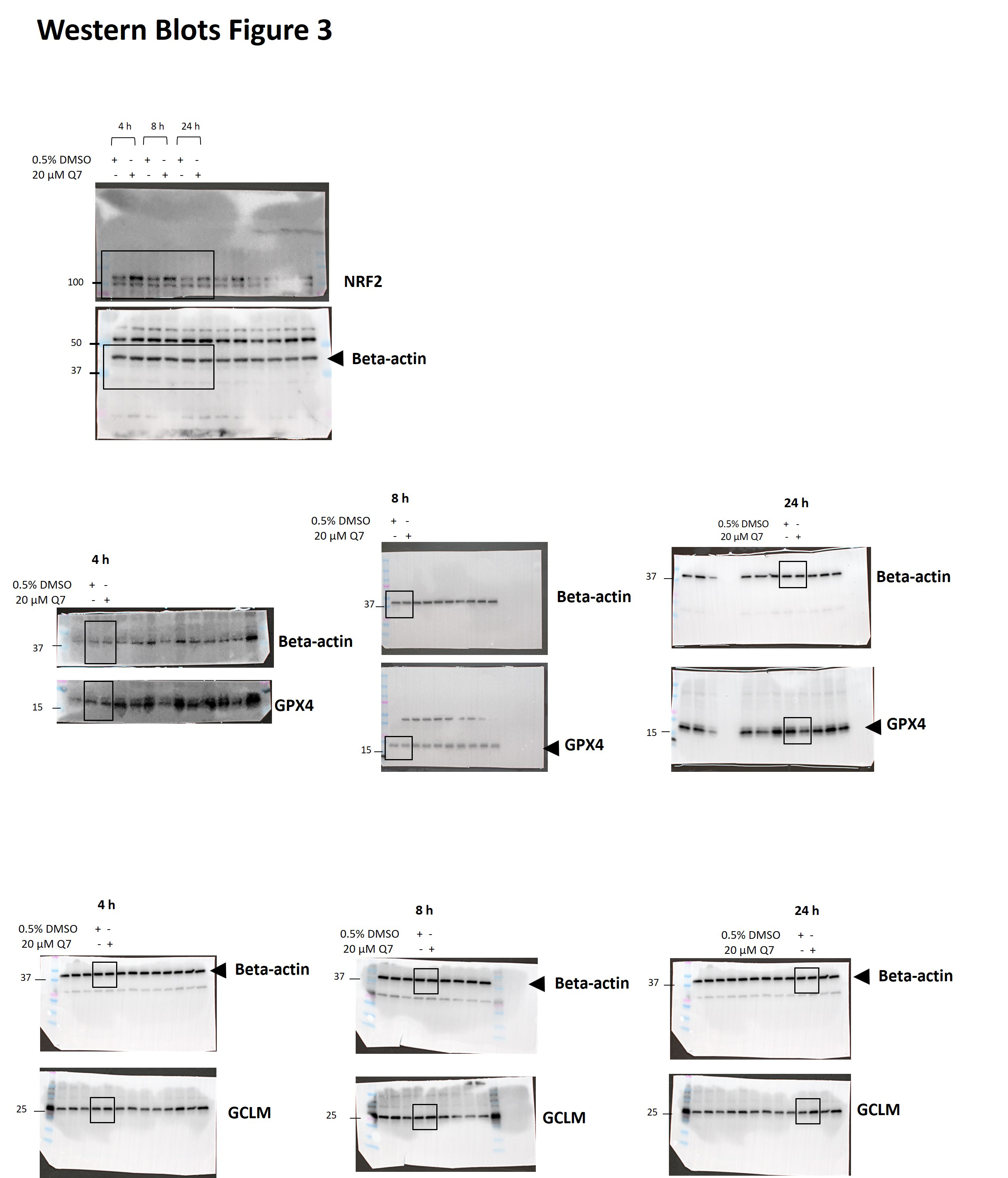

Supplement: Supplementary file 7 — Original Western Blot Figure 3 [file 41420_2023_1773_MOESM7_ESM.png]

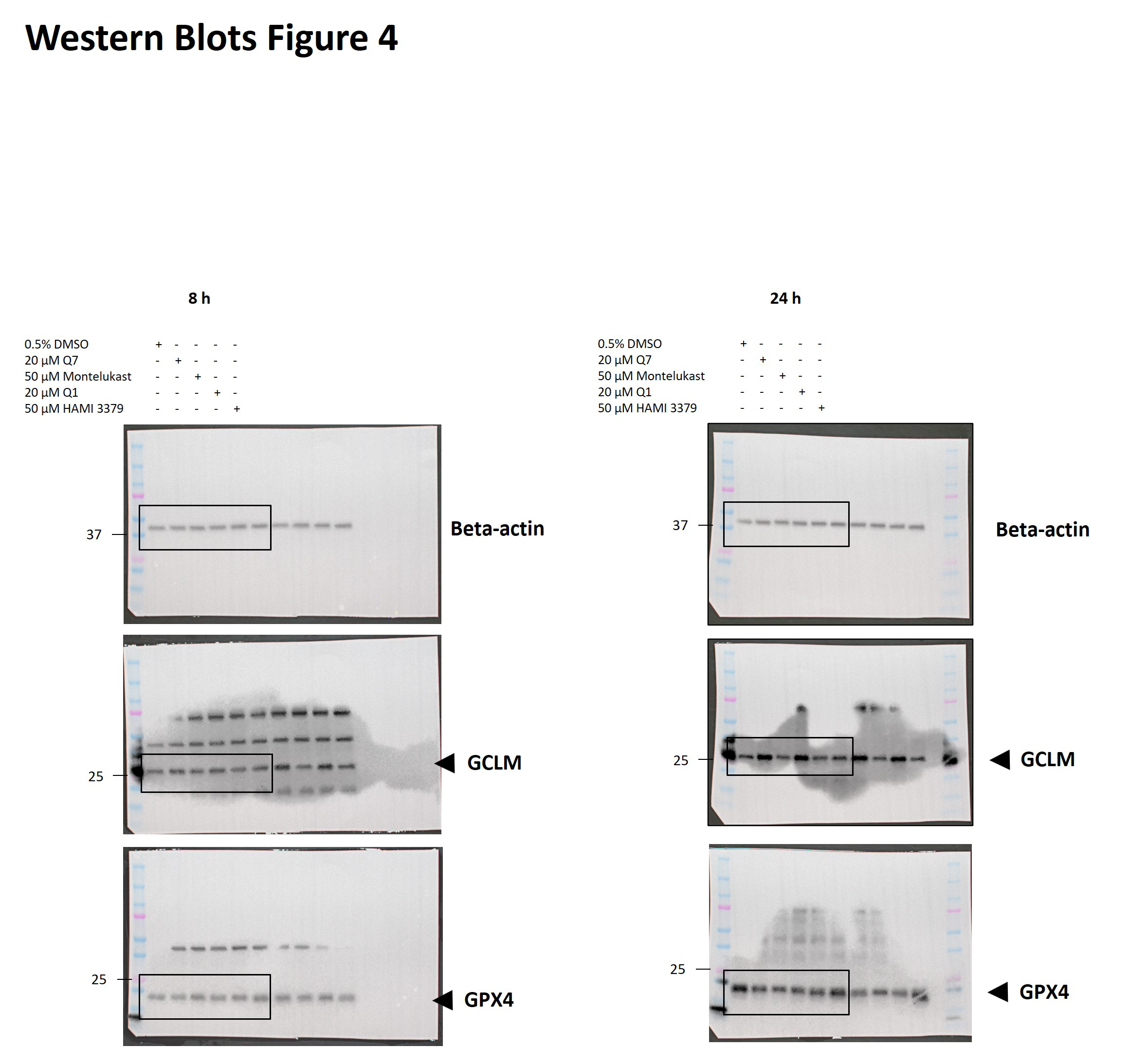

Supplement: Supplementary file 8 — Original Western Blot Figure 4 [file 41420_2023_1773_MOESM8_ESM.png]

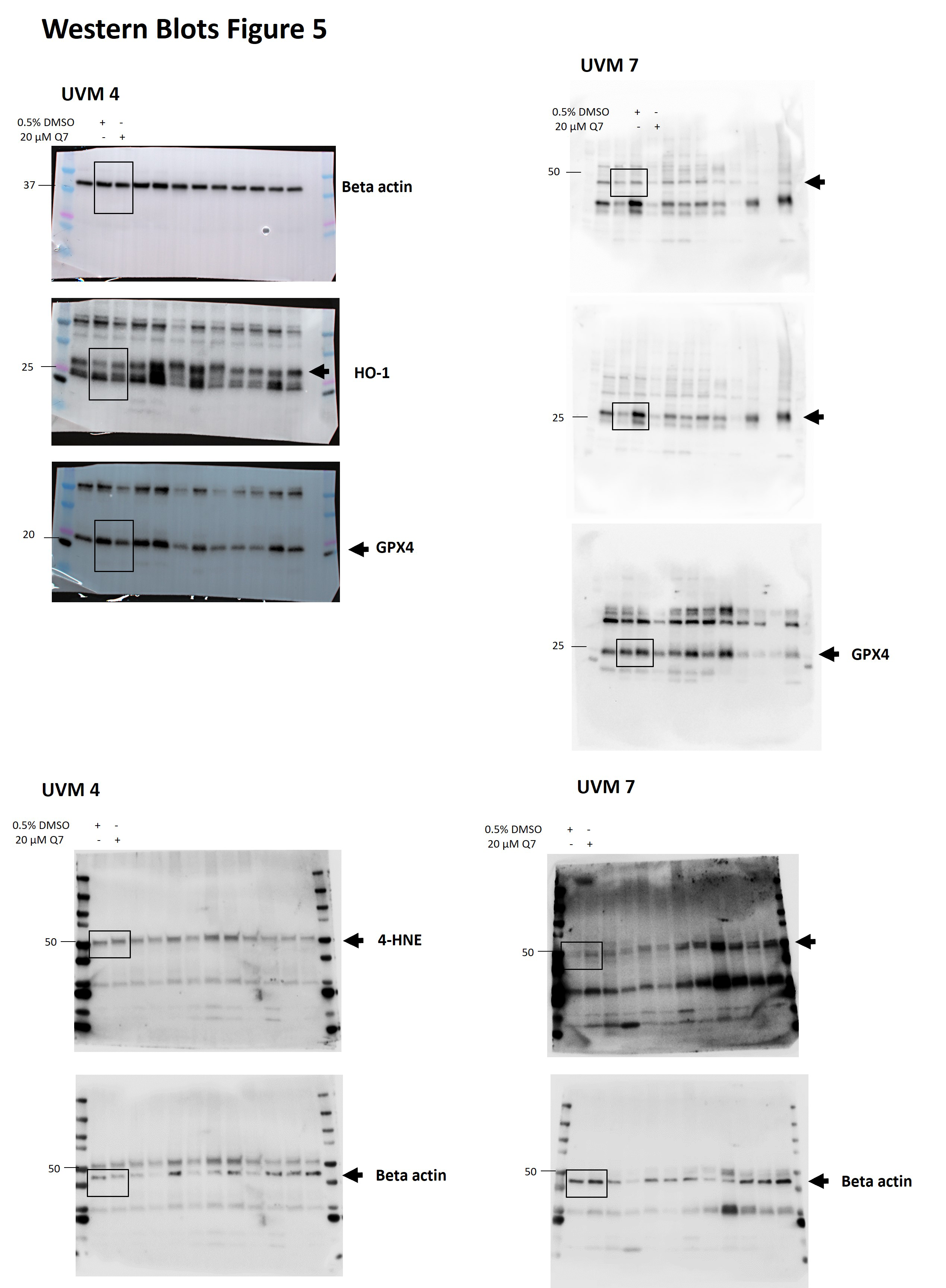

Supplement: Supplementary file 9 — Original Western Blot Figure 5 [file 41420_2023_1773_MOESM9_ESM.png]
