# Supplementary material for: 1,4-dihydroxy quininib activates ferroptosis pathways in metastatic uveal melanoma and reveals a novel prognostic biomarker signature
Source: Cell Death Discov. 2024 Feb 10;10:70. doi: 10.1038/s41420-023-01773-8 (PMC10858877; doi:10.1038/s41420-023-01773-8)
Supplement: Supplementary file 5 — Supplementary Figure Legends [file 41420_2023_1773_MOESM5_ESM.docx]

**Supplementary Dataset 1.** Dataset containing the results from the proteome-profiling of whole OMM2.5 cell extracts treated with 0.5% DMSO or 20 μM 1,4-dihydroxy quininib for 4, 8 and 24 hours.

**Supplementary Figure 1.** Proteins showing a significant time-dependent expression in OMM2.5 cells upon treatment with 20 μM 1,4-dihydroxy quininib for 4 (T1), 8 (T2) and 24 (T3) hours.

**Supplementary Figure 2. 24-hour treatment with 20 μM 1,4 dihydroxy quininib affects HO-1 and GPX4 in Mel285 cells. (A)** Western blot analysis of HO-1 and GPX4 expression in 0.5% DMSO or 20 μM Q7 treated Mel285 cells after 24 (n = 6 independent experiments) hours of treatment (*, p < 0.05). **(B)** Densitometric quantification of HO-1 (upper panel) and GPX4 (lower panel) vs beta-actin as determined by at least three independent western blot experiments as in **(B)**. The results are expressed as means ± SEM. Q7 = 1,4-dihydroxy quininib; h = hours.

**Supplementary Dataset 2.** Dataset containing the results from the proteome-profiling of OMM2.5 cells after the multiple testing corrections. The q-value is intended to be analogous to the p-value but takes into account multiple testing corrections.
